# The PAXT connection is a modifier of rare codon-enriched protein expression

**DOI:** 10.1038/s41467-026-77792-7

**Published:** 2026-09-17

**Authors:** Taylor Niehoff, Jackson Peterson, Siqi Li, Sijin Chen, Christopher V. Nicchitta, Alain Laederach, Christopher M. Counter

**Affiliations:** 1 Department of Pharmacology & Cancer Biology, Duke University Medical Center, Durham, NC USA; 2 Department of Biology, University of North Carolina at Chapel Hill, Chapel Hill, NC USA; 3 Department of Cell Biology, Duke University Medical Center, Durham, NC USA

**Keywords:** Transcription, Translation, CRISPR-Cas systems, RNA metabolism

## Abstract

Synonymous codons occur at unequal rates in the coding genome, with those represented least often termed ‘rare.’ Rare codon-enriched genes typically exhibit poor expression due to reduced transcription, mRNA stability, export, and translation. However, there are an increasing number of examples whereby rare codon-enriched genes are paradoxically highly expressed, suggestive of mechanisms to overcome the poor expression imposed by rare codons. To identify codon-dependent modifiers, we screen a targeted sgRNA library for genes that, when inactivated, increase the expression of rare versus common codon-enriched fluorescent reporters. This identifies the PAXT connection, a nuclear complex that targets transcripts to the exosome for degradation. Targeted loss of a PAXT component leads to a small but preferential increase in mRNA levels and stability and a more pronounced increase in protein expression of rare compared to common codon-enriched reporters. Transcriptomics coupled to proteomics further reveals that this leads to a preferential increase in the expression of endogenous proteins encoded by poorly expressed and long mRNAs specifically enriched in rare codons. Thus, we identify the PAXT connection as a genetic suppressor of poorly expressed genes enriched in rare codons.

## Abstract (web summary)

Rare codons are generally associated with reduced gene expression, but mechanisms that enable efficient expression of rare codon-enriched genes remain unclear. Here, the authors identify the PAXT connection as a suppressor of rare codon-enriched transcripts, whose loss preferentially increases their stability and protein expression.

## Introduction

There is redundancy in the genetic code, whereby all but two amino acids are encoded by more than one codon, with codons encoding the same amino acid termed “synonymous.”^1^ Synonymous codons occur at unequal rates in the coding genome, with those most represented termed “common” and those least represented termed “rare.”^2–7^ Genes enriched in rare codons tend to be expressed more poorly^2–4,7^. In humans, this poor expression has been shown to occur at multiple levels. At the gene level, a bias of A/T nucleotides at the 3′ position of rare codons has been linked to a decrease in activating histone marks^8^. At the transcript level, the same bias can result in reduced transcription and abundance of the encoded mRNA^9,10^. Such transcripts can also be less stable and more poorly exported from the nucleus^11,12^. At the protein level, rare codon bias can impede translation, potentially as the cognate tRNAs are underrepresented, which can lead to ribosome pausing^8,13–15^, impeding translation initiation and leading to RNA decay^16,17^.

There is an increasing number of examples throughout evolution whereby rare codon-enriched genes are paradoxically highly expressed. In viruses, some viral genomes have a bias towards the rare codons of their host, yet their genes are highly expressed^18–21^. Infection of these viruses can also preferentially increase expression of host genes enriched in rare codons^18,19^. In insects, a GFP reporter enriched in rare codons was found to be highly expressed in the brain and testis of *Drosophila* larvae^22^. In humans, ectopic *KRAS* is expressed at high levels in some cancer cell lines, despite being enriched in rare codons^8,15^. A number of mechanisms have been suggested to account for this reversal in expression. For example, infection by the human virus CHIKV was shown to increase expression of the host gene *TRMT9B*, which encodes the putative tRNA methyltransferase 9B. This led to increased conversion of 5-carboxymethyl to 5-methoxy-carbonyl-methyl on uridines at the wobble position in tRNAs and, in turn, increased decoding of specific rare codons through wobble pairing. The end result was higher expression of human transcripts enriched in rare codons^19^. Inactivation of the *Drosophila* gene *orb2*, which encodes an RNA-binding protein, reduced the stability of a rare codon-enriched reporter transcript in neurons, leading to reduced protein expression^23^. The human virus HIV-1 has a bias towards rare codons of the host and specifically packages rare codon-decoding tRNA in the virions to promote viral translation^20^. Interestingly, as part of the host antiviral defense, the human protein Schlafen 11 binds and sequesters these tRNAs^24^. Collectively, these findings prompted us to ask if there may be other genes that affect protein levels in a rare codon-dependent manner.

Here we report a screen for genes affecting codon-dependent expression that identified two subunits and accessory proteins of the poly(A) tail exosome targeting (PAXT) connection as suppressors of rare codon-enriched expression. The core of this complex is composed of three subunits: polyadenylate-binding protein nuclear 1 (PABPN1), an RNA-binding protein that associates with the poly(A) tail of mRNA^25^; MTR4 exosome RNA helicase (MTREX), an RNA helicase that unwinds RNA for degradation^26^; and zinc finger C3H1-type containing protein (ZFC3H1), a zinc-finger protein that associates with both MTREX and the exosome^27,28^. While PABPN1 and MTREX are components of the polyadenylation^25^, NEXT^29^ and other complexes, ZFC3H1 is specific to PAXT^27^. Functionally, PAXT is involved in nuclear RNA surveillance via binding to and transporting defective or export-incompetent polyadenylated transcripts to the nuclear exosome for degradation^27,28,30^. Mechanistically, it has been proposed that PABPN1 binds polyadenylated transcripts, which in turn recruits ZFC3H1 and then MTREX to unwind the RNA, followed by degradation via the nuclear exosome^27,28,31,32^. Alternatively, ZFC3H1 may bind to RNA when transcribed in competition with splicing and export machinery. If ZFC3H1 outcompetes these proteins, it recruits RBM26/27 and ZC3H3^32^, which causes ZFC3H1 to adopt an open conformation. In this open conformation, ZFC3H1 can recruit MTREX to initiate the degradation process^33^. Thus, PAXT appears to have another role, namely as a suppressor of rare codon-dependent gene expression.

## Results

### A CRISPR/Cas9 screen identifies components of the PAXT connection as suppressors of rare codon-dependent expression

**Fig. 1:**
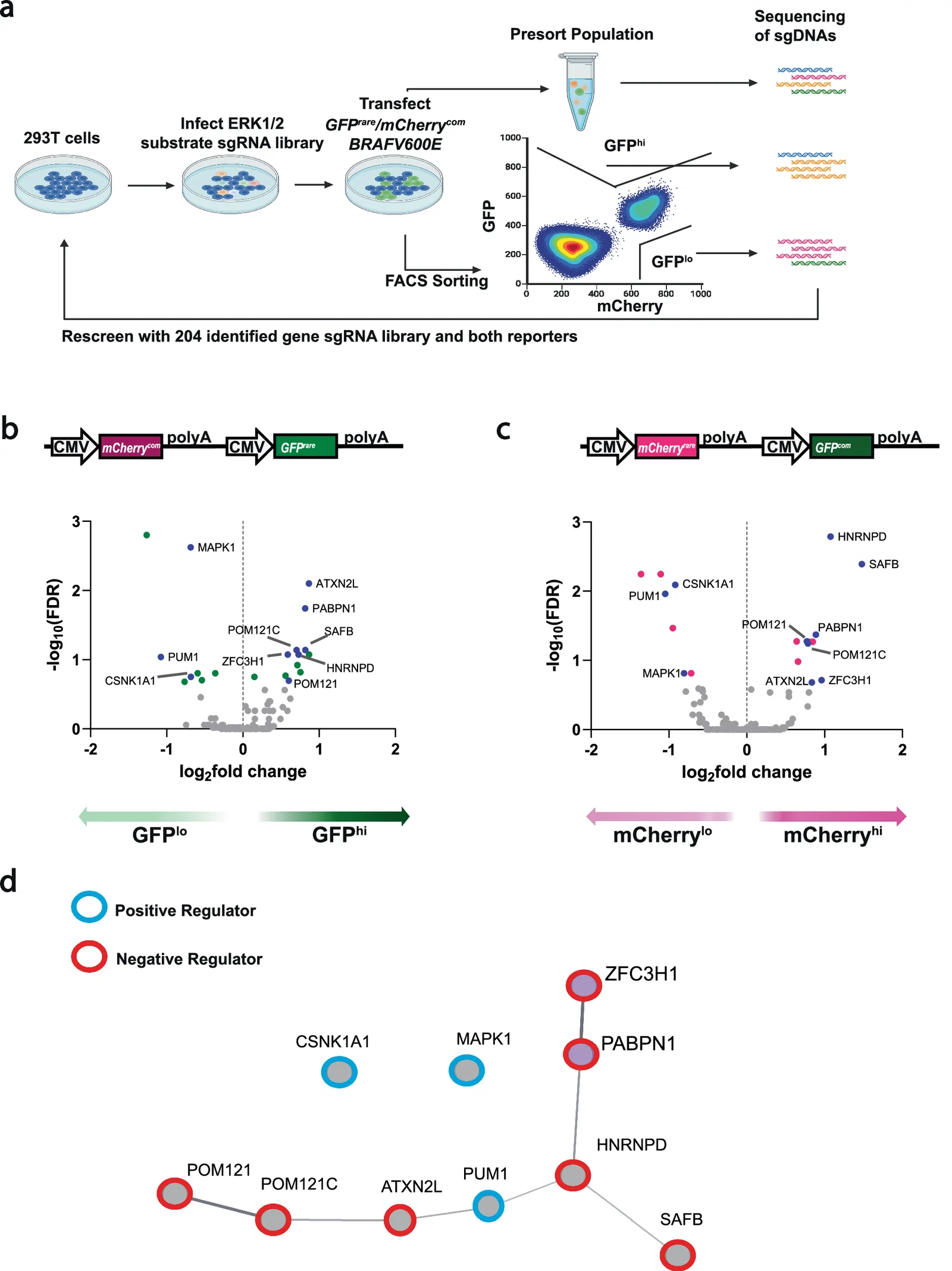
A loss-of-function screen for modifiers of rare codon-dependent expression. **a** Schematic of the screen (FACS plot is not actual data and is for description purposes only, partially created in BioRender. Niehoff, T. (2026) https://BioRender.com/buma0d3). Volcano plot of the most significant log_10_ FDR versus log_2_-fold difference of sgRNA abundance (in either hi or lo populations compared to the other at 14 days) for each of the 204 genes in BRAF^V600E^-expressing 293T cells transduced with the second 204-gene sgRNA library, and either the **b** GFP^rare^/mCherry^com^ or **c** mCherry^rare^/GFP^com^ dual reporter, followed by FACS analysis to separate cells in the top and bottom populations based on difference in fluorescence between the two reporters. Cells in which sgRNAs reached an FDR < 0.25 using only the GFP^rare^/mCherry^com^, mCherry^rare^/GFP^com^, or both reporters are shown as green, pink, or blue circles, respectively, with the target genes identified in the latter. Top: reporter construct used. *n* = 3 biological replicates per screen. **d** STRING analysis of the candidate genes identified from the screen (FDR < 0.25 with both reporters) that were negative (red outline) or positive (blue outline) modifiers of rare codon-dependent expression. Source data are provided as a Source Data file.

To screen for modifiers of rare codon-dependent expression, 293T cells were stably infected with a CRISPR/Cas9 lentiviral sgRNA library targeting known or putative ERK1/2 substrates. These cells were then co-transfected with a reporter vector that dually expresses green fluorescent protein (GFP) encoded by rare codons (*GFP*^*rare*^) and red fluorescent mCherry encoded by common codons (*mCherry*^*com*^) and a vector expressing oncogenic BRAF^V600E^ (Fig. 1a). The human 293T cell line was chosen because these cells are both commonly used for screens and express GFP^rare^ less than mCherry^com^ protein^34^. The ERK1/2 substrate sgRNA library was chosen because ERK1/2 are the terminal kinases in the MAPK pathway, which was previously shown to preferentially increase expression of GFP^rare^ compared to mCherry^com^ protein^34^. This library was designed with five independent sgRNAs targeting 1223 human genes^35,36^. As controls, the library also contained 50 randomly selected sgRNAs with no known targets in the human genome, sgRNAs targeting ten human ribosomal genes essential for viability, and sgRNAs targeting genes encoding proteins within the MAPK pathway. The vector expressing oncogenic BRAF^V600E^ was chosen to stimulate the MAPK pathway. Finally, the GFP^rare^/mCherry^com^ dual reporter was chosen as it co-expresses two different fluorescent proteins encoded with rare versus common codons, each individually driven by a CMV promoter^34^. In more detail, this dual reporter is comprised of a CMV promoter, *GFP*^*rare*^ cDNA, a polyadenylation (poly(A)) sequence, another CMV promoter, *mCherry*^*com*^ cDNA, and another poly(A) sequence. Relative Synonymous Codon Usage (RSCU) was engineered to favor rare codons in 136 of the 239 codons encoding *GFP*^*rare*^ and to favor common codons in 209 of the 236 codons encoding *mCherry*^*com*^ (Supplementary Fig. 1). Further metrics of codon bias, GC%, GC3%, codon adaptation index (CAI)^37^, and tRNA adaptation index (tAI)^38^, were also highly different between *GFP*^*rare*^ and *mCherry*^*com*^ (Supplementary Table 1). To mitigate possible position effects^12,39,40^, codon bias in both reporters was designed to be uniform throughout their cDNAs (Supplementary Fig. 1)^34^. Finally, to ensure reproducibility and rigor, 293T cells were infected and transfected in triplicate. The three replicate cultures were selected and then cultured for 7 or 14 days, after which a “presort” population was removed as a control, and the remaining cells were subjected to fluorescence-activated cell sorting (FACS) analysis for green and red fluorescence. GFP^lo^ and GFP^hi^ populations were isolated by drawing gates above and below an expression line generated by luciferase-transfected control cells. Genomic DNA was then isolated from the presort, GFP^lo^, and GFP^hi^ populations, DNA encoding sgRNA sequences was PCR amplified and indexed, and the resultant library was subjected to paired-end next-generation sequencing (Fig. 1a). This identified 203 genes for which the corresponding sgRNA sequences were significantly enriched (false-discovery rate, FDR < 0.26) in either the GFP^hi^- or GFP^lo^-sorted cells, compared to either the other population or the presorted cells, at either 7- or 14-days post-selection (Supplementary Data 1).

To refine the number of putative targets, we generated a second smaller CRISPR/Cas9 sgRNA lentiviral library comprised of five independent guides targeting the identified 203 genes and *BRAF* as a control (Supplementary Data 1), as well as the aforementioned control guides, and an additional 100 non-targeting control sgRNAs. This library was screened as above with the GFP^rare^/mCherry^com^ dual reporter. To rule out reporter-specific modifiers, the screen was also performed in parallel with an inverse dual reporter comprised of *mCherry* cDNA encoded by rare codons (*mCherry*^*rare*^) and *GFP* cDNA encoded by common codons (*GFP*^*com*^) (Supplementary Table 1). To ensure reproducibility and rigor, 293T cells were infected with this sgRNA library and then co-transfected with either of the dual reporters and the BRAF^V600E^-expressing vector in triplicate, after which the cells were cultured as above and the presort, GFP^lo^, and GFP^hi^ populations subjected to sequencing (Fig. 1a). This secondary screen identified ten genes with significant enrichment (FDR < 0.25) in both the GFP^hi^- or GFP^lo^- (Fig. 1b) and mCherry^hi^- or mCherry^lo^- (Fig. 1c) sorted cells, compared to either the other population or the presorted cells at 14 days post-selection, or 7 days post-selection (Supplementary Data 2).

Analysis by Search Tool for the Retrieval of Interacting Genes/Proteins (STRING)^41^ grouped eight of the proteins encoded by these ten genes into one cluster, with the remaining two being *CSNK1A1* and *MAPK1,* encoding casein kinase 1α1 and ERK2 (Fig. 1d). Of note, identification of ERK2 independently validates the screen, as the MAPK pathway was previously identified as a positive regulator of GFP^rare^ protein expression^34^. The eight-member cluster contains the genes zinc finger C3H1-type containing protein (*ZFC3H1)*, polyadenylate-binding protein nuclear 1 (*PABPN1*), heterogeneous nuclear ribonucleoprotein D *(HNRNPD)*, ataxin 2 like *(ATXN2L)*, Pumilio RNA binding family member *1 (PUM1)*, Scaffold Attachment Factor B *(SAFB)*, POM121 transmembrane nucleoporin (*POM121)*, and POM121 transmembrane nucleoporin C (*POM121C)*, all of which are involved in RNA binding, catabolism, and/or nuclear export^11,27,42–45^. Of these eight, *ZFC3H1* and *PABPN1* stood out as the most promising candidates for two reasons. First, ZFC3H1 and PABPN1 proteins comprise two out of the three core subunits of the poly(A) tail exosome targeting (PAXT) connection^27^, with the remaining subunit, MTREX, not targeted in the sgRNA library. Second, molecular function Gene Ontology (GO) analysis of these ten genes revealed that two of the five most commonly enriched terms were 'regulation of mRNA metabolic process' and 'mRNA destabilization' (Supplementary Table 2), terms of interest as PAXT targets transcripts to the exosome for degradation^27,28^. Thus, we focused on the PAXT connection.

### ZFC3H1 loss preferentially increases rare codon-enriched reporter protein levels

**Fig. 2:**
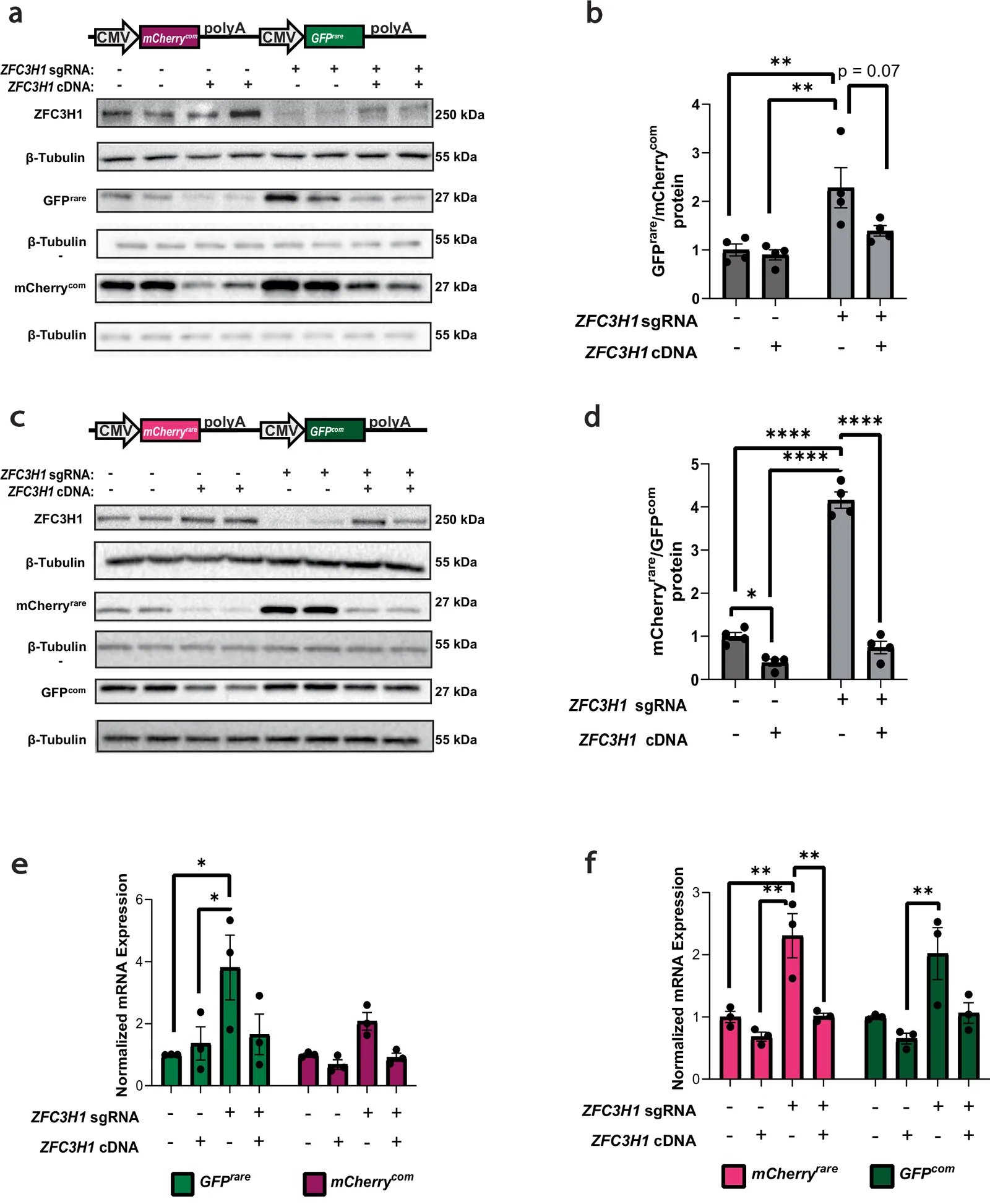
The effect of ZFC3H1 loss on reporter protein and mRNA levels. **a**, **c** Examples of immunoblots (2 of 4 biological replicates) for the indicated proteins (right: predicted molecular weight of the indicated protein), **b**, **d** the derived mean ± SEM of the ratio of the indicated reporter protein levels (normalized to β-tubulin and *Ctrl* sgRNA cells), or **e**, **f** the mean ± SEM of mRNA levels, as assessed by RT-qPCR (normalized to *18S* rRNA and *Ctrl* sgRNA cells) of 293T cells transduced with vectors encoding *Ctrl* or *ZFC3H1* sgRNA, no transgene or *ZFC3H1* cDNA, and the **a**, **b**, **e** GFP^rare^/mCherry^com^ or **c**, **d**, **f** mCherry^rare^/GFP^com^ dual reporter. Black circles indicate biological replicates (**b**, **d** *n* = 4; **e**, **f** *n* = 3). Two-way ANOVA and Tukey’s post hoc test was performed for **b** (F ratio = 7.6302, *p* = 0.0041, *n* = 4 biological replicates) and **d** (F ratio = 171.65, *p* < 0.0001, *n* = 4 biological replicates). Three-way ANOVA analysis with Tukey’s post hoc test was performed for **e** (F ratio = 4.2529, *p* = 0.0078, *n* = 3 biological replicates) and **f** (F ratio = 8.3921, *p* = 0.0002, *n* = 3 biological replicates). *p < 0.05, **p < 0.01, and ****p < 0.0001. Different exposure times were required for each antibody (see uncropped images in Supplementary Data 5). Source data are provided as a Source Data file.

To independently evaluate PAXT as a modifier of rare codon-dependent expression, we focused on ZFC3H1, as it is the only one of the three core subunits known to be unique to the PAXT connection^27^. Of the other two subunits, PABPN1 is also involved in polyadenylation regulation^25^ and RNA export^46^, while MTREX is also a component of the NEXT complex involved in degrading non-polyadenylated RNA^29^. To this end, 293T cells were stably infected independently with a lentivirus encoding Cas9 and either one of two independent *ZFC3H1* sgRNAs or one of two independent non-targeting control (*Ctrl*) sgRNAs. Thus, four independent (two different sgRNAs tested twice) experimental and control biological replicates were generated to ensure reproducibility and rigor. These eight cell lines were then co-transfected with the aforementioned GFP^rare^/mCherry^com^ dual reporter and a vector encoding either no transgene or a FLAG epitope-tagged *ZFC3H1* cDNA designed to be resistant to both *ZFC3H1* sgRNAs. Immunoblot analysis with an anti-ZFC3H1 antibody of lysates collected from the resultant 16 cell cultures confirmed reduced ZFC3H1 protein levels by both *ZFC3H1* sgRNAs and restoration thereof in cells transfected with the ZFC3H1 expression vector. Immunoblot analysis with an anti-GFP antibody revealed an increase in GFP^rare^ protein when ZFC3H1 expression was reduced compared to *Ctrl* cells, supporting ZFC3H1 as a suppressor of rare codon-dependent expression. This effect was reversed upon restoring ZFC3H1, with ZFC3H1 expression alone even reducing GFP^rare^ levels, suggestive of a potential dominant-negative effect (Fig. 2a and Supplementary Fig. 2a). Quantification of the four biological replicate experiments, when normalized to tubulin levels, revealed a significant 3.5-fold average increase in GFP^rare^ protein levels upon reducing ZFC3H1 expression (Supplementary Fig. 2b). Immunoblot analysis with an anti-mCherry antibody revealed a similar trend, although these effects were less pronounced (Fig. 2a and Supplementary Fig. 2a). Calculating the ratio of GFP^rare^ to mCherry^com^ protein level revealed a significant ~2-fold average increase upon reducing ZFC3H1 expression, which was reversed by re-expressing ZFC3H1 (Fig. 2b), thereby confirming the original identification of ZFC3H1 from the fluorescence screen by an independent assay. To validate these findings, the identical experiment was repeated with the mCherry^rare^/GFP^com^ dual reporter. Reducing ZFC3H1 expression, as confirmed by immunoblot, also significantly increased mCherry^rare^ protein levels compared to GFP^com^, and again, this effect was reversed by re-expressing ZFC3H1, although in this case the effect of ZFC3H1 loss on GFP^com^ levels was negligible (Fig. 2c and Supplementary Fig. 2c, d). Calculating the ratio of mCherry^rare^ to GFP^com^ revealed a statistically significant increase upon reducing ZFC3H1 to an even greater degree (~4-fold on average) compared to the GFP^rare^ to mCherry^com^ ratio, which was similarly reversed by re-expressing ZFC3H1 (Fig. 2d).

To independently validate these findings using a more quantitative approach, 293T cells were again stably infected in triplicate with the lentiviruses encoding one of two independent *Ctrl* or *ZFC3H1* sgRNAs, then co-transfected with the vectors encoding no transgene or *ZFC3H1* cDNA and the GFP^rare^/mCherry^*com*^ dual reporter. The resultant cells were split in half. One half was subjected to immunoblot analysis at increasing amounts of total protein lysate loaded. This revealed a larger increase in GFP^rare^ compared to mCherry^com^ protein expression upon reducing ZFC3H1 expression, which was reversed by re-expressing ZFC3H1. Moreover, this increase was observed at all tested amounts (Supplementary Fig. 3a). The other half of the cells was subjected to flow cytometry analysis, which revealed a 1.6-fold significant average increase in the ratio of GFP^rare^ to mCherry^com^ fluorescence upon reducing ZFC3H1 expression, which again was reversed by re-expressing ZFC3H1 (Supplementary Fig. 3b and Supplementary Data 3). Thus, two different assays demonstrate that reducing ZFC3H1 expression preferentially increases protein levels of a rare codon-enriched reporter.

The changes in rare codon-enriched protein levels upon ZFC3H1 loss could point to either global changes in translation that rare codon-enriched transcripts are more sensitive to or a preferential increase in expression of these reporters. To determine if the increase in rare codon-enriched protein expression was caused by global changes to translation levels upon ZFC3H1 loss, we performed a puromycin surface sensing of translation (SUnSET) assay, which measures puromycin incorporation into actively translated peptides^47^. As controls, we demonstrate that addition of puromycin to 293T cells resulted in labeled proteins, as detected by immunoblot with an anti-puromycin antibody, and further, this incorporation was blocked by pre-incubation with cycloheximide, a translation inhibitor (Supplementary Fig. 4a). In parallel, 293T cells were stably infected in quadruplicate with a lentivirus encoding one of two independent *Ctrl* or *ZFC3H1* sgRNAs or transfected in triplicate with either *Ctrl* or *ZFC3H1* siRNA, similarly incubated with puromycin, and analyzed for puromycin incorporation by immunoblot (Supplementary Fig. 4b). Inactivation of ZFC3H1 by either sgRNA or siRNA did not significantly change puromycin incorporation compared to *Ctrl* cells (Supplementary Fig. 4c). Thus, the loss of ZFC3H1 does not significantly change the rate of global protein synthesis.

### ZFC3H1 loss increases reporter mRNA levels

Given that the PAXT connection is known to target mRNA to the nuclear exosome for degradation^27,28,32^, we measured the levels of reporter mRNAs upon reducing ZFC3H1 expression. Namely, at the same time we collected lysates from the above 32 cell cultures for immunoblot analysis (Fig. 2a, c, and Supplementary Fig. 2a, c), we also collected lysates for RNA analysis, although we reduced the number of biological replicates tested from four to three. mRNA levels were determined by quantitative reverse-transcription PCR (RT-qPCR), normalized to *18S* rRNA levels, and expressed as a ratio of that detected in *Ctrl* sgRNA cells. Quantitation of all three biological replicates revealed that, on average, *GFP*^*rare*^ mRNA levels significantly increased 3.5-fold upon the reduction of ZFC3H1, and that re-expressing ZFC3H1 trended towards reversing this effect. On the other hand, loss of ZFC3H1 did not lead to a significant increase in *mCherry*^*com*^ mRNA levels, although the level did trend towards an increase (Fig. 2e). In the case of the mCherry^rare^/GFP^com^ dual reporter, loss of ZFC3H1 increased both *mCherry*^*rare*^ and *GFP*^*com*^ mRNA to a similar degree, which was also reversed by re-expressing ZFC3H1 (Fig. 2f). Due to differences in the degree that the loss of ZFC3H1 affected the two reporters, 293T cells were again infected in triplicate with lentivirus encoding one of two independent *Ctrl* or *ZFC3H1* sgRNAs after which the loss of ZFC3H1 was confirmed by immunoblot (Supplementary Fig. 5a). These six stable cell lines were then co-transfected with both the GFP^rare^/mCherry^com^ and mCherry^rare^/GFP^com^ dual reporters together to compare mRNA levels in the same cells. The levels of all four reporter transcripts significantly increased upon the loss of ZFC3H1, with a slight trend towards a greater increase in rare codon-enriched transcripts, although admittedly this did not reach significance (Supplementary Fig. 5b). Lastly, we repeated these experiments, using siRNA depletion rather than CRISPR/Cas9 loss, to control for potential off-target effects at the genome level (Supplementary Fig. 6a), which revealed a similar trend. Namely, reducing ZFC3H1 trended towards an increase in mRNA (Supplementary Fig. 6b) and especially protein (Supplementary Fig. 6c–e) levels of rare codon-enriched reporters compared to their common counterparts. Taken together, the loss of ZFC3H1 led to an increase in all reporter mRNA, but with a variable and small preference for the rare codon-enriched reporters.

### The effect of ZFC3H1 loss on mRNA stability and export

**Fig. 3:**
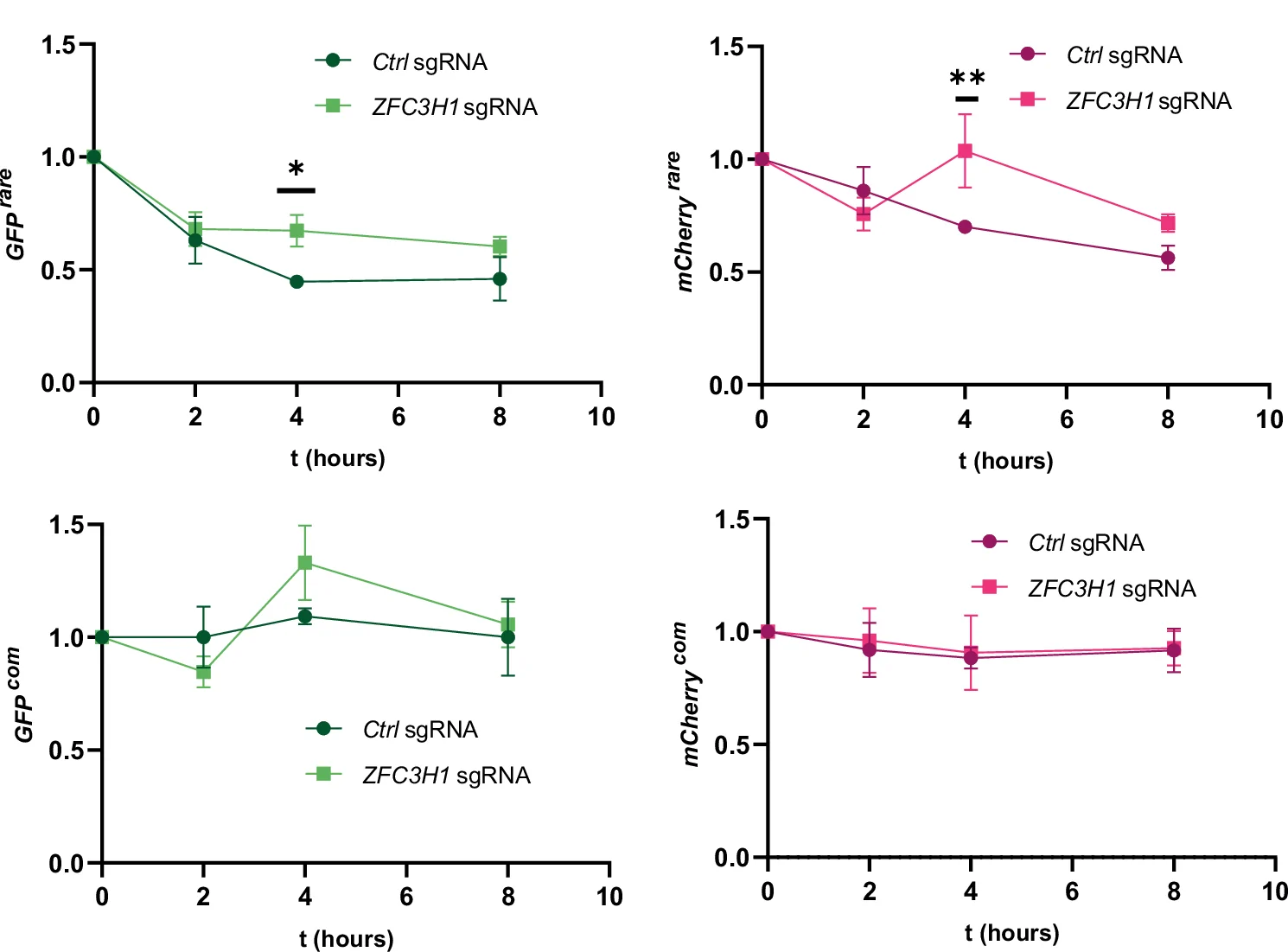
The effect of ZFC3H1 loss on reporter mRNA stability. Mean ± SEM of the indicated reporter mRNA, as assessed by RT-qPCR, in *Ctrl* and *ZFC3H1* sgRNA cells co-transfected with both GFP^rare^/mCherry^com^ and mCherry^rare^/GFP^com^ dual reporters after treatment with Actinomycin D for the indicated time (*t*), normalized to *18S* rRNA and *t* = 0 values for each replicate from *n* = 3 biological replicate experiments. Two-way ANOVA was performed for each reporter (*GFP*^*rare*^: F ratio = 10.9559, *p* < 0.0001, *n* = 3 biological replicates; *mCherry*^*rare*^: F ratio = 4.9947, *p* = 0.0037, *n* = 3 biological replicates; *GFP*^*com*^: F ratio = 1.6427, *p* = 0.1941, *n* = 3 biological replicates; *mCherry*^*com*^: F ratio = 0.1861, *p* = 0.9844, *n* = 3 biological replicates) with sliced two-tailed Student’s *t* test post hoc for each time point. *p < 0.05 and **p < 0.01. Source data are provided as a Source Data file.

Although less pronounced and more variable than at the protein level, the loss of ZFC3H1 showed a trend towards a preferential increase in the mRNA levels of the rare codon-enriched reporters. Given that the canonical function of PAXT is to target polyadenylated RNA to the exosome for degradation^27,28,32^, we hypothesized that loss of PAXT may stabilize rare codon-enriched transcripts. Thus, we measured the effect of ZFC3H1 loss on the stability of reporter mRNA^48^. 293T cells were again stably infected with either one of two independent *Ctrl* or *ZFC3H1* sgRNAs and then co-transfected with both the GFP^rare^/mCherry^com^ and mCherry^rare^/GFP^com^ dual reporters together. The six resultant cultures were treated with Actinomycin D for 0, 2, 4, and 8 h before being subjected to RT-qPCR and immunoblot analysis. As expected^11,49^, both rare codon-enriched reporter transcripts were less stable than their common counterparts in the *Ctrl* cells. However, in *ZFC3H1* sgRNA cells in which ZFC3H1 protein levels were confirmed to be reduced by immunoblot analysis (Supplementary Fig. 7a), the stability of the rare, but not common, codon-enriched reporter transcripts significantly increased after 4 h of Actinomycin D treatment (Fig. 3 and Supplementary Fig. 7b). To validate these results, we both repeated the experiment with three newly generated *Ctrl* or *ZFC3H1* sgRNA (from one of two independent sgRNAs for each) stable cell lines, the latter confirmed to have reduced ZFC3H1 protein levels (Supplementary Fig. 8a), and included analysis of *SNHG19* lncRNA, a known PAXT target, as a positive control. As previously published^27^, *SNHG19* lncRNA was significantly more stable 4–8 h post Actinomycin D treatment upon the loss of ZFC3H1 (Supplementary Fig. 8b). Loss of *ZFC3H1* again led to a small stabilization of rare, but not common, codon reporter transcripts either significantly after 2 h of Actinomycin D treatment in the case of *GFP*^*rare*^ or trended towards an increase at all time points in the case of *mCherry*^*rare*^, albeit non-significantly (Supplementary Fig. 8c), confirming the preferential effects of ZFC3H1 depletion on rare codon-enriched reporter mRNA stability.

**Fig. 4:**
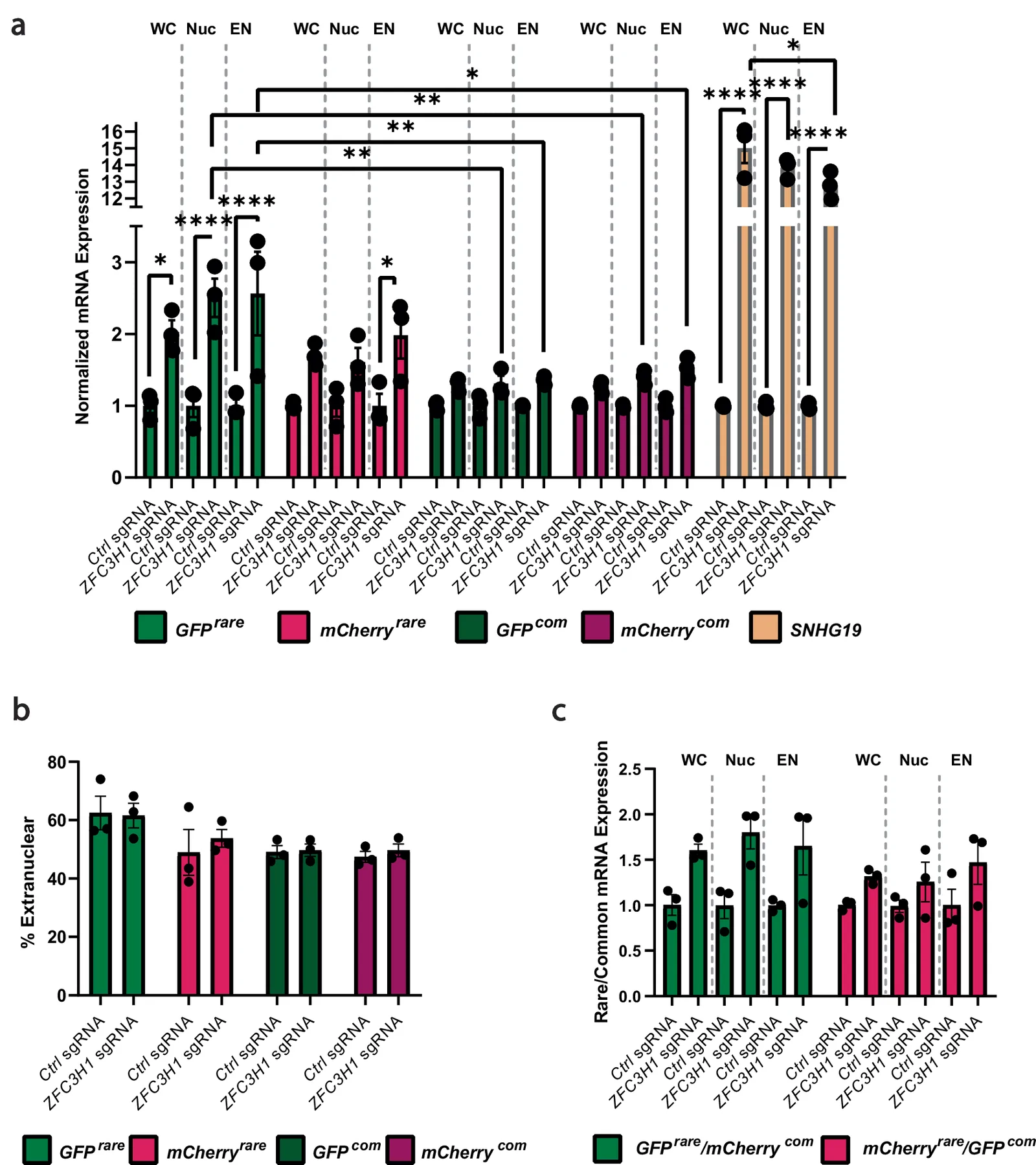
The effect of ZFC3H1 loss on reporter mRNA export. Mean ± SEM of the **a** indicated reporter mRNA and *SNHG19* levels, as assessed by RT-qPCR, in whole cell (WC), nuclear (Nuc), or extranuclear (EN) fractions, **b** derived percent extranuclear transcript (en/en+nuc X 100) of the indicated reporter mRNA, and **c** derived ratio of the indicated reporter mRNA levels from *Ctrl* and *ZFC3H1* sgRNA cells co-transfected with both GFP^rare^/mCherry^com^ and mCherry^rare^/GFP^com^ dual reporters and subsequently subjected to detergent fractionation, normalized to *GAPDH* mRNA and *Ctrl* sgRNA cells from *n* = 3 biological replicate experiments. Three-way ANOVA analysis with Tukey’s post hoc test was performed for reporter mRNA in **a** (F ratio = 7.6809, *p* < 0.0001, *n* = 3 biological replicates) and two-way ANOVA analysis with Tukey’s post hoc test was performed for *SNHG19* in **a** (F ratio = 255.9494, *p* < 0.0001, *n* = 3 biological replicates). Welch’s T-tests was performed for each reporter in **b**. Three-way ANOVA analysis with Tukey’s post hoc test was performed in **c** (F ratio = 3.5304, *p* = 0.0047, *n* = 3 biological replicates). *p < 0.05, **p < 0.01, and ****p < 0.0001. Source data are provided as a Source Data file.

Given that the PAXT connection has also been shown to retain transcripts in nuclear foci^28,30,50,51^, we measured the level of reporter mRNAs in the nuclear versus extranuclear fractions upon reducing ZFC3H1 expression. 293T cells were again stably infected (Supplementary Fig. 9a) and co-transfected as above, after which whole-cell, nuclear, and extranuclear (includes both cytoplasm and endoplasmic reticulum compartments) lysates from detergent fractionation^52^ were collected from the 12 resultant cultures. We confirmed the purity of each fraction by immunoblot analysis^52^. Namely, Lamin B1 (nuclear marker) was enriched in the nuclear fractions, while SSR1 (endoplasmic reticulum marker) and β-tubulin (cytoplasmic marker) were enriched in the extranuclear fractions (Supplementary Fig. 9b). RNA integrity was examined by assessing *28S* and *18S* rRNA via the bleach agarose gel method^53^, which revealed intact bands for both rRNAs, as well as higher *28S* rRNA signal, indicative of intact RNA, in all fractions and replicates (Supplementary Fig. 9c). Given the purity and rRNA stability in these fractions, we next measured the levels of reporter mRNAs and positive-control *SNHG19* lncRNA by RT-qPCR in all three fractions. The level of *SNHG19* lncRNA was significantly increased in both the nuclear and extranuclear fractions with no significant difference in these increases between the two fractions, validating the known stabilization of RNA upon disrupting the PAXT connection (Fig. 4a). Examining reporter mRNA levels revealed the above trend of an increase in mRNA levels of all reporters upon the loss of ZFC3H1, with *GFP*^*rare*^ and *mCherry*^*rare*^ mRNA preferentially and significantly increased in the extranuclear fraction compared to their common counterparts (Fig. 4a). As with *SNHG19*, there was no significant difference in the increases in reporter mRNA between the two fractions upon ZFC3H1 depletion (Fig. 4a), and consistently, the percent of each reporter mRNA transcript in the extranuclear fraction did not significantly change with ZFC3H1 loss (Fig. 4b). With both reporters, ZFC3H1 loss trended towards a greater increase in rare codon-enriched reporter mRNA compared to their common counterparts from the same reporter across both fractions (Fig. 4c). Thus, ZFC3H1 loss resulted in a preferential increase in the stability and extranuclear levels of the rare codon-enriched reporter mRNAs compared to the common codon-enriched reporter mRNAs.

### The increase in reporter protein upon ZFC3H1 loss is influenced by mRNA levels

**Fig. 5:**
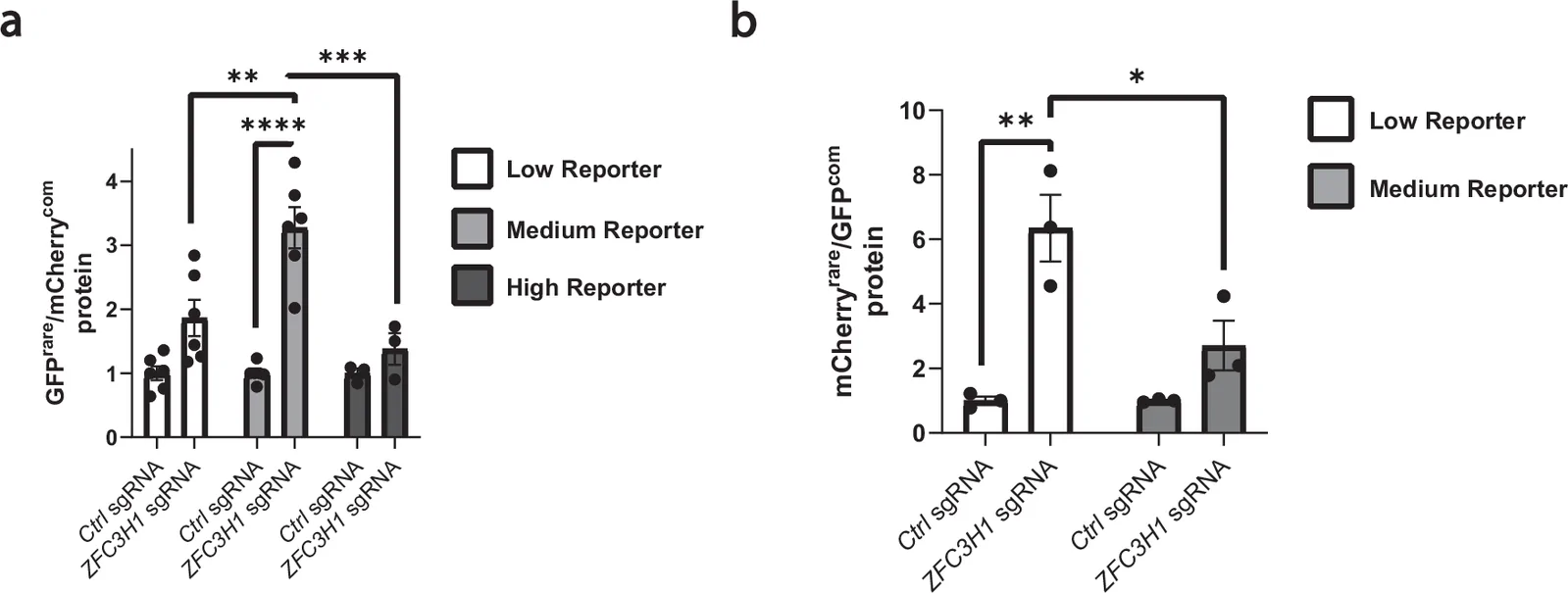
The effect of ZFC3H1 loss on reporter protein levels in cells transduced with increasing amounts of the dual reporter plasmid. The derived mean ± SEM of the ratio of levels of **a** GFP^rare^ versus mCherry^com^ or **b** mCherry^rare^ versus GFP^com^ protein, as assessed by immunoblot, of lysates isolated from 293T cells transduced with vectors encoding *Ctrl* or *ZFC3H1* sgRNA and low (0.4 µg), medium (1.0 µg), or high (2.0 µg) amounts of the **a** GFP^rare^/mCherry^com^ or **b** mCherry^rare^/GFP^com^ dual reporter, derived from *n* = 3 to 6 biological replicate experiments (black circles). Two-way ANOVA with Tukey’s post hoc test was performed for **a** (F ratio = 17.1394, *p* < 0.0001, *n* = 3 (high) or 6 (low, medium) biological replicates) and **b** (F ratio = 15.1788, *p* = 0.0012, *n* = 3 biological replicates). *p < 0.05, **p < 0.01, ***p < 0.001, and ****p < 0.0001. Source data are provided as a Source Data file.

The loss of ZFC3H1 preferentially increased rare over common codon reporters more consistently at the protein compared to the mRNA level. Related, disrupting one allele of the *Drosophila* gene *Rps21* was previously shown to preferentially increase protein expression of an inducible *Ras85* transgene when encoded by rare compared to common codons. However, reducing the expression of *Ras85* encoded by common codons rendered the transgene sensitive to the effect of Rps21 disruption. These results suggest that low expression levels promote the preferential increase of the rare codon transgene upon the loss of Rps21^54^. Notably, we also saw a large difference in expression of the rare- and common codon-enriched reporters in control conditions. In more detail, both common reporter transcripts were expressed an average of ~15 times higher than their rare counterparts (Supplementary Fig. 10a), with the caveat that different qPCR primers were utilized for each transcript. At the protein level, we also observed that mCherry^com^ fluorescence was, on average, ~15-fold higher than GFP^rare^ fluorescence (Supplementary Fig. 10b). To test if changing the expression of the reporters influences the preferential increase in rare codon-enriched reporter transcript levels upon the loss of ZFC3H1, we stably infected 293T cells in triplicate with a lentivirus encoding one of two independent *Ctrl* or *ZFC3H1* sgRNAs, followed by transfection of increasing amounts of the GFP^rare^/mCherry^com^ (Supplementary Fig. 11a, b) or mCherry^rare^/GFP^com^ (Supplementary Fig. 11c) dual reporter. As above, we determined the ratio of rare to common reporters in *ZFC3H1* versus *Ctrl* sgRNA cells. This revealed that the ratio of GFP^rare^/mCherry^com^ increased with more plasmid upon the loss of ZFC3H1, an effect nullified at the highest amount of dual reporter transfected into cells (Fig. 5a). In the case of the mCherry^rare^/GFP^com^ dual reporter, the loss of ZFC3H1 was similarly less efficient at increasing the ratio of these two proteins at an even lower amount of dual reporter transfected into cells (Fig. 5b). Again, while loss of ZFC3H1 resulted in an increase in reporter mRNA levels and some expression level differences, the changes in mRNA with varying amounts of reporter were small compared to the changes at the protein level (Supplementary Fig. 12a, b). Thus, the preferential increase in rare compared to common codon-enriched reporter protein upon loss of ZFC3H1 was lessened by increasing the amount of the reporters.

### PABPN1 loss phenocopies ZFC3H1 loss and preferentially binds to the rare codon-enriched reporter transcripts

**Fig. 6:**
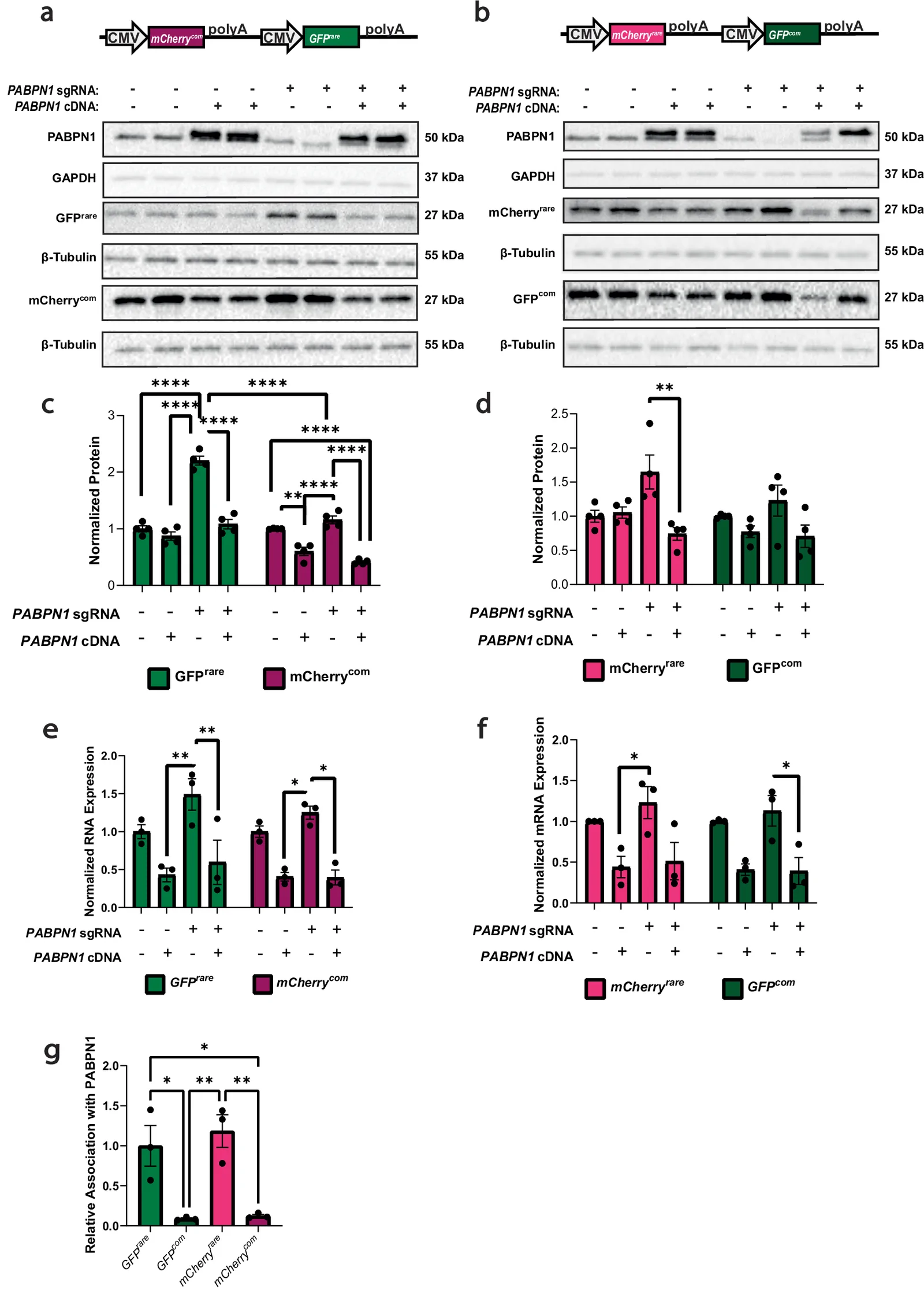
The effect of PABPN1 loss on reporter mRNA and protein levels and association with reporter mRNA. **a**, **b** Examples of immunoblots (2 of 4 biological replicates) of the indicated proteins (right: predicted molecular weight of the indicated protein) and **c**–**f** mean ± SEM of the quantification of the indicated reporter **c**, **d** proteins, as assessed by immunoblot normalized to β-tubulin or GAPDH and *Ctrl* sgRNA cells, or **e**, **f** mRNA, as assessed by RT-qPCR and normalized to *18S* rRNA and *Ctrl* sgRNA cells, from *n* = 4 (protein) or *n* = 3 (mRNA) biological replicate experiments (black circles) of 293T cells transduced with vectors encoding either a *Ctrl* or *PABPN1* sgRNA, either no transgene or *PABPN1* cDNA, and either the **a**, **c**, **e** GFP^rare^/mCherry^com^ or **b**, **d**, **f** mCherry^rare^/GFP^com^ dual reporter. **g** Mean ± SEM of mRNA co-immunoprecipitated with an anti-FLAG antibody from 293T cells co-transfected with both GFP^rare^/mCherry^com^ and mCherry^rare^/GFP^com^ dual reporters and a vector encoding FLAG-PABPN1, as assessed by RT-qPCR and normalized to input mRNA levels of each reporter in the cell and then to the level of *GFP*^*rare*^ mRNA. Three-way ANOVA performed with Tukey’s post hoc test was performed for **c** (F ratio = 71.9048, *p* < 0.0001, *n* = 4 biological replicates), **d** (F ratio = 4.5083, *p* = 0.0025, *n* = 4 biological replicates), **e** (F ratio = 8.4125, *p* = 0.0002, *n* = 3 biological replicates), and **f** (F ratio = 5.8357, *p* = 0.0017, *n* = 3 biological replicates). One-way ANOVA was performed with Tukey’s post hoc test for **g** (F ratio = 12.3719, *p* = 0.0023, *n* = 3 biological replicates). *p < 0.05, **p < 0.01, and ****p < 0.0001. Different exposure times were required for each antibody (see uncropped images in Supplementary Data 5). Source data are provided as a Source Data file.

To independently evaluate the PAXT connection as a modifier of rare codon-dependent expression, we repeated the above analysis but instead inactivated PABPN1, another subunit of the PAXT connection^27,28^, with the caveat that PABPN1 is also involved in polyadenylation regulation^25^. 293T cells were stably infected in quadruplicate with a lentivirus encoding one of two independent *Ctrl* or *PABPN1* sgRNAs, followed by co-transfection with either the GFP^rare^/mCherry^com^ or mCherry^rare^/GFP^com^ dual reporter and a vector encoding either no transgene or FLAG epitope-tagged PABPN1 designed to be resistant to the corresponding sgRNAs. Immunoblot analysis of the resultant 24 cell cultures confirmed the appropriate reduction of endogenous PABPN1 protein and the corresponding restoration by the ectopic version (Fig. 6a, b, and Supplementary Fig. 13a, b). Immunoblot analysis of the four reporter proteins, followed by quantification, revealed a significant increase or a trend towards an increase in GFP^rare^ and mCherry^rare^ protein levels compared to mCherry^com^ and GFP^com^ levels, respectively, upon reducing PABPN1, all of which were reversed by re-expressing PABPN1 (Fig. 6c, d). RT-qPCR analysis of three biological replicates of the same cells revealed a statistically significant increase in all four reporter mRNAs in *PABPN1* sgRNA versus ectopic PABPN1 cells (Fig. 6e, f). This effect did not appear to be indirect via a reduction in ZFC3H1 expression. Specifically, 293T cells were independently infected in quadruplicate with a lentivirus encoding *Ctrl, PABPN1*, *ZFC3H1*, or *MTREX*-targeting sgRNA, and the resultant 16 cell lines were subjected to immunoblot analysis with antibodies against each of the three proteins. This revealed the appropriate reduction of the targeted genes. Apart from a decrease in ZFC3H1 levels upon MTREX depletion, as previously reported^28,31^, loss of one component did not alter the expression of the remaining two (Supplementary Fig. 14). Given that PABPN1 is an RNA-binding protein^25^, we also tested whether PABPN1 exhibits a preference for rare versus common codon-enriched reporter transcripts. 293T cells were co-transfected in triplicate with a vector encoding FLAG-PABPN1 and both the GFP^rare^/mCherry^com^ and mCherry^rare^/GFP^com^ dual reporters together. Protein lysates were collected from the resultant three cell cultures, a portion of which was retained as an input control, while the remainder was immunoprecipitated with either an anti-FLAG or IgG antibody. RNA was extracted from the immunoprecipitates and subjected to RT-qPCR with primers specific to the four reporters, after which reporter transcript levels were normalized to input levels. As controls, we verified that the four reporter transcripts were essentially absent in the IgG immunoprecipitates (Supplementary Fig. 15a) and that FLAG-PABPN1 was immunoprecipitated with the anti-FLAG, with very little immunoprecipitated with the IgG antibody (Supplementary Fig. 15b). We found a significant increase in the amount of both rare codon-enriched reporter transcripts compared to their common counterparts co-immunoprecipitating with FLAG-PABPN1 (Fig. 6g). Collectively, these data support that PABPN1 loss phenocopies ZFC3H1 loss, an effect associated with preferential co-immunoprecipitation with the rare codon-enriched reporters.

### ZFC3H1 loss increases protein levels of endogenous rare codon-enriched genes

To address whether the effects of ZFC3H1 loss on the aforementioned reporters reflect codon-specific effects of endogenous genes, we analyzed the transcriptome and proteome of cells in the absence and presence of ZFC3H1. In more detail, 293T cells were independently infected in triplicate with two independent *Ctrl* or *ZFC3H1* sgRNAs and the resultant stable 12 cell lines expanded into three plates each for simultaneous immunoblot (Supplementary Fig. 16), transcriptomic, and proteomic analysis (Supplementary Fig. 17a). For immunoblot analysis, we confirmed appropriate ZFC3H1 protein expression (Supplementary Fig. 16). For transcriptomics, isolated RNA was poly(A)-selected, reverse transcribed, PCR amplified, indexed, and the resultant library subjected to paired-end next generation sequencing. This recovered 48–150 million reads with similar percentage of uniquely mapped reads across all samples (81.64–88.06%), validating the quality of the libraries (Supplementary Data 4)^55^. For proteomics, protein lysates were subjected to quantitative liquid chromatography tandem mass spectrometry, which identified ~9400 unique proteins, ~8500 of which were detected in all 12 samples (Supplementary Data 4). There was also high precision, with a low coefficient of variation (median 5.6–7.3%) within each of the triplicate samples, validating the quality of the samples.

We confirmed by principal component analysis that the transcriptomes of the six *Ctrl* sgRNAs cell lines clustered together, as did the six *ZFC3H1* sgRNA cell lines, and that the two clusters were different from each other (Supplementary Fig. 17b). Further, we demonstrated that the levels of three validated PAXT targets (*SNHG10*, *19*, and *20* lncRNAs) increased upon the loss of ZFC3H1 (Supplementary Fig. 17c)^27^. Consistent with the role of the PAXT connection in RNA degradation^27,28,32^, ZFC3H1 depletion was associated with a general increase in the level of polyadenylated transcripts (Supplementary Fig. 17d). Principal component analysis of the proteomics similarly confirmed that the *Ctrl* versus *ZFC3H1* sgRNA cell lines clustered within the same guide but were different from each other (Supplementary Fig. 17e). Lastly, we confirmed that the number of ZFC3H1 peptides significantly decreased in *ZFC3H1* sgRNA cells, which we also validated by immunoblot analysis (Supplementary Fig. 17f). We thus demonstrate by multiple assays that *ZFC3H1* mRNA and protein were successfully depleted, leading to established changes to the transcriptome.

**Fig. 7:**
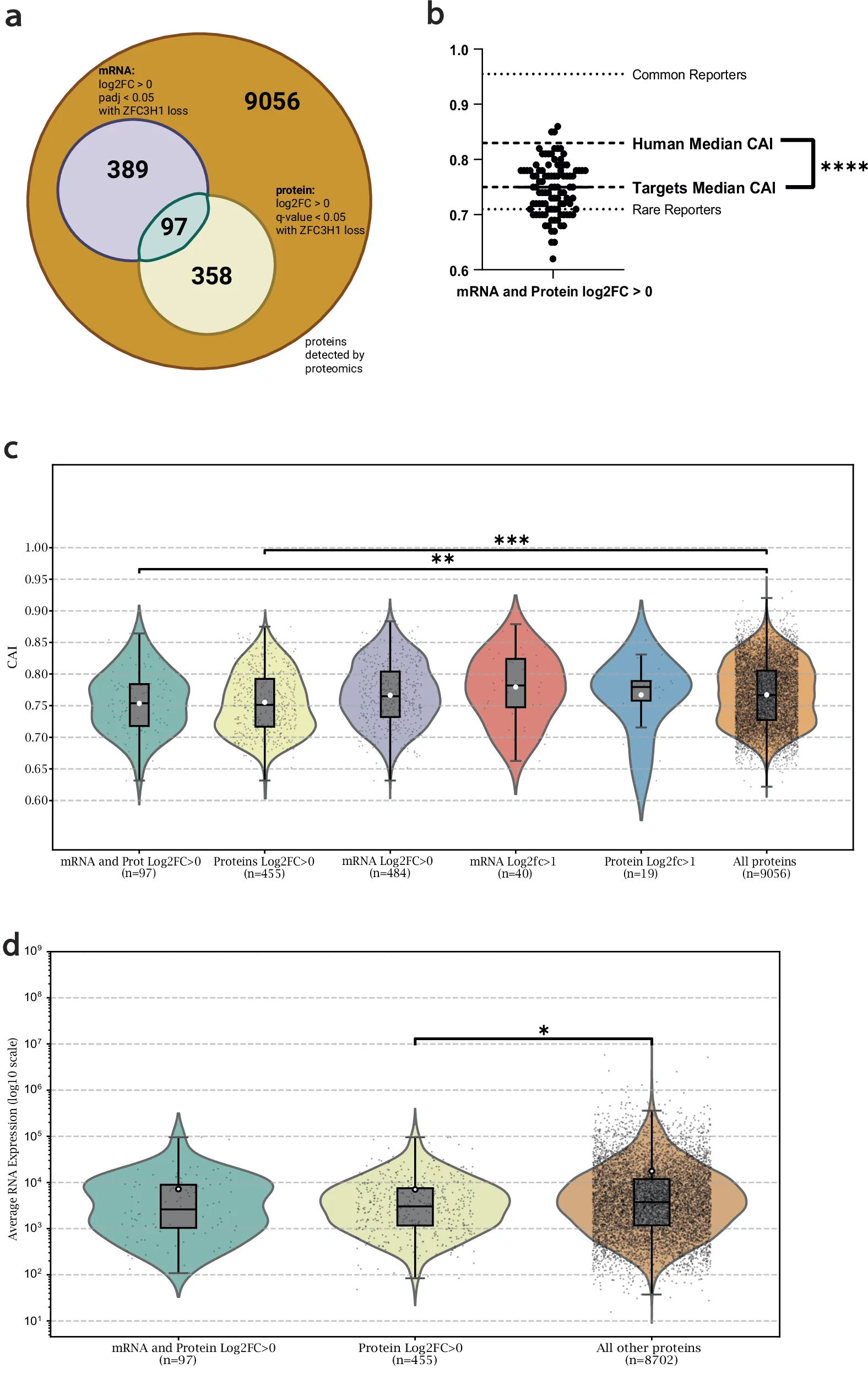
Codon usage and expression of endogenous genes that exhibit increased expression upon ZFC3H1 loss. **a** Schematic of RNA-seq and proteomics analysis groupings showing 9056 genes detected by proteomics and all genes significantly increased at either the mRNA (486), protein (455), or both (97) levels upon ZFC3H1 loss (created in BioRender. Niehoff, T. (2026) https://BioRender.com/s4ytj9w). **b** CAI for all genes with significant log_2_FC > 0 mRNA and protein levels with ZFC3H1 loss. Dotted lines: human median CAI, along with average CAI for the rare and common (both *GFP* and *mCherry* for each) reporters. **c** Violin plot distributions of CAI for the indicated groups. **d** Violin plot distributions of log_10_ average RNA expression in *Ctrl* sgRNA cells by read count for the indicated groups. Two-sided Wilcoxon signed-rank test was performed for **b**. Two-sided *p*-values, comparing the mean of each distribution to the distribution of all genes detected by proteomics (orange), were generated using a size-matched random subsampling approach for **c**, **d**. For violin plots, white dots indicate the population mean. Boxes in boxplots represent Q1, median, and Q3. Whiskers represent 1.5× IQR, excluding outliers. *n* = 6 biological replicates for *Ctrl* and *ZFC3H1* sgRNA cells for RNA-seq and proteomics. *p < 0.05, **p < 0.01, ***p < 0.001, and ****p < 0.0001. Source data are provided as a Source Data file.

For analysis of the transcriptome, counts for each transcript were first normalized to the total library size, and the fold-change of the average counts between the six replicate *ZFC3H1* and *Ctrl* sgRNA cell lines was calculated utilizing DESeq2^56^. We then conducted a census for transcripts significantly increased (log_2_ fold-change > 0, *p* adjusted < 0.05) upon ZFC3H1 depletion that were also detected by proteomics, which identified 486 mRNAs. For analysis of the proteome, we determined the fold-change in normalized protein counts (derived from peptide counts) between the six *ZFC3H1* versus *Ctrl* sgRNA cell lines utilizing a two-sample test in Perseus^57^, which identified 455 proteins (log_2_ fold-change > 0, *q* < 0.05). We then filtered for genes in common between the two datasets (Fig. 7a). Namely, to identify genes affected by ZFC3H1 loss, we applied a high stringency of selecting only genes whose expression increased at both the mRNA and protein level in *ZFC3H1* versus *Ctrl* sgRNA cell lines, which identified 97 genes. We next determined the CAI for each of these 97 transcripts, namely how close the codon usage of these transcripts was to highly expressed genes, with 0 indicating completely different codon usage and 1 indicating identical codon usage^58,59^. This revealed that these genes had CAI  values between 0.62 and 0.86, with a median of 0.75 (Fig. 7b). The median CAI of these genes is significantly lower (e.g., more rare, *p* < 0.0001) than the median CAI (0.83)^8^ of the human transcriptome. In fact, the CAI of *GFP*^*rare*^ and *mCherry*^*rare*^ (0.71 for both) is much more similar to these candidate ZFC3H1 target transcripts than the CAI of *GFP*^*com*^ (0.96) and *mCherry*^*com*^ (0.95) (Fig. 7b). Thus, unbiased transcriptome and proteome analysis to identify targets of ZFC3H1, namely genes in which both the mRNA and protein expression was increased upon depleting ZFC3H1, revealed that these candidate ZFC3H1 targets have a significant bias towards rare codons. To independently confirm these effects, we demonstrated by RT-qPCR that the levels of three representative transcripts (*PIGW*, *TIGD1*, and *TSPYL4*) from these 97 genes were increased in three newly created and validated *ZFC3H1* versus *Ctrl* sgRNA 293T cell lines (Supplementary Fig. 18a, b).

To explore this phenomenon further, we determined the CAI of all genes significantly increased at the mRNA (486), protein (455), or both (97) levels upon ZFC3H1 loss in comparison to all genes whose proteins were detected by proteomics (9056). This revealed a significant decrease in the average CAI (i.e., rare codon-enriched genes) of genes that exhibited increased protein levels or increased protein and mRNA levels upon ZFC3H1 loss. On the other hand, the average CAI of genes that increased at the mRNA level was not significantly different from the CAI of all genes in the proteome dataset (Fig. 7c). This mirrors the observations of the reporters, namely, that the increase in protein levels of rare codon-enriched reporters was larger and more consistent than observed at the mRNA level. Increasing the stringency to a log_2_ fold-change > 1 reduced the number of candidates that increased at the mRNA (40) or protein (19) level upon the loss of ZFC3H1, reducing statistical power and the ability to detect significant changes in CAI (Fig. 7c). We next compared the CAI in the first (Supplementary Fig. 19a) or last (Supplementary Fig. 19b) 50 codons, which revealed significantly lower CAI at both ends in genes upregulated at the protein level, and a significantly lower CAI at the 3′ end for genes upregulated at both the mRNA and protein level. These observations suggest that transcripts that are increased upon the loss of ZFC3H1 do not exhibit an overt clustering of rare codons at one end of the transcript or the other. Collectively, therefore, these data demonstrate that the loss of ZFC3H1 preferentially led to an increase in the protein levels of genes enriched in rare codons.

### Feature analysis of genes upregulated upon the loss of ZFC3H1

To explore whether there may be other features associated with genes upregulated upon the loss of ZFC3H1, we determined the level of mRNA (read counts) of genes that increased at the protein (455) or both protein and mRNA (97) level, compared to all remaining genes detected by proteomics (excluding the aforementioned 455 genes) in *Ctrl* cells. This revealed that the average mRNA level of genes upregulated at the protein or protein and mRNA level was significantly lower or trended lower, respectively, than that of all other genes in the proteomic dataset. These data indicate that genes upregulated by the loss of ZFC3H1 tend to be poorly expressed at the mRNA level (Fig. 7d). We similarly analyzed the average protein expression (peptide counts) of genes that increased at the mRNA (486) or both protein and mRNA (97) level, compared to all remaining genes detected by proteomics (excluding the aforementioned 486 genes) in *Ctrl* cells. This revealed that the average protein level of genes upregulated at the mRNA or both the mRNA and protein level was significantly or trended, respectively, towards being lower than that of all other genes in the proteomic dataset (Supplementary Fig. 20), further indicating that mRNA or proteins significantly increased upon the loss of ZFC3H1 are poorly expressed. Because nuclear export^12^ and ZFC3H1 activity^33^ can be influenced by GC content at the 5′ end of the transcript, we also examined GC content of these same four gene sets. This revealed that the average GC fraction of genes upregulated at the mRNA level upon ZFC3H1 loss was significantly higher than all genes detected by proteomics; namely, these genes were GC-rich (Supplementary Fig. 21a). Perhaps related, it has been shown that GC-rich motifs increase ZFC3H1 targeting to transcripts^33^. On the other hand, the average GC fraction of genes upregulated at the protein level upon ZFC3H1 loss was significantly lower, or AU-rich (Supplementary Fig. 21a). Further analysis of GC content in the first (Supplementary Fig. 21b) or last (Supplementary Fig. 21c) 50 codons revealed a significant decrease in the average GC fraction (i.e., AU-rich) at the 3′ end of genes upregulated at the protein level upon ZFC3H1 loss. Additionally, we repeated this analysis, but on the introns of these four gene sets, which also revealed a significant decrease in the average GC fraction in the introns of genes upregulated at the protein level upon ZFC3H1 loss (Supplementary Fig. 22a), and this was more evident at the 3′ end, but not 5′, end of these genes (Supplementary Fig. 22b, c). Since RNA-binding protein motifs have been previously identified as a potential predictor for PAXT targeting^60^, we examined the presence of such motifs in these four gene sets. Not surprisingly, genes upregulated at the mRNA level upon the loss of ZFC3H1, which are GC-rich, were enriched in GC-rich motifs (Supplementary Table 3), while those upregulated at the protein level, which are AU-rich, were enriched in AU-rich motifs (Supplementary Table 4). Finally, PAXT has been suggested to target long^27^ or short^33^ transcripts. Thus, we compared the average length of both the coding DNA sequence (CDS) and introns of these four gene sets, which revealed that genes upregulated at the protein level upon ZFC3H1 loss were significantly longer on average (~400 nucleotides) than all genes detected by proteomics (Supplementary Fig. 23), but there was no difference in the total length of their introns, only genes upregulated at the mRNA level exhibited shorter introns (Supplementary Fig. 24). Thus, feature analysis suggests that beyond being enriched in rare codons, genes upregulated at the protein level upon the loss of ZFC3H1 are also more poorly expressed at the mRNA level, AU-rich, especially at the 3′ end, and have a longer coding sequence.

## Discussion

We performed a CRISPR/Cas9 loss-of-function screen for modifiers of GFP^rare^ compared to mCherry^com^ fluorescence, which identified the PAXT connection as a suppressor of rare codon-dependent expression. Further analysis revealed that the loss of ZFC3H1 led to a trend or significant increase in the level of *GFP*^*rare*^ and *mCherry*^*rare*^ mRNA in the nucleus, and interestingly, PABPN1 preferentially immunoprecipitated with these two transcripts compared to their common counterparts. One interpretation of these findings consistent with the known function of the PAXT connection is that PAXT preferentially targets rare codon-enriched reporter transcripts in the nucleus for degradation. In support, the stability of *GFP*^*rare*^ and *mCherry*^*rare*^ transcripts was also slightly increased upon the loss of ZFC3H1, but other interpretations are possible. We also observed that the levels of *GFP*^*rare*^ and *mCherry*^*rare*^ mRNA were significantly and preferentially increased compared to *GFP*^*com*^ and *mCherry*^*com*^ mRNA in the cytoplasm upon the loss of ZFC3H1—potentially due to nuclear leakage of the increased nuclear transcript pools, although again, other mechanisms could similarly explain this increase. Reassuringly, a recent study^51^ also identified the PAXT connection as a modifier of rare codon-enriched transcripts and identified increased nuclear stability and cytoplasmic levels of *GFP*^*rare*^. Taken together, we hypothesize that the loss of ZFC3H1 increases all reporter mRNA expression. However, the admittedly small and variable preferential increase in the rare codon-enriched reporter transcripts compared to their common versions ultimately manifests in their higher protein expression. We note that the difference in protein levels between rare and common codon reporters in the absence of ZFC3H1 was consistently larger than the increase in their mRNA levels. Additionally, increasing the amount of rare codon-enriched reporters transfected into cells reduced the ability of ZFC3H1 loss to impart this increase. These findings suggest that low mRNA levels enhance the ability of ZFC3H1 loss to affect reporter expression in a rare codon-dependent manner. Indeed, whole-cell transcriptomics and proteomics revealed a general increase in mRNAs independent of codon usage upon the loss of ZFC3H1, but the proteins that significantly increased were encoded by transcripts that were both rare codon-enriched (and hence also AU-rich)^11^ and poorly expressed (and, perhaps relatedly, long)^61^. We thus suggest that loss of a functional PAXT connection leads to a preferential increase in the protein levels of rare codon-enriched transcripts that are poorly expressed.

Caveats to this study include that the experiments were performed in one cell line, and hence, ZFC3H1 loss could affect different stages of gene expression to different degrees. Given this, we suggest our findings provide one explanation for the effect of ZFC3H1 loss on rare codon-enriched genes. Another caveat is that because small changes in mRNA levels of the rare codon-enriched reporters were amplified at the protein level, it may be an accumulation of very small effects at different stages of mRNA production that together result in the increase in protein expression. As such, differences in any one stage (transcription, export, stability, etc.) may be difficult to detect. This may be especially true for RNA stability, where PAXT may elicit stronger effects earlier in the nucleus on rare and/or common codon-enriched transcripts than detected by the Actinomycin D experiments. Pulse-chase labeling to examine early degradation of reporter transcripts may resolve this issue. Given this, we recognize that many stages of rare codon-enriched reporters may yet be affected by the loss of ZFC3H1.

Changing rare codons to common in the cyanobacterial *KaiBC* gene disrupted the dampening of circadian rhythms at low temperature^62^. Thus, certain pathways may be enriched in genes with a bias towards rare codons to provide dynamic expression changes during specific conditions^63^. This may even extend to multiple genes within the same pathway. Indeed, in yeast, the pheromone-sensing pathway is enriched in genes with a bias towards rare codons, and this is a pathway involved in transient responses to stimuli^64^. Given this, we explored whether the genes enriched in rare codons that were more highly expressed at the protein level upon the loss of ZFC3H1 were associated with pathways. GO analysis identified pathways involved in RNA degradation, perhaps pointing towards a potential negative feedback loop, and general metabolic processes (Supplementary Fig. 25a). Kyoto Encyclopedia of Genes & Genomes (KEGG) enrichment pathway analysis identified RNA degradation and cell cycle as being significantly upregulated (Supplementary Fig. 25b). Human Phenotype enrichment analysis identified phenotypes involving developmental abnormalities (Supplementary Fig. 25c), and perhaps related, loss of ZFC3H1 impairs mouse embryonic stem cell differentiation^65^. Perhaps, then, modifiers like ZFC3H1 could reprogram the proteome by fostering small increases in the mRNA levels of poorly expressed genes enriched in rare codons.

## Methods

### Cells

293T cells^66,67^ were obtained from the Duke University Cell Culture Facility and cultured at 37 °C and 5% CO_2_ in DMEM supplemented with 10% fetal bovine serum (and 1% penicillin/streptomycin for the initial screen). Cells were regularly tested for mycoplasma using the MycoAlert Mycoplasma Detection Kit (Lonza Bioscience).

### Plasmids

pcDNA3.1+GFP^rare^/mCherry^com^ or pcDNA3.1+mCherry^rare^/GFP^com^ were previously described^34^. pLX303BRAF^V600E^ and pLX303Luciferase were cloned from pCW107^34,68^ to pLX303 (a gift from David Root, Addgene vector # 25897)^69^ using Gateway recombination methods (Thermo Fisher Scientific). pCW107 vectors were mixed with the BP-Clonase reaction and the pDONR223 entry vector (a gift from David Root; Addgene vector # 25894)^69^, and the sequence of the resulting pDONR223 vectors was verified. Next, a LR-Clonase reaction with the pDONR223 entry vector and pLX303 destination vector was performed, and vectors were validated through sequencing. pcDNA3.1+FLAG-ZFC3H1 was created by amplifying the template vector encoding the ZFC3H1 sequence acquired from the DNASU Plasmid Repository (Plasmid ID HsCD00297039) by PCR using Q5 High-Fidelity DNA Polymerase (NEB), subcloning into the pcDNA3.1+ vector (Thermo Fisher Scientific), and validated by sequencing. pcDNA3.1+FLAG-PABPN1 was created by gene synthesis using GeneArt (Thermo Fisher Scientific) and subcloned into the pcDNA3.1+ vector. For individual *ZFC3H1* and *PABPN1* sgRNAs, two sgRNAs most enriched/depleted in MAGeCK sgRNA summary output files from the ERK1/2 substrate subset CRISPR screen were selected, along with two non-targeting sgRNAs that were not enriched/depleted (see Supplementary Data 7 for sgRNA sequences). *MTREX* sgRNA sequence was generated using CHOPCHOP^70^. Oligonucleotides encoding these sgRNAs were synthesized by Integrated DNA Technologies (IDT) and separately cloned into pLentiCRISPRv2 (a gift from Feng Zhang; Addgene vector # 52961)^71^ vector. Any plasmids generated in this study are available from the corresponding author upon request.

### CRISPR/Cas9 sgRNA screen

#### Library preparation

The ERK1/2 substrate CRISPR/Cas9 sgRNA library was previously described^35^. A 150 mm plate of 80% confluent 293T cells was transfected with 12 μg of this library, 9 μg of psPAX2, and 3 μg of pVSVg using the FuGENE 6 reagent following the manufacturer’s protocol (Fugene). Media was changed the next day to DMEM containing 30% FBS, collected 2 days later, and filtered through a 0.45 μm filter, similar to that previously described^72,73^. Viral titer was determined by infection with varying dilutions of the virus-containing media as previously described^73^.

#### Infection

6 × 10^6^ 293T cells were seeded in triplicate and infected the next day with lentivirus derived from the ERK1/2 substrate LentiCRISPR library at an ~0.3 MOI, as previously described^74^. Twenty-four hours later, the cells were cultured in 1 µg/ml puromycin for 4–6 days. One portion of these stably infected cells were frozen for subsequent genomic DNA isolation as the presort sample, while 8 × 10^6^ of the second portion were seeded in duplicate cultures and transfected the next day with 6 μg of pcDNA3.1+ GFP^rare^/mCherry^com^ and 6 μg of pLX303BRAF^V600E^ or pLX303Luciferase using FuGENE 6 reagent following the manufacturer’s protocol (Fugene).

#### Flow sorting

Forty-eight hours after transfection, cells were trypsinized and flow-sorted by a BC Astrios Sorter. Flow gates were set with parallel luciferase transfected controls, which revealed that approximately 10–12% of BRAF^V600E^-transfected cells occupied a GFP+ gate absent in Luciferase-transfected cells. Six to 8 × 10^5^ cells were harvested from the GFP^hi^ gate while 2 × 10^6^ cells were harvested from GFP^lo^ gates. Sorted cells were pelleted, washed twice with PBS, and frozen for subsequent genomic DNA isolation and screen processing.

#### Sequencing

Genomic DNA was prepared from the presorted cells as well as the GFP^lo^- and GFP^hi^-sorted cells using the DNeasy kit according to the manufacturer’s protocol (Qiagen). DNA encoding sgRNA sequences from 10 μg of genomic DNA were amplified in 4–100 μl GECKO PCR1 reactions (see Supplementary Data 7 for primer sequences) utilizing Taq polymerase HS (Takara) and the cycles of 95 °C for 5 min, 20 cycles of 95 °C for 30 s, 60 °C for 30 s, and 72 °C for 30 s, and a final extension step of 72 °C for 5 min. PCR1 reactions were then pooled and partitioned into four lentiCRISPR PCR2 reactions per sample (see Supplementary Data 7 for primer sequences). PCR2 reactions utilizing Taq polymerase HS (Takara) and cycles of 95 °C for 5 min, 20 cycles of 95 °C for 15 s, 58 °C for 30 s, and 72 °C for 30 s, and a final extension step of 72 °C for 7 min. PCR2 reactions were assessed by agarose electrophoresis to validate amplification at the proper 355 bp-amplified fragment. Ten microliters from each PCR2 reaction were pooled into a single sample and purified with AxyPrep Magnetic Bead according to the manufacturer’s protocol (Axygen) and sequenced by Next Generation Sequencing on a MiSeq instrument (Hudson Alpha Labs). Reads were mapped to sgRNA references and analyzed by MAGeCK (Version 0.5.8)^75^. The output file with gene summaries was used for downstream analysis. BRAF and 203 genes that had FDR < 0.26 from the GFP^hi^ versus presort, GFP^hi^ versus GFP^lo^, or GFP^lo^ versus presort cells at either time point or in the two time points collapsed together and were selected for validation.

#### Validation

An sgRNA LentiCRISPR library was generated with 1021 sgRNAs targeting the above 204 genes, 50 control sgRNAs from the primary screen, as well as 100 additional non-targeting control sgRNAs from the human GeCKOv2 library (HGLibA_64384 to HGLibA_64483), as previously described^74^. 293T cells were infected, selected, transfected, and sorted exactly as above with the following four exceptions: (1) A second population of stably infected cells was transfected with pcDNA3.1+mCherry^rare^/GFP^com^ and pLX303BRAF^V600E^. (2) 2 to 4 × 10^5^ cells were sorted from the GFP^hi^ gate (pcDNA3.1+ GFP^rare^/mCherry^com^) or mCherry^hi^ gate (pcDNA3.1+mCherry^rare^/GFP^com^), while 4 × 10^5^ to 1 × 10^6^ cells were sorted from GFP^lo^ gate (pcDNA3.1+GFP^rare^/mCherry^com^) or mCherry^lo^ gate (pcDNA3.1+mCherry^rare^/GFP^com^). (3) Genomic DNA was isolated by phenol/chloroform extraction followed by ethanol precipitation using standard procedures. (4) Sequencing libraries (see Supplementary Data 7 for primer sequences) were sequenced on an Illumina NextSeq 500 instrument (Sequencing and Genomic Technologies Shared Resource at Duke Cancer Institute). Gene groupings of the ten identified modifiers were determined by STRING (Version 12.0)^41^, and the node network was visualized in Cytoscape (Version 3.10.3)^76^.

### Generating ZFC3H1, PABPN1, and MTREX sgRNA and rescued cells

3 × 10^5^ 293T cells were plated in wells of a 6-well plate. The next day, cells were transfected with 1 µg of LCV2 plasmid containing either a *Ctrl*, *ZFC3H1*, *MTREX*, or *PABPN1*-targeting sgRNA sequence, 0.5 µg psPAX2, and 0.5 µg of pVSVg, using the FuGENE 6 reagent following the manufacturer’s protocol (Fugene). Four to 6 h after transfection the media was replaced with 2 ml of DMEM supplemented with 10% FBS. Forty-eight hours after transfection, viral media was harvested and filtered through a 0.45 µm syringe filter. 1 × 10^6^ 293T cells were added to wells in a 6-well plate in 1 ml of DMEM (with 10% FBS). One milliliter of viral media with 8 µg/ml polybrene was then added, followed by spinfection at 265 × *g* for 60 min. Cells were incubated at 37 °C overnight and replated in 10 cm dishes. The next day, the cells were selected in 2 µg/ml of puromycin for 48 h and allowed to recover for 1–3 days in fresh media. Cells were then plated in a 6-well plate at 3.5 × 10^5^ cells/well, and the next day transfected with 0.4 µg of either pcDNA3.1+GFP^rare^/mCherry^com^ or pcDNA3.1+mCherry^rare^/GFP^com^ (unless otherwise noted) and 0.4 µg of either pcDNA3.1+ or pcDNA3.1+FLAG-ZFC3H1/PABPN1 (if a cDNA rescue was not included in the experiment, no pcDNA3.1 was transfected) using the FuGENE 6 reagent following the manufacturer’s protocol (Fugene). Forty-eight hours later, cells were rinsed with ice-cold PBS, collected, pelleted at 400 × *g* for 5 min at 4 °C, and stored at −80 °C for subsequent immunoblot or RT-PCR analysis.

### Generating *ZFC3H1* siRNA cells

2 × 10^5^ 293T cells were plated in wells of a 12-well tissue plate and allowed to attach overnight. The next day, media was replaced with 900 µl of serum-free Opti-MEM (Gibco) media 30 min before transfection. Twenty-five pmol of siRNA (MedChemExpress, catalog: HY-RS16034) was diluted in 50 µl of Opti-MEM. Three microliters of RNAiMAX (Invitrogen) was diluted in 50 µl of Opti-MEM. siRNA and RNAiMAX solutions were added together, gently mixed, and incubated at room temperature for 5 min. The siRNA mixture was then added dropwise to cells, and cells were incubated at 37 °C for 4 h. The media was then replaced with DMEM + 10% FBS. The next day, 6 × 10^5^ siRNA-transfected 293T cells were plated in the wells of a 6-well plate, and 4 h later, transfected with either pcDNA3.1+GFP^rare^/mCherry^com^ or pcDNA3.1+mCherry^rare^/GFP^com^ or both using the FuGENE 6 reagent following the manufacturer’s protocol (Fugene). Media was changed the next day. One day later, the cells were harvested. Half the wells were incubated in RLT plus lysis buffer (Qiagen); RNA was isolated using the RNeasy Plus Kit according to the manufacturer’s protocol (Qiagen) and subjected to reverse transcription followed by quantitative PCR (RT-qPCR), as described below. The other half of the wells were incubated with RIPA buffer (50 mM Tris-HCl pH 8.0, 150 mM NaCl, 1% NP40, 0.5% sodium deoxycholate, 0.1% sodium dodecyl sulfate, 1× Proteinase Inhibitor EDTA-free (Roche)) for immunoblot analysis.

### Flow cytometry analysis

*ZFC3H1* sgRNA and rescue cells expressing pcDNA3.1+GFP^rare^/mCherry^com^ were generated as described above, except 293T cells were transfected with 1 µg of GFP^rare^/mCherry^com^ reporter. Cells were trypsinized and resuspended in PBS + 5% FBS. Cells were analyzed on a LSRFortessa X-20 (BD) using BD FACSDiva 9.0 software. Live singlet cells were first isolated, and then the double positive (GFP and mCherry-expressing population) cells were isolated, and their average GFP and mCherry fluorescence signal was calculated for each condition. See Supplementary Data 3 for gating conditions.

### Measuring RNA stability after Actinomycin D

2.5 × 10^6^ *ZFC3H1* or *Ctrl* sgRNA 293T cells were plated on a 10 cm culture plate and allowed to attach overnight. The next day, cells were transfected with 10.71 µg of pcDNA3.1+GFP^rare^/mCherry^com^ and pcDNA3.1+mCherry^rare^/GFP^com^ per plate utilizing the Fugene 6 reagent following the manufacturer’s protocol (Fugene). The next day, 5 × 10^5^ cells were plated from each transfection in a 6-well plate for each time point. The next day, two wells of each replicate were harvested: one for immunoblot analysis (in RIPA buffer) and one for *t* = 0 timepoint RNA (in Trizol). At the same time, wells were treated with 5 µg/ml Actinomycin D (Gibco). At 2, 4, and 8 h post Actinomycin D treatment, cells were rinsed once in PBS and then lysed in Trizol according to the manufacturer’s protocol (Invitrogen). RNA was then extracted and subjected to RT-qPCR as described below. For Supplementary Fig. 8, Cq technical replicate values were excluded that were not within 2 Cq values of one of the other two technical replicate values for a batch. The three technical replicate values for one batch were removed if there was high inconsistency among the three (no 2 values within 2 cq of each other). Two batches of three technical replicates were performed and averaged together for each biological replicate at each time point. See Supplementary Data 6 for excluded values.

### Nuclear-extranuclear fractionation

3 × 10^6^ *ZFC3H1* or *Ctrl* sgRNA 293T cells were plated on a 10 cm cell-culture plate and allowed to attach overnight. The next day, cells were transfected with 3.43 µg pcDNA3.1+GFP^rare^/mCherry^com^ and pcDNA3.1+mCherry^rare^/GFP^com^ per plate utilizing the FuGENE 6 reagent following the manufacturer’s protocol (Fugene). Media was replaced 4–6 h after transfection. Forty-eight hours thereafter, cells were fractionated as previously described with minor modifications^52^. Briefly, cells were trypsinized, isolated by centrifugation at 400 × *g*, and washed three times in PBS. Ten percent of cells were lysed in Trizol, while the remaining cells were resuspended in 500 µL φ buffer (150 mM potassium acetate, 5 mM magnesium acetate, 20 mM HEPES pH 7.4, 1 mM sodium fluoride, 1 mM sodium orthovanadate, 1× protease inhibitor cocktail EDTA-free (Roche), 1:1000 dilution of SUPERase In™ RNase (Invitrogen), and 0.1% diethylpyrocarbonate). Five hundred microliters φ plus detergent (1% Triton X-100 and 0.2% sodium deoxycholate) was added, and cells were incubated for 3 min on ice. Cells were spun at 800 × *g* for 5 min at 4 °C; the supernatant/extranuclear fraction was removed; the cell pellet underwent two washes (once with 500 µL φ and 500 µL φ plus detergent buffer, and once with 1 mL φ buffer), and then the pellet was resuspended in 1 mL φ buffer, and Trizol was added. The extranuclear fraction was spun down at 16,100 × *g* for 10 min at 4 °C, and the supernatant was lysed in Trizol as the extranuclear fraction. Before addition of Trizol, 50 µL of each fraction was reserved for immunoblot analysis. All fractions then underwent Trizol-chloroform extraction as described below. RNA was dissolved in 20 µl of water and DNase-treated by adding 2.5 µl of DNase I (Thermo Fisher) and 2.5 µl of DNase buffer, and incubated at 37 °C for 1 h. RNA was enriched using the RNeasy Plus Kit following the manufacturer’s protocol (Qiagen). Five hundred nanograms of RNA was resolved on a 1% bleach agarose gel as previously described^53^. Five hundred nanograms of RNA per sample was reverse transcribed and analyzed using qPCR using the Gotaq 2-step RT-qPCR system following the manufacturer’s protocol (Promega). qPCR reactions were performed in the CFX 384 real-time system C1000 touch thermal cycler (Bio-Rad). See below for qPCR cycling conditions.

### Puromycin SUnSET assay

The protocol was performed as previously described^47,77,78^. Briefly, 8 × 10^6^ *Ctrl* or *ZFC3H1* sgRNA or siRNA cells were plated in a 6-well plate and allowed to attach overnight, such that cells were 60–70% confluent the next day. The next day, cells were treated with 10 µg/mL puromycin for 10 min, rinsed in ice-cold PBS, and lysed in RIPA buffer. Cycloheximide-treated cells were pretreated with 10 µg/mL cycloheximide for 10 min, media was removed, and puromycin was added to fresh media after as described above. Immunoblot analysis was performed as described below.

### RNA immunoprecipitation

RNA immunoprecipitation was performed as previously described^79^ with minor modifications. 8 × 10^6^ 293T cells were plated in a 10 cm tissue culture dish. The next day, cells were co-transfected with 1.5 µg of both pcDNA3.1+GFP^rare^/mCherry^com^ and pcDNA3.1+mCherry^rare^/GFP^com^ and 3 µg of pcDNA3.1+FLAG-PABPN1, using the FuGENE 6 reagent following the manufacturer’s protocol (Fugene). Two days after transfection, cells were fixed in 1% formaldehyde in PBS for 10 min at room temperature. Glycine was then added to a final concentration of 0.125 M and incubated for 5 min at room temperature. Cells were rinsed twice in ice cold PBS and then scraped into ice cold PBS. Cells were spun down at 1000 × *g* for 5 min at 4 °C. Cells were then resuspended in a mixture of 2 ml nuclear isolation buffer (1.28 M sucrose, 40 mM Tris-HCl pH 7.5, 20 mM MgCl_2_, 4% Triton-X 100, 200 U/ml RNaseOUT (Invitrogen)), 2 ml PBS, and 6 ml H_2_O, and sheared with two strokes of an 18 g needle. Cells were then incubated on ice for 20 min with frequent mixing. Nuclei were pelleted at 2500 × *g* for 15 min at 4 °C. Nuclei were resuspended in 1 ml RIP buffer (150 mM KCl, 25 mM Tris pH 7.4, 5 mM EDTA, 0.5 mM DTT, 0.5% NP40, 1× cOmplete EDTA-free proteinase inhibitor (Roche), 200 U/ml RNaseOUT) and sheared with 6 strokes through a 25 g needle. Nuclear debris were pelleted at 16,000 × *g* for 10 min at 4 °C. Fifty microliters of supernatant was flash-frozen as input control. Lysates were divided equally, precleared by rotation with 40 µl of Pierce Protein A/G beads (Thermo Scientific) for 20 min, and then incubated with an anti-FLAG antibody at 1:100 dilution or IgG antibody at a 1:40 dilution overnight at 4 °C with rotation. The next day, 40 µl of Pierce Protein A/G beads was added, and the samples were rotated for 1 h at 4 °C. Beads were washed three times in RIP buffer. A quarter of the beads were frozen down for later immunoblot analysis, where they were boiled in RIPA + lamelli buffer to extract proteins for immunoblot. The remainder of the beads were resuspended in 75 µl of elution buffer (100 mM Tris, pH 8.0, 10 mM EDTA, 1% (w/v) SDS, 200 U/ml RNaseOUT) and incubated at 37 °C for 10 min. Eluates were then removed, as they contained the mRNA of interest. Elution was repeated twice. Six microliters of 5 M NaCl and 20 µg of proteinase K (NEB) was added to the 150 µl of eluate. Eluates were then incubated at 42 °C for 1 h and then 65 °C for 1 h. Trizol LS (Invitrogen) was added, and samples were processed for RT-qPCR as described in the section below.

### Immunoblot analysis

Protein lysates were prepared from cell pellets or dishes by rinsing the cells once in PBS, adding RIPA buffer either directly to the well or cell pellet, incubating for 15 min on ice, briefly vortexing, incubating for an additional 15 min, and centrifuging at 13,000 × *g* for 5 min at 4 °C. Protein was quantitated using the Pierce BSA Protein Assay Kit (Thermo Fisher). 10 μg (unless otherwise noted, to detect GFP or mCherry) or 10–25 μg (to detect all other proteins) of lysate in Laemmli sample buffer was boiled for 5 min, resolved by SDS-PAGE using standard protocols, and transferred using the TransBlot Turbo system (Bio-Rad). Membranes were blocked in 5% milk in TBST buffer (0.1% Tween-20 in TBS buffer) for 1 h and then incubated with primary antibody (see Supplementary Data 7 for antibodies and dilutions) overnight at 4 °C. Blots were washed three times in TBST for 10 min each. The secondary antibody in 5% milk in TBST buffer was then added and incubated for 1 h at room temperature. Membranes were washed three times in TBST for 10 min at room temperature and imaged using the Clarity or Clarity Max Western ECL substrate according to the manufacturer’s protocol (Bio-Rad). Bands were quantified using the relative expression function on the Image Lab (Version 6.1) software and normalized to *Ctrl* samples within blots. Rolling disk background subtraction (disk size: 5.0 mm) in Image Lab software used for Supplementary Fig. 3a due to high background.

### RT-qPCR analysis

Cell pellets were extracted using the Trizol reagent according to the manufacturer’s protocol (Thermo Fisher Scientific), except isopropanol was added in a 1:1 ratio to each sample and 0.3 M sodium acetate was added to improve precipitation. DNase-treated RNA was further purified using the RNeasy PLUS Kit according to the manufacturer’s protocol (Qiagen). Some samples were directly extracted using the RNeasy PLUS kit, instead of Trizol, with on-column DNase step added. Thirty to 500 ng of RNA was reverse transcribed and subjected to RT-qPCR using the Gotaq 2-step RT-qPCR system (Promega) and analyzed on a CFX 384 real-time system C1000 touch thermal cycler using CFX Manager Software Version 3.1 (Bio-Rad). Cycles for amplifying *GFP, mCherry*, and *GAPDH* cDNA were 95 °C for 2 min and 40 cycles of 95 °C for 15 s and 52 °C for 1 min. All other targets were amplified at 95 °C for 2 min and 40 cycles of 95 °C for 15 s and 55 °C for 1 min. The delta-Ct method was utilized for calculating expression^80^. Cq technical replicate values were excluded that were not within 2 Cq values of one of the other two technical replicate values for a batch. See Supplementary Data 6 for excluded values. qPCR primers were synthesized by Integrated DNA Technologies (IDT). *SNHG19*^28^, *GAPDH*^51^, and *18S*^81^ primers were from previous literature. NCBI Primer-BLAST was used to generate *PIGW*, *TSPYL4*, and *TIGD1* primers.

### RNA-Seq

RNA was extracted from stable *sgZFC3H1* and *sgCtrl* cells using the RNeasy PLUS Kit (Qiagen) according to the manufacturer’s protocol. Sequencing libraries were prepared from the RNA using the KAPA mRNA Hyperprep Kit (Roche) according to the manufacturer’s protocol and sequenced on a NovaSeq X Plus 1.5B by the Duke Cancer Institute Sequencing and Genomic Technologies Shared Resource. Fifty base pair paired-end reads were aligned to the human genome (GRCh38) using the STAR aligner. Specifically, STAR version 2.7.11a was used to generate a spliced genome from the “GRCh38.primary_assembly.genome.fa” and gencode.v46.annotation.gtf” files contained within the Gencode release 46. Default parameters were used with the exception of “sjdbOverhang”, which was set to 99. Reads were aligned with “quantMode” set to GeneCounts to generate count tables. Raw counts were filtered for any gene with counts <10 across samples. Raw counts were then input into the DESeq 2 package for R^56^, which normalizes counts based on library size and calculates a log2FC value for each gene.

### Proteomics

Stable *sgZFC3H1* and sg*Ctrl* 293T cells were lysed in RIPA buffer (50 mM Tris-HCl pH 8.0, 150 mM NaCl, 1% NP40, 0.5% sodium deoxycholate, 0.1% sodium dodecyl sulfate, 1× Proteinase Inhibitor EDTA-free (Roche)), incubated on ice for at least 15 min and sonicated using Q55 Sonicator (QSonica) at 15 amplitude for 5 s, incubated at least 5 min on ice, and then sonicated. Samples were split into two: one was frozen for LC-MS/MS and the other prepared for immunoblot to confirm knockout. Samples confirmed to exhibit reduced ZFC3H1 expression were quantitated using the Bradford Assay, after which 20 μg was reduced with 10 mM DTT at 80 °C for 10 min followed by alkylation with 25 mM iodoacetamide for 30 min at room temperature. Samples were then supplemented with a final concentration of 1.2% phosphoric acid and 200 µl of S-Trap (Protifi) binding buffer (90% MeOH/100 mM TEAB). Proteins were trapped on the S-Trap micro cartridge, digested using 80 ng/µl sequencing grade trypsin (Promega) for 1 h at 47 °C, and eluted using 50 mM triethylammonium bicarbonate (TEAB), followed by 0.2% fluoroacetic acid (FA), and lastly, using 50% acetonitrile (ACN)/0.2% FA. Samples were lyophilized to dryness, then resuspended in 40 µl of 1% trifluoroacetic acid (TFA)/2% acetonitrile (MeCN). A study pool QC (SPQC) was created by combining equal volumes of each sample. Two microliters of each sample (1 µg) was diluted in 20 µl of 0.1% formic acid and loaded onto Evotips. Quantitative LC/MSMS was performed on each sample in singlicate and three replicates of an SPQC pool, using Evosep One LC interfaced to a Thermo Orbitrap Astral. The Evosep used a 30 sample-per-day method with a Pepsep 15 cm × 150 μm column (1.5 μm particle size) with a PepSep Sprayer and stainless steel (30 μm) emitter. Data collection on the Orbitrap Astral mass spectrometer was performed in a data-independent acquisition (DIA) mode of acquisition with a *r* = 240,000 (@ *m/z* 200) full MS scan every 0.6 s from *m/z* 380 to 980, with an AGC target of 500%. DIA MS/MS scans were acquired in the Astral analyzer using fixed windows of 4 *m/z* from *m/z* 380, target AGC of 500%, and max fill time of 6 ms. HCD collision energy setting of 28% was used for all MS2 scans. Raw MS data were demultiplexed and converted to *.htrms format using the HTRMS converter and processed in Spectronaut 18 (18.7.240325.55695 Biognosys). A spectral library was built using direct-DIA searches, which used a UniProt *Homo sapiens* database downloaded on 9/30/22, and appended contaminant sequences using FragPipe. Search settings included trypsin/P specificity up to 2 missed cleavages; peptide length from 7 to 52 amino acids with the following modifications: acetyl (N-term) and carbamidomethyl (Cys). For DIA analysis, default extraction, calibration, identification, and protein inference settings were used. Data were filtered at a 1% precursor and protein group false discovery rate (*q*-value). All precursors that met a *q*-value were used for local normalization based on previous reports^82^ and protein abundances were calculated using the MaxLFQ algorithm. Data were analyzed using the Perseus proteomics software (2.0.11)^57^. Data were log_2_-transformed and then filtered for any proteins that did not have at least three valid values (out of six samples) for each knockout. A two-sample test was performed using Perseus to calculate fold change between *ZFC3H1* and *Ctrl* sgRNA populations^57^.

### CAI and feature calculations

CAI was calculated for genes significantly increased in both the transcriptome and proteomic datasets upon the loss of ZFC3H1 using the GenRCA Rare Codon Analysis Tool (GenScript)^58^. Coding sequences were obtained from the NCBI gene database and input into the GenRCA Rare Codon Analysis Tool. When multiple isoforms were reported, the coding sequence of the most common was selected if listed; otherwise, isoform 1 was selected for all genes to maintain an unbiased approach. For CAI calculations between all groups, CAI was similarly calculated utilizing the same formula utilized by the GenRCA Rare Codon Analysis Tool^58^, the code for which is provided in the aforementioned GitHub repository. RBP motifs were analyzed using the MEME Suite SEA tool (5.5.8) by comparing the test set (either RNA-seq or proteomics log2FC > 0) to all genes detected by proteomics utilizing the CISBP RNA Single Species RNA, *Homo Sapiens* motif set^83^. Gene set enrichment analyses were undertaken using STRING (Version 12.0)^41^.

### Statistics

CRISPR screens were analyzed using MAGECK software^75^. RNA-seq data were analyzed using the DESeq2 (version 1.44.0) package in R (version 4.4.1)^56^. Transcripts with read counts <10 were filtered out. Proteomics data were analyzed using Perseus software^57^. To analyze proteomics data, a two-sample test was performed on all proteins with at least three valid values out of six in both groups (*Ctrl* and *ZFC3H1*). Analysis was performed on log_2_-transformed data. For all violin plot analyses, two-sided *p*-values were calculated comparing the mean of each distribution to all genes detected by proteomics distribution, using a size-matched random subsampling approach: for each group of *n* genes, *n* values were sampled at random without replacement from all genes detected by proteomics and their mean recorded, repeated 1000 times (CAI and GC content) or 10,000 times (expression, CDS length, and intron length) with a fixed random seed (123) to build a null distribution of subsampled means; the two-sided *p*-value is the fraction of subsampled means deviating from the all-gene mean by at least as much as the observed group mean (code available in Github Repository). All other data were analyzed using JMP Student Edition 19 (SAS Institute Inc). Tukey’s post hoc and sliced Student’s post hoc (for RNA stability) were performed on all multi-sample analyses that had a model *p* < 0.05. Welch’s t-tests and ANOVA were always performed as two-sided analyses, unless otherwise noted. All samples were normalized within a replicate and then combined with other replicates. *P* ≥ 0.05 was considered not significant; *p < 0.05, **p < 0.01, ***p < 0.001, and ****p < 0.0001 were considered significant thresholds. Individual dots overlaying bars correspond to individual biological replicates. Biological replicates for qPCR were obtained by averaging two to three technical replicates (see Supplementary Data 6 for technical replicates).

### Reporting summary

Further information on research design is available in the Nature Portfolio Reporting Summary linked to this article.

## Supplementary information

Supplementary Information

Description of Additional Supplementary Files

Supplementary Data 1

Supplementary Data 2

Supplementary Data 3

Supplementary Data 4

Supplementary Data 5

Supplementary Data 6

Supplementary Data 7

Reporting Summary

Transparent Peer Review File

## Source data

Source Data

## Data Availability

The sequencing reads generated in this study from the primary and validation CRISPR screens are available in the NCBI Sequence Read Archive under accession code PRJNA606038. The RNA-seq data generated in this study is available in the NCBI GEO database under accession code GSE289748. The proteomics data generated in this study is available in the UCSD MassIVE database under accession code MSV000102919. Source data are provided in this paper. MAGeCK statistics generated in this study for the primary and validation CRISPR screen are available in Supplementary Data 1 and 2. FACS plots generated in this study for flow cytometry validation are available in Supplementary Data 3. Processed RNA-seq and proteomics counts and statistics generated in this study are available in Supplementary Data 4. Uncropped immunoblots generated in this study are available in Supplementary Data 5. Raw Cq values from RT-qPCR generated in this study are available in Supplementary Data 6. Source data are provided with this paper.

## Code availability

Original code for CAI and feature analysis is available on GitHub [https://github.com/sijinsmart/CAI-analysis-code]^84^.
