# Supplementary material for: The PAXT connection is a modifier of rare codon-enriched protein expression
Source: Nat Commun. 2026 Sep 17;17:10928. doi: 10.1038/s41467-026-77792-7 (PMC13680609; doi:10.1038/s41467-026-77792-7)
Supplement: Supplementary file 1 [file 41467_2026_77792_MOESM10_ESM.pdf]

Corresponding author(s): Christopher Counter
Last updated by author(s): Sep 2, 2026

Reporting Summary

Nature Portfolio wishes to improve the reproducibility of the work that we publish. This form provides structure for consistency and transparency in reporting. For further information on Nature Portfolio policies, see our Editorial Policies and the Editorial Policy Checklist.

Statistics

For all statistical analyses, confirm that the following items are present in the figure legend, table legend, main text, or Methods section.

n/a	Confirmed	
☐	☒	The exact sample size (*n*) for each experimental group/condition, given as a discrete number and unit of measurement
☐	☒	A statement on whether measurements were taken from distinct samples or whether the same sample was measured repeatedly
☐	☒	The statistical test(s) used AND whether they are one- or two-sided<br>*Only common tests should be described solely by name; describe more complex techniques in the Methods section.*
☒	☐	A description of all covariates tested
☐	☒	A description of any assumptions or corrections, such as tests of normality and adjustment for multiple comparisons
☐	☒	A full description of the statistical parameters including central tendency (e.g. means) or other basic estimates (e.g. regression coefficient) AND variation (e.g. standard deviation) or associated estimates of uncertainty (e.g. confidence intervals)
☐	☒	For null hypothesis testing, the test statistic (e.g. *F*, *t*, *r*) with confidence intervals, effect sizes, degrees of freedom and *P* value noted<br>*Give P values as exact values whenever suitable.*
☒	☐	For Bayesian analysis, information on the choice of priors and Markov chain Monte Carlo settings
☒	☐	For hierarchical and complex designs, identification of the appropriate level for tests and full reporting of outcomes
☒	☐	Estimates of effect sizes (e.g. Cohen's *d*, Pearson's *r*), indicating how they were calculated

Our web collection on statistics for biologists contains articles on many of the points above.

Software and code

Policy information about availability of computer code

Data collection

Flow cytometry acquisition: BD FACSDiva 9.0
Immunoblot acquisition: Image Lab 6.1
qPCR acquistion: Bio-Rad CFX Manager Software Version 3.1

Data analysis

Original code was generated for CAI and feature analysis. It is available at https://github.com/sijinsmart/CAI-analysis-code.

CRISPR-Cas9 sgRNA screen analysis: MAGeCK 0.5.8 (https://sourceforge.net/projects/mageck/)
Western blot quantification: Image Lab 6.1 (https://www.bio-rad.com/en-us/product/image-lab-software)
Figure Creation: GraphPad Prism 10.4.1 (https://www.graphpad.com/)
Statistical Analysis: JMP Student 19 (https://www.jmp.com/en/academic/jmp-student-edition)
Proteomics: Perseus 2.0.11.0 (https://maxquant.net/perseus/)
RNA Seq Analysis: DESeq2 1.44.0 (https://bioconductor.org/packages/release/bioc/html/DESeq2.html) in R 4.4.1 (https://www.r-project.org/)
RBP Motif Analysis: Meme Suite SEA 5.5.8 (https://meme-suite.org/meme/tools/sea)
STRING Analysis: String Version 12.0 (https://string-db.org/), Node image creation: Cytoscape Version 3.10.3

CRISPR Screen Analysis: Reads were mapped to sgRNA references and analyzed by MAGeCK. The output file with gene summary was used for downstream analysis.

Western Blot Quantification: Bands were quantified using the relative expression function in Image Lab. Expression was normalized both to loading control (either B-tubulin or GAPDH) and Ctrl sgRNA cells.

Statistical Analysis: All statistical analysis was run in JMP Student Edition, besides CAI and feature analysis (see code above). "Fit Model" was always used for ANOVA and Welch's t-test (two-tail analysis, unless otherwise noted). Number of variables for ANOVA indicated in figure legends as X-way ANOVA with X being the number of variables. p<0.05 considered significant and cutoff for performing Tukey's post-hoc tests or spliced Student's t-test post-hoc tests after ANOVA.

RNA Seq Analysis: 50 base pair paired-end reads were aligned to the human genome (GRCh38) using the STAR aligner. Specifically, STAR version 2.7.11a was used to generate a spliced genome from the "GRCh38.primary_assembly.genome.fa" and gencode.v46.annotation.gtf" files contained within the Gencode release. Default parameters were used with the exception of "sjdbOverhang", which was set to 99. Reads were aligned with "quantMode" set to GeneCounts to generate count tables. Raw counts were censored for any gene with counts > 10. Raw counts were then input into the DESeq 2 package for R, which normalized counts based on library size and calculates a log2FC value for each gene.

Proteomics Analysis: Raw MS data were demultiplexed and converted to *.htrms format using HTRMS converter and processed in Spectronaut 18 (18.7.240325.55695 Biognosys). A spectral library was built using direct-DIA searches which used a Uniprot Homo sapiens database downloaded on 9/30/22, and appended contaminant sequences using FragPipe. Search settings included trypsin/P specificity up to 2 missed cleavages; peptide length from 7-52 amino acids with the following modifications: acetyl (N-term) and carbamidomethyl(Cys). For DIA analysis, default extraction, calibration, identification, and protein inference settings were used. Data were filtered at a 1% precursor and protein group false discovery rate (q-value). All precursors that met a q-value were used for local normalization based on previous reports61 and protein abundances calculated using the MaxLFQ algorithm. Data were analyzed using the Perseus proteomics software. Data were log2-transformed and then filtered for any proteins that did not have at least three valid values (out of six samples) for each knockout. A two-sample test was performed using Perseus to calculate fold change between ZFC3H1 and Ctrl sgRNA populations.

For manuscripts utilizing custom algorithms or software that are central to the research but not yet described in published literature, software must be made available to editors and reviewers. We strongly encourage code deposition in a community repository (e.g. GitHub). See the Nature Portfolio guidelines for submitting code & software for further information.

Data

Policy information about availability of data

All manuscripts must include a data availability statement. This statement should provide the following information, where applicable:

- Accession codes, unique identifiers, or web links for publicly available datasets
- A description of any restrictions on data availability
- For clinical datasets or third party data, please ensure that the statement adheres to our policy

The sequencing reads generated in this study from the primary and validation CRISPR screens are available in the NCBI Sequence Read Archive under accession code
The sequencing reads generated in this study from the primary and validation CRISPR screens are available in the NCBI Sequence Read Archive under accession code
PRJNA606038 [https://www.ncbi.nlm.nih.gov/sra/?term=PRJNA606038]. The RNA seq data generated in this study is available in the NCBI GEO database under accession code GSE289748 [https://www.ncbi.nlm.nih.gov/geo/query/acc.cgi?acc=GSE289748]. The proteomics data generated in this study is available in the UCSD MassIVE database under accession code MSV000102919 [https://massive.ucsd.edu/ProteoSAFe/dataset.jsp?task=d24d0ed9dcc24a13be37e8b3b662a85b]. Source Data are provided in this paper. MAGeCK statistics generated in this study for the primary and validation CRISPR screen are available in Supplementary Data 1 and 2. FACS plots generated in this study for flow cytometry validation are available in Supplementary Data 3. Processed RNA seq and proteomics counts and statistics generated in this study are available in Supplementary Data 4. Uncropped immunoblots generated in this study are available in Supplementary Data 5. Raw Cq values from RT-qPCR generated in this study are available in Supplementary Data 6.

Research involving human participants, their data, or biological material

Policy information about studies with human participants or human data. See also policy information about sex, gender (identity/presentation), and sexual orientation and race, ethnicity and racism.

Reporting on sex and gender	*Use the terms sex (biological attribute) and gender (shaped by social and cultural circumstances) carefully in order to avoid confusing both terms. Indicate if findings apply to only one sex or gender; describe whether sex and gender were considered in study design; whether sex and/or gender was determined based on self-reporting or assigned and methods used.*<br>*Provide in the source data disaggregated sex and gender data, where this information has been collected, and if consent has been obtained for sharing of individual-level data; provide overall numbers in this Reporting Summary. Please state if this information has not been collected.*<br>*Report sex- and gender-based analyses where performed, justify reasons for lack of sex- and gender-based analysis.*
Reporting on race, ethnicity, or other socially relevant groupings	*Please specify the socially constructed or socially relevant categorization variable(s) used in your manuscript and explain why they were used. Please note that such variables should not be used as proxies for other socially constructed/relevant variables (for example, race or ethnicity should not be used as a proxy for socioeconomic status).*<br>*Provide clear definitions of the relevant terms used, how they were provided (by the participants/respondents, the researchers, or third parties), and the method(s) used to classify people into the different categories (e.g. self-report, census or administrative data, social media data, etc.)*<br>*Please provide details about how you controlled for confounding variables in your analyses.*
Population characteristics	*Describe the covariate-relevant population characteristics of the human research participants (e.g. age, genotypic information, past and current diagnosis and treatment categories). If you filled out the behavioural & social sciences study design questions and have nothing to add here, write "See above."*

Recruitment	*Describe how participants were recruited. Outline any potential self-selection bias or other biases that may be present and how these are likely to impact results.*
Ethics oversight	*Identify the organization(s) that approved the study protocol.*

Note that full information on the approval of the study protocol must also be provided in the manuscript.

Field-specific reporting

Please select the one below that is the best fit for your research. If you are not sure, read the appropriate sections before making your selection.

☒ Life sciences ☐ Behavioural & social sciences ☐ Ecological, evolutionary & environmental sciences

For a reference copy of the document with all sections, see nature.com/documents/nr-reporting-summary-flat.pdf

Life sciences study design

All studies must disclose on these points even when the disclosure is negative.

Sample size	No statistical methods were used to predetermine sample size. At least three biological replicates were run per experiment to ensure reproducibility and consistency of findings. Sample size is denoted in figure legends. Covariates were not applicable because experiments were performed in a controlled cell culture system.
Data exclusions	No data was excluded, except for Cq technical replicate values that were not within 2 Cq values of one of the other two technical replicate values for a batch. See Supplementary Data 6 for excluded values.
Replication	The experiments were performed in a minimum of three biological replicates. Two to three technical replicates were averaged for each biological replicate in qPCR experiments. Replicate values are available in source data and represented on graphs.
Randomization	No randomization was applied because experimental groups were defined by the molecular perturbation being tested.
Blinding	Blinding was not used. Experimental groups were defined by the molecular perturbation being tested and knowledge of the experimental condition was required during experimental procedures.

Reporting for specific materials, systems and methods

We require information from authors about some types of materials, experimental systems and methods used in many studies. Here, indicate whether each material, system or method listed is relevant to your study. If you are not sure if a list item applies to your research, read the appropriate section before selecting a response.

Materials & experimental systems

n/a	Involved in the study	
☐	☒	Antibodies
☐	☒	Eukaryotic cell lines
☒	☐	Palaeontology and archaeology
☒	☐	Animals and other organisms
☒	☐	Clinical data
☒	☐	Dual use research of concern
☒	☐	Plants

Methods

n/a	Involved in the study	
☒	☐	ChIP-seq
☐	☒	Flow cytometry
☒	☐	MRI-based neuroimaging

Antibodies

Antibodies used

Target,Manufacturer,Catalog, Lot, Dilution
Puromycin DSHB PMY-2A4-s 3/21/24-31ug/mL 1:100
GFP, Invitrogen, A21311, 251333, 1:10000
ZFC3H1, Novus, NB100-68267, A4, 1:1000
mCherry (RFP), MBL, PM005, 046, 1:1000
PABPN1, Proteintech, 66807-1-Ig, 10019974 (barcode), 1:5000
GAPDH, Santa Cruz, SC-365062, C3021, 1:5000
B-tubulin, Sigma-Aldrich, T4026-2 mL, 0000239424, 1:1000
Mouse HRP (secondary), Invitrogen, 62-6520, YF375236, 1:5000
Rabbit HRP (Secondary), Invitrogen, A24537, 62-147-102418, 1:5000
Lamin B1, Cell Signaling, 13435S, 2, 1:1000
MTREX (SKIV2L2), Bethyl, A300-614A-T, 4, 1:2000
SSR1/TRAP-alpha, Bethyl, A304-978A-T, 1, 1:2500
mouse IgG, Santa Cruz, sc-2025, D2022, 1:40 (immunoprecipitation)

FLAG, Sigma-Aldrich, F1804-1mg, SLCD6338, 1:1000 (immunoblot), 1:100 (immunoprecipitation)
Mouse HRP (secondary, light chain-specific), Jackson Immuno Research, 115-035-174, 103872, 1:5000 (used for PABPN1 immunoprecipitation blot only)

Validation

Antibodies were validated by respective suppliers. ZFC3H1 and PABPN1 antibodies were confirmed by sgRNA depletion and rescue with plasmid (validated by immunoblot). GFP and RFP antibodies were confirmed by adding in varying concentrations of GFP or mCherry-expressing plasmids into cells (validated by immunoblot). GFP and mCherry were further verified by transfecting in either the single reporters or dual reporters or neither to confirm correct band size and specificity (not shown in manuscript).

Eukaryotic cell lines

Policy information about cell lines and Sex and Gender in Research

Cell line source(s)	293T cells were obtained from the Duke University Cell Culture Facility (CCF) and maintained in the lab.
Authentication	Cell lines were not independently authenticated after obtaining from CCF.
Mycoplasma contamination	Cell lines were tested monthly for mycoplasma contamination using the Mycoalert Mycoplasma Detection Kit (Lonza Biosciences). Cells tested negative.
Commonly misidentified lines (See ICLAC register)	No commonly misidentified cell lines were used.

Plants

Seed stocks	*Report on the source of all seed stocks or other plant material used. If applicable, state the seed stock centre and catalogue number. If plant specimens were collected from the field, describe the collection location, date and sampling procedures.*
Novel plant genotypes	*Describe the methods by which all novel plant genotypes were produced. This includes those generated by transgenic approaches, gene editing, chemical/radiation-based mutagenesis and hybridization. For transgenic lines, describe the transformation method, the number of independent lines analyzed and the generation upon which experiments were performed. For gene-edited lines, describe the editor used, the endogenous sequence targeted for editing, the targeting guide RNA sequence (if applicable) and how the editor was applied.*
Authentication	*Describe any authentication procedures for each seed stock used or novel genotype generated. Describe any experiments used to assess the effect of a mutation and, where applicable, how potential secondary effects (e.g. second site T-DNA insertions, mosiacism, off-target gene editing) were examined.*

Flow Cytometry

Plots

Confirm that:

☒ The axis labels state the marker and fluorochrome used (e.g. CD4-FITC).

☒ The axis scales are clearly visible. Include numbers along axes only for bottom left plot of group (a 'group' is an analysis of identical markers).

☒ All plots are contour plots with outliers or pseudocolor plots.

☒ A numerical value for number of cells or percentage (with statistics) is provided.

Methodology

Sample preparation	For CRISPR screen, 293T cells expressing ERK1/2 substrate CRISPR sgRNA library were transfected with reporters. 48 hours later, cells were trypsinized and collected in PBS with 1% BSA and 2mM EDTA prior to sorting. For flow cytometry, 48 hours after transfection with reporters,
Instrument	CRISPR screen: MoFlo Astrios sorter (Beckman Coulter) Flow cytometry validation: LSRFortessa X-20 (BD)
Software	For flow cytometry validation, BD FACSDiva 9.0
Cell population abundance	For CRISPR screen, the purity of sorted cells were higher than 95% detected via flow cytometer after sorting. For flow cytometry validation, 50,000 live cells were analyzed for each replicate.
Gating strategy	For CRISPR screen, gating strategy for GFP and mCherry was previously published in Peterson et al, Scientific Reports, 2020 (Ref 34 in manuscript). Debris excluded by FSC-A and SSC-A; Doublets excluded by FSC-A and FSC-H; GFP/mCherry-positive gates were defined using un-transfected cells as negative control; For ERK1/2 substrate CRISPR sgRNA library screen, GFP/mCherry-high or low population in BRAFV600E-transfected cells were defined using luciferase-transfected cells as control.

For flow cytometry validation, debris excluded by FSC-A and SSC-A, singlets identified by FSC-A and FSC-H, GFP and mCherry gates drawn using untransfected controls, GFP or mCherry transfected cells were used to adjust compensation and gates if necessary, double-positive cells (GFP and mCherry) were identified from singlets and average expression of each was calculated from these populations.

☒ Tick this box to confirm that a figure exemplifying the gating strategy is provided in the Supplementary Information.
