# Supplementary material for: The PAXT connection is a modifier of rare codon-enriched protein expression
Source: Nat Commun. 2026 Sep 17;17:10928. doi: 10.1038/s41467-026-77792-7 (PMC13680609; doi:10.1038/s41467-026-77792-7)
Supplement: Supplementary file 2 [file 41467_2026_77792_MOESM11_ESM.pdf]

nature portfolio

Peer Review File

The PAXT connection is a modifier of rare codon-enriched protein expression

Corresponding Author: Professor Christopher Counter

This file contains all reviewer reports in order by version, followed by all author rebuttals in order by version.

Version 0:

Reviewer comments:

Reviewer #1

(Remarks to the Author)
Via an sgRNA screening approach, Niehoff et al. identify an influence of the PAXT subunits ZFC3H1 and PABPN1 on the expression of rare-codon encoded proteins in human cells. sgRNA targeting ZFC3H1 or PABPN1 leads to increased rare-codon protein expression, which cannot simply be explained by the preferential accumulation of rare-codon mRNAs. Overall, the manuscript presents an interesting body of work. However, the transcriptomics and proteomics datasets are under-analyzed, and the manuscript would benefit from a more comprehensive data presentation and interpretation. Additionally, the discussion lacks a better reasoning of the proposed working models.
With proper revision, this paper might be suitable for publication in Nature Communications.

Major points
Lack of a mechanistic model explaining the observed phenomenon
Despite speculative suggestions in the Discussion section, the authors need to perform more groundwork to rationalize the observed phenomenon. Remaining question include: How are ZFC3H1 and PABPN1 capable of selectively retaining mRNAs with rare codons? Are there e.g. specific motifs or other features in the identified RNAs? Are rare-codon encoding RNAs different in their synthesis/decay/export rates? Here, metabolic labelling approaches may yield insights, even if done only for reporter constructs.
Manuscript structure and flow

- Some of the figures could easily be combined. For example figures 2 and 3, which use the same samples for protein and RNA expression analyses, but instead are presented separately. Similarly, figures 6 and 7 could be combined with some of the validation panels (e.g., PCA plots and ZFC3H1 protein counts) being moved to supplementary figures.
Experimental results
- In the screen in figure 1, what are the 204 putative targets selected for the second library? How was the selection done and which thresholds were applied for the GFPlo and GFPhi populations?

- In fig. 1b and 1c, the x-axes naming is not informative. Moreover, including a vertical ‘zero’ line would more clearly visualize the targets causing the upregulation or downregulation of the GFPrare reporter.

- The knockdown efficacy in fig. 2a is not very strong. If possible, using an alternative approach to achieve a better depletion efficiency could be used to validate the result.

- The validation of the fractionation experiment in fig. 4a should be a supplementary figure and it should also include mainly cytoplasmic RNAs as controls, to ensure that the nuclear fraction is not contaminated with RNAs from the cytoplasmic/extra-nuclear fraction.

- The depletion efficacy shown in fig. S3 is not convincing. In addition, the RNA phenotype is considerably lower, compared to that observed in fig. 3a and fig. 4b for GFPrare in WCE (from average fold change of 4 to 2, respectively). The lack of significance in this panel could at least partly be due to only mild depletion.

- Overall, but especially visible in figures 2, 3, and 5, the observed effects tend to be small and with a lack of consistency between the results obtained with the different reporter constructs (GFPrare and mCherryrare). Might this also be a reflection

of poor and variable depletion efficacies?

- In fig. 6d, the main text and the figure legend are not in agreement, and it is not clear if the plot is considering only mRNAs or the whole transcriptome. Both the plot itself and the legends should be more informative.
o “Also consistent with the role of the PAXT connection in RNA degradation ZFC3H1 depletion was associated with a general increase in mRNA levels (Figure 6D).”
o “(d) MA (Microarray – M (log ratio) vs A (mean average)) plot of ZFC3H1 vs Ctrl sgRNA 293T cells (d), plotting mean of normalized counts for each transcript versus its log fold change.”

- In figure 7, log2FC > 0 is not necessarily high stringency. Moreover, the proteomics and transcriptomics data are under-analyzed. Among the selected mRNAs targeted by ZFC3H1, are there any common features (e.g., length, intron content, motifs) that could hint to a mechanism for targeting these transcripts for decay/retention?

- As suggested in the discussion, one possibility is that ZFC3H1 targets mRNAs regardless of their codon enrichment, but as rare-codon enriched mRNAs are normally less expressed, this accumulation would lead to a significant increase in protein expression. This could already be tested with the available data, comparing all ZFC3H1 RNA targets (regardless of the phenotype on the protein levels) and analyzing if there is a correlation between accumulation of these mRNAs, their protein levels and their respective codon content.

- The proteomics analysis might be improved by the labelling of nascent proteins upon ZFC3H1 depletion. This could e.g. be dome using SILAC for quantification of nascent protein levels, potentially allowing a better sensitivity for identifying ZFC3H1 targets.
Minor points
- Fig 1e is not informative and could be removed, as ARS2 or CBC and other PAXT components are not present in the screening.
- Typo in ‘extra-nuclear factions’ in page 5.
- In figure 4, would the extra-nuclear fraction simply be represented by the cytoplasm? Is there a special reason for the different denomination?
- The writing of the manuscript is often redundant, as exemplified from the text:
o “While PABPN1 and MTREX are found in a number of other complexes, such as a polyadenylation and the NEXT complexes, respectively, ZFC3H1 is the only known subunit specific to the PAXT connection.”
o “… we focused on ZFC3H1, as it is the only protein unique to the PAXT connection. In more detail, ZFC3H1 is not known to function outside of the PAXT connection.”
o “… we repeated the above analysis, except reduced expression of PABPN1, another subunit of the PAXT connection…”
- The figures are often not self-explanatory, and the figure legends are not always well-described, especially for the last figures (fig. 6 and fig. 7).

Reviewer #2

(Remarks to the Author)
In the submitted manuscript, Niehoff and colleagues provide good evidence that the PAXT complex suppresses the protein expression from mRNAs derived from genes that have an abundance of rare codons. This is partially through the nuclear retention and partially through decay of the target mRNAs. The work is well presented and would be of general interest to the readership of Nature Communications. I would be in general support of its publication provided that the authors address the following issues:

1) The fractionation experiments (Figure 4) are not very clean. In particular, the nuclear fraction seems heavily contaminated with cytosol (called “extra-nuclear” by the authors). The authors should redo this experiment with a much more rigorous protocol. In particular I refer them to the RNA Frac-Seq (see in Materials & Methods of Lee et al., NAR 2020 - PMID: 33091126), which uses 1% Triton X-100 and 0.2% sodium deoxycholate detergents – although the exact percentage of these detergents should be tested and adjusted. Note that the nuclei should be extensively washed or they will contain cytoplasmic contamination. I also advise the authors to keep some of the fractions (pre-TRIZOL treatment) to analyze by immunoblot for nuclear, ER and cytoplasmic proteins.

2) The authors should determine whether ZFC3H1-depletion causes the co-depletion of other PAXT proteins and vice versa. If components tend to be co-depleted (as observed by some groups), this will not change the authors' interpretations, but this information is important.

3) I may have missed this, but the authors should indicate the CAI values of their various GFP and mCherry reporters (are these the dotted lines in Figure 7C?)

4) In Mordstein et al., Cell Syst 2020, the authors report that GC-content in the first half of the mRNA plays a key role in modulating their nuclear export, especially when the mRNA is generated from an intron-poor gene. Indeed, this is in line with the findings of other groups (see Palazzo et al., RNA Biol 2024 for an overview) and in line with the recent report that ZFC3H1 binding/activity is affected by GC-content near the 5' end of the mRNA (Fan et al., Mol Cell 2024). Note that rare-codons are typically GC-poor. Given these prior findings, are ZFC3H1-sensitive mRNAs derived from intron-poor genes? Are they GC-poor? Is the CAI (or GC-content) at the 5’ end of the ORF more predictive of ZFC3H1-sensitivity? These questions should be easily answered given the sequencing data.

Reviewer #3

(Remarks to the Author)
Niehoff et al screened an sgRNA library to identify genes involved in expression of a rare codon reporter. This analysis identified 10 genes, including ZFC3F1 and PABPN1 subunits of PAXT. The effect of ZFC3H1 and PABPN1 on expression of the rare codon reporter compared to the common codon reporter were tested by RNAi known-down, with and without rescue using a plasmid expressing an RNAi-resistant mutant. The authors concluded that ZFC3H1 and PABPN1 specifically affected expression of the rare codon reporter. RNA analysis was also performed in parallel. However, many of these results are inconclusive. Finally, they tested whether ZFC3H1 affects expression of endogenous rare codon-enriched transcripts. The results suggested that transcripts and proteins upregulated following loss of ZFC3H1 contain more rare codons than those found on average in the human transcriptome. While the results potentially highlight a novel role for ZFC3H1 and PAXT, the overall conclusion that these factors specifically affect expression of rare codon transcripts seems premature.

A general problem in figures using the reporter system is that the expression of ZFC3H1 is quite variable between duplicate samples, and also between the different knock-down, over-expression and rescue conditions, and also between the converse configurations of the reporters (2a vs 2c for example). To point out an example, in Fig 2a, the expression of ZFC3H1 doesn't strictly follow the experimental condition. There is less ZFC3H1 in rep 2 of the -/- control, only rep 2 of -/+ seems to be in fact over-expressed, and neither rescue rep is really rescued, and so on. This in seen in Fig 2a, Fig 2c and supp figs. This is not necessarily a problem as it's extremely difficult to achieve perfect expression profiles in such experiments, the problem is that the expression of the rare codon doesn't always follow the expression of ZFC3H1.

The authors also conclude that the effect is specific for the rare codon reporter. However, there is a significant effect on the common codon reporter as well, in Fig 2a. How well is the common reporter (GFP or Cherry) expressed relative to the rare codon reporter? If it is much more highly expressed, this could make it difficult to quantitatively compare the effect of the knock-downs on expression of the common codon reporter. Since the increase in rare vs common reporter expression following loss of ZFC3H1 leads to the suggestion that the effect is specific for rare codon reporters, it is important to resolve this issue.

For the experiments using Western blot analysis, on which the major conclusions of the manuscript are based, it would be preferable to use a more quantitative approach. In addition, at least some samples could be tested in dilution series to validate the quantification given by band density analyzing software. This may seem old fashioned, but it is important to be sure that the differences detected following quantification are real and not due to antibody saturation of other known western blotting artefacts.

Next, the authors analyzed RNA levels corresponding the reporter plasmids. RNA levels largely mirrored those of protein levels, or rather, protein levels mirrored those of RNA. As above, it is important to know the relative expression level of the common codon reporter compared to the rare codon reporter. Absolute levels of RNAs could be shown, on separate graphs if the expression levels are not equal.

In fig 4b, siZFC3H1 is not significantly different to siCtrl whereas in Fig 3 a and b, it is. How reproducible is the result ?
In Fig 5, it is difficult to compare the 'significance' of the western blot results with those of RNA quantification, since the RNA experiments appear to have been calculated using only 2 replicates, whereas protein results use 3 replicates.

In Fig 7, since the authors have both RNA-seq and proteomics data, it would have been interesting to identify genes that are increased at the protein level but whose transcripts are not affected, which might provide candidates to further address a potential role in RNA export or translation. Concerning the statistical analysis of this data set, given the large difference in N for each category (97 for ZFC3H1 targets versus > 10,000 transcripts for the human transcriptome presumably), the analysis should be performed using a bootstrapping method, rather than simply calculating the median CAIs, if I understand the statistical method correctly.

To strengthen the conclusions of the study, candidate transcripts (3 to 5) identified in Fig 7 should be validated using the cell lines used for the reporter plasmids.

Minor points:
Line 96 and 97, should be 'favor', not 'favored'.
Line 144: 'independently twice' means 'in duplicate' ?
Line 265, 273 : most readers know what 'both' means, no need to italicize and underline it.
Line 280: 'to put this into context' could be removed. Instead, it would be more informative to clarify 'significantly' by including the p-val obtained.
Line 297: should be 'highly'

Note to authors: An early career researcher participated in the co-review initiative.
I co-reviewed this manuscript with one of the reviewers who provided the listed reports. This is part of the Nature Communications initiative to facilitate training in peer review and to provide appropriate recognition for Early Career Researchers who co-review manuscripts.

Version 1:

Reviewer comments:

Reviewer #1

(Remarks to the Author)
The authors have addressed some of my previous concerns, significantly improving the manuscript, and I appreciate the efforts collecting and analysing new data. However, I remain unconvinced by the variable effects on rare-codon RNA levels (Figure 2e,f) and degradation rates (Figure 3a), upon ZFC3H1 and PABPN1 loss, as compared to that of the rare-codon protein reporters. It seems to me that alternative mechanisms, such as differences in nuclear export rates or translation efficiencies, might better explain the observed phenotypes. Given that both ZFC3H1 and PABPN1 were shown to influence nuclear retention of pA+ RNAs, the authors could use their fractionation data to quantify whether more common/rare-codon reporter transcripts would be preferably located to the nucleus or cytoplasm in normal condition as compared to the ZFC3H1-perturbed condition. The data provided in Figure 3b does not readily estimate if RNA leakage from the nucleus has a significant effect. In addition, the PABPN1 RIP-qPCR results of Figure 5g might be explained by nuclear retention of rare-codon mRNAs given that PABPN1 is localized to the nucleus? There is also the possibility that an indirect effect of ZFC3H1 loss would lead to preferential translation of rare-codon proteins. In this vein, it has been shown that leakage of spurious transcripts into the cytoplasm upon ZFC3H1 loss affects global translation levels. The authors could explore (or at least discuss) these possibilities.
Minor Issue:
The manuscript will benefit from standardisation of data presentation, like using similar ways to show statistical significance (asterisks) across the manuscript.

(Remarks on code availability)

Reviewer #2

(Remarks to the Author)
The authors did their best to answer all of the comments, however my enthusiasm for the work has dampened substantially. Notably, in Figure 4 and Supplemental Figure 10, the authors demonstrate that their assay is dependent on the level of mRNA expression. This is especially striking in Supplemental Figure 9b where data from “low” mCherry-com looks essentially the same as GFP-rare. At the same time, ZFC3H1 has almost no effect for the “High Reporter” experiments (Figure 4a, Supplemental Figure 10a). These findings calls into question the interpretation of the screen (Figure 1) and of many other experiments. Could it simply be that mRNAs expressed at lower levels are more sensitive to ZFC3H1? If so this effect could have nothing to do with the presence of rare codons.

There are other inconsistencies that have popped up in the revision. Despite the fact that all reporters are affected (Figure 2e-f), when mRNA turnover kinetics are measured, only minor changes are noted (Figure 2a). I suspect that any potential change in mRNA turnover occurs only at early timepoints in the nucleus (where the PAXT complex resides), whereas a sizeable fraction of the mRNA analyzed in Figure 2a is cytoplasmic (i.e. it has already escaped potential decay by the PAXT complex). Perhaps a pulse-chase experiment with EU would give more reliable results. I am also not convinced about the fractionation data – for example, the mCherry-rare reporter, which shows a substantial increase in protein (Figure 2c) and a 2.5 fold increase in mRNA levels (Figure 2f), shows no increase in cytoplasmic levels (“EN” Figure 3b). I suspect that there is something off in the fractionation results. During fractionation, RNA is extremely susceptible to non-specific decay, and levels of 28S and 18S should be monitored to ensure that the RNA survived the procedure. In addition, from the written protocol, it is not clear what standard was used for the RT-qPCR to determine these RNA levels in the various fractions. The authors may want to monitor the nuclear/cytoplasmic ratios using Fluorescence In Situ Hybridization (FISH).

Other problems:
- Supplemental Figure 7. The Lamin B1 protein is hard to evaluate in the blot from the Ctrl sgRNA samples - there is no reliable band present.
- Supplemental Figure 8. I’m not sure how GFP and mCherry mRNAs can be compared to each other and normalized in the manner presented. These are presumably amplified using different primers, which will likely have different amplification rates.
- Page 7. “We then censored for transcripts” I would not use “censored” in this sentence – perhaps “conducted a census”?
- Figure 6b. “FC > 0” should be “log2FC > 0”
- Figure 6d and Supplemental Figures 18 and 22. Something is wrong with the violin plots.
- Figure 7. The authors do not provide evidence that PABPN1 recruits ZFC3H1 to its purported substrates.
- Supplemental Figure 12. I’m surprized how much protein remains in the CRISPR’d cells.

(Remarks on code availability)

Reviewer #3

(Remarks to the Author)
The authors have adequately addressed the concerns raised during review.

(Remarks on code availability)

Version 2:

Reviewer comments:

Reviewer #1

(Remarks to the Author)
Major point:
The authors have addressed my remaining comments from the second round of review. However, they did not explain in the rebuttal what specific changes were made in response to my concerns regarding the results in Figures 2e–f and 3a.
Minor point:
The manuscript would still benefit from a more consistent presentation of statistical significance. For example, the formatting of the significance asterisks differs between Figures 2–6 and Figure 7.

(Remarks on code availability)

Reviewer #2

(Remarks to the Author)
The concerns were mostly addressed.
For clarity the authors should just make the box plot in Supplemental Figure 21a ("all proteins") more visible (it is currently hidden behind the data points). They should also change the word "censored", which appears 3 times in the current text. "Censored" means to remove or alter communications by an authority that wants to limit the exchange of ideas, and not to conduct a census. The authors should use more appropriate wording, such as "conducted a census" or "analyzed".

(Remarks on code availability)
NA

Open Access This Peer Review File is licensed under a Creative Commons Attribution 4.0 International License, which permits use, sharing, adaptation, distribution and reproduction in any medium or format, as long as you give appropriate credit to the original author(s) and the source, provide a link to the Creative Commons license, and indicate if changes were made.
In cases where reviewers are anonymous, credit should be given to 'Anonymous Referee' and the source.
The images or other third party material in this Peer Review File are included in the article’s Creative Commons license,

unless indicated otherwise in a credit line to the material. If material is not included in the article's Creative Commons license and your intended use is not permitted by statutory regulation or exceeds the permitted use, you will need to obtain permission directly from the copyright holder.
To view a copy of this license, visit https://creativecommons.org/licenses/by/4.0/

Reviewer #1 (Remarks to the Author)

Major comment #1: "Lack of a mechanistic model explaining the observed phenomenon. Despite speculative suggestions in the Discussion section, the authors need to perform more groundwork to rationalize the observed phenomenon. Remaining question include: How are ZFC3H1 and PABPN1 capable of selectively retaining mRNAs with rare codons? Are there e.g. specific motifs or other features in the identified RNAs? Are rare-codon encoding RNAs different in their synthesis/decay/export rates? Here, metabolic labelling approaches may yield insights, even if done only for reporter constructs."

As requested, we determined the *1*) stability of reporter mRNAs over time after transcriptional inhibition with Actinomycin D in the absence and presence of ZFC3H1 (*see* **Figure 3A**), *2*) relative binding of reporter mRNAs to PABPN1 by RNA immunoprecipitation (**Figure 5G**), and *3*) effect of increasing reporter levels on codon-specific expression upon ZFC3H1 loss (**Figure 4A,B**). Additionally, we analyzed all the endogenous transcripts that were more highly expressed at the mRNA and/or protein level upon loss of ZFC3H1 for RNA-binding motifs (**Supplementary Tables 3,4**). Collectively, these experiments suggest a model (**Figure 7**) whereby ZFC3H1 preferentially degrades rare codon-enriched reporter transcripts, potentially through preferential binding of PABPN1. In rare codon-enriched transcripts expressed at low levels, this is amplified at the protein level, giving rise to observed bias of on higher rare codon-enriched expression upon ZFC3H1 loss.

Major comment #2: "Some of the figures could easily be combined. For example figures 2 and 3, which use the same samples for protein and RNA expression analyses, but instead are presented separately. Similarly, figures 6 and 7 could be combined with some of the validation panels (e.g., PCA plots and ZFC3H1 protein counts) being moved to supplementary figures."

As requested, we made the suggested changes to the structure of the manuscript. Figures 2 and 3 are now combined (**Figure 2**) while Figure 6 is now in the supplement (**Supplementary Figure 15**).

Major comment #3: "In the screen in figure 1, what are the 204 putative targets selected for the second library? How was the selection done and which thresholds were applied for the GFPlo and GFPhi populations?"

As requested, we now list the identity of the 204 targets in a new sheet in the previous **Supplementary Data 1**. We also now include in the results and materials and methods the gating used to sort GFP^{lo} and GFP^{hi} cells.

Major comment #4: "In fig. 1b and 1c, the x-axes naming is not informative. Moreover, including a vertical 'zero' line would more clearly visualize the targets causing the upregulation or downregulation of the GFPrare reporter."

As requested**,** we added more information to the x-axis titles (along with more information in the results section and figure legend) and added the suggested vertical line.

Major comment #5: "The knockdown efficacy in fig. 2a is not very strong. If possible, using an alternative approach to achieve a better depletion efficiency could be used to validate the result."

As requested**,** we tested and found that sgRNA-mediated inactivation of ZFC3H1 resulted in an increase in rare over common reporters as determined by simultaneously testing both immunoblot at progressively lower amount of lysates (**Supplementary Figure 3A**) AND by FACS analysis (**Supplementary Figure 3B**). We also tested and found that reducing ZFC3H1 expression by siRNA (as confirmed by immunoblot, **Supplementary Figure 5A**) preferentially increased expression of rare codon reporters (**Supplementary Figure 5C-E**), although siRNA was not as effective as sgRNA. Finally, we note that a side-by-side comparison of cells infected with the vector encoding ZFC3H1 sgRNAs and Cas9 revealed an 80% reduction of ZFC3H1 protein by both immunoblot and proteomics (**Supplementary Figure 15F**), validating the efficiency of these sgRNAs.

Major comment #6: "The validation of the fractionation experiment in fig. 4a should be a supplementary figure and it should also include mainly cytoplasmic RNAs as controls, to ensure that the nuclear fraction is not contaminated with RNAs from the cytoplasmic/extra-nuclear fraction."

As requested**,** we moved these validation data to the supplement and immunoblotted fractions for nuclear, ER, and cytoplasmic protein markers, which confirmed their purity (**Supplementary Figure 7B**).

Major comment #7: "The depletion efficacy shown in fig. S3 is not convincing. In addition, the RNA phenotype is considerably lower, compared to that observed in fig. 3a and fig. 4b for GFPrare in WCE (from average fold change of 4 to 2, respectively). The lack of significance in this panel could at least partly be due to only mild depletion."

As requested**,** we repeated this experiment utilizing a new fractionation protocol (*see* comment #1, Reviewer 2) and analyzed both reporter pairs, which revealed much less variability between replicates and hence a consistent increase in GFP^{rare} levels in the absence of ZFC3H1 (**Figure 3B**).

Major comment #8: "Overall, but especially visible in figures 2, 3, and 5, the observed effects tend to be small and with a lack of consistency between the results obtained with the different reporter constructs (GFPrare and mCherryrare). Might this also be a reflection of poor and variable depletion efficacies?"

As requested, to address whether reporter-specific differences arose due to variation in ZFC3H1 depletion between experiments, cells in which ZFC3H1 was depleted by either sgRNA (**Supplementary Figure 4A**) or siRNA (**Supplementary Figure 5A**) were co-transfected with *both* reporters simultaneously to eliminate variation in ZFC3H1 depletion, after which reporter levels were assessed. We find that all four reporters increased, with a trend towards a greater increase in the rare reporters (**Supplementary Figure 4B, 5B**), as previously observed when cells were independently transfected with one reporter or the other (**Figure 3E,F**)

Major comment #9: "In fig. 6d, the main text and the figure legend are not in agreement, and it is not clear if the plot is considering only mRNAs or the whole transcriptome. Both the plot itself and the legends should be more informative."

As requested, we clarified in the text that this experiment includes all polyadenylated RNA. We also added more information to the plot and the figure legend (**Supplementary Figure 15D**).

Major comment #10: "In figure 7, log2FC > 0 is not necessarily high stringency. Moreover, the proteomics and transcriptomics data are under-analyzed. Among the selected mRNAs targeted by ZFC3H1, are there any common features (e.g., length, intron content, motifs) that could hint to a mechanism for targeting these transcripts for decay/retention?"

As requested, we performed CAI analysis on genes selected using the higher stringency of log2fc > 1 increase at the mRNA or protein level (**Figure 6C**). We also collaborated with RNA bioinformatician Dr. Alain Laederach to perform extensive feature analysis on the RNAseq and proteome datasets. Dr. Laederach assessed whether transcripts that increased in expression (mRNA, protein, or both) upon the loss of ZFC3H1 exhibited any bias in their length, intron content, enrichment or position of GC-rich sequences, or CAI (**Figure 6C, Supplementary Figures 17-22**). These data revealed that in addition to our previous finding that the 97 transcripts exhibiting an increase in both mRNA and protein expression upon the loss of ZFC3H1 had a significantly lower median CAI compared to a published median CAI of the coding genome (**Figure 6B**), the 455 transcripts increased at the protein level upon ZFC3H1 loss were again enriched in rare codons (**Figure 6C**) but also AU-sequences at their 3' end (**Supplementary Figures 19c,20c**), expressed poorly (*see* the next major comment, **Figure 6D**) and longer (**Supplementary Figure 21**) than all genes detected by proteomics.

Major comment #11: "As suggested in the discussion, one possibility is that ZFC3H1 targets mRNAs regardless of their codon enrichment, but as rare-codon enriched mRNAs are normally less expressed, this accumulation would lead to a significant increase in protein expression. This could already be tested with the available data, comparing all ZFC3H1 RNA targets (regardless of the phenotype on the protein levels) and analyzing if there is a correlation between accumulation of these mRNAs, their protein levels and their respective codon content."

As requested, we analyzed the endogenous RNA counts in Ctrl cells for those genes that were more highly expressed at the protein level upon the loss of ZFC3H1 versus all other genes identified by proteomics, which bioinformatically was deemed to be a better comparison. As predicted by this reviewer, the mRNA expression of those genes that were most affected by the loss of ZFC3H1 were indeed significantly more poorly expressed (**Figure 6D**). We also analyzed the endogenous protein (peptide) counts in Ctrl cells for those genes that were more highly expressed at the mRNA level upon the loss of ZFC3H1 versus all other genes identified by proteomics. Again, as predicted by this reviewer, the protein expression of those genes that were most affected by the loss of ZFC3H1 were indeed significantly more poorly expressed (**Supplementary Figure 18**). We thank the reviewer for this suggestion, as it really shed light on a possible mechanism to explain how ZFC3H1 loss preferentially increases the expression of rare codon-enriched genes at the protein level (**Figure 7**).

Major comment #12: "The proteomics analysis might be improved by the labelling of nascent proteins upon ZFC3H1 depletion. This could e.g. be dome using SILAC for quantification of nascent protein levels, potentially allowing a better sensitivity for identifying ZFC3H1 targets.

We agree that SILAC labeling is a powerful tool for accurately quantifying proteins and hence would be useful for future analyses. However, we were particularly interested in how the steady-state proteome changes with ZFC3H1 loss and thus turned to whole cell proteomics. We also note here that the quantitative mass spectrometer used for these studies (Orbital Astrotrap) detected and accurately quantified roughly half (9056 proteins) of the known proteome.

Minor comments:

- "Fig 1e is not informative and could be removed, as ARS2 or CBC and other PAXT components are not present in the screening."
- "Typo in 'extra-nuclear factions' in page 5."
- "In figure 4, would the extra-nuclear fraction simply be represented by the cytoplasm? Is there a special reason for the different denomination?"
- "The writing of the manuscript is often redundant, as exemplified from the text "While PABPN1 and MTREX are found in a number of other complexes, such as a polyadenylation and the NEXT complexes, respectively, ZFC3H1 is the only known subunit specific to the PAXT connection."…"we focused on ZFC3H1, as it is the only protein unique to the PAXT

connection. In more detail, ZFC3H1 is not known to function outside of the PAXT connection."…"we repeated the above analysis, except reduced expression of PABPN1, another subunit of the PAXT connection"

- The figures are often not self-explanatory, and the figure legends are not always well-described, especially for the last figures (fig. 6 and fig. 7).

***As requested*,** we removed figure 1E, provided more information and clarified the figure legends, removed the redundancy in the above passages, described the extra-nuclear fraction as including both the cytoplasm and the endoplasmic reticulum and clarified this nomenclature in the text.

Reviewer #2 (Remarks to the Author)

Comment #1: "The fractionation experiments (Figure 4) are not very clean. In particular, the nuclear fraction seems heavily contaminated with cytosol (called "extra-nuclear" by the authors). The authors should redo this experiment with a much more rigorous protocol. In particular I refer them to the RNA Frac-Seq (see in Materials & Methods of Lee et al., NAR 2020 - PMID: 33091126), which uses 1% Triton X-100 and 0.2% sodium deoxycholate detergents – although the exact percentage of these detergents should be tested and adjusted. Note that the nuclei should be extensively washed or they will contain cytoplasmic contamination. I also advise the authors to keep some of the fractions (pre-TRIZOL treatment) to analyze by immunoblot for nuclear, ER and cytoplasmic proteins."

As requested, we repeated with both the suggested more rigorous protocol and validation of fractionations by immunoblot in cells co-expressing both reporters in the absence and presence of ZFC3H1, but did not observe any overt preference of nuclear export of rare codon reporters with ZFC3H1 loss (**Figure 3B and Supplementary Figure 7**).

Comment #2: "The authors should determine whether ZFC3H1-depletion causes the co-depletion of other PAXT proteins and vice versa. If components tend to be co-depleted (as observed by some groups), this will not change the authors' interpretations, but this information is important."

As requested, we individually depleted ZFC3H1, PABPN1, or MTREX by CRISPR/Cas9 and analyzed the expression level of all three endogenous proteins by immunoblot, which revealed that, with the exception of a small decrease in ZFC3H1 levels upon MTREX depletion (as previously reported), loss of one component did not alter the expression of the remaining two (**Supplementary Figure 12**).

Comment #3: "I may have missed this, but the authors should indicate the CAI values of their various GFP and mCherry reporters (are these the dotted lines in Figure 7C?)"

As requested, we now include this information, which was admittedly hard to locate as it was part of **Supplementary Table 1**, in the main text to make it clear that the dotted lines are the average CAI of both rare and common reporters.

Comment #4: "In Mordstein et al., Cell Syst 2020, the authors report that GC-content in the first half of the mRNA plays a key role in modulating their nuclear export, especially when the mRNA is generated from an intron-poor gene. Indeed, this is in line with the findings of other groups (see Palazzo et al., RNA Biol 2024 for an overview) and in line with the recent report that ZFC3H1 binding/activity is affected by GC-content near the 5' end of the mRNA (Fan et al., Mol Cell 2024). Note that rare-codons are typically GC-poor. Given these prior findings, are ZFC3H1-sensitive mRNAs derived from intron-poor genes? Are they GC-poor? Is the CAI

(or GC-content) at the 5' end of the ORF more predictive of ZFC3H1-sensitivity? These questions should be easily answered given the sequencing data."

As requested, We collaborated with RNA bioinformatician Dr. Alain Laederach to perform extensive feature analysis on the RNAseq and proteome datasets. Dr. Laederach assessed whether transcripts that increased in expression (mRNA, protein, or both) upon the loss of ZFC3H1 exhibited any bias in their length, intron content, enrichment or position of GC-rich sequences, or CAI (**Figure 6C, Supplementary Figures 17-22**). These data revealed that in addition to our previous finding that the 97 transcripts exhibiting an increase in both mRNA and protein expression upon the loss of ZFC3H1 had a significantly lower median CAI compared to a published median CAI of the coding genome (**Figure 6B**), the 455 transcripts increased at the protein level upon ZFC3H1 loss were again enriched in rare codons (**Figure 6C**) but also AU-sequences at their 3' end (**Supplementary Figures 19c,20c**), expressed poorly (**Figure 6D**), longer (**Supplementary Figure 21**) than all genes detected by proteomics. Additionally, analysis of the 486 transcripts that were significantly increased upon ZFC3H1 loss were significantly more GC-rich (albeit not at the 5' end, **Supplementary Figure 19**), supporting Fan et al and had shorter total intron length(**Supplementary Figure 22**) than all genes detected by proteomics. These additional analyses really shed light on a possible mechanism to explain how ZFC3H1 loss preferentially increases the expression of rare codon-enriched genes at the protein level (**Figure 7**).

Reviewer #3 (Remarks to the Author)

Major Comment #1: "A general problem in figures using the reporter system is that the expression of ZFC3H1 is quite variable between duplicate samples, and also between the different knock-down, over-expression and rescue conditions, and also between the converse configurations of the reporters (2a vs 2c for example). To point out an example, in Fig 2a, the expression of ZFC3H1 doesn't strictly follow the experimental condition. There is less ZFC3H1 in rep 2 of the -/- control, only rep 2 of -/+ seems to be in fact over-expressed, and neither rescue rep is really rescued, and so on. This in seen in Fig 2a, Fig 2c and supp figs. This is not necessarily a problem as it's extremely difficult to achieve perfect expression profiles in such experiments, the problem is that the expression of the rare codon doesn't always follow the expression of ZFC3H1."

We agree, and hence we tested and found that sgRNA-mediated inactivation of ZFC3H1 resulted in an increase in rare over common reporters, as determined by simultaneously testing both immunoblot at progressively lower amount of lysates (**Supplementary Figure 3A**) *and* by FACS analysis (**Supplementary Figure 3B**). We also tested and found that reducing ZFC3H1 expression by siRNA (as confirmed by immunoblot, **Supplementary Figure 5A**) preferentially increased expression of rare codon reporters (**Supplementary Figure 5C-E**), although siRNA was not as effective as sgRNA.

Major Comment #2: "The authors also conclude that the effect is specific for the rare codon reporter. However, there is a significant effect on the common codon reporter as well, in Fig 2a. How well is the common reporter (GFP or Cherry) expressed relative to the rare codon reporter? If it is much more highly expressed, this could make it difficult to quantitatively compare the effect of the knock-downs on expression of the common codon reporter. Since the increase in rare vs common reporter expression following loss of ZFC3H1 leads to the suggestion that the effect is specific for rare codon reporters, it is important to resolve this issue."

As requested, we tested and found that the common codon reporter (mCherry^{com}) is indeed expressed more highly than the rare codon reporter (GFP^{rare}), as measured by more quantitative flow cytometry (**Supplementary Figure 8B**). In fact, this suggested to us that the preferentially effect of ZFC3H1 loss on the rare codon reporters may be due, in part, to a larger dynamic range of expression possible with rare codon reporters compared to common, or to put it another way, the common codon reporters are already expressed so high that a small increase in their mRNA does not generate more protein. Indeed, the same explaination was posited to explain why disrupting one allele of the *Drosophila* gene *Rps21* preferntially increase protein expression of an inducible Ras85 transgene when encoded by rare compared to common codons. One prediction of this model is that increasing the amount of rare codon reporter mRNA should blunt the ability of SFC3H1 loss to increase protein expression. Indeed, we tested and found this to be the case (**Figure 4A,B**), namely increasing the amount of either reporter led to a smaller and smaller difference in protein expression between the rare and common reporter transgenes. Consistent with this, we

analyzed the endogenous RNA counts in Ctrl cells for those genes that were more highly expressed at the protein level upon the loss of ZFC3H1 versus all other genes identified by proteomics. We found that the mRNA expression of those genes that were most affected by the loss of ZFC3H1 were indeed significantly more poorly expressed (**Figure 6D**). Thus, we have expanded our discussion of possible mechanisms to account for the rare codon-specific effect of ZFC3H1 loss and included a model thereof (**Figure 7**).

Major Comment #3: "For the experiments using Western blot analysis, on which the major conclusions of the manuscript are based, it would be preferable to use a more quantitative approach. In addition, at least some samples could be tested in dilution series to validate the quantification given by band density analyzing software. This may seem old fashioned, but it is important to be sure that the differences detected following quantification are real and not due to antibody saturation of other known western blotting artefacts."

As requested, we tested and found that sgRNA-mediated inactivation of ZFC3H1 resulted in an increase in rare over common reporters, as determined by simultaneously testing both immunoblot at progressively lower amount of lysates (**Supplementary Figure 3A**) *and* by FACS analysis (**Supplementary Figure 3B**). This is not old fashioned, just good molecular biology.

Major Comment #4: "Next, the authors analyzed RNA levels corresponding the reporter plasmids. RNA levels largely mirrored those of protein levels, or rather, protein levels mirrored those of RNA. As above, it is important to know the relative expression level of the common codon reporter compared to the rare codon reporter. Absolute levels of RNAs could be shown, on separate graphs if the expression levels are not equal."

As requested, we measured the absolute levels of the RNA of all four reporters when co-expressed in the same cells, and find that the common reporter transgenes are expressed some 15-fold higher than their rare counterparts (**Supplementary Figure 8A**). Again, we suggest that this is further evidence that changing the rare to common codons of the identical gene (*i.e.* GFP^{rare} to GFP^{com} or $mCherry^{rare}$ to $mCherry^{com}$) results in higher transcript expression, and consequently a small increase in mRNA upon ZFC3H1 loss does not make much of a difference at the protein level. This suggests that rare codon-enriched genes expressed at low levels should be the most sensitive to the loss ofZFC3H1, and that is exactly what we see (**Figure 4**).

Major Comment #5: "In fig 4b, siZFC3H1 is not significantly different to siCtrl whereas in Fig 3 a and b, it is. How reproducible is the result?"

As requested**,** we repeated this experiment utilizing a new fractionation protocol (*see* comment #1, Reviewer 2) and analyzed both reporter pairs simultaneously, which revealed much less variability between replicates and hence a consistent increase in GFP^{rare} levels in

the absence of ZFC3H1, but did not observe any overt preference of nuclear export of rare codon reporters with ZFC3H1 loss (**Figure 3B and Supplementary Figure 7**).

Major Comment #6: "In Fig 5, it is difficult to compare the 'significance' of the western blot results with those of RNA quantification, since the RNA experiments appear to have been calculated using only 2 replicates, whereas protein results use 3 replicates."

As requested, we added another replicate for each experiment (**Figure 5**).

Major Comment #7: "In Fig 7, since the authors have both RNA-seq and proteomics data, it would have been interesting to identify genes that are increased at the protein level but whose transcripts are not affected, which might provide candidates to further address a potential role in RNA export or translation. Concerning the statistical analysis of this data set, given the large difference in N for each category (97 for ZFC3H1 targets versus > 10,000 transcripts for the human transcriptome presumably), the analysis should be performed using a bootstrapping method, rather than simply calculating the median CAIs, if I understand the statistical method correctly."

As requested, we provided more analysis and information, including pathway analysis and CAI values, for those genes that are affected at the protein level, the mRNA level, or both. We also have performed all new analyses with a bootstrapping method (*see* **Figure 6C,D** and **Supplementary Figures 17-23** for new CAI, feature analysis, and pathway analysis for multiple new groupings, including all proteins increased with ZFC3H1 loss). We found that proteins significantly increased upon ZFC3H1 loss were enriched in rare codons, AU-rich (specifically at the 3- end, differing from some previous studies of transcripts), and poorly expressed, compared to all genes detected by proteomics. We also note that these proteins are involved in cell cycle, metabolism, and RNA degradation pathways, adding new understanding in how ZFC3H1 loss transforms the cellular environment.

Major Comment #8: "To strengthen the conclusions of the study, candidate transcripts (3 to 5) identified in Fig 7 should be validated using the cell lines used for the reporter plasmids."

As requested, we validated that three candidate genes (*PIGW, TSPYL4,* and *TIGD1*) from the 97 identified to be increased at both the mRNA and protein level upon the loss of ZFC3H1, were similarly expressed at higher levels upon the loss of ZFC3H1 as measured by qRT-PCR. Moreover, these cells were transfected with the GFP^{rare}/$mCherry^{com}$ dual reporter to confirm that both transgenes also were expressed at higher levels upon the loss of ZFC3H1 (**Supplementary Figure 16**).

Minor points:
Line 96 and 97, should be ‘favor’, not ‘favored’.
Line 144: ‘independently twice’ means ‘in duplicate’ ?
Line 265, 273 : most readers know what ‘both’ means, no need to italicize and underline it.
Line 280: ‘to put this into context’ could be removed. Instead, it would be more informative to clarify ‘significantly’ by including the p-val obtained.
Line 297: should be ‘highly’

As requested, we have made the above corrections to the written text.

Response to Reviewer #1

<u>Comment 1a:</u> *"Given that both ZFC3H1 and PABPN1 were shown to influence nuclear retention of pA+ RNAs, the authors could use their fractionation data to quantify whether more common/rare-codon reporter transcripts would be preferably located to the nucleus or cytoplasm in normal condition as compared to the ZFC3H1-perturbed condition. The data provided in Figure 3b does not readily estimate if RNA leakage from the nucleus has a significant effect."*

<u>Reply:</u> We thank the reviewer for this suggestion, as such a mechanism lines up with the function of ZFC3H1, and hence we devoted a fair amount of time and effort to this experiment. ***As requested,*** we repeated our previous fractionation experiment (with additional controls requested by reviewer 2) and determined the percent that each reporter transcript was detected in the extranuclear fraction. However, we failed to detect an appreciable change in the extranuclear levels upon the loss of ZFC3H1 (Figure 4B). Instead, it appears that the loss of ZFC3H1 leads to a small, but preferential increase in the level of the rare codon reporter transcripts (Figure 4C).

<u>Comment 2:</u> *"In addition, the PABPN1 RIP-qPCR results of Figure 5g might be explained by nuclear retention of rare-codon mRNAs given that PABPN1 is localized to the nucleus?"*

<u>Reply:</u> See the above, namely we do not find evidence that either codon bias or the loss of ZFC3H1 alters the amount of extranuclear transcripts.

<u>Comment 3:</u> *"There is also the possibility that an indirect effect of ZFC3H1 loss would lead to preferential translation of rare-codon proteins. In this vein, it has been shown that leakage of spurious transcripts into the cytoplasm upon ZFC3H1 loss affects global translation levels. The authors could explore (or at least discuss) these possibilities."*

<u>Reply:</u> Thank you for alerting us to this potential mechanism. ***As requested***, we investigated whether ZFC3H1 loss affected global translation levels via the puromycin sunset assay, which utilizes puromycin incorporation in actively translating proteins as a proxy for translation rates. However, depletion of ZFC3H1 (as confirmed by immunoblot) in triplicate or quadruplicate by siRNA or sgRNA, respectively, did not significantly change global puromycin incorporation (Supplementary Figure 4).

<u>Minor point 1:</u> *"The manuscript will benefit from standardisation of data presentation, like using similar ways to show statistical significance (asterisks) across the manuscript."*

<u>Reply:</u> ***As requested,*** we have standardized data presentation.

Response to Reviewer #2

<u>Comment 1:</u> *"The authors did their best to answer all of the comments, however my enthusiasm for the work has dampened substantially. Notably, in Figure 4 and Supplemental Figure 10, the authors demonstrate that their assay is dependent on the level of mRNA expression. This is especially striking in Supplemental Figure 9b where data from "low" mCherry-com looks essentially the same as GFP-rare. At the same time, ZFC3H1 has almost no effect for the "High Reporter" experiments (Figure 4a, Supplemental Figure 10a). These findings calls into question the interpretation of the screen (Figure 1) and of many other experiments. Could it simply be that mRNAs expressed at lower levels are more sensitive to ZFC3H1? If so this effect could have nothing to do with the presence of rare codons."*

<u>Reply:</u> ***We note*** that the genetic screen was specifically designed to identify genes affecting protein levels in a rare codon-specific fashion, with the top candidate being ZFC3H1. Not only were *ZFC3H1* sgRNAs identified from the initial screen, but also the secondary screens, and throughout the manuscript, the loss of ZFC3H1 consistently increased rare over common reporter protein expression, which speaks to the validity of the screen. As we marched through possible mechanisms to account for this reproducible effect, including those suggested by reviewers, evidence pointed towards an accumulation of small effects at multiple levels leading to a preferential increase in the rare codon reporters upon the loss of ZFC3H1. Namely, admittedly small but preferential increases in rare codon-reporter mRNA and stability (Figs 2,3) and finally protein. Interestingly, the ability of ZFC3H1 to preferentially increases rare codon reporter protein levels was blunted by increasing the amount of rare codon mRNA, suggesting an intimate connection to low transcript levels. This played out beautifully at the whole cell level. Namely, whole cell transcriptomic and proteomic analysis revealed that the loss of ZFC3H1 reprogramed the proteome *<u>specifically towards rare codon-enriched transcripts that were expressed at low levels</u>* (Fig 7). In this way, rare codons could be seen as a rather unique feature hardwired into the mRNA that allows protein levels to be increased via small increases in poorly expressed transcripts.

<u>Comment 2:</u> *"There are other inconsistencies that have popped up in the revision. Despite the fact that all reporters are affected (Figure 2e-f), when mRNA turnover kinetics are measured, only minor changes are noted (Figure 2a). I suspect that any potential change in mRNA turnover occurs only at early timepoints in the nucleus (where the PAXT complex resides), whereas a sizeable fraction of the mRNA analyzed in Figure 2a is cytoplasmic (i.e. it has already escaped potential decay by the PAXT complex). Perhaps a pulse-chase experiment with EU would give more reliable results.*"

<u>Reply:</u> ***As requested,*** we note that Meola et al. 2016 (ref 27) utilized actinomycin D at similar timepoints as our experiments to originally demonstrate stabilization of PAXT-target transcripts. We thus employed their assay to measure the decay kinetics of the reporter transcripts. We now demonstrate in two independent experiments performed in triplicate that loss of ZFC3H1 (confirmed by immunoblot) leads to a small increase in the rare codon reporter transcript stability compared to their common counterparts. As a control, we confirm the previous stabilization of the PAXT-target transcript *SNHG19*

(Supplementary Figure 8). Given this, we agree with this reviewer that the effect of ZFC3H1 loss may be an early event and now note this possibility in the discussion.

Comment 3: *"I am also not convinced about the fractionation data – for example, the mCherry-rare reporter, which shows a substantial increase in protein (Figure 2c) and a 2.5 fold increase in mRNA levels (Figure 2f), shows no increase in cytoplasmic levels ("EN" Figure 3b). I suspect that there is something off in the fractionation results. During fractionation, RNA is extremely susceptible to non-specific decay, and levels of 28S and 18S should be monitored to ensure that the RNA survived the procedure. In addition, from the written protocol, it is not clear what standard was used for the RT-qPCR to determine these RNA levels in the various fractions. The authors may want to monitor the nuclear/cytoplasmic ratios using Fluorescence In Situ Hybridization (FISH)."*

Reply: ***As requested*,** we repeated the fractionation experiment in triplicate and demonstrated that *28S* and *18S* RNA were intact in all fractions, as detected by the bleach agarose gel method (Supplementary Figure 9). In this repeat experiment we observed a trend or significant increase GFP^{rare} and $mCherry^{rare}$ mRNA in all fractions, with again these differences be blunted in the common counterparts (Figure 4). Last, we now note in the materials and methods that *GAPDH* was used to normalize mRNA levels, as previously reported by Silla et al. 2018 (ref 28 in manuscript) in their fractionation assay to study the localization of PAXT-target transcripts after PAXT depletion.

Minor comment 1: *"Supplemental Figure 7. The Lamin B1 protein is hard to evaluate in the blot from the Ctrl sgRNA samples - there is no reliable band present."*

Reply: ***As requested,*** we repeated this experiment and now detect Lamin B1 in all nuclear fraction samples (supplementary figure 9).

Minor comment 2: *"Supplemental Figure 8. I'm not sure how GFP and mCherry mRNAs can be compared to each other and normalized in the manner presented. These are presumably amplified using different primers, which will likely have different amplification rates."*

Reply: This comparison was specifically requested by a reviewer but now note the caveat of comparing mRNA levels in this fashion in the text.

Minor comment 3: *"Page 7. "We then censored for transcripts" I would not use "censored" in this sentence – perhaps "conducted a census?"*

As requested, we changed the text accordingly**.**

Minor comment 4: *"Figure 6b. FC > 0 should be og2FC > 0."*

As requested, we corrected this labeling error, thank you for pointing this out**.**

<u>Minor comment 5:</u> *"Figure 6d and Supplemental Figures 18 and 22. Something is wrong with the violin plots."*

As requested, we corrected this error in data scaling, thank you for pointing this out. While the data are correct, the problem arose in that we used a kernel density estimation (KDE) model based linear-scale data to plot data on a log scale, which led to some violins and whiskers artifactually extending toward zero. We have thus updated the plots (Figure 7D, Supplementary Figures 20, 23, 24) using an appropriate KDE treatment for log-scaled data, which resolves the visual inconsistency while preserving the correct distributional structure. We have additionally corrected the description of the statistical test used for these violin-plot comparisons in the figure legends and Methods. It was previously described as a "leave 10% out bootstrap," whereas the procedure actually used is a size-matched random subsampling test: for each group of n genes, n values are drawn at random, without replacement, from all genes detected by proteomics to build a null distribution of means, against which the observed group mean is compared. The underlying values and the reported p-values are unchanged.

<u>Minor comment 6:</u> *" Figure 7. The authors do not provide evidence that PABPN1 recruits ZFC3H1 to its purported substrates."*

As requested, we removed this figure.

<u>Minor comment 7:</u> " Supplemental Figure 12. I'm surprized how much protein remains in the CRISPR'd cells."

As requested, we repeated this experiment with two additional biological replicates (Supplementary Figure 14) and demonstrate more robust depletion of PABPN1, ZFC3H1, and MTREX protein by the corresponding sgRNA.

Response to Reviewer #3

<u>Comment 1:</u> *"The authors have adequately addressed the concerns raised during review."*

Reply: ***We thank this reviewer*** for their time and effort in reviewing this manuscript; their comments and suggestions have improved the original study.

Response to Reviewers

Reviewer #1

Major point: "*The authors have addressed my remaining comments from the second round of review. However, they did not explain in the rebuttal what specific changes were made in response to my concerns regarding the results in Figures 2e–f and 3a.*"

Response: In regard to the referenced comment that "*However, I remain unconvinced by the variable effects on rare-codon RNA levels (Figure 2e,f) and degradation rates (Figure 3a), upon ZFC3H1 and PABPN1 loss, as compared to that of the rare-codon protein reporters. It seems to me that alternative mechanisms, such as differences in nuclear export rates or translation efficiencies, might better explain the observed phenotypes. Given that both ZFC3H1 and PABPN1 were shown to influence nuclear retention of pA+ RNAs, the authors could use their fractionation data to quantify whether more common/rare-codon reporter transcripts would be preferably located to the nucleus or cytoplasm in normal condition as compared to the ZFC3H1-perturbed condition. The data provided in Figure 3b does not readily estimate if RNA leakage from the nucleus has a significant effect.*" we did the following

- Repeated our previous fractionation experiment to determine the percent that each reporter transcript is detected in the extranuclear fraction. However, we failed to detect an appreciable change in the extranuclear levels upon the loss of ZFC3H1 (Figure 4B). Instead, it appears that the loss of ZFC3H1 leads to a small, but preferential increase in the level of the rare codon reporter transcripts (Figure 4C).
- Investigated whether ZFC3H1 loss affected global translation levels via the puromycin sunset assay. However, depletion of ZFC3H1 in triplicate or quadruplicate by siRNA or sgRNA, respectively, did not significantly change global puromycin incorporation (Supplementary Figure 4).
- Beyond performing these two additional experiments to "*quantify whether more common/rare-codon reporter transcripts would be preferably located to the nucleus or cytoplasm in normal condition as compared to the ZFC3H1-perturbed condition*" and determine if "*RNA leakage from the nucleus has a significant effect*" we also revised the summary to include alternative mechanisms and caveats

Minor point: "*The manuscript would still benefit from a more consistent presentation of statistical significance. For example, the formatting of the significance asterisks differs between Figures 2–6 and Figure 7.*"

Response: ***Thank you*** for pointing out this discrepancy. The formatting of the asterisks is now consistent across all figures.

<u>Reviewer #2:</u>

Minor point: "*The concerns were mostly addressed. For clarity the authors should just make the box plot in Supplemental Figure 21a ("all proteins") more visible (it is currently hidden behind the data points). They should also change the word "censored", which appears 3 times in the current text. "Censored" means to remove or alter communications by an authority that wants to limit the exchange of ideas, and not to conduct a census. The authors should use more appropriate wording, such as "conducted a census" or "analyzed".*"

Response: ***Thank you*** for these helpful suggestions. As requested, *1*) the box plot in Supplemental Figure 21a was revised to be more visible, and 2) we removed the word censored in the text and replaced it with more clear wording.
