# Supplementary material for: The PAXT connection is a modifier of rare codon-enriched protein expression
Source: Nat Commun. 2026 Sep 17;17:10928. doi: 10.1038/s41467-026-77792-7 (PMC13680609; doi:10.1038/s41467-026-77792-7)
Supplement: Supplementary file 3 [file 41467_2026_77792_MOESM1_ESM.pdf]

Supplementary Information for
The PAXT connection is a modifier of rare codon-enriched protein expression

Taylor Niehoff,[1] Jackson Peterson,[1] Siqi Li,[1] Sijin Chen,[2] Christopher V Nicchitta,[3] Alain Laederach,[2] and Christopher M. Counter[1]✻

[1]Department of Pharmacology & Cancer Biology, Duke University Medical Center, Durham, NC, USA
[2]Department of Biology, University of North Carolina at Chapel Hill, Chapel Hill, NC, USA
[3]Department of Cell Biology, Duke University Medical Center, Durham, NC, USA
✻Correspondence: count004@mc.duke.edu

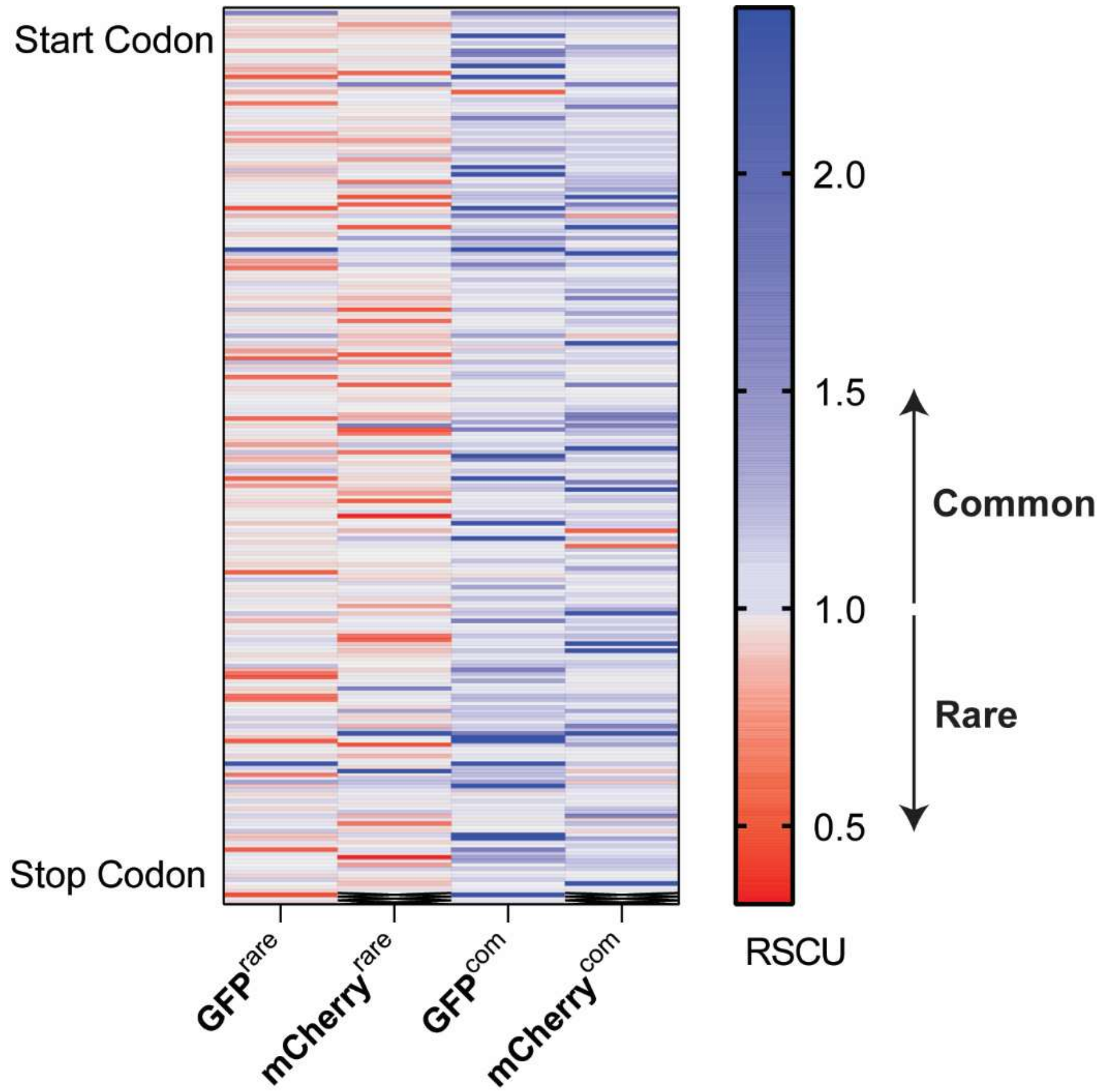

Supplementary Figure 1. Comparison of the codon usage between the rare and common codon-enriched reporters. Heat map of _R_elative _S_ynonymous _C_odon _U_sage (RSCU) across each of the indicated transcripts. 1 (white), >1 (blue), and <1 (red) refers to no bias, a positive bias, or a negative bias, respectively, between the actual versus expected frequency in the coding human genome of the indicated codon. Note that mCherry is three amino acids shorter than GFP, and hence three black bars were inserted at the 3' termini of the mCherry alignment. Source data are provided as a **Source Data** file.

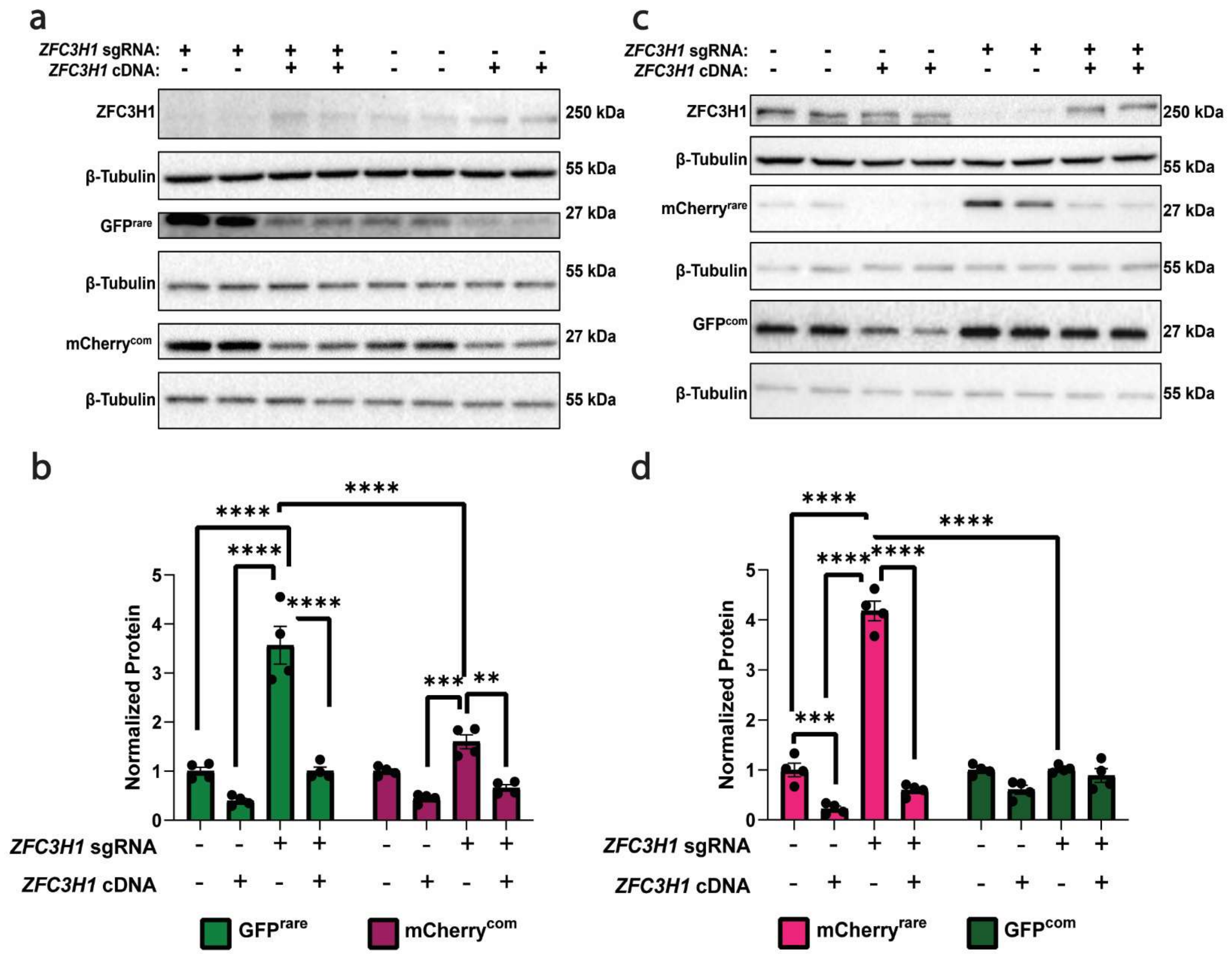

Supplementary Figure 2. Reporter protein levels upon ZFC3H1 depletion and rescue thereof. (**a,c**) Additional replicate (2 of 4 biological replicates) immunoblots for the indicated proteins from 293T cells transduced with vectors encoding *Ctrl* or *ZFC3H1* sgRNA, no transgene or *ZFC3H1* cDNA, and the (**a**) $GFP^{rare}/mCherry^{com}$ or (**c**) $mCherry^{rare}/GFP^{com}$ dual reporter. (**b,d**) Mean ± SEM of protein levels from 293T cells transduced with vectors encoding *Ctrl* or *ZFC3H1* sgRNA, no transgene or *ZFC3H1* cDNA, and (**b**) $GFP^{rare}/mCherry^{com}$ or (**d**) $mCherry^{rare}/GFP^{com}$ dual reporter, as assessed by immunoblot and normalized to β-tubulin and *Ctrl* sgRNA cells, derived from n=4 biological replicate experiments (black circles). Three-way ANOVA and Tukey's post hoc tests performed for panels **b** (F ratio=44.3804, $p<0.0001$, n=4 biological replicates) and **d** (F ratio=131.3949, $p<0.0001$, n=4 biological replicates). **$p<0.01$, ***$p<0.001$, and ****$p<0.0001$. Different exposure times were required for each antibody (*see* uncropped images in **Supplementary Data 5**). Source data are provided as a **Source Data** file.

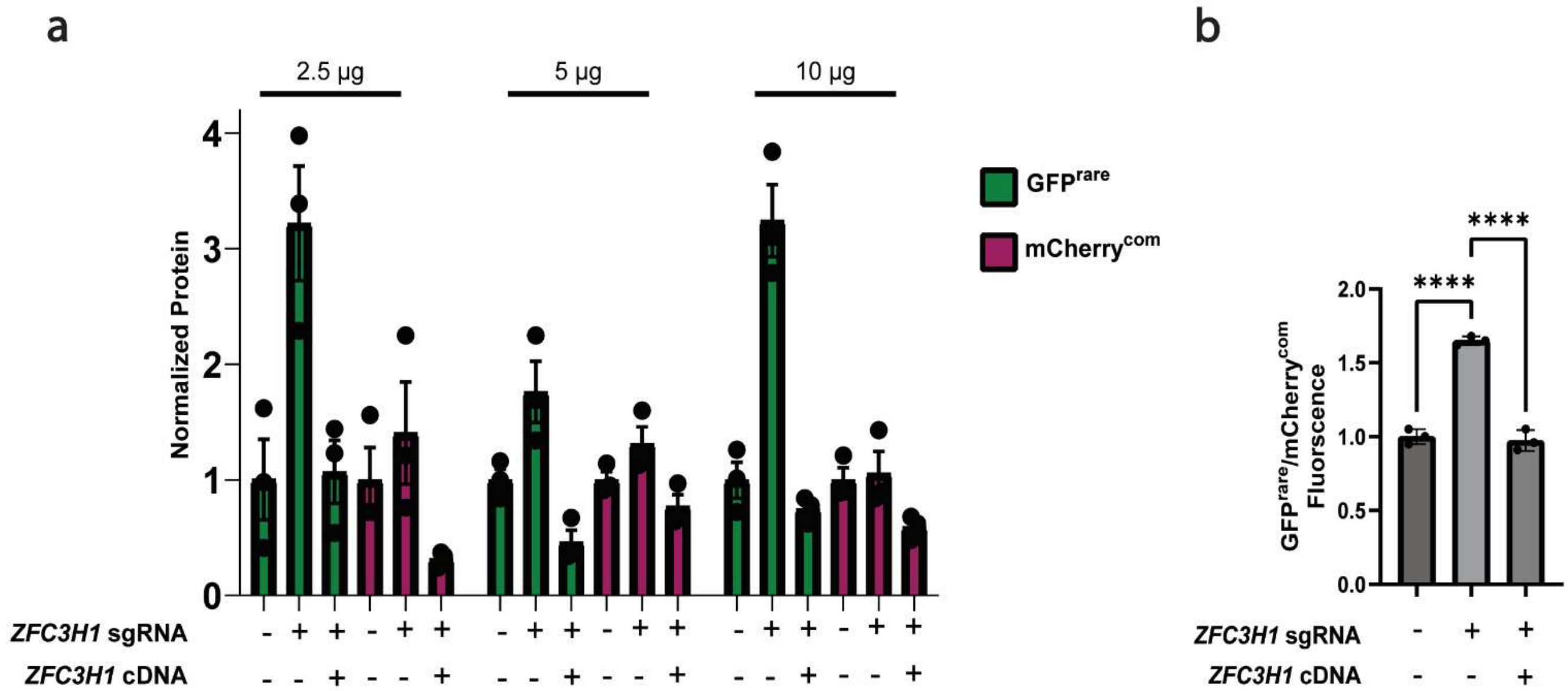

Supplementary Figure 3. Validation of reporter quantification. (**a**) The derived mean ± SEM of the normalized protein expression levels of the indicated proteins, as detected by immunoblot of 293T cells infected with a lentivirus encoding *Ctrl* or *ZFC3H1* sgRNA followed by co-transfection with a vector encoding no transgene or *ZFC3H1* cDNA and the GFP^{rare}/mCherry^{com} dual reporter. Different total amounts of protein (2.5, 5, or 10 µg) were loaded for detection for all samples. Immunoblot levels normalized to β-tubulin and *Ctrl* sgRNA cells. (**b**) The derived mean ± SEM of the GFP^{rare}/mCherry^{com} fluorescence ratio, normalized to *Ctrl* cells, as detected by flow cytometry, from the same cell populations as (**a**). Quantifications derived from n=3 biological replicate experiments (black circles). One-way ANOVA and Tukey's post hoc tests performed for panel **b** (F ratio=156.7154, p<0.0001, n=3 biological replicates). ****p<0.0001. Corresponding, uncropped immunoblots are shown in **Supplementary Data 5**. Source data are provided as a **Source Data** file.

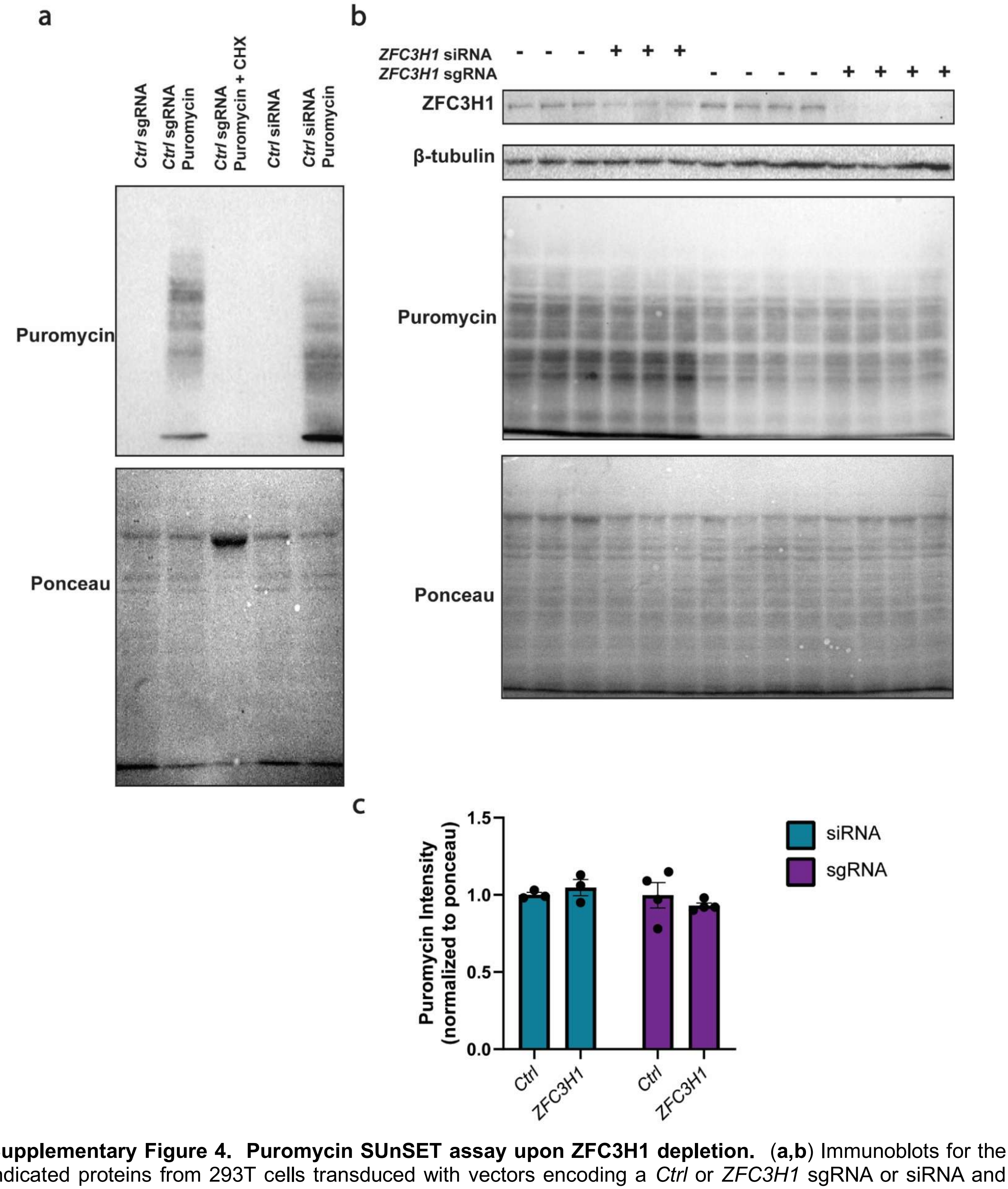

Supplementary Figure 4. Puromycin SUnSET assay upon ZFC3H1 depletion. (**a**,**b**) Immunoblots for the indicated proteins from 293T cells transduced with vectors encoding a *Ctrl* or *ZFC3H1* sgRNA or siRNA and treated with either (**a**) 10 μg/mL puromycin for 10 minutes, 10 μg/mL cycloheximide for 10 minutes and then 10 μg/mL puromycin for 10 minutes, or neither or (**b**) 10 μg/mL puromycin for 10 minutes. (**c**) the derived mean ± SEM of puromycin intensity in (**b**) (normalized to ponceau staining and *Ctrl* sgRNA or siRNA cells), derived from *n*=3 (siRNA) to 4 (sgRNA) biological replicate experiments (black circles). Two-tail Welch's t-test performed for both siRNA and sgRNA for panel **c** (non-significant for both). Different exposure times were required for each antibody (*see* uncropped images in **Supplementary Data 5**). Source data are provided as a **Source Data** file.

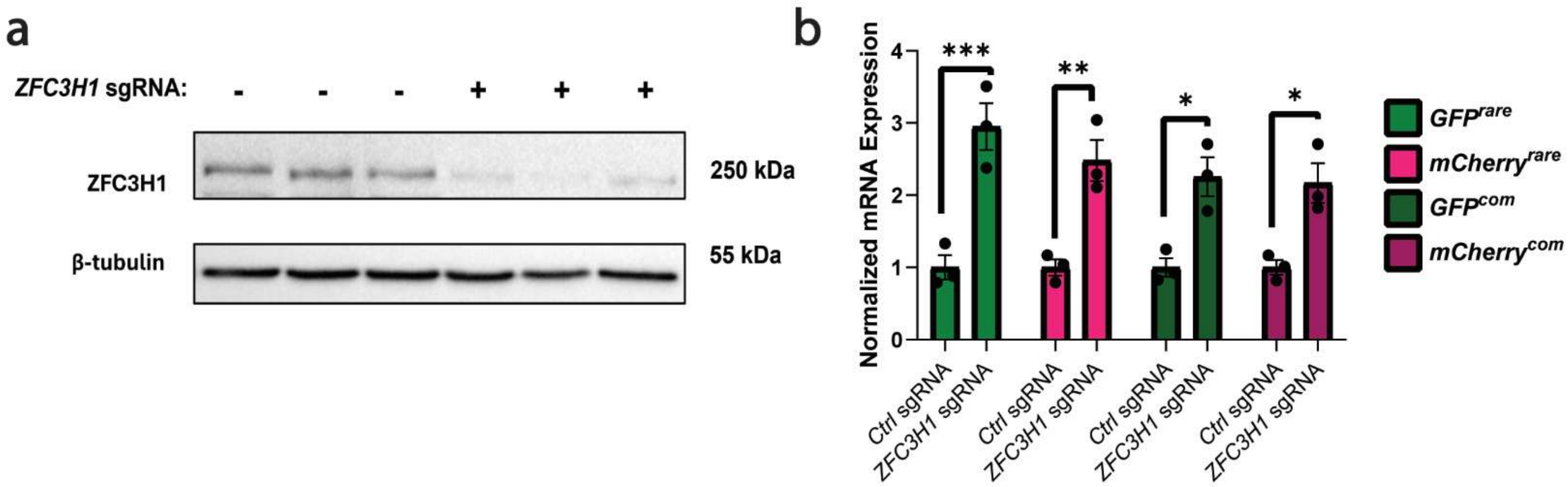

Supplementary Figure 5. Co-transfection and quantification of all reporter mRNA levels in ZFC3H1 depleted cells. (**a**) Immunoblots of the indicated proteins from 293T cells transduced with vectors encoding either a *Ctrl* or *ZFC3H1* sgRNA and both GFP^{rare}/mCherry^{com} and mCherry^{rare}/GFP^{com} dual reporters. Different exposure times were required for each antibody (*see* uncropped images in **Supplementary Data 5**). (**b**) Mean ± SEM of mRNA levels, as assessed by RT-qPCR and normalized to *18S* rRNA and *Ctrl* sgRNA cells, of the indicated reporters from n=3 biological replicate experiments (black circles). Two-way ANOVA analysis with Tukey's post hoc test for panel **b** (F ratio=13.2411, $p<0.0001$, n=3 biological replicates). *$p<0.05$, **$p<0.01$, and ***$p<0.001$. Source data are provided as a **Source Data** file.

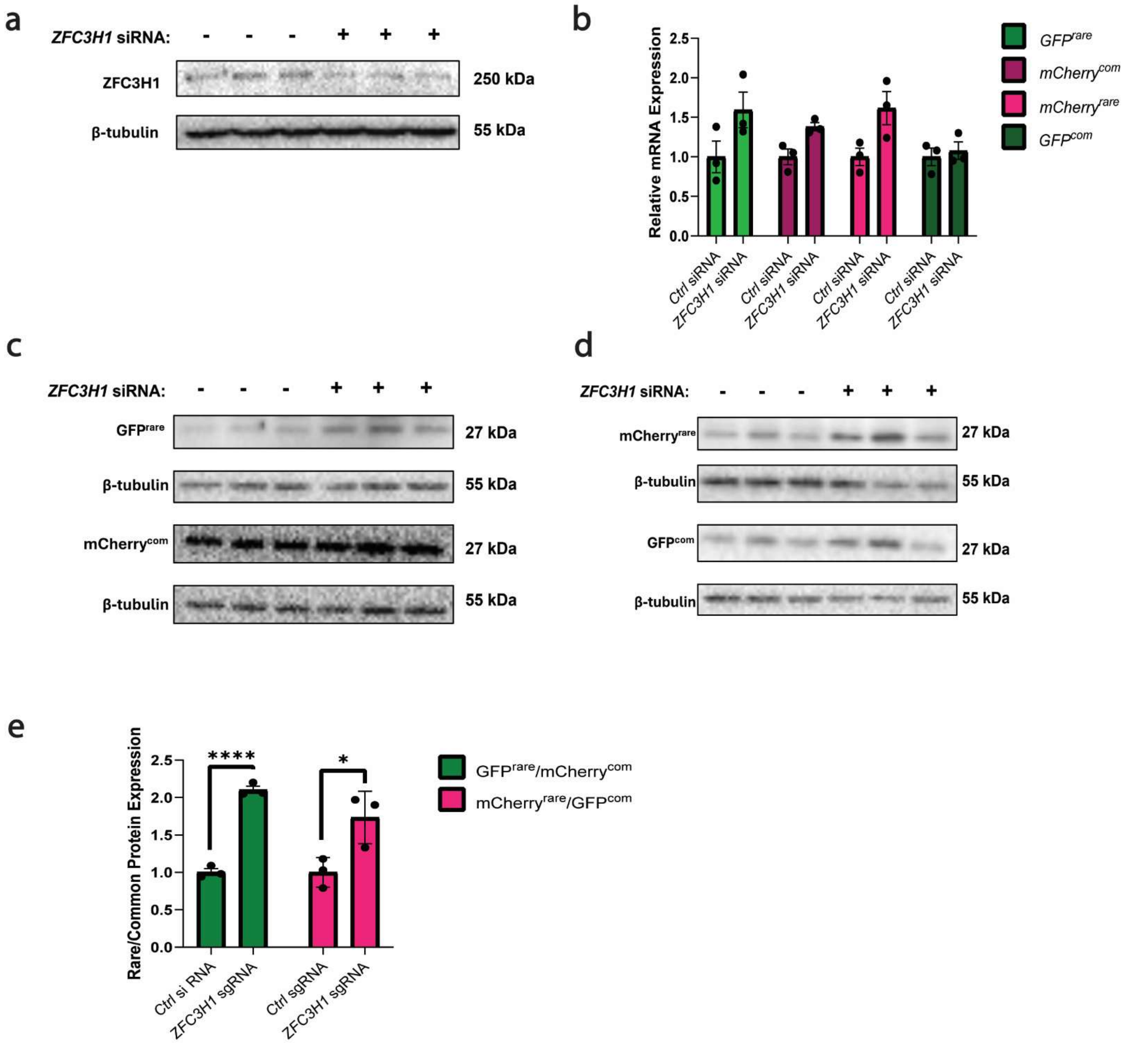

Supplementary Figure 6. The effect of ZFC3H1 loss by RNAi on reporter protein and mRNA levels. (**a,c-d**) Immunoblots of the indicated proteins (*right*: predicted molecular weight of the indicated protein), (**b**) mean ± SEM mRNA levels from 293T cells transfected with *Ctrl* or *ZFC3H1* siRNA and both GFP^{rare}/mCherry^{com} and mCherry^{rare}/GFP^{com} dual reporters, as assessed by RT-qPCR and normalized to *18S* rRNA and *Ctrl* sgRNA cells, derived from *n*=3 biological replicate experiments (black circles), and (**e**) the derived mean ± SEM ratio of (**c,e**) GFP^{rare} versus mCherry^{com} or (**d,e**) mCherry^{rare} versus GFP^{com} protein in 293T cells transfected with *Ctrl* or *ZFC3H1* siRNA and the (**c**) GFP^{rare}/mCherry^{com} or (**d**) mCherry^{rare}/GFP^{com} dual reporter, derived from *n*=3 biological replicate experiments (black circles). Two-way ANOVA and Tukey's post hoc performed for panel **b** (F ratio=3.3302, *p*=0.0219, *n*=3 biological replicates). Welch's t-test performed for each reporter for panel **e**. **p*<0.05 and *****p*<0.0001. Different exposure times were required for each antibody (*see* uncropped images in **Supplementary Data 5**). Source data are provided as a **Source Data** file.

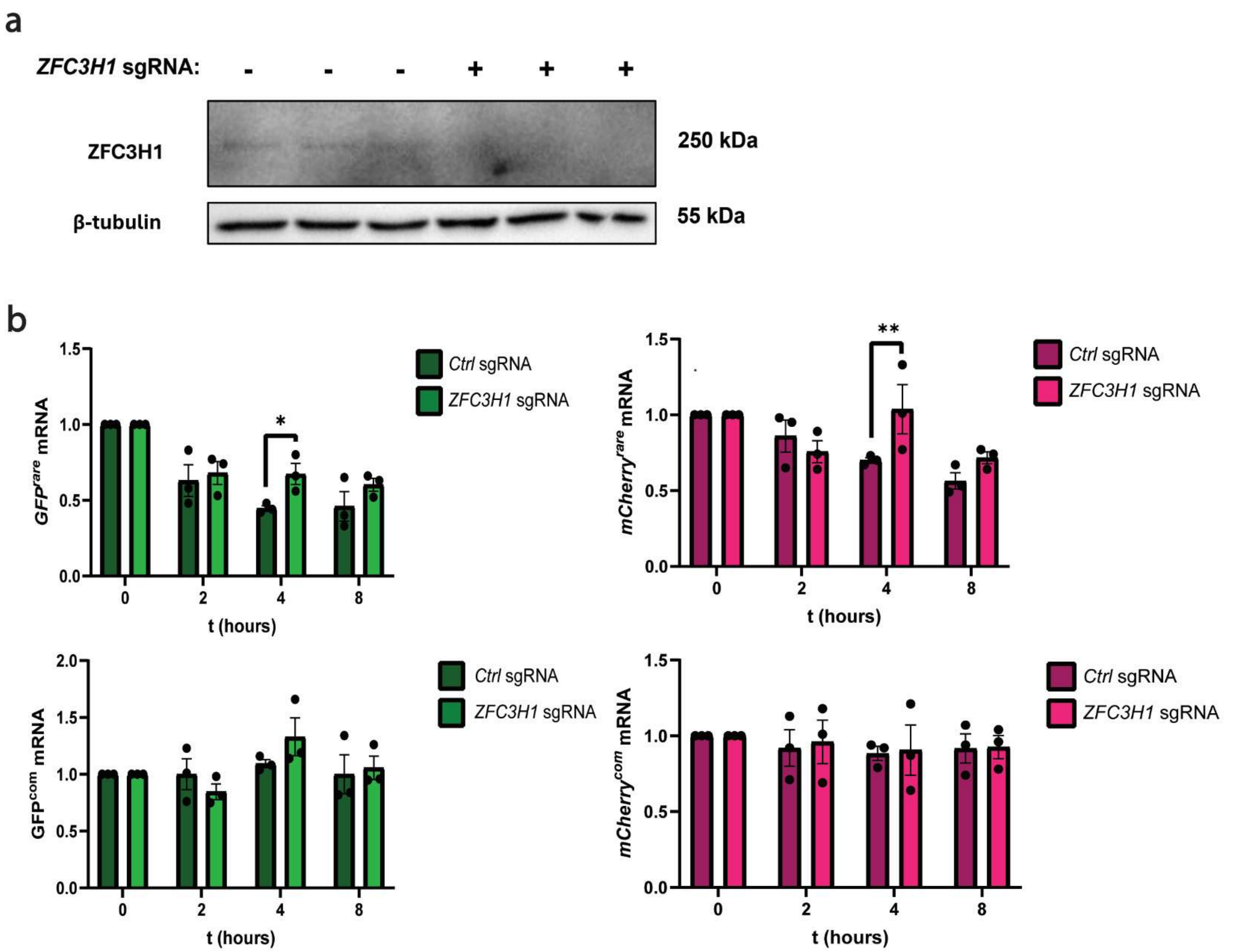

Supplementary Figure 7. Validation of ZFC3H1 depletion and replicates for RNA stability measurements after Actinomycin D treatment. (**a**) Immunoblots of the indicated proteins from 293T cells transduced with vectors encoding either a *Ctrl* or *ZFC3H1* sgRNA. (**b**) Mean ± SEM of the indicated reporter mRNA, as assessed by RT-qPCR, in *Ctrl* and *ZFC3H1* sgRNA cells co-transfected with both GFP^{rare}/mCherry^{com} and mCherry^{rare}/GFP^{com} dual reporters after treatment with Actinomycin D for the indicated amount of time (t) normalized to *18S* rRNA and t=0 values from *n*=3 biological replicate experiments (black circles). Two-way ANOVA for each reporter with sliced two-tailed student's t-test post hoc for each time point in panel **b**. **p*<0.05 and ***p*<0.01. Different exposure times were required for each antibody (*see* uncropped images in **Supplementary Data 5**). Source data are provided as a **Source Data** file.

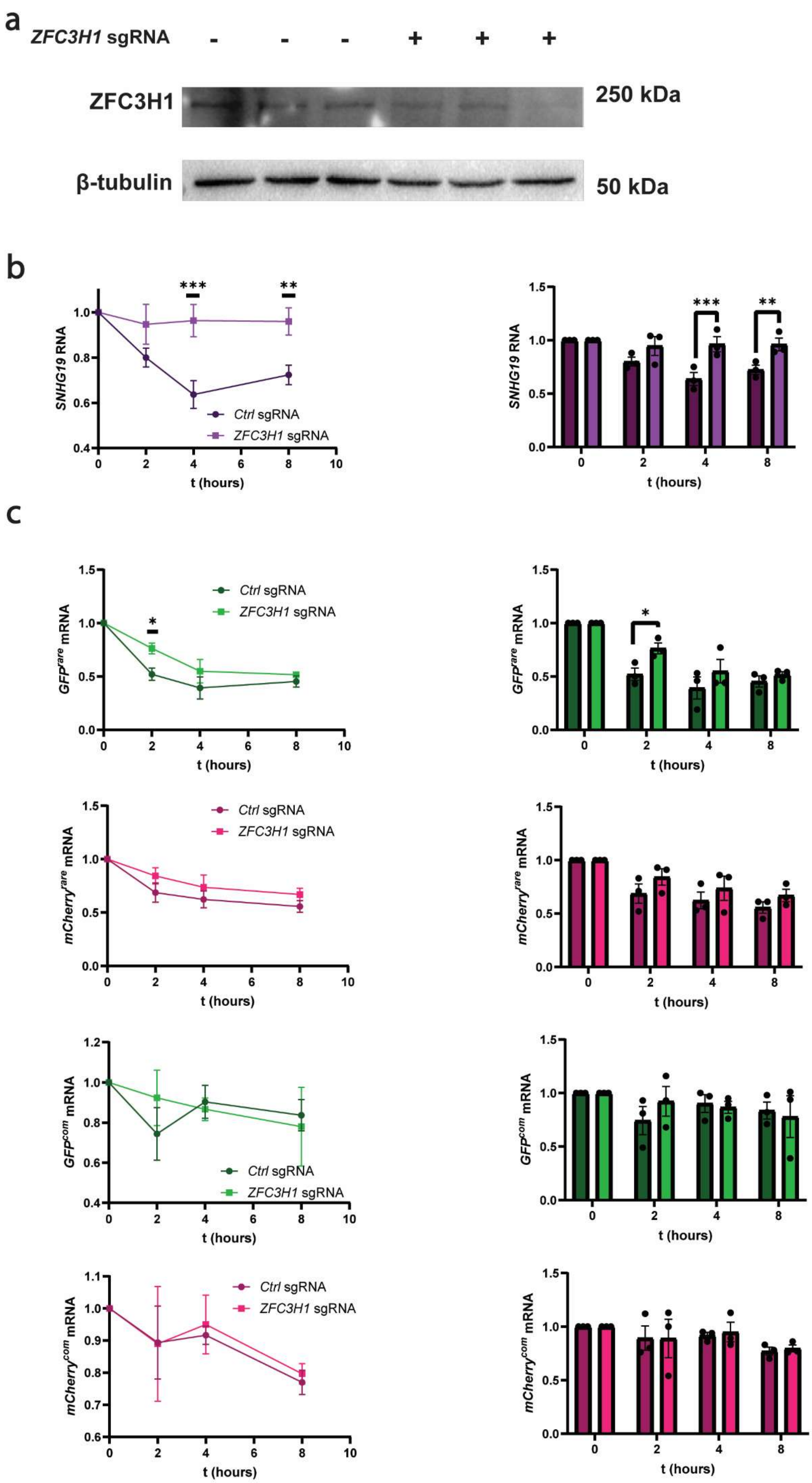
a
ZFC3H1 sgRNA - - - + + +
ZFC3H1
250 kDa
β-tubulin
50 kDa
b
SNHG19 RNA
t (hours)
Ctrl sgRNA
ZFC3H1 sgRNA
***
**
c
GFP^rare mRNA
mCherry^rare mRNA
GFP^com mRNA
mCherry^com mRNA
Ctrl sgRNA
ZFC3H1 sgRNA
t (hours)
*

Supplementary Figure 8. Repeat of the effect of ZFC3H1 loss on reporter mRNA stability. (**a**) Immunoblots of the indicated proteins from 293T cells transduced with vectors encoding either a *Ctrl* or *ZFC3H1* sgRNA. (**b,c**) Mean ± SEM of (**b**) SNHG19 RNA or (**c**) the indicated reporter mRNA, as assessed by RT-qPCR, in *Ctrl* and *ZFC3H1* sgRNA cells co-transfected with both $GFP^{rare}/mCherry^{com}$ and $mCherry^{rare}/GFP^{com}$ dual reporters after treatment with Actinomycin D for the indicated amount of time (t) normalized to *GAPDH* mRNA and t=0 values from *n*=3 biological replicate experiments (colored circles). Two-way ANOVA for *SNHG19* in panel **b** (F ratio=6.5444, p=0.0009, *n*=3 biological replicates) and each reporter in panel **c** (GFP^{rare}: F ratio=14.3688, p=0<0.0001, *n*=3 biological replicates; $mCherry^{rare}$: F ratio=5.6827, p=0.002, *n*=3 biological replicates; GFP^{com}: F ratio=0.7785, p=0.6143, *n*=3 biological replicates; $mCherry^{com}$: F ratio=1.0178, p=0.4556, *n*=3 biological replicates) with sliced two-tailed student's t-test post hoc for each time point. Different exposure times were required for each antibody (*see* uncropped images in **Supplementary Data 5**). **p*<0.05, ***p*<0.01, and ****p*<0.001. Source data are provided as a **Source Data** file.

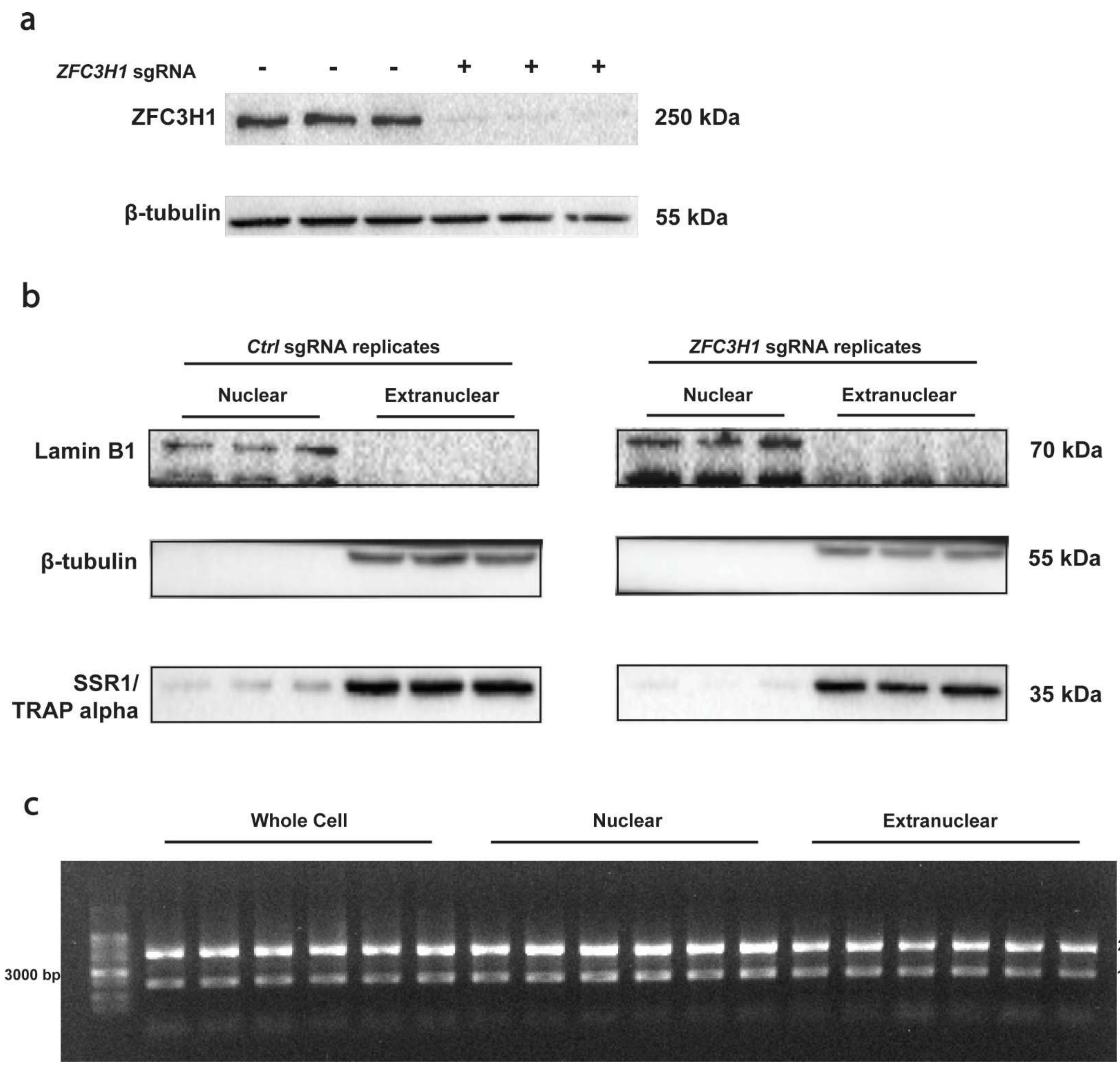

Supplementary Figure 9. Fractionation efficiency and RNA quality controls. (**a,b**) Immunoblots (3 biological replicates) of the indicated proteins from 293T cells transduced with vectors encoding either a *Ctrl* or *ZFC3H1* sgRNA and subjected to nuclear and extranuclear fractionation. (**c**) Bleach agarose gel of RNA single strand ladder (lane one) and RNA from whole cell, nuclear, and extranuclear replicates from *ZFC3H1* and *Ctrl* sgRNA cells. Different exposure times were required for each antibody (*see* uncropped images in **Supplementary Data 5**).

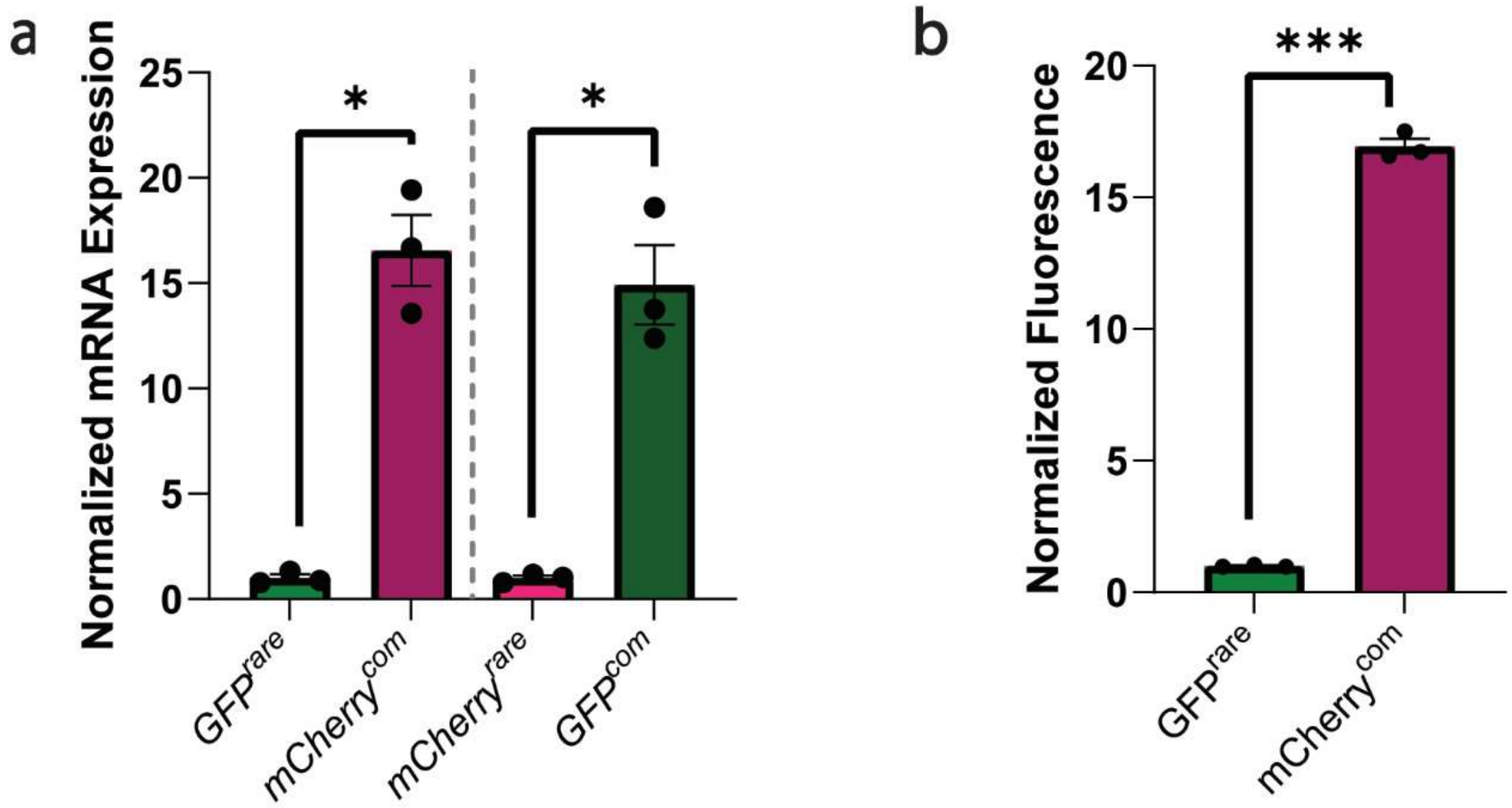

Supplementary Figure 10. Relative expression levels of reporter mRNA and protein in *Ctrl* cells. (**a**) Mean ± SEM mRNA levels from *Ctrl* sgRNA cells co-transfected with both GFP^{rare}/mCherry^{com} and mCherry^{rare}/GFP^{com} dual reporters from **Supplementary Figure 5B**, as assessed by RT-qPCR and normalized to *18S* rRNA and either *GFP^{rare}* for the GFP^{rare}/mCherry^{com} or *mCherry^{rare}* for the mCherry^{rare}/GFP^{com} dual reporter, derived from *n*=3 biological replicate experiments (black circles). (**b**) Mean ± SEM of GFP^{rare} and mCherry^{com} fluorescence in *Ctrl* sgRNA cells from **Supplementary Figure 3B**, as assessed by flow cytometry and normalized to GFP^{rare} fluorescence, derived from *n*=3 biological replicate experiments (black circles). Two-tailed Welch's t-test utilized for each rare/common comparison for panels **a** and **b**. *p<0.05 and ***p<0.001. Source data are provided as a **Source Data** file.

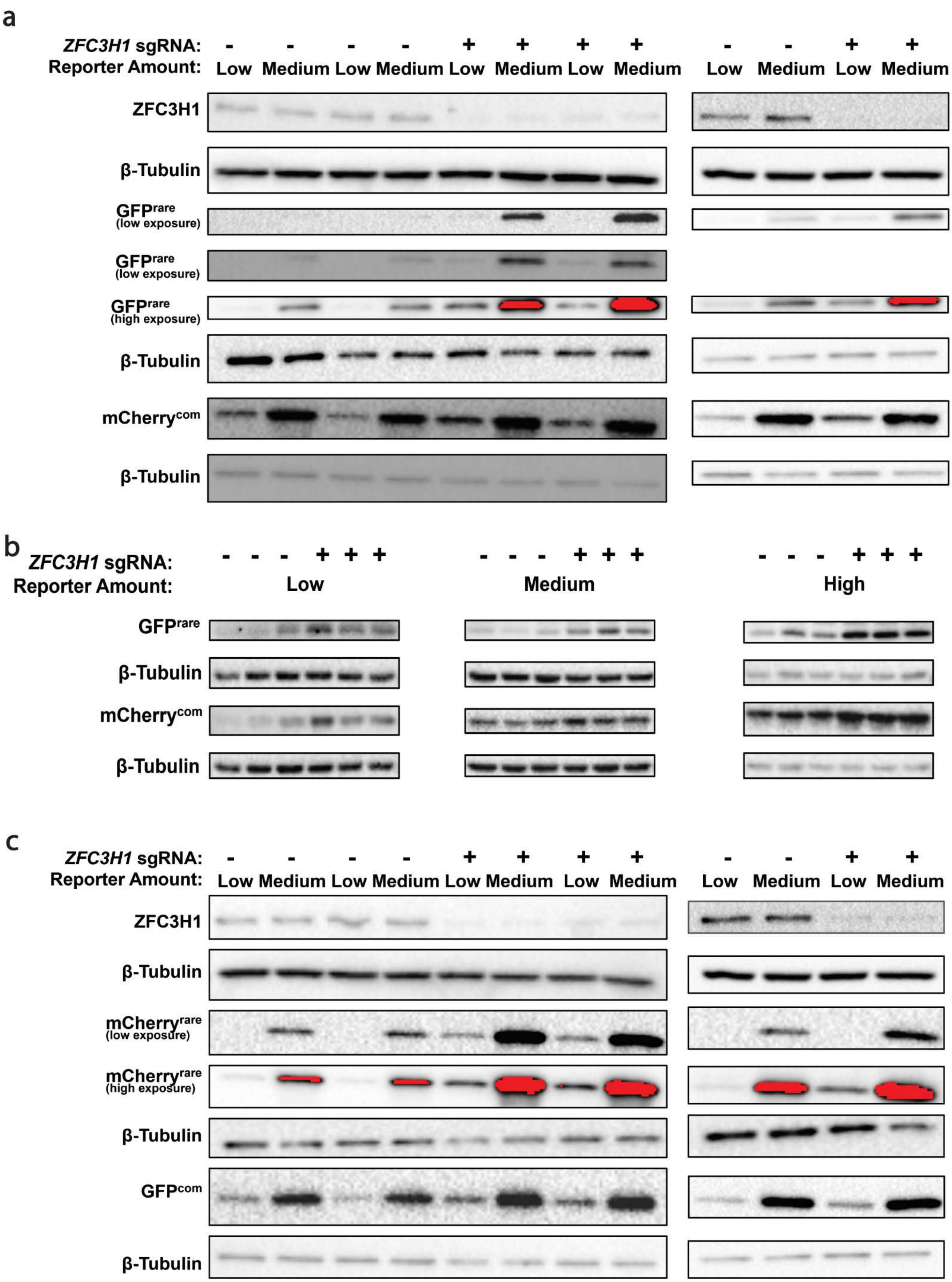
a
ZFC3H1 sgRNA: - - - - + + + + - - + +
Reporter Amount: Low Medium Low Medium Low Medium Low Medium Low Medium Low Medium
ZFC3H1
β-Tubulin
GFPrare (low exposure)
GFPrare (low exposure)
GFPrare (high exposure)
β-Tubulin
mCherrycom
β-Tubulin
b
ZFC3H1 sgRNA: - - - + + + - - - + + + - - - + + +
Reporter Amount: Low Medium High
GFPrare
β-Tubulin
mCherrycom
β-Tubulin
c
ZFC3H1 sgRNA: - - - - + + + + - - + +
Reporter Amount: Low Medium Low Medium Low Medium Low Medium Low Medium Low Medium
ZFC3H1
β-Tubulin
mCherryrare (low exposure)
mCherryrare (high exposure)
β-Tubulin
GFPcom
β-Tubulin

Supplementary Figure 11. Reporter protein levels upon ZFC3H1 loss in cells transduced with increasing amounts of dual reporters. (**a,b,c**) Immunoblots for the indicated proteins from 293T cells transduced with vectors encoding *Ctrl* or *ZFC3H1* sgRNA and transfected with low (0.4 µg), medium (1 µg), or high (2 µg) amounts of the (**a,b**) $GFP^{rare}/mCherry^{com}$ (*n*=3 (high) or 6 (low to medium) biological replicates) or (**c**) $mCherry^{rare}/GFP^{com}$ dual reporter (*n*=3 biological replicates). Immunoblot of ZFC3H1 for (**b**) is shown in **Supplementary Figure 5A.** Different exposure times were required for each antibody (*see* uncropped images in **Supplementary Data 5**).

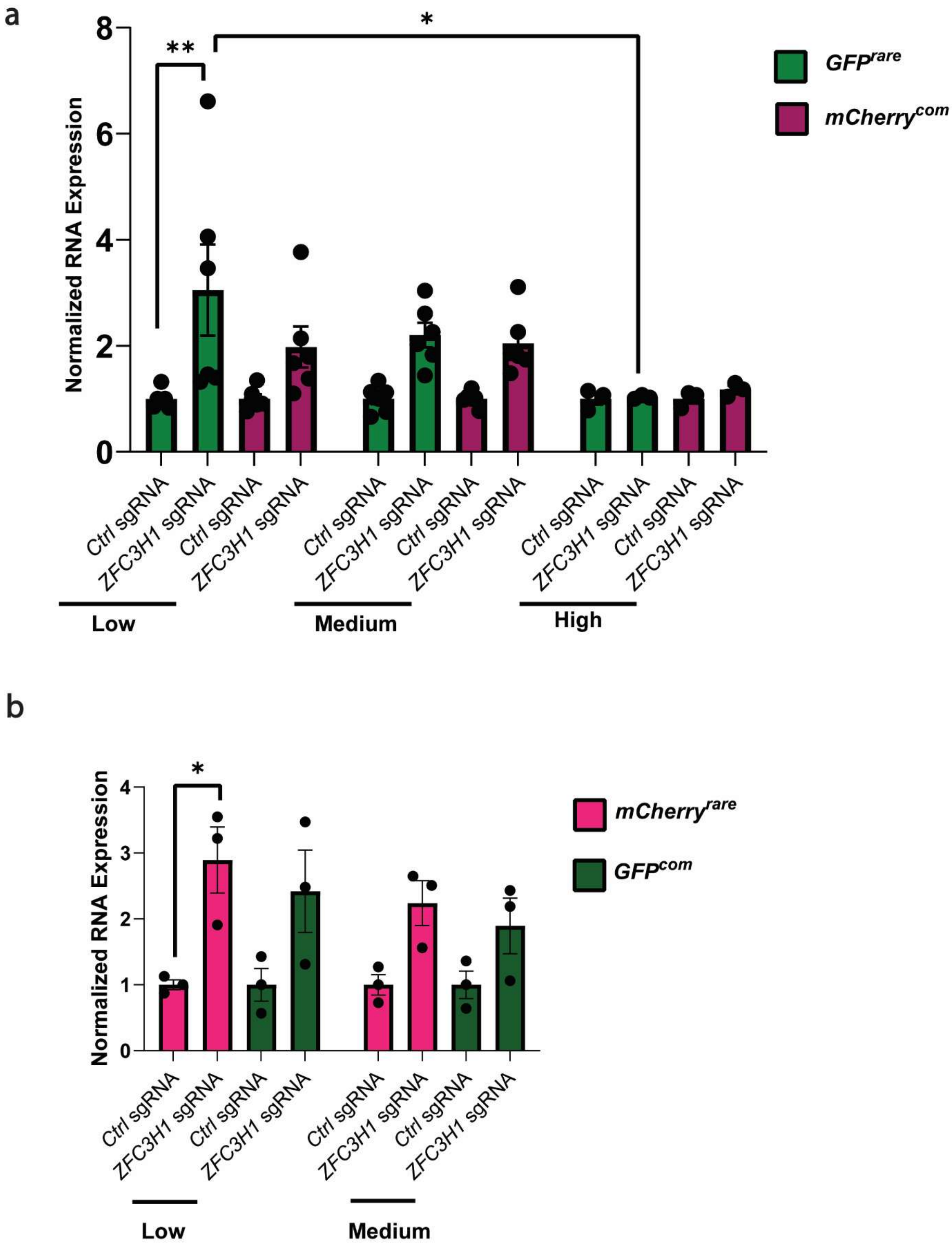

Supplementary Figure 12. Reporter mRNA levels upon ZFC3H1 loss in cells transduced with increasing amounts of dual reporters (**a**,**b**) Mean ± SEM of mRNA levels from 293T cells transduced with vectors encoding *Ctrl* or *ZFC3H1* sgRNA and transfected with low (0.4 µg), medium (1 µg), or high (2 µg) amounts of the (**a**) GFP^{rare}/$mCherry^{com}$ or (**b**) $mCherry^{rare}$/GFP^{com} dual reporter, as assessed by RT-qPCR and normalized to *18S* rRNA and *Ctrl* sgRNA cells, derived from *n*=3-6 biological replicate experiments (black circles). Three-way ANOVA and Tukey's post hoc performed for panel **a** (Exp 1: F ratio=4.0915, *p*=0.0003, *n*=3 (high) or *n*=6 (low, medium) biological replicates) and **b** (F ratio=4.5125, *p*=0.006, *n*=3 biological replicates). *$p<0.05$ and **$p<0.01$. Source data are provided as a **Source Data** file.

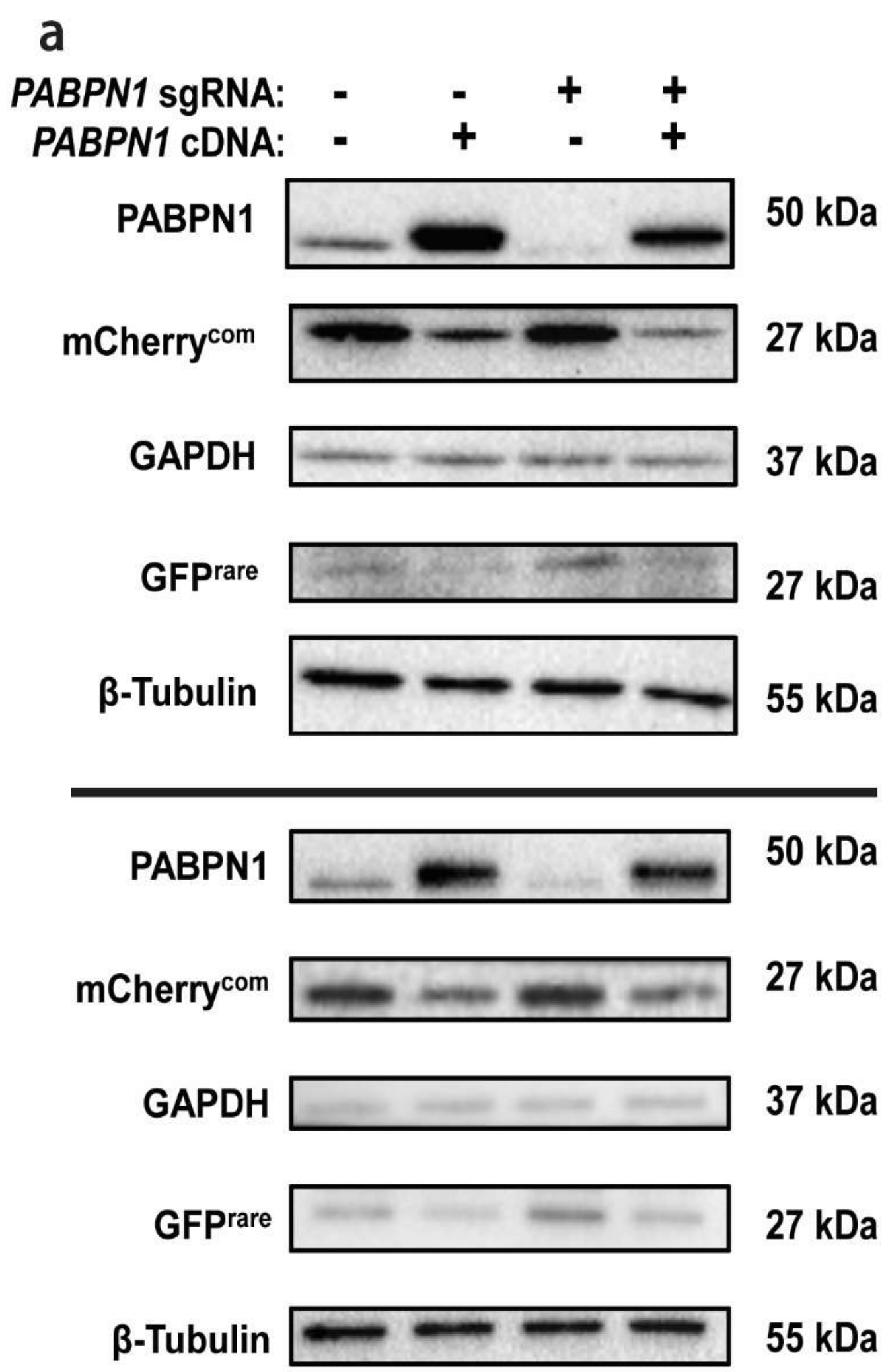

Supplementary Figure 13. Reporter protein levels upon PABPN1 depletion and rescue. (**a**,**b**) Replicate immunoblots (2 of 4 biological replicates) for the indicated proteins from 293T cells transduced with vectors encoding a *Ctrl* or *PABPN1* sgRNA, no transgene or *PABPN1* cDNA, and the (**a**) $GFP^{rare}/mCherry^{com}$ or (**b**) $mCherry^{rare}/GFP^{com}$ dual reporter. Black lines separate individual replicates for each panel. Different exposure times were required for each antibody (*see* uncropped images in **Supplementary Data 5**).

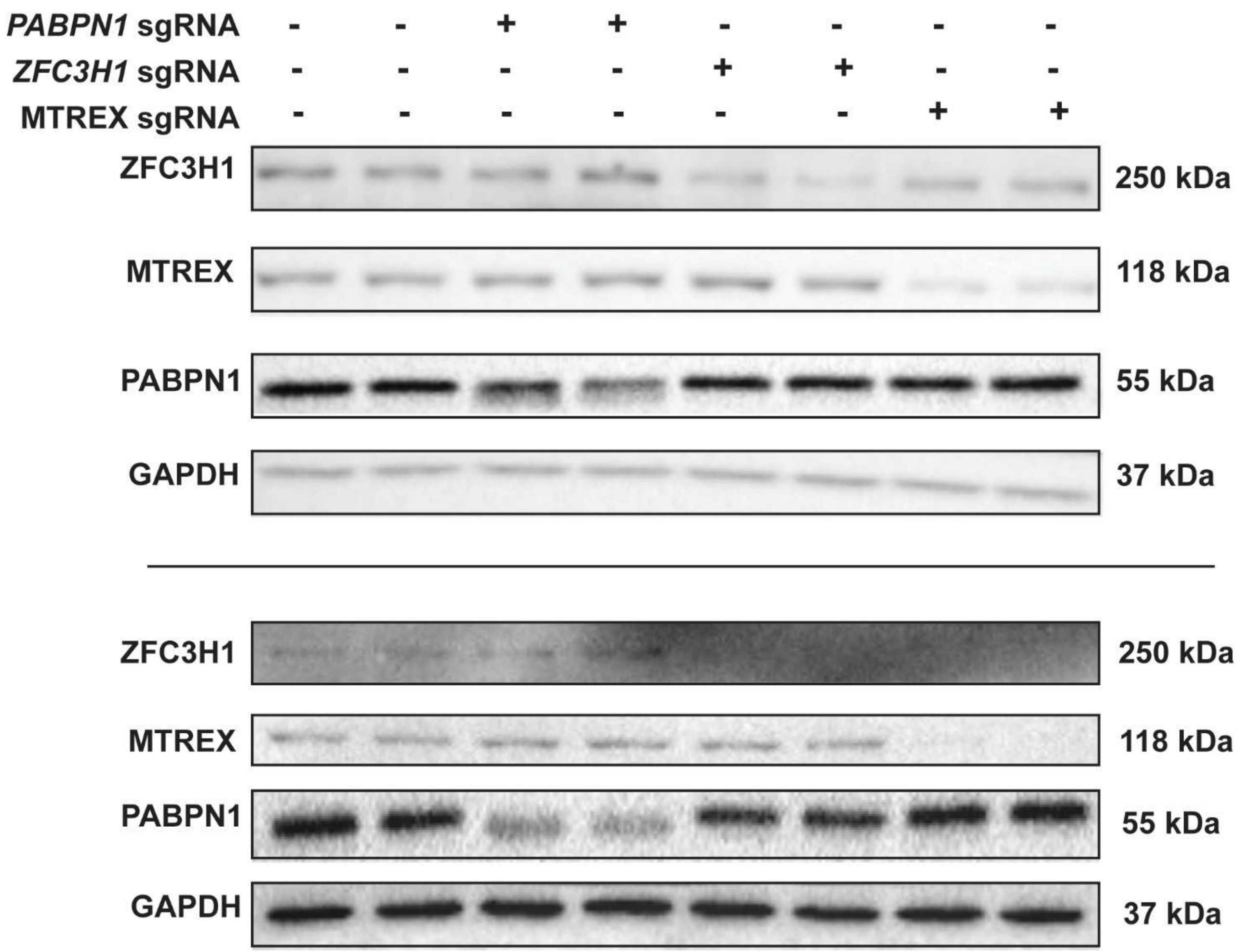

Supplementary Figure 14. Protein levels of the three core subunits of the PAXT components upon their individual depletion. Immunoblots for the indicated proteins from 293T cells transduced with vectors encoding a *Ctrl*, *PABPN1, ZFC3H1,* or *MTREX* sgRNA. *n*=4 biological replicate experiments. Different exposure times were required for each antibody (*see* uncropped images in **Supplementary Data 5**).

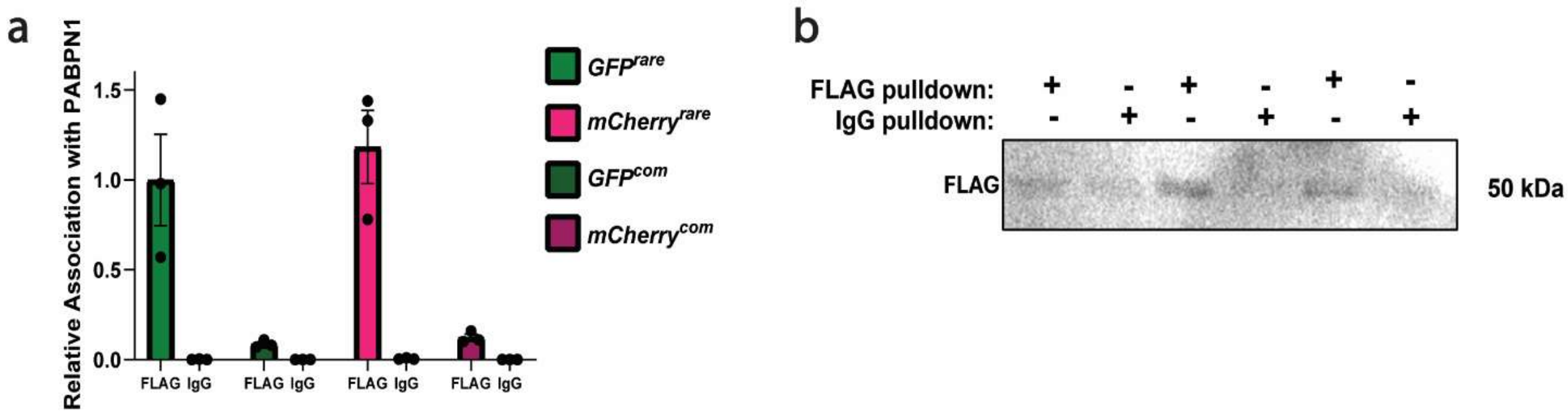

Supplementary Figure 15. ***PABPN1* RNA immunoprecipitation controls.** (**a**) Mean ± SEM of indicated mRNA transcripts co-immunoprecipitated with an anti-FLAG or IgG antibody from 293T cells co-transfected with both GFP^{rare}/$mCherry^{com}$ and $mCherry^{rare}$/GFP^{com} dual reporters and a vector encoding FLAG-PABPN1, as assessed by RT-qPCR and normalized to input mRNA levels of each reporter in the cell and then to the level of GFP^{rare} mRNA co-immunoprecipitated with an anti-FLAG antibody. Black circles indicate n=3 biological replicates. (**b**) Immunoblot from the same cells of FLAG-tagged proteins immunoprecipitated (pulldown) with an anti-FLAG or IgG antibody and detected with an anti-FLAG antibody. Source data are provided as a **Source Data** file.

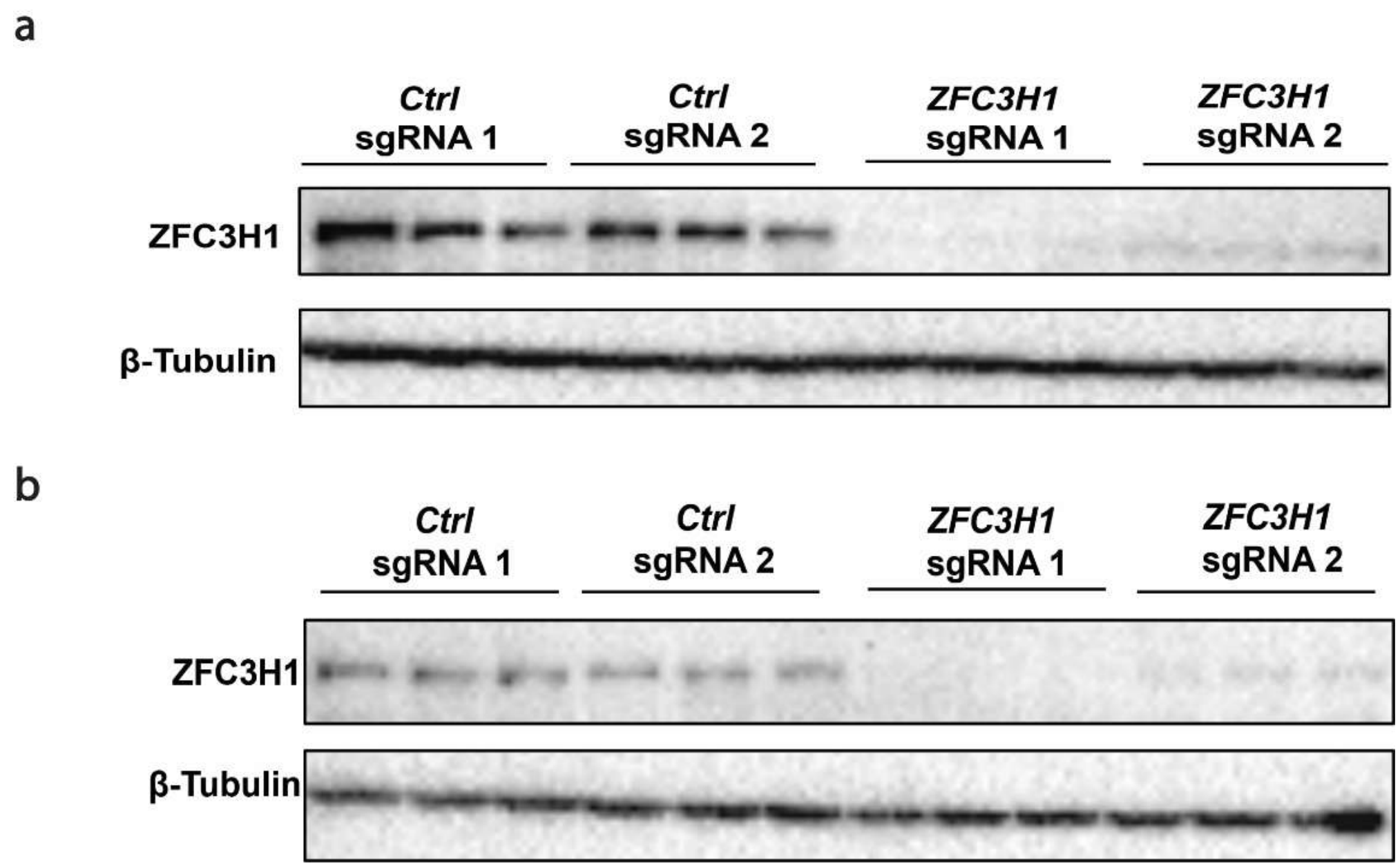

Supplementary Figure 16. ZFC3H1 protein levels for samples subjected to RNA sequencing and proteomics. (**a**,**b**) Immunoblots (*n*=6 biological replicates) for the indicated proteins from 293T cells infected with one of two different *Ctrl* or *ZFC3H1*-targeting sgRNAs and then prepared for (**a**) transcriptomic or (**b**) proteomic analysis. Different exposure times were required for each antibody (*see* uncropped images in **Supplementary Data 5**).

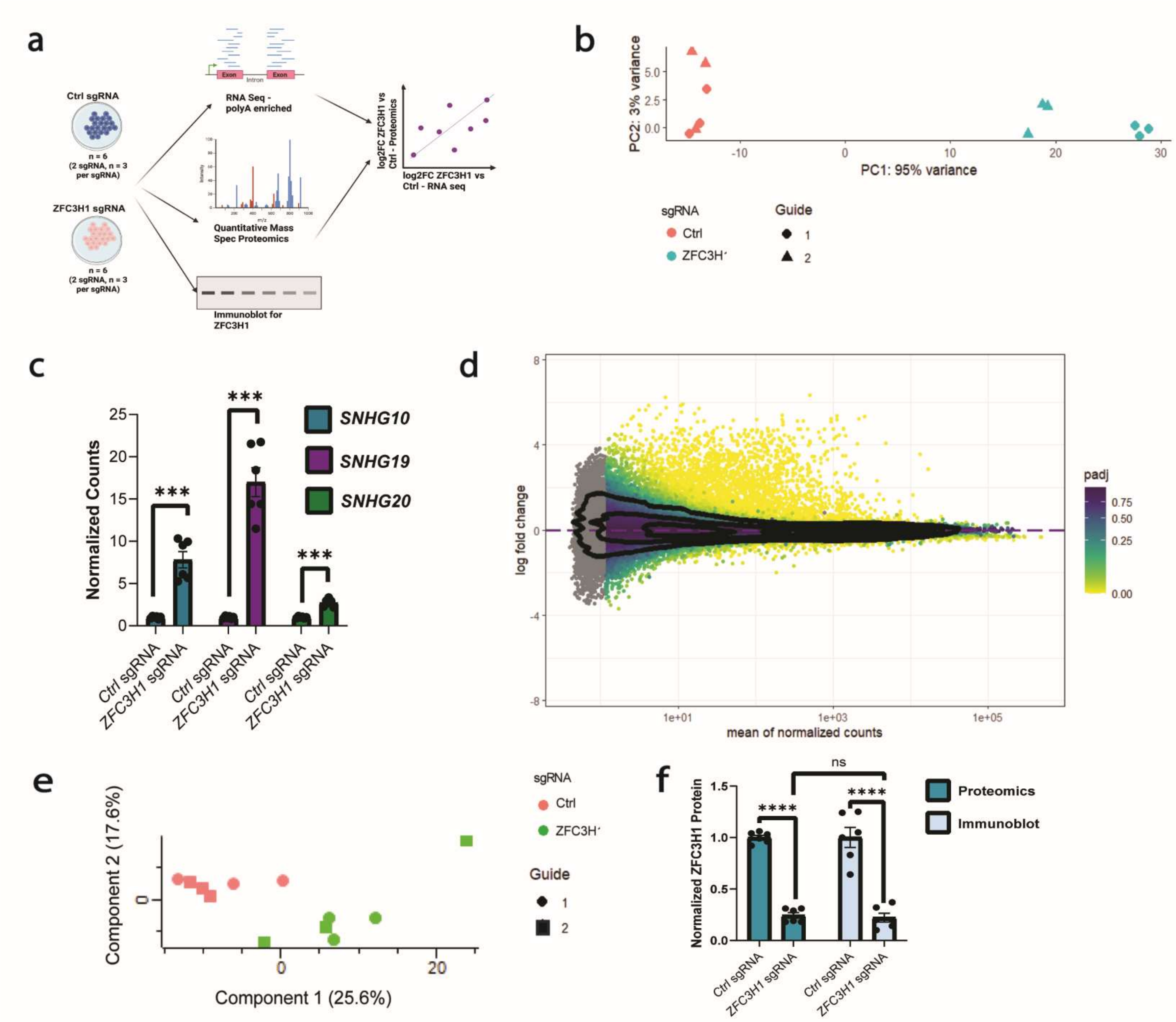

Supplementary Figure 17. Quality control of RNA-Seq and proteomics upon ZFC3H1 loss. (**a**) Schematic of the approach (not actual data images, Created in BioRender. Niehoff, T. (2026) https://BioRender.com/6pi5xh3). (**b,e**) Principal component analysis of the (**b**) transcriptomes and (**e**) proteomes derived from triplicate cultures of 293T cells transduced with a vector encoding one of two different (triangle and square) *Ctrl* (pink) or *ZFC3H1* (green) sgRNAs. (**c**) Mean ± SEM of the indicated normalized transcript read counts from the transcriptome dataset for both *Ctrl* and *ZFC3H1* sgRNA cells, normalized to *Ctrl*. (**d**) Mean of normalized counts for each detected transcript versus its log fold change, color coded by the significance (padj) of the log fold change. Log_2 FC>0 indicates transcripts significantly increased with ZFC3H1 loss. (**f**) Mean ± SEM of average peptide counts of ZFC3H1 normalized to the average total number of peptides determined by mass spectrometry or immunoblot in the six *Ctrl* versus six *ZFC3H1* sgRNAs cell lines. Two-tailed Welch's t-test performed for each transcript in (**c**) and two-way ANOVA performed with Tukey's post hoc test for panel (**f**) (n=6 biological replicates, F ratio=63.6221, p<0.0001). DESeq2 R package performed to obtain analysis in (**d**). ns: nonsignificant, ***p<0.001, and ****p<0.0001. n=6 biological replicates (denoted by individual circles or squares). Source data are provided as a **Source Data** file.

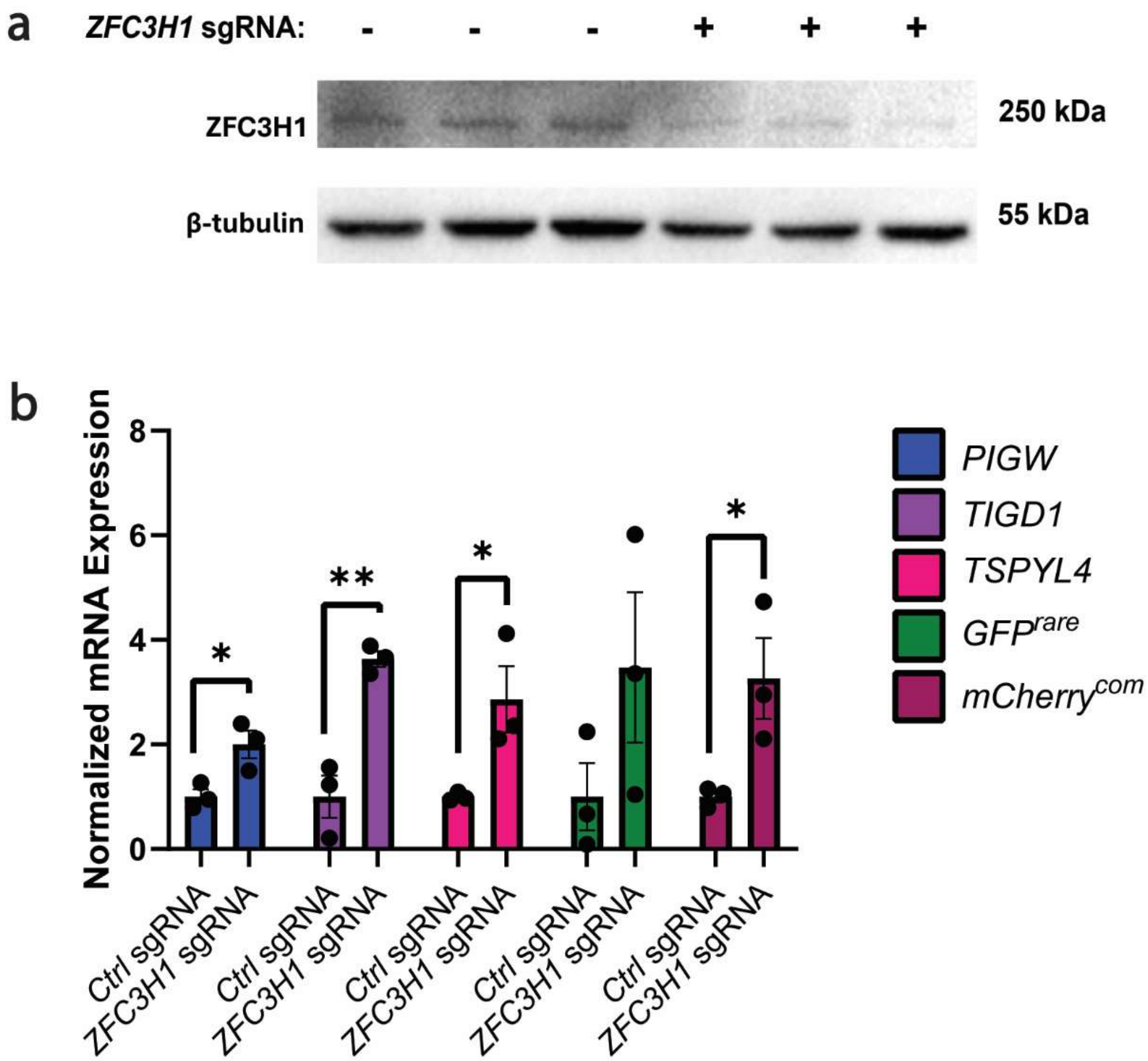

Supplementary Figure 18. RT-qPCR validation of increased levels of identified targets upon ZFC3H1 loss. (**a**) Immunoblots of the indicated proteins (*right*: predicted molecular weight of the indicated protein) from 293T cells transduced with a vector encoding *Ctrl* or *ZFC3H1* sgRNA and the GFP^{rare}/$mCherry^{com}$ dual reporter, derived from *n*=3 biological replicate experiments. (**b**) Mean ± SEM of mRNA levels from the same cells, as assessed by RT-qPCR and normalized to *18S* rRNA and *Ctrl* sgRNA cells, derived from *n*=3 biological replicate experiments (black circles). One-tailed Welch's t-test performed for each reporter and each transcript in panel **b.** $^*p<0.05$ and $^{**}p<0.01$. Different exposure times were required for each antibody (*see* uncropped images in **Supplementary Data 5**). Source data are provided as a **Source Data** file.

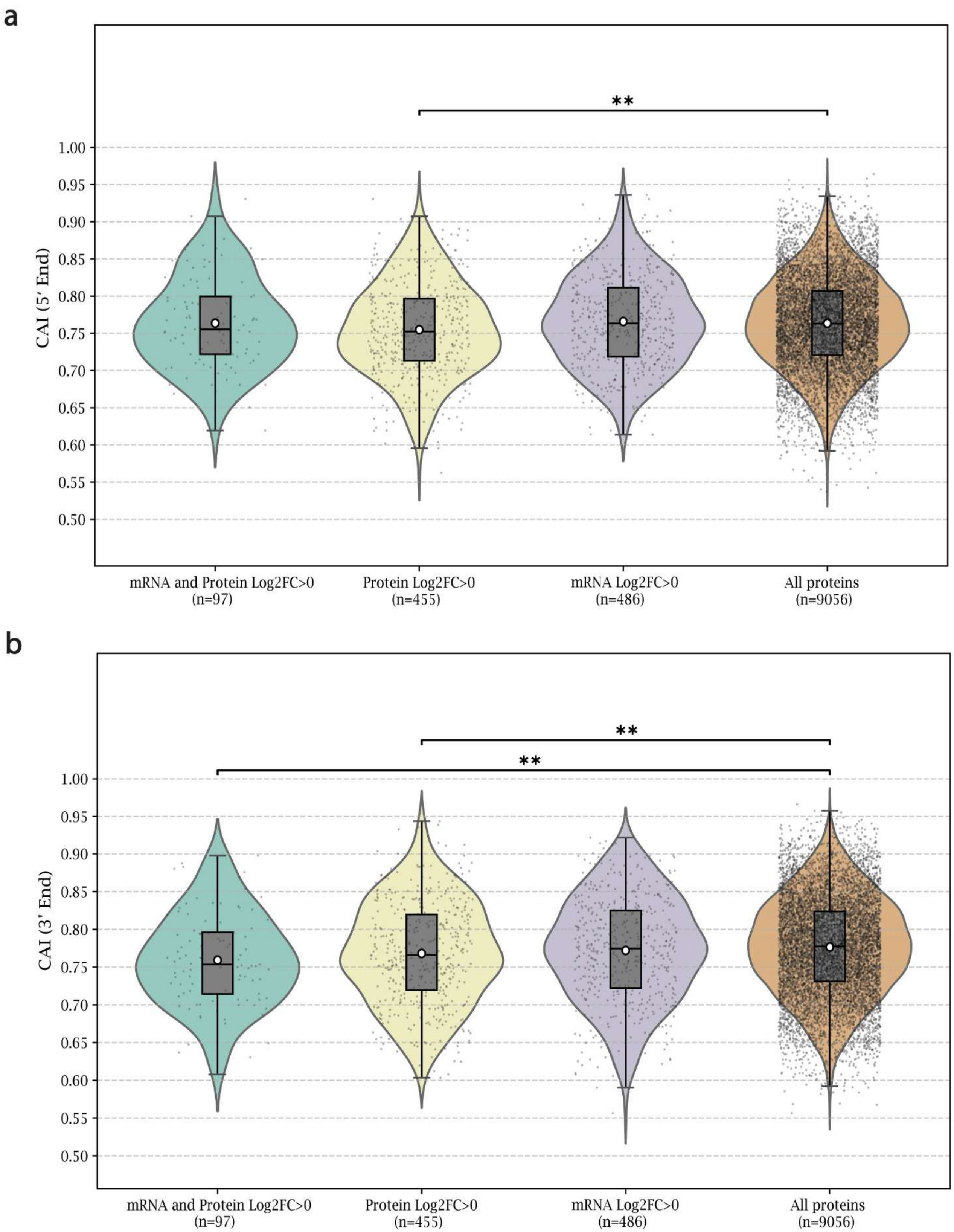

Supplementary Figure 19. Codon usage at the 5' and 3' end of endogenous genes exhibiting increased expression upon ZFC3H1 loss. (**a,b**) Violin plot distributions of CAI at the (**a**) 5' (first 50 codons/150 nucleotides) and (**b**) 3' (last 50 codons/150 nucleotides) end of the Coding DNA Sequence (CDS) of genes in the indicated groups. Two-sided *p*-values comparing the mean of each distribution to all genes detected by proteomics distribution (orange), using a size-matched random subsampling approach. For violin plots, white dots indicate mean of population. Boxes in boxplots represent Q1, median, and Q3. Whiskers represent 1.5x IQR, excluding outliers. **$p<0.01$.Source data are provided as a **Source Data** file.

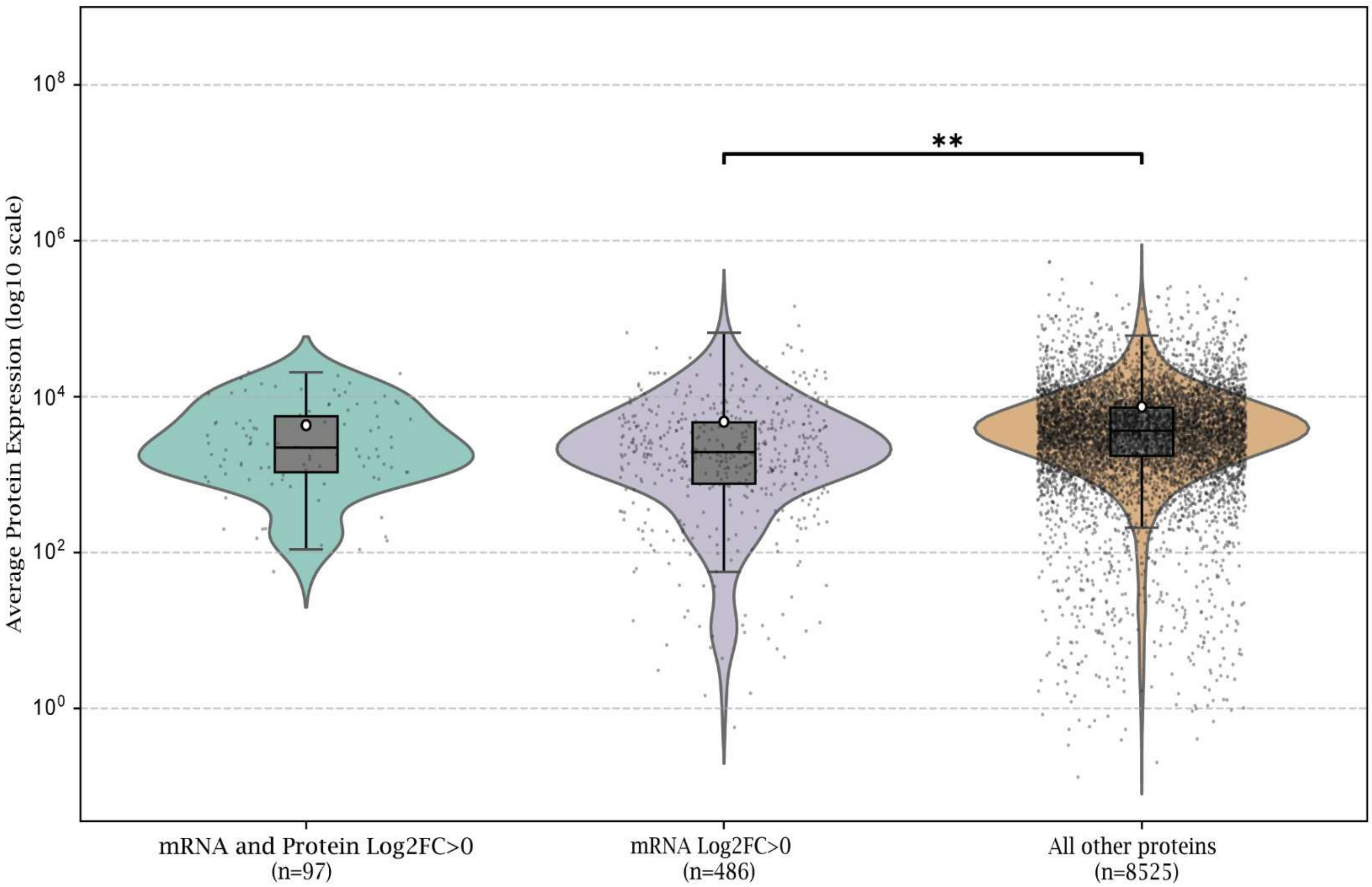

Supplementary Figure 20. Protein expression of endogenous genes with increased transcript expression upon ZFC3H1 loss. Violin plot distributions of the $\log_{10}$ average of the normalized peptide counts in *Ctrl* sgRNA cells for the indicated groups. Two-sided *p*-values comparing the mean of each distribution to all other genes detected by proteomics distribution (orange), using a size-matched random subsampling approach. For violin plots, white dots indicate mean of population. Boxes in boxplots represent Q1, median, and Q3. Whiskers represent 1.5x IQR, excluding outliers. $^{**}p<0.01$. Source data are provided as a **Source Data** file.

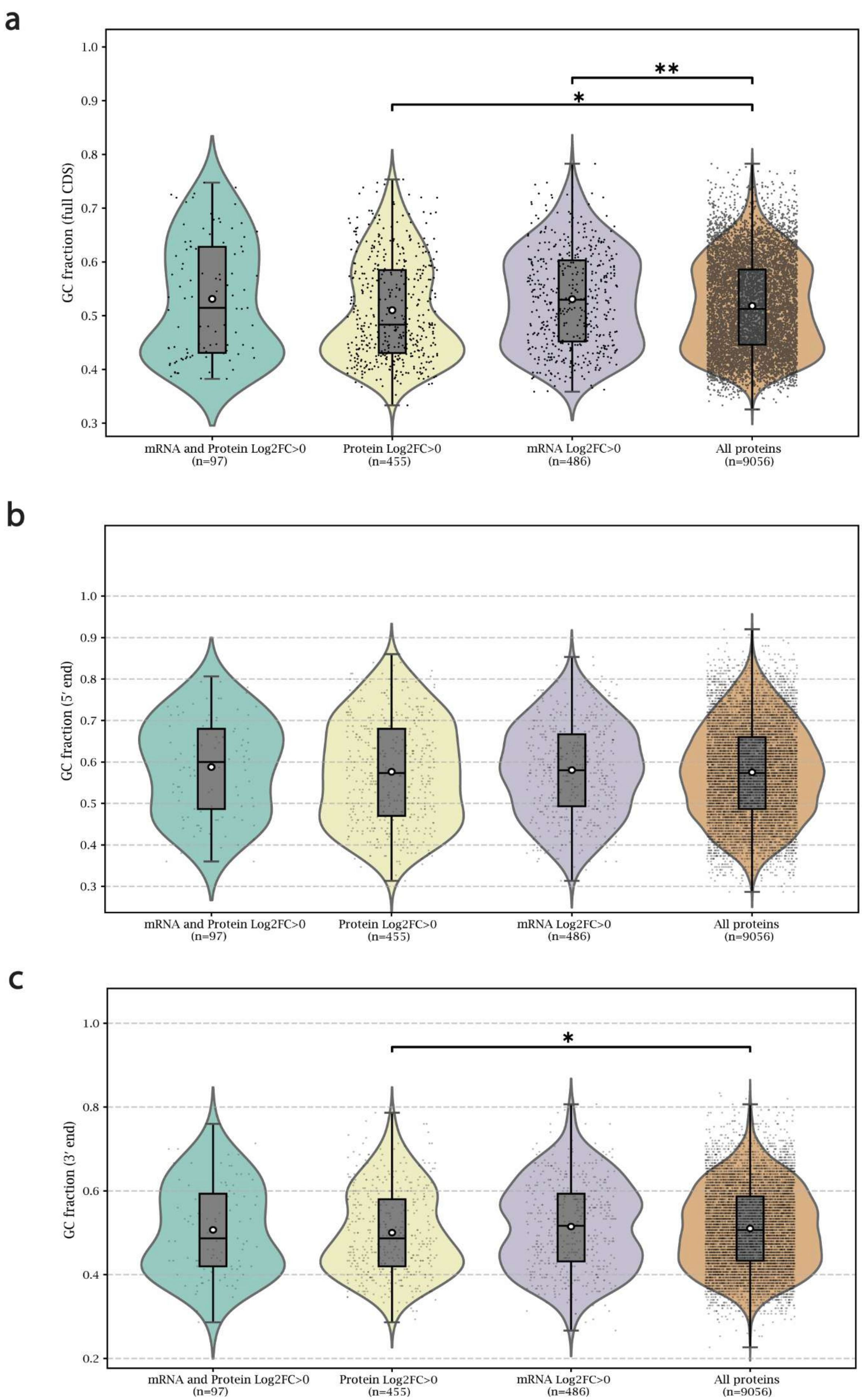
a
GC fraction (full CDS)
1.0
0.9
0.8
0.7
0.6
0.5
0.4
0.3
**
*
mRNA and Protein Log2FC>0
(n=97)
Protein Log2FC>0
(n=455)
mRNA Log2FC>0
(n=486)
All proteins
(n=9056)
b
GC fraction (5′ end)
1.0
0.9
0.8
0.7
0.6
0.5
0.4
0.3
mRNA and Protein Log2FC>0
(n=97)
Protein Log2FC>0
(n=455)
mRNA Log2FC>0
(n=486)
All proteins
(n=9056)
c
GC fraction (3′ end)
1.0
0.8
0.6
0.4
0.2
*
mRNA and Protein Log2FC>0
(n=97)
Protein Log2FC>0
(n=455)
mRNA Log2FC>0
(n=486)
All proteins
(n=9056)

Supplementary Figure 21. GC content at the full, 5', and 3' end of endogenous genes with increased expression upon ZFC3H1 loss. (**a**,**b**,**c**) Violin plot distributions of GC% at the (**a**) full, (**b**) 5', and (**c**) 3' end of the Coding DNA Sequence (CDS) of genes in the indicated groups. Two-sided *p*-values comparing the mean of each distribution to all genes detected by proteomics distribution (orange), using a size-matched random subsampling approach. For violin plots, white dots indicate mean of population. Boxes in boxplots represent Q1, median, and Q3. Whiskers represent 1.5x IQR, excluding outliers. *$p<0.05$ and **$p<0.01$. Source data are provided as a **Source Data** file.

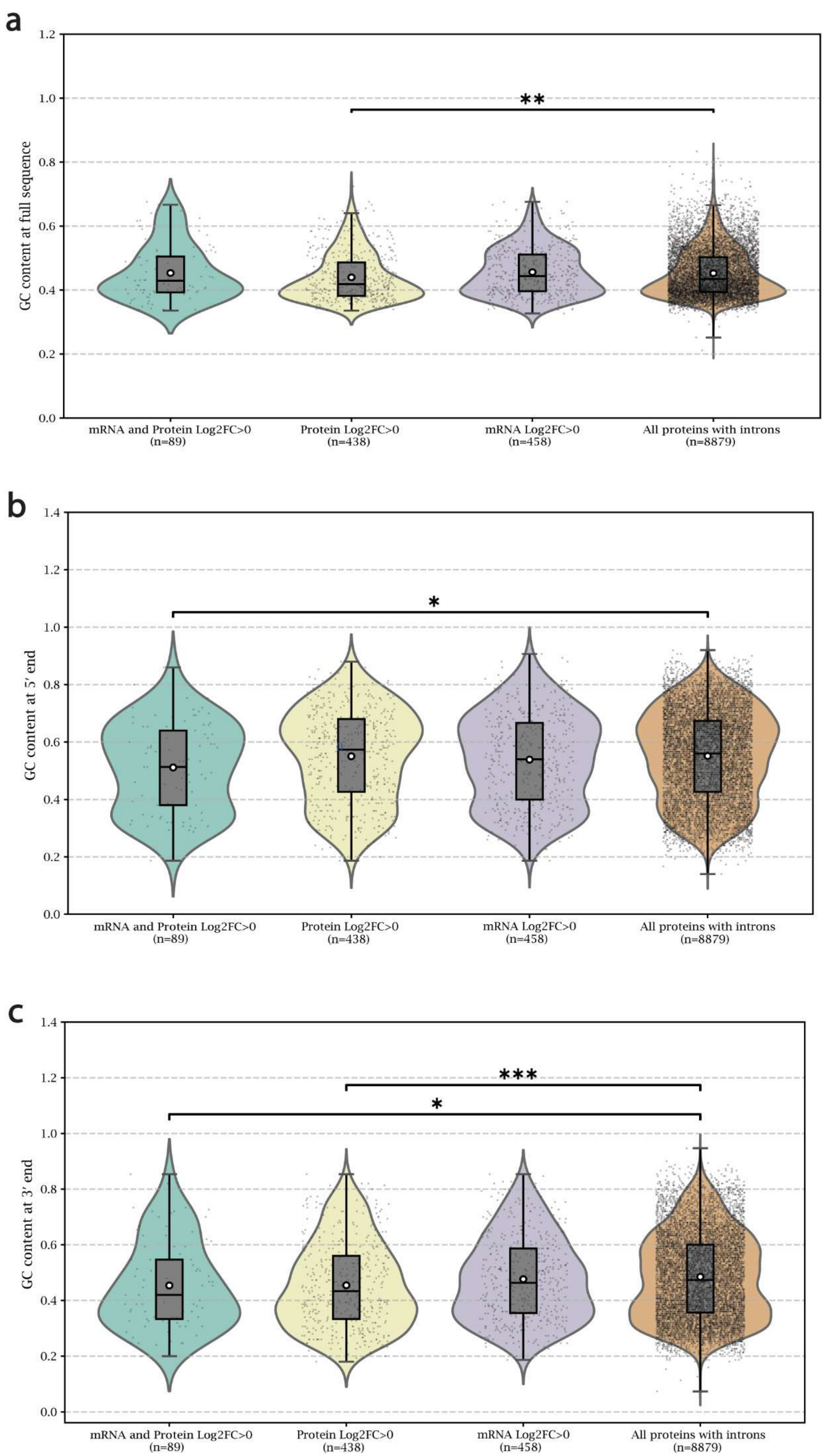
a
GC content at full sequence
**
mRNA and Protein Log2FC>0
(n=89)
Protein Log2FC>0
(n=438)
mRNA Log2FC>0
(n=458)
All proteins with introns
(n=8879)
b
GC content at 5' end
*
mRNA and Protein Log2FC>0
(n=89)
Protein Log2FC>0
(n=438)
mRNA Log2FC>0
(n=458)
All proteins with introns
(n=8879)
c
GC content at 3' end
***
*
mRNA and Protein Log2FC>0
(n=89)
Protein Log2FC>0
(n=438)
mRNA Log2FC>0
(n=458)
All proteins with introns
(n=8879)

Supplementary Figure 22. Intron GC content at the full, 5', and 3' end of endogenous genes with increased expression upon ZFC3H1 loss. (**a,b,c**) Violin plot distributions of intron GC% at the (**a**) full (**b**) 5' (first 150 nucleotides) and (**c**) 3' (last 150 nucleotides) end of intron DNA sequence of genes in the indicated groups. Two-sided *p*-values comparing the mean of each distribution to all genes detected by proteomics distribution (orange), using a size-matched random subsampling approach. For violin plots, white dots indicate mean of population. Boxes in boxplots represent Q1, median, and Q3. Whiskers represent 1.5x IQR, excluding outliers. *$p<0.05$, **$p<0.01$, and ***$p<0.001$. Source data are provided as a **Source Data** file.

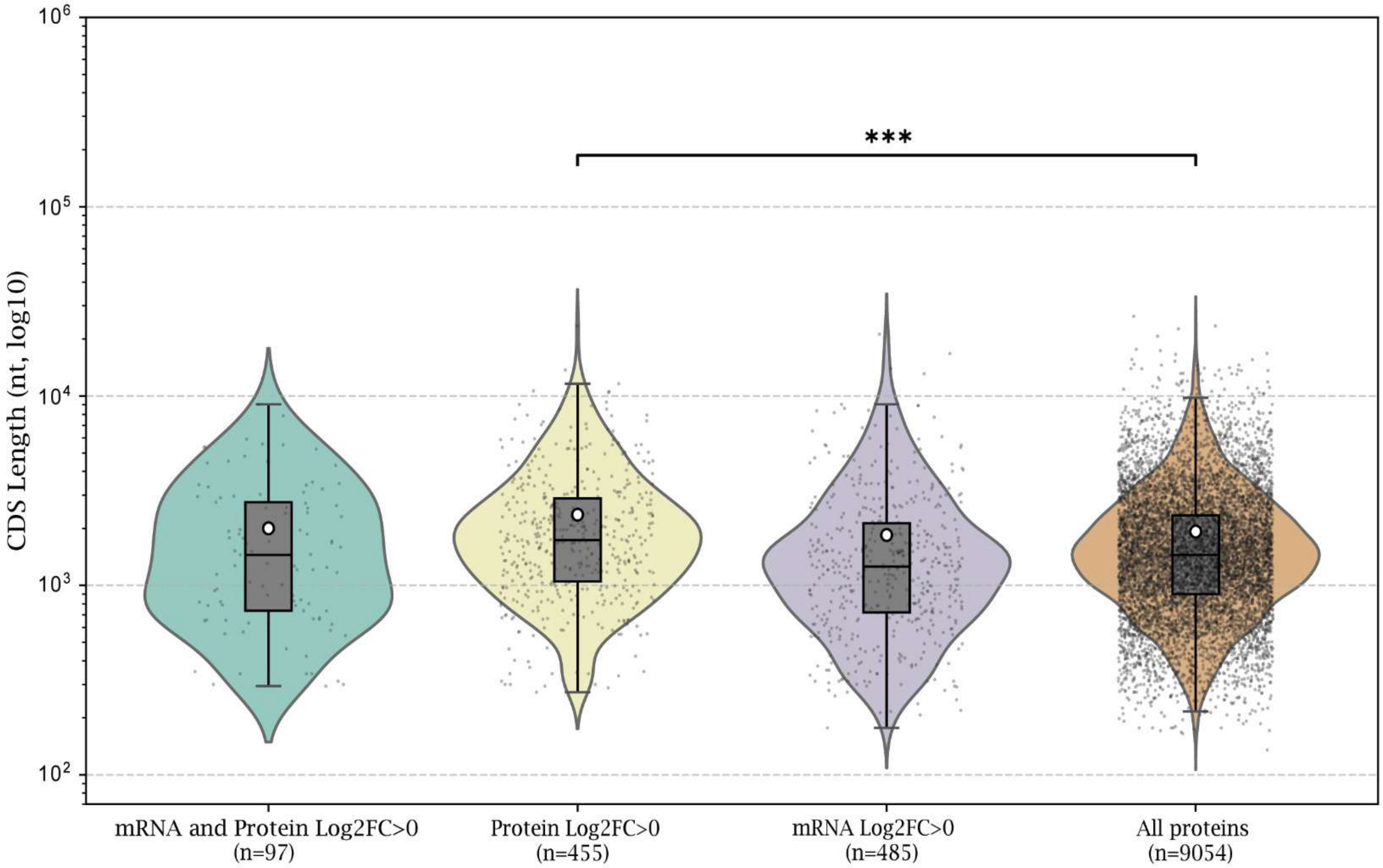

Supplementary Figure 23. Coding DNA sequence length of endogenous genes with increased expression upon ZFC3H1 loss. Violin plot distributions of the log_{10} *C*oding *D*NA *S*equence (CDS) length (nucleotides) for the indicated groups. Two-sided *p*-values comparing the mean of each distribution to all genes detected by proteomics distribution (orange), using a size-matched random subsampling approach. For violin plots, white dots indicate mean of population. Boxes in boxplots represent Q1, median, and Q3. Whiskers represent 1.5x IQR, excluding outliers. ***p<0.001. Source data are provided as a **Source Data** file.

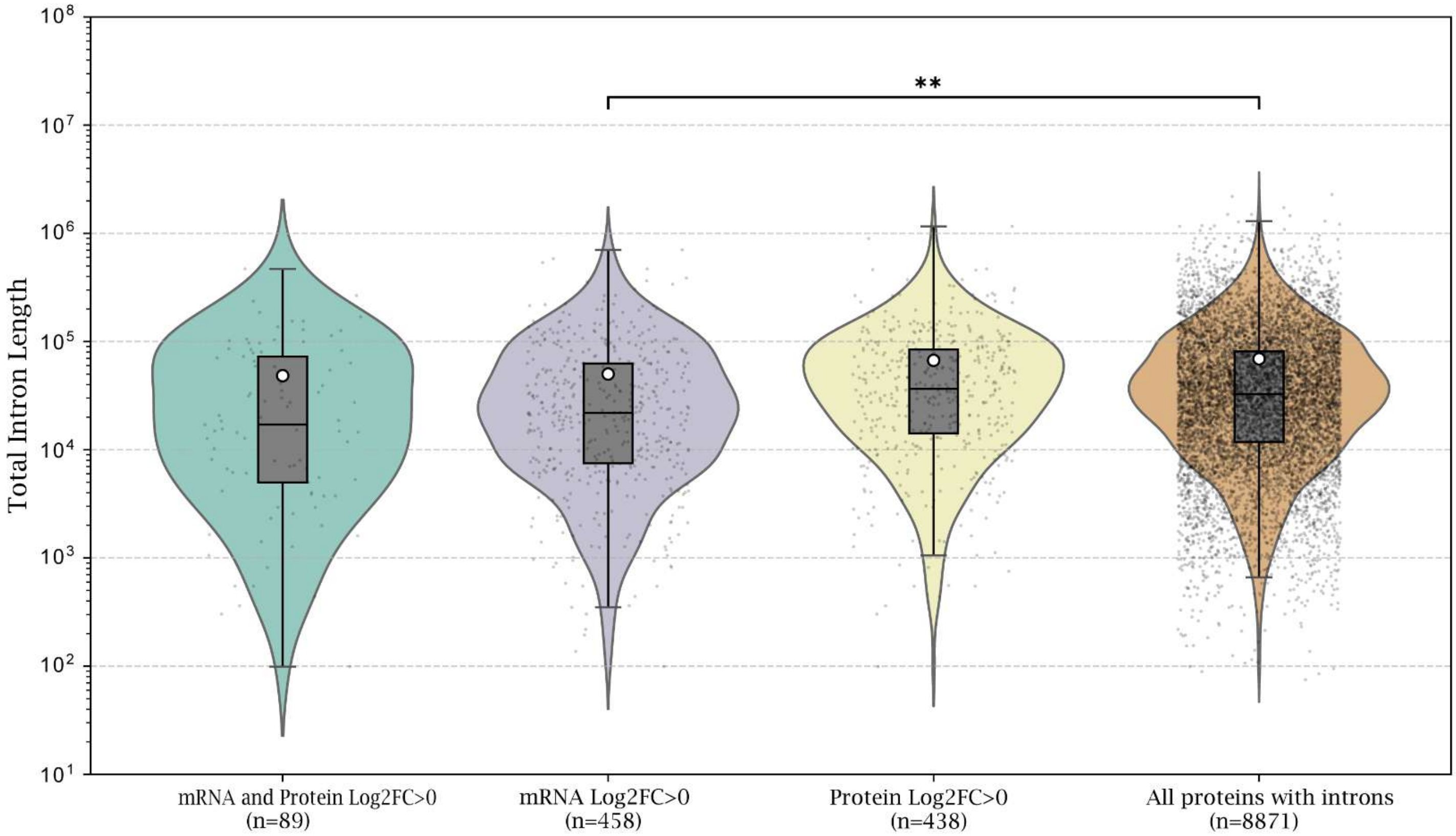

Supplementary Figure 24. Total intron length of endogenous genes with increased expression upon ZFC3H1 loss. Violin plot distributions of the $\log_{10}$ total intron length (nucleotides) for genes in the indicated groups. Two-sided *p*-values comparing the mean of each distribution to all genes detected by proteomics distribution (orange), using a size-matched random subsampling approach. For violin plots, white dots indicate mean of population. Boxes in boxplots represent Q1, median, and Q3. Whiskers represent 1.5x IQR, excluding outliers. $^{**}p<0.01$. Source data are provided as a **Source Data** file.

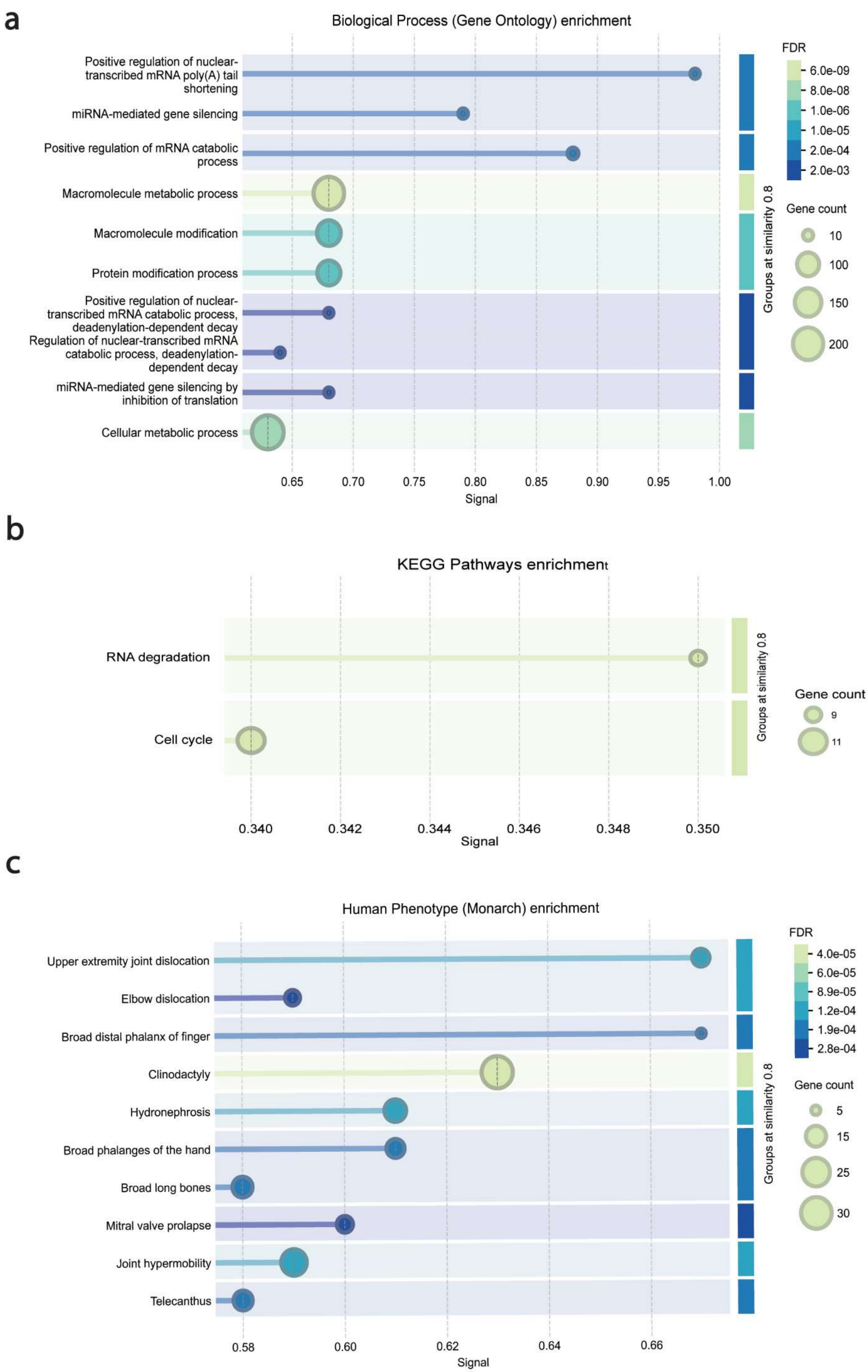
a
Biological Process (Gene Ontology) enrichment
Positive regulation of nuclear-transcribed mRNA poly(A) tail shortening
miRNA-mediated gene silencing
Positive regulation of mRNA catabolic process
Macromolecule metabolic process
Macromolecule modification
Protein modification process
Positive regulation of nuclear-transcribed mRNA catabolic process, deadenylation-dependent decay
Regulation of nuclear-transcribed mRNA catabolic process, deadenylation-dependent decay
miRNA-mediated gene silencing by inhibition of translation
Cellular metabolic process
0.65
0.70
0.75
0.80
0.85
0.90
0.95
1.00
Signal
Groups at similarity 0.8
FDR
6.0e-09
8.0e-08
1.0e-06
1.0e-05
2.0e-04
2.0e-03
Gene count
10
100
150
200
b
KEGG Pathways enrichment
RNA degradation
Cell cycle
0.340
0.342
0.344
0.346
0.348
0.350
Signal
Groups at similarity 0.8
Gene count
9
11
c
Human Phenotype (Monarch) enrichment
Upper extremity joint dislocation
Elbow dislocation
Broad distal phalanx of finger
Clinodactyly
Hydronephrosis
Broad phalanges of the hand
Broad long bones
Mitral valve prolapse
Joint hypermobility
Telecanthus
0.58
0.60
0.62
0.64
0.66
Signal
Groups at similarity 0.8
FDR
4.0e-05
6.0e-05
8.9e-05
1.2e-04
1.9e-04
2.8e-04
Gene count
5
15
25
30

Supplementary Figure 25. Biological processes, pathways, and phenotypes associated with proteins that increased upon ZFC3H1 loss. (**a**) Top 15 enriched biological pathways, (**b**) significantly enriched KEGG pathways, and (**c**) top 10 enriched phenotypes for proteins (*n*=455) significantly increased upon ZFC3H1 loss, as detected by proteomics. Groupings, FDR, and signal calculated by STRING.[1]

Supplementary Table 1. Comparison of codon usage between rare and common codon reporters.

**Transcript**	**Length of coding sequence (bp)**	**CAI**	**GC%**	**GC3%**	**tAI**
GFP^{rare}	720	0.71	39	33	0.33
mCherry^{rare}	711	0.71	43	38	0.33
GFP^{com}	720	0.96	62	97	0.4
mCherry^{com}	711	0.95	63	95	0.4

Supplementary Table 2. Top five biological process GO terms from STRING analysis[1] of ten genes identified from the validation screen.

GO term	Description	Count in gene set	FDR
1903311	Regulation of mRNA metabolic process	4 of 238	0.0035
1904355	Positive regulation of telomere capping	2 of 17	0.0078
0071407	Cellular response to organic cyclic compound	4 of 505	0.0078
0071396	Cellular response to lipid	4 of 486	0.0078
0061157	mRNA destabilization	2 of 23	0.0078

Supplementary Table 3. Significantly ($p<0.05$, as assessed using a binomial test in SEA – Simple Enrichment Analysis[2]) enriched RBP motifs in genes with log2 FC > 0 increase in mRNA levels upon ZFC3H1 loss (*n* = 436) compared to all genes detected by proteomics (*n* = 8151), ordered by the number of genes the motif is present in.

Motif	RBP	p-value	Number of genes with motif	Enrichment ratio (number of genes with motif in population of interest compared to control population)
GCGCGCG	RBM8A	0.00868	49	1.49
GCGCGSG	RBM4B	0.0147	35	1.55
SSGCGCS	PPRC1	0.00862	17	2.10
UUUUUYC	U2AF2	0.0471	15	1.77

Supplementary Table 4. **Significantly ($p < 0.05$, as assessed using a binomial test in SEA – Simple Enrichment Analysis[2]) enriched RBP motifs in genes with $\log_2$ FC > 0 increase in protein levels upon ZFC3H1 loss ($n = 410$) compared to all genes detected by proteomics ($n = 8151$), ordered by the number of genes the motif is present in.**

**Motif**	**RBP**	**p-value**	**Number of genes with motif**	**Enrichment ratio (number of genes with motif in population of interest compared to control population)**
UUUUUYC	U2AF2	0.0233	151	1.47
HUYMUUUWUWUUYK	ELAVL2	0.00633	98	1.64
CWUUUUU	CPEB2	0.0289	91	1.54
CUUUUUU	CPEB4	0.0444	89	1.51
AACAWCD	YBX2	0.0128	41	1.86
SSGCGCS	PPRC1	0.00642	24	2.32
AGAAAWU	PABPC5	0.0479	19	2.01
GCGCGSG	RBM4B	0.00455	18	2.71
AGGAURA	G3BP2	0.00591	18	2.64
GCGCGCG	RBM8A	0.0013	17	3.19

Supplementary References

1. Szklarczyk, D. *et al.* The STRING database in 2023: protein–protein association networks and functional enrichment analyses for any sequenced genome of interest. *Nucleic Acids Res.* 51, D638–D646 (2023).

2. Bailey, T. L. & Grant, C. E. SEA: Simple Enrichment Analysis of motifs. *bioRxiv (preprint)* Preprint at https://doi.org/10.1101/2021.08.23.457422 (2021).
