# Supplementary material for: The PAXT connection is a modifier of rare codon-enriched protein expression
Source: Nat Commun. 2026 Sep 17;17:10928. doi: 10.1038/s41467-026-77792-7 (PMC13680609; doi:10.1038/s41467-026-77792-7)
Supplement: Supplementary file 4 [file 41467_2026_77792_MOESM2_ESM.pdf]

Description of Additional Supplementary Files

Supplementary Data 1: Primary CRISPR screen sgRNA sequences and statistics

Supplementary Data 2: Validation CRISPR screen sgRNA sequences and statistics

Supplementary Data 3: FACS plots for flow cytometry validation of ZFC3H1 loss and rescue

Supplementary Data 4: RNA seq and proteomics processed counts and statistics

Supplementary Data 5: Uncropped immunoblots

Supplementary Data 6: Raw Cq values from RT-qPCR

Supplementary Data 7: Nucleotide sequences and antibody information
