# Supplementary material for: The PAXT connection is a modifier of rare codon-enriched protein expression
Source: Nat Commun. 2026 Sep 17;17:10928. doi: 10.1038/s41467-026-77792-7 (PMC13680609; doi:10.1038/s41467-026-77792-7)
Supplement: Supplementary file 5 [file 41467_2026_77792_MOESM5_ESM.pdf]

Untransfected Control

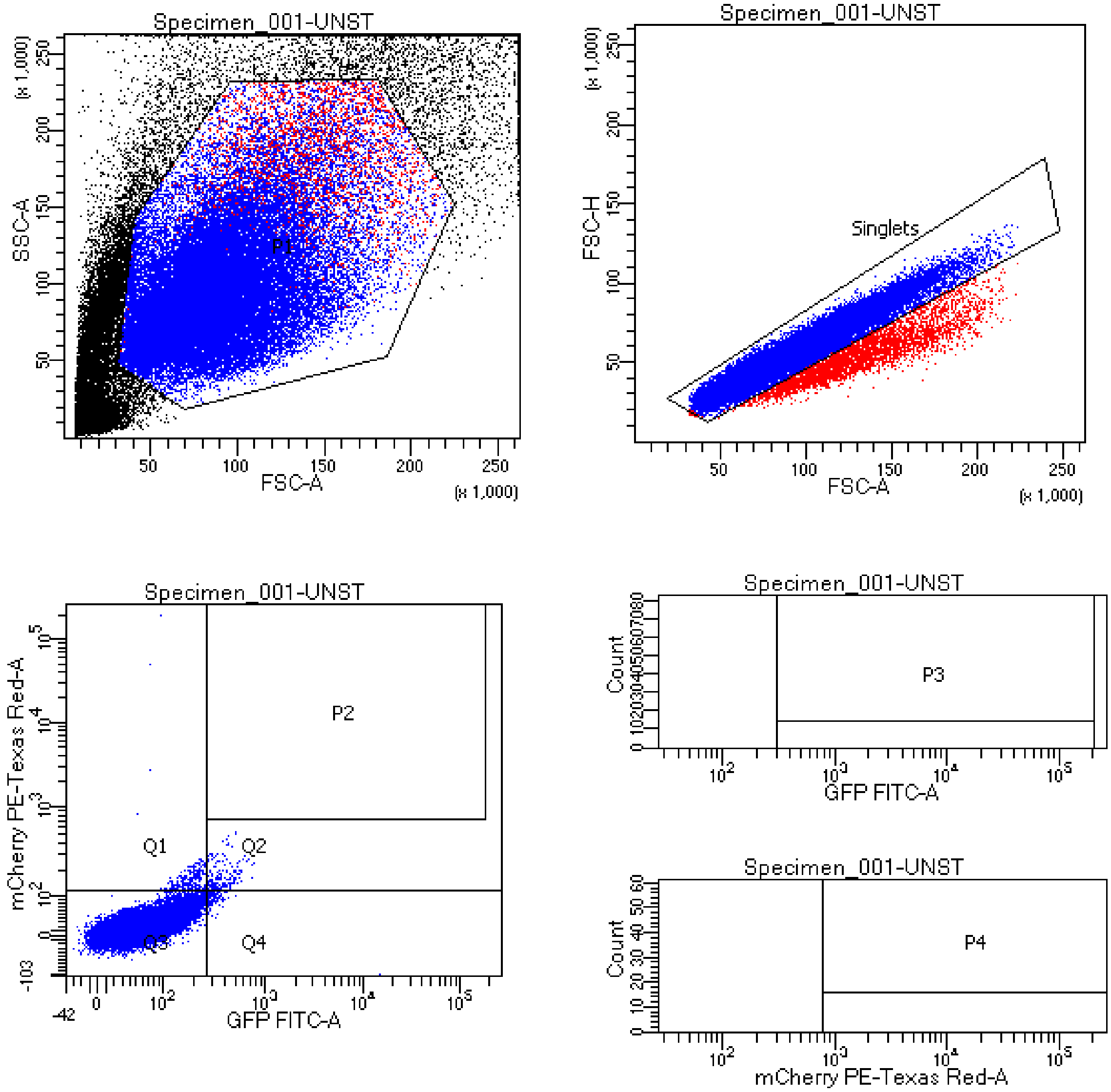

Tube: UNST

Population	#Events	%Parent	%Total
All Events	70,886	####	100.0
P1	50,000	70.5	70.5
Single	45,862	91.7	64.7
P2	0	0.0	0.0
	0	####	0.0
	0	####	0.0
Q1	215	0.5	0.3
Q2	179	0.4	0.3
Q3	45,330	98.8	63.9
Q4	138	0.3	0.2

Experiment Name: Niehoff 303000583 GFP/mCh...
Specimen Name: Specimen_001

Population	#Events	%Parent	GFP FITC-A Mean	mCherry P... Mean
All Events	70,886	####	70	28
P1	50,000	70.5	58	25
Singlets	45,862	91.7	55	24
P2	0	0.0	####	####
P3	0	####	####	####
P4	0	####	####	####
Q1	215	0.5	187	1,358
Q2	179	0.4	383	191
Q3	45,330	98.8	52	17
Q4	138	0.3	405	94

Ctrl guide 1, rep 1

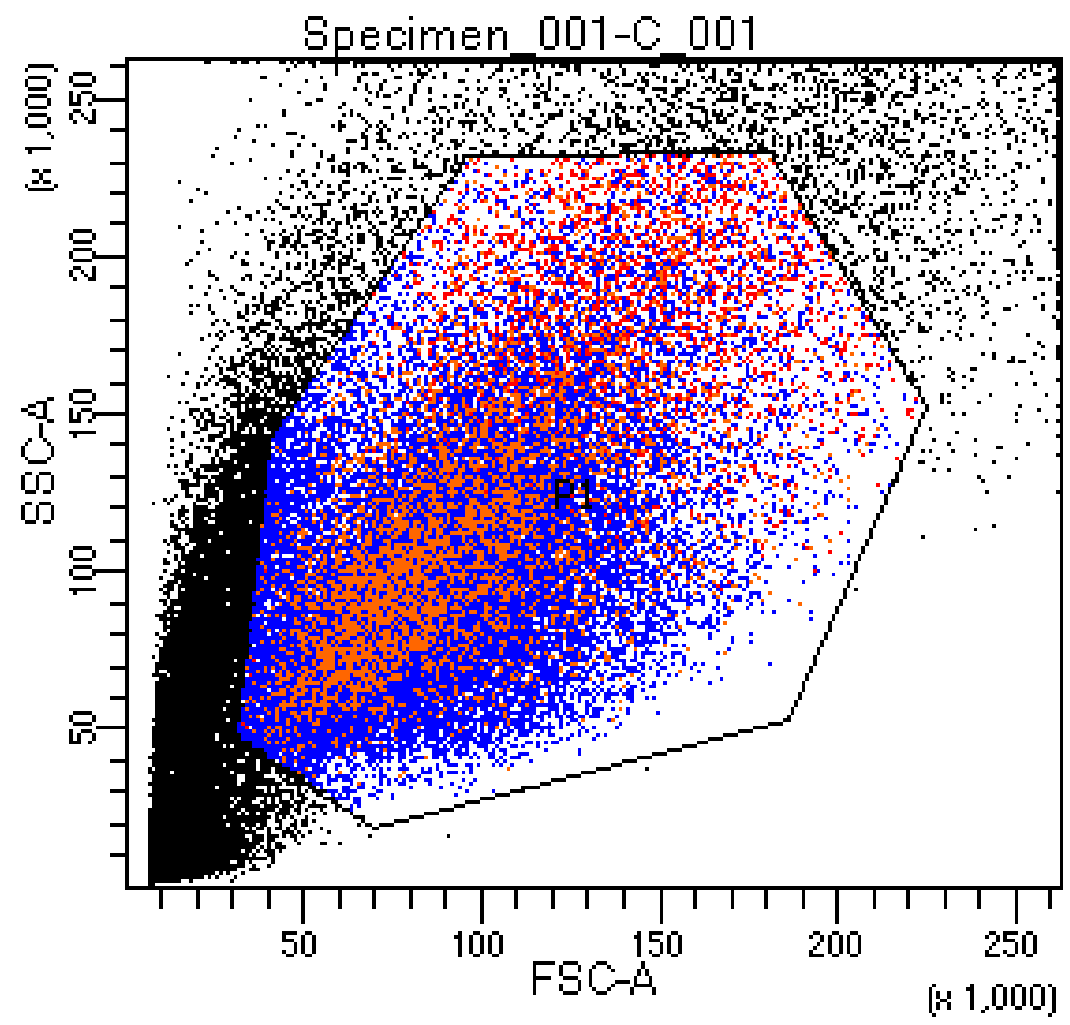

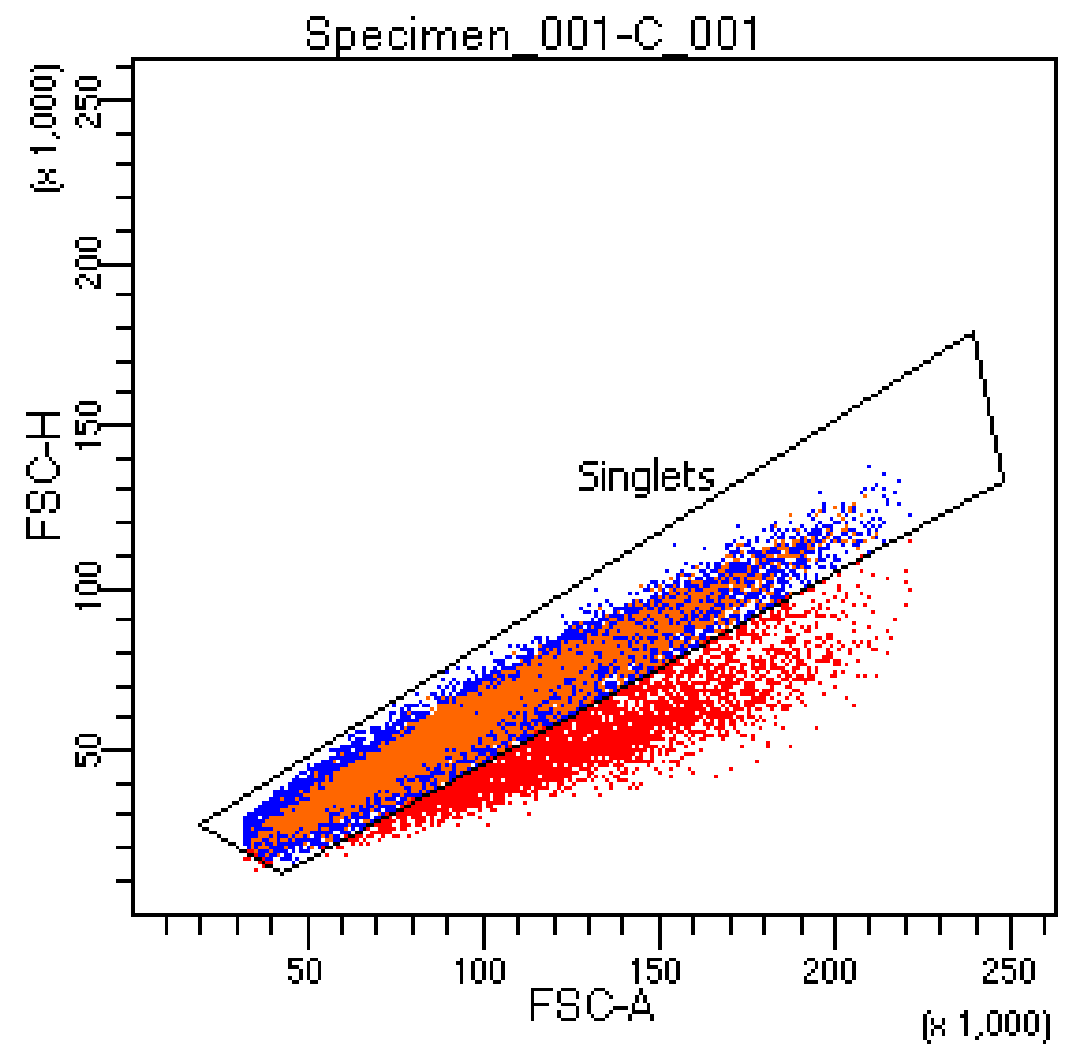

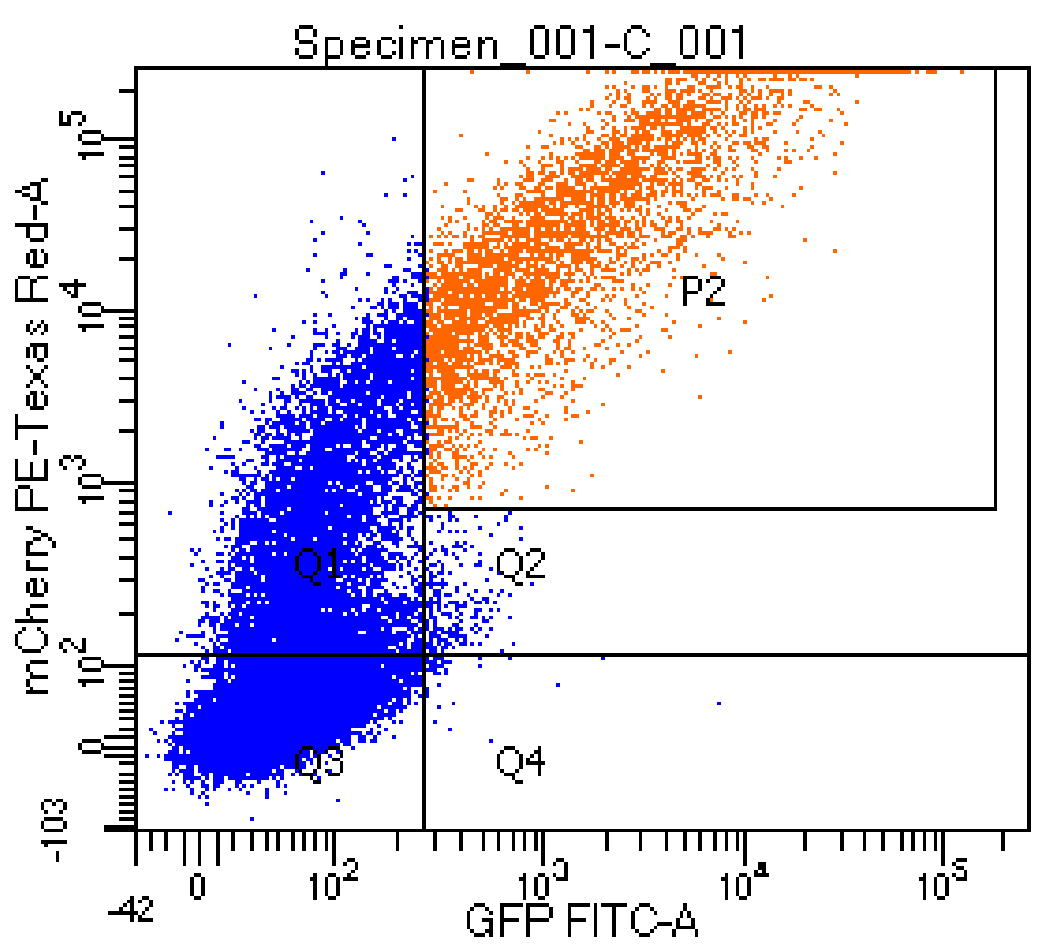

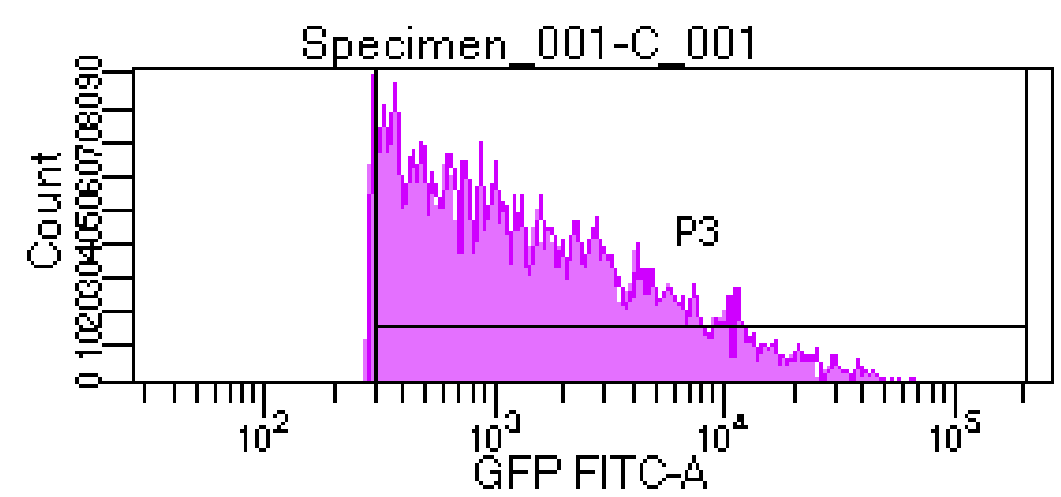

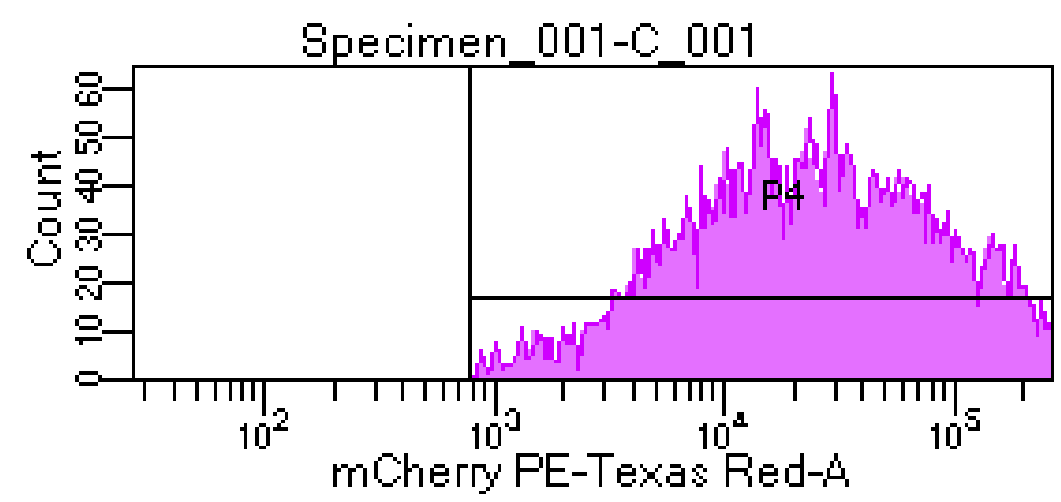

Tube: C_001

Population	#Events	%Parent	%Total
All Events	74,442	####	100.0
P1	50,000	67.2	67.2
Single	46,076	92.2	61.9
P2	4,815	10.5	6.5
	4,572	95.0	6.1
	4,815	100.0	6.5
Q1	8,879	19.3	11.9
Q2	5,121	11.1	6.9
Q3	32,013	69.5	43.0
Q4	63	0.1	0.1

Experiment Name: Niehoff 303000583 GFP/mCh...
Specimen Name: Specimen_001

Population	#Events	%Parent	GFP FITC-A Mean	mCherry P... Mean
All Events	74,442	####	477	6,172
P1	50,000	67.2	436	6,706
Singlets	46,076	92.2	409	6,337
P2	4,815	10.5	3,388	57,598
P3	4,572	95.0	3,553	60,320
P4	4,815	100.0	3,388	57,598
Q1	8,879	19.3	100	1,482
Q2	5,121	11.1	3,206	54,234
Q3	32,013	69.5	47	34
Q4	63	0.1	462	96

Ctrl guide 1, rep 2

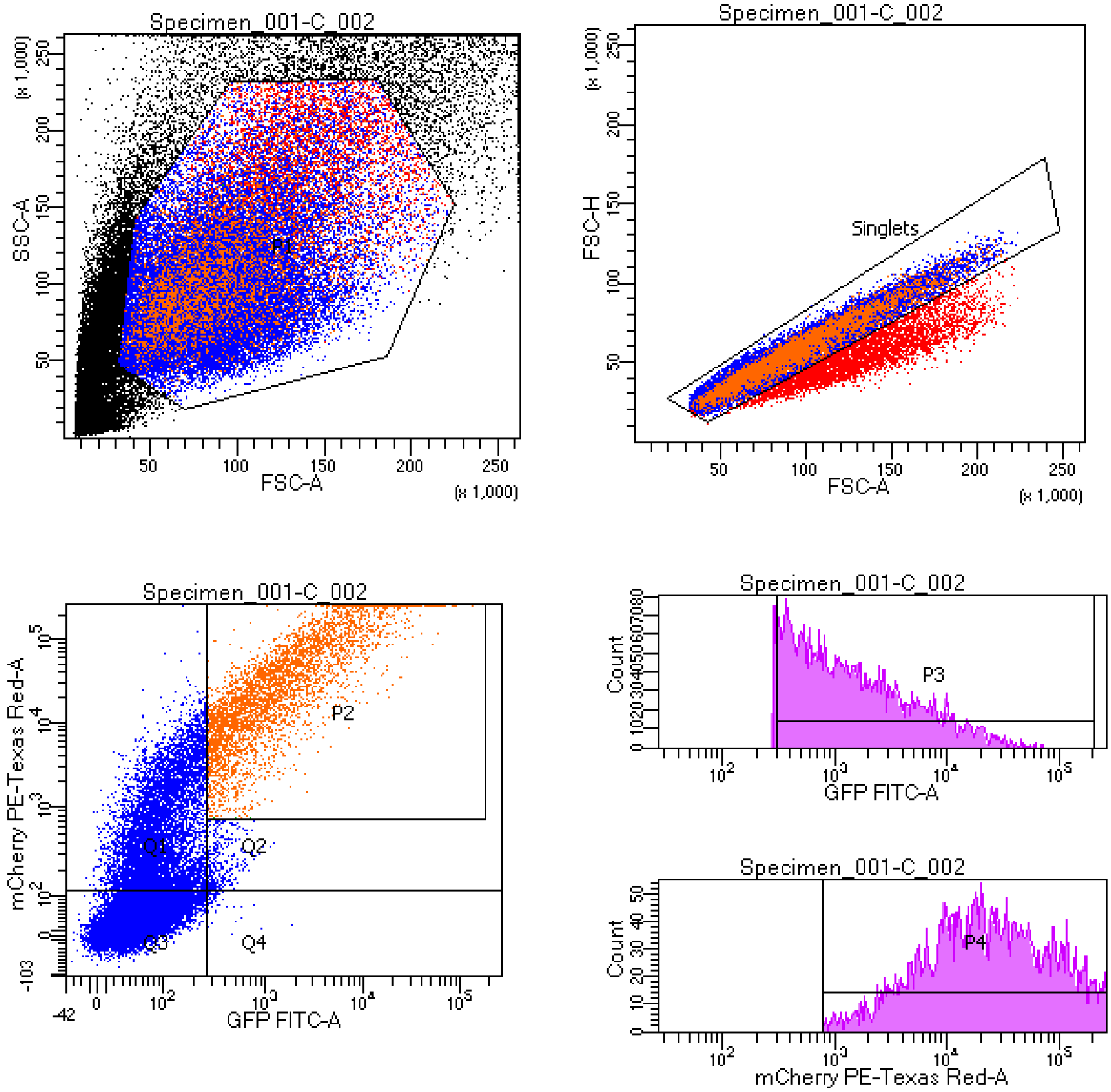

Tube: C_002

Population	#Events	%Parent	%Total
All Events	76,063	####	100.0
P1	50,000	65.7	65.7
Single	44,963	89.9	59.1
P2	4,395	9.8	5.8
	4,160	94.7	5.5
	4,392	99.9	5.8
Q1	8,021	17.8	10.5
Q2	4,763	10.6	6.3
Q3	32,112	71.4	42.2
Q4	67	0.1	0.1

Experiment Name: Niehoff 303000583 GFP/mCh...
Specimen Name: Specimen_001

Population	#Events	%Parent	GFP FITC-A Mean	mCherry P... Mean
All Events	76,063	####	574	6,721
P1	50,000	65.7	441	6,388
Singlets	44,963	89.9	412	5,971
P2	4,395	9.8	3,622	58,134
P3	4,160	94.7	3,811	61,012
P4	4,392	99.9	3,624	58,173
Q1	8,021	17.8	104	1,445
Q2	4,763	10.6	3,371	53,712
Q3	32,112	71.4	50	32
Q4	67	0.1	353	87

Ctrl guide 2

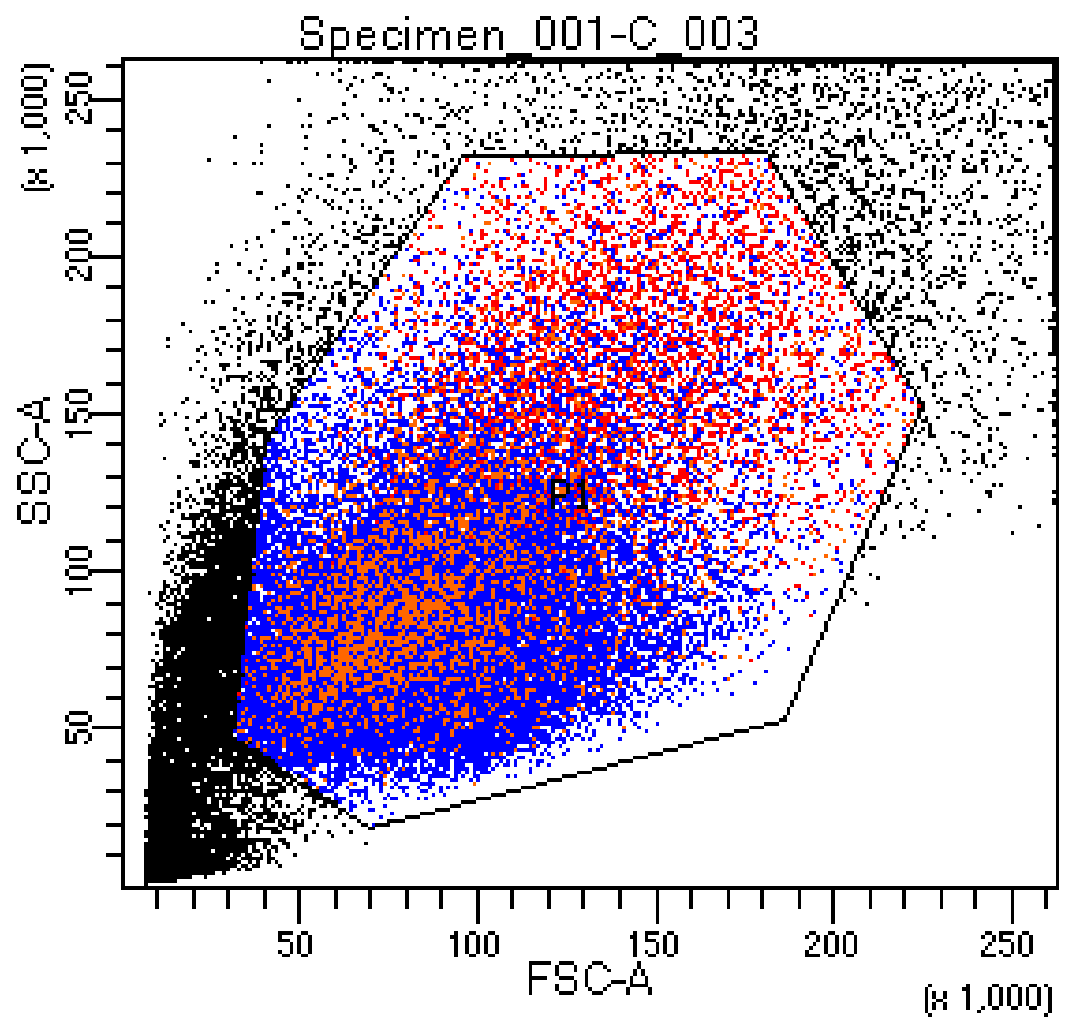

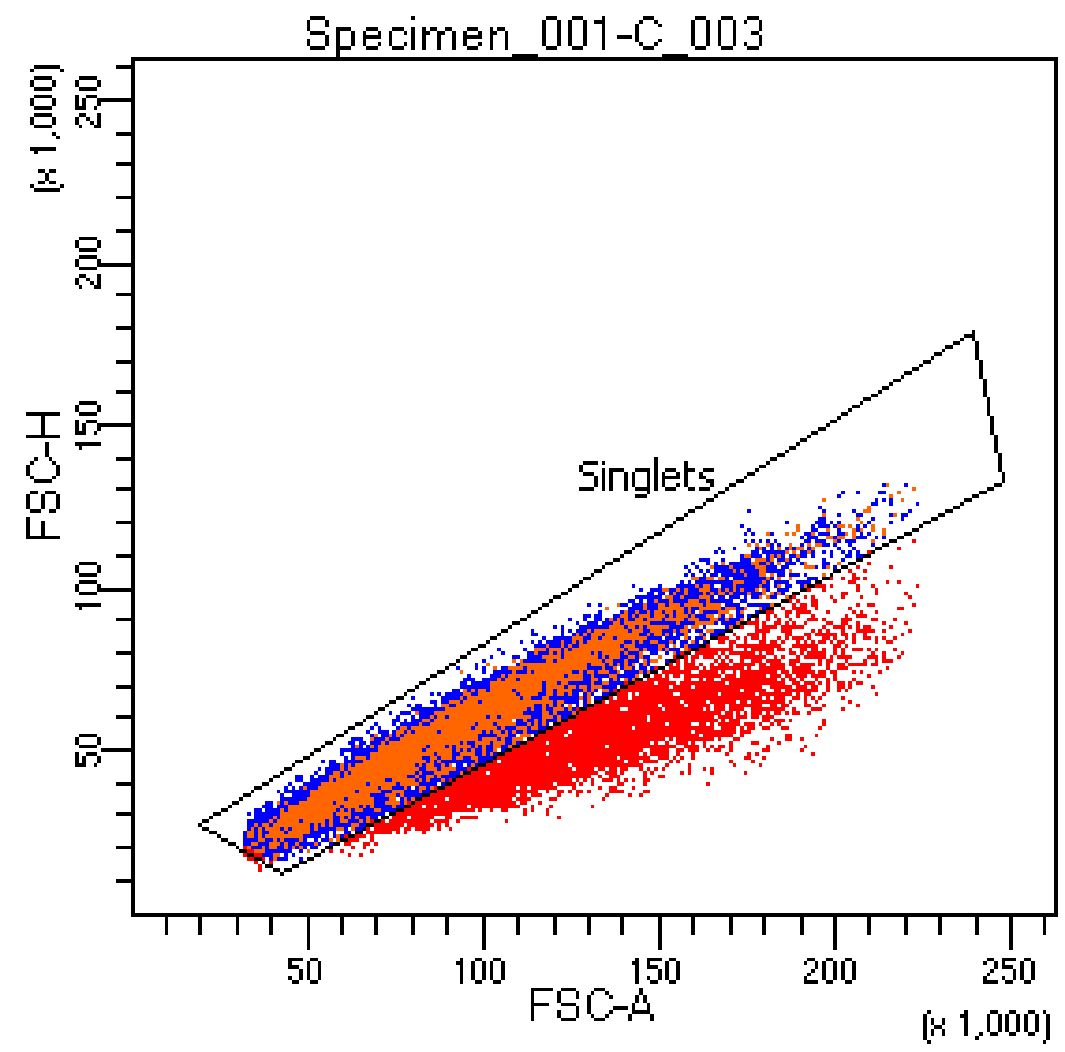

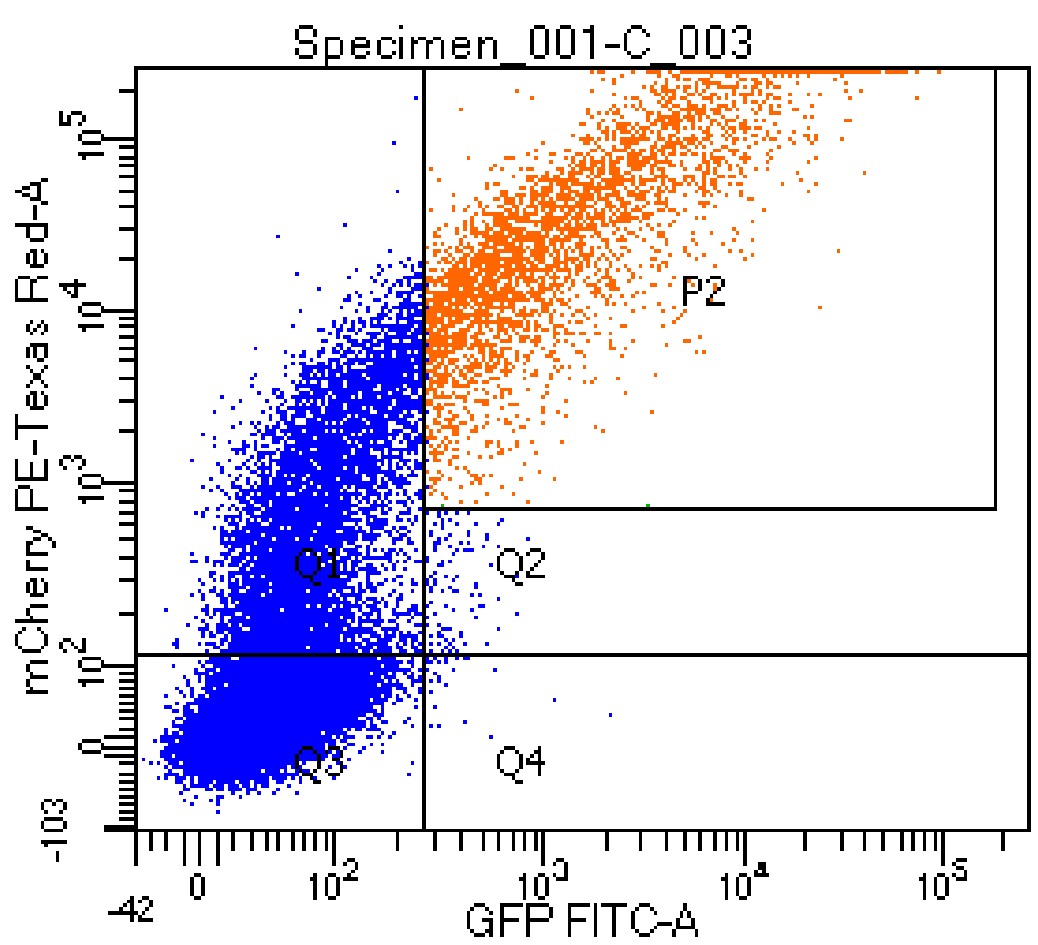

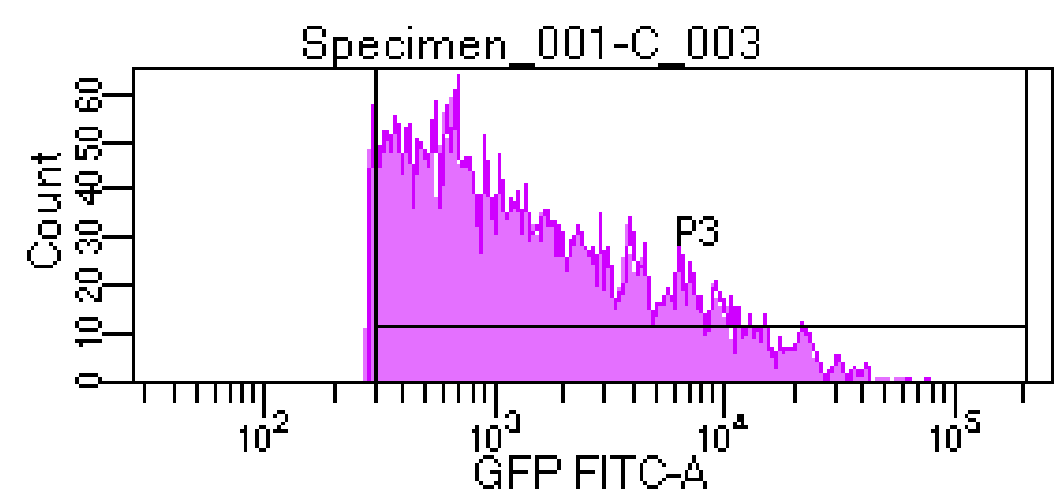

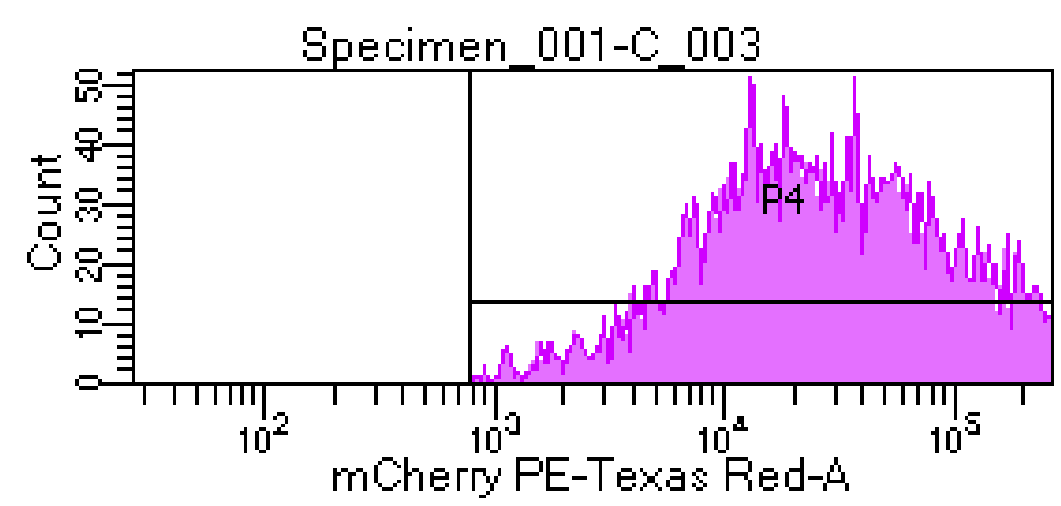

Tube: C_003

Population	#Events	%Parent	%Total
All Events	66,148	####	100.0
P1	50,000	75.6	75.6
Single	44,380	88.8	67.1
P2	3,752	8.5	5.7
	3,580	95.4	5.4
	3,750	99.9	5.7
Q1	7,249	16.3	11.0
Q2	3,892	8.8	5.9
Q3	33,203	74.8	50.2
Q4	36	0.1	0.1

Experiment Name: Niehoff 303000583 GFP/mCh...
Specimen Name: Specimen_001

Population	#Events	%Parent	GFP FITC-A Mean	mCherry P... Mean
All Events	66,148	####	449	5,975
P1	50,000	75.6	361	5,794
Singlets	44,380	88.8	335	5,420
P2	3,752	8.5	3,412	60,812
P3	3,580	95.4	3,562	63,334
P4	3,750	99.9	3,413	60,844
Q1	7,249	16.3	92	1,543
Q2	3,892	8.8	3,302	58,678
Q3	33,203	74.8	40	29
Q4	36	0.1	397	89

ZFC3H1 guide 1, rep 1

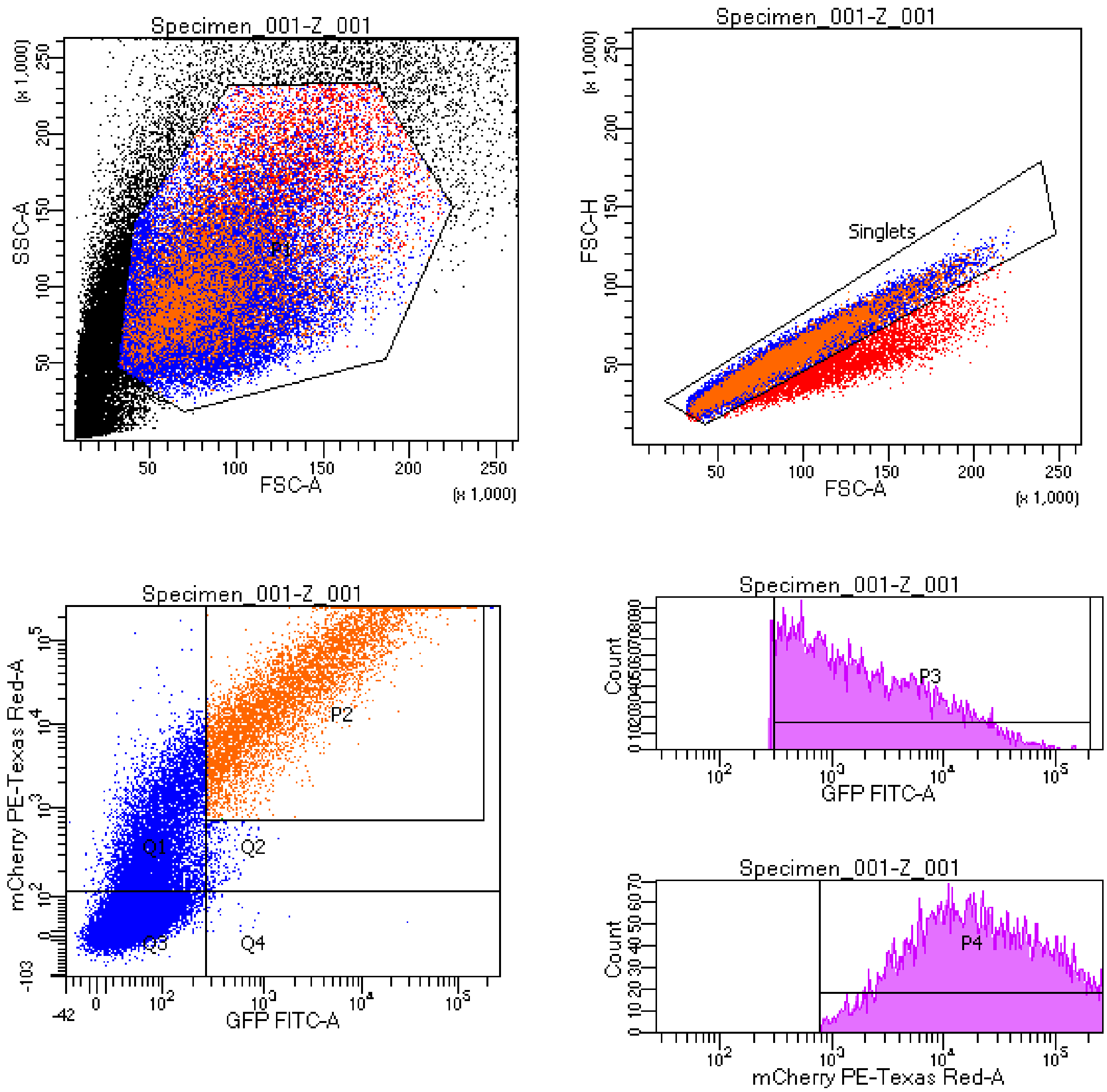

Tube: Z_001

Population	#Events	%Parent	%Total
All Events	73,169	####	100.0
P1	50,000	68.3	68.3
Single	45,541	91.1	62.2
P2	6,489	14.2	8.9
	6,229	96.0	8.5
	6,488	100.0	8.9
Q1	8,704	19.1	11.9
Q2	6,734	14.8	9.2
Q3	30,063	66.0	41.1
Q4	40	0.1	0.1

Experiment Name: Niehoff 303000583 GFP/mCh...
Specimen Name: Specimen_001

Population	#Events	%Parent	GFP FITC-A Mean	mCherry P... Mean
All Events	73,169	####	912	8,130
P1	50,000	68.3	901	8,830
Singlets	45,541	91.1	853	8,385
P2	6,489	14.2	5,545	57,037
P3	6,229	96.0	5,765	59,177
P4	6,488	100.0	5,546	57,045
Q1	8,704	19.1	102	1,132
Q2	6,734	14.8	5,422	55,077
Q3	30,063	66.0	46	37
Q4	40	0.1	1,142	77

ZFC3H1 guide 1, rep 2

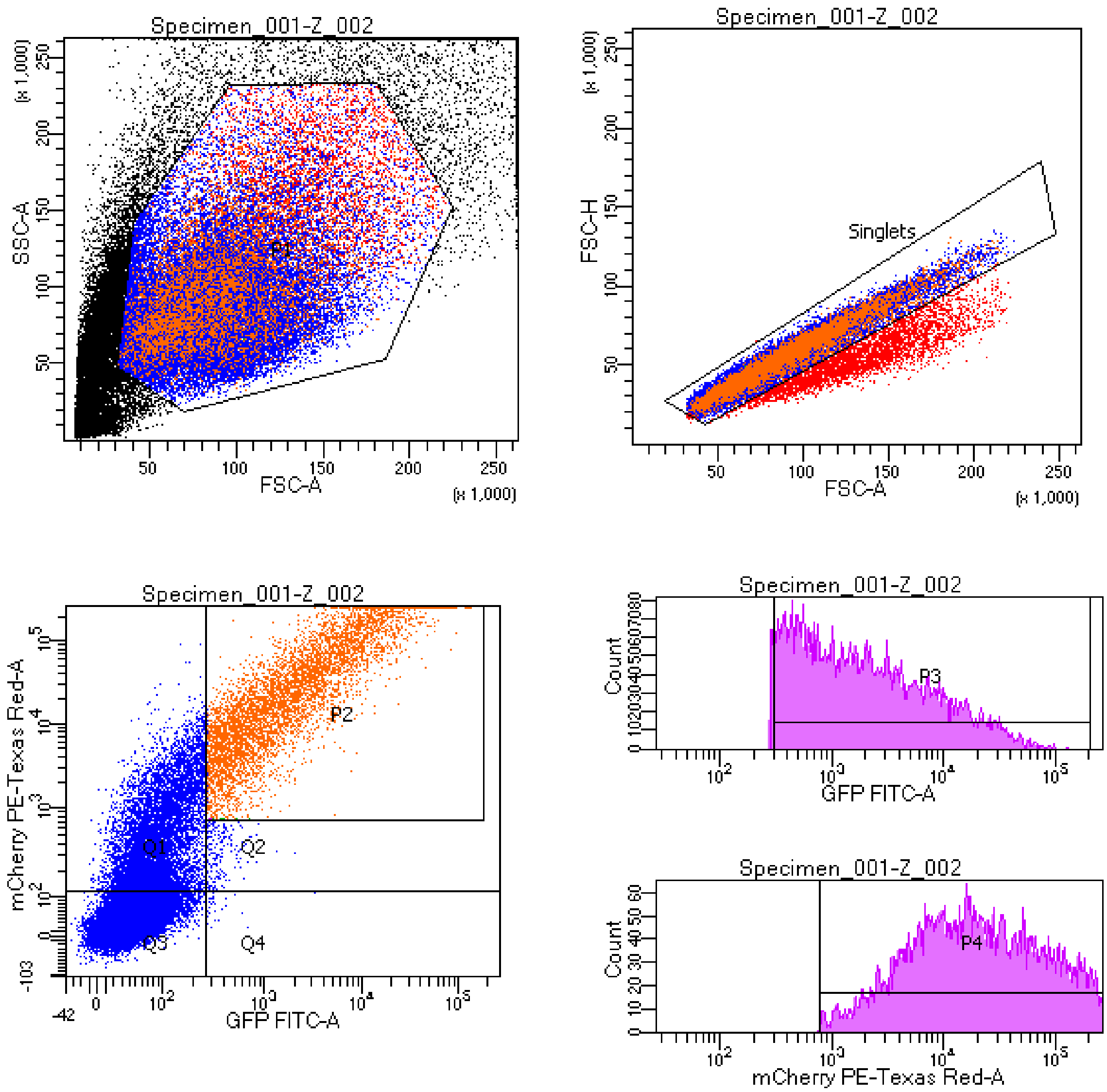

Tube: Z_002

Population	#Events	%Parent	%Total
All Events	72,346	####	100.0
P1	50,000	69.1	69.1
Single	46,067	92.1	63.7
P2	5,684	12.3	7.9
	5,473	96.3	7.6
	5,675	99.8	7.8
Q1	8,293	18.0	11.5
Q2	5,905	12.8	8.2
Q3	31,839	69.1	44.0
Q4	30	0.1	0.0

Experiment Name: Niehoff 303000583 GFP/mCh...
Specimen Name: Specimen_001

Population	#Events	%Parent	GFP FITC-A Mean	mCherry P... Mean
All Events	72,346	####	738	6,652
P1	50,000	69.1	753	7,590
Singlets	46,067	92.1	707	7,084
P2	5,684	12.3	5,331	55,571
P3	5,473	96.3	5,526	57,539
P4	5,675	99.8	5,339	55,658
Q1	8,293	18.0	99	1,085
Q2	5,905	12.8	5,145	53,537
Q3	31,839	69.1	43	38
Q4	30	0.1	432	77

ZFC3H1 guide 2

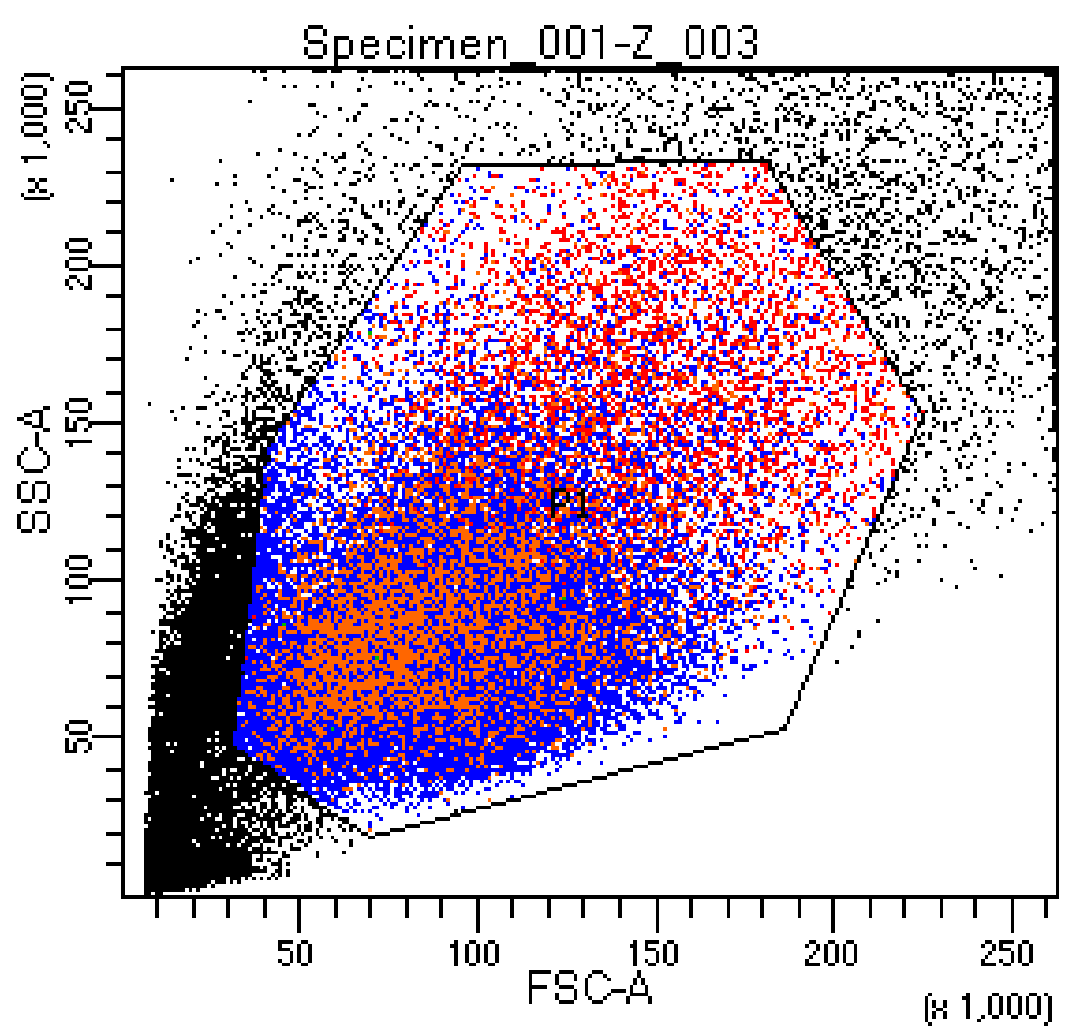

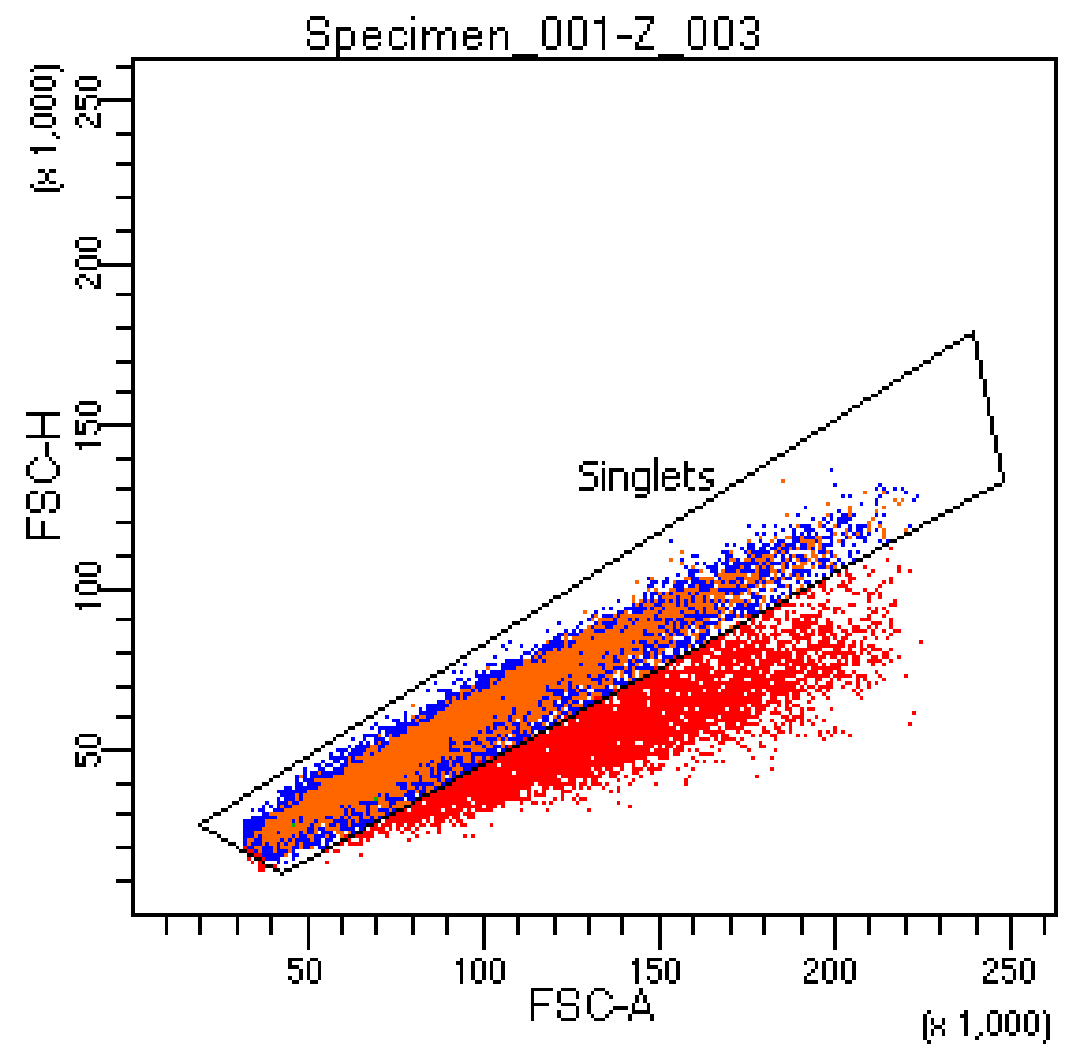

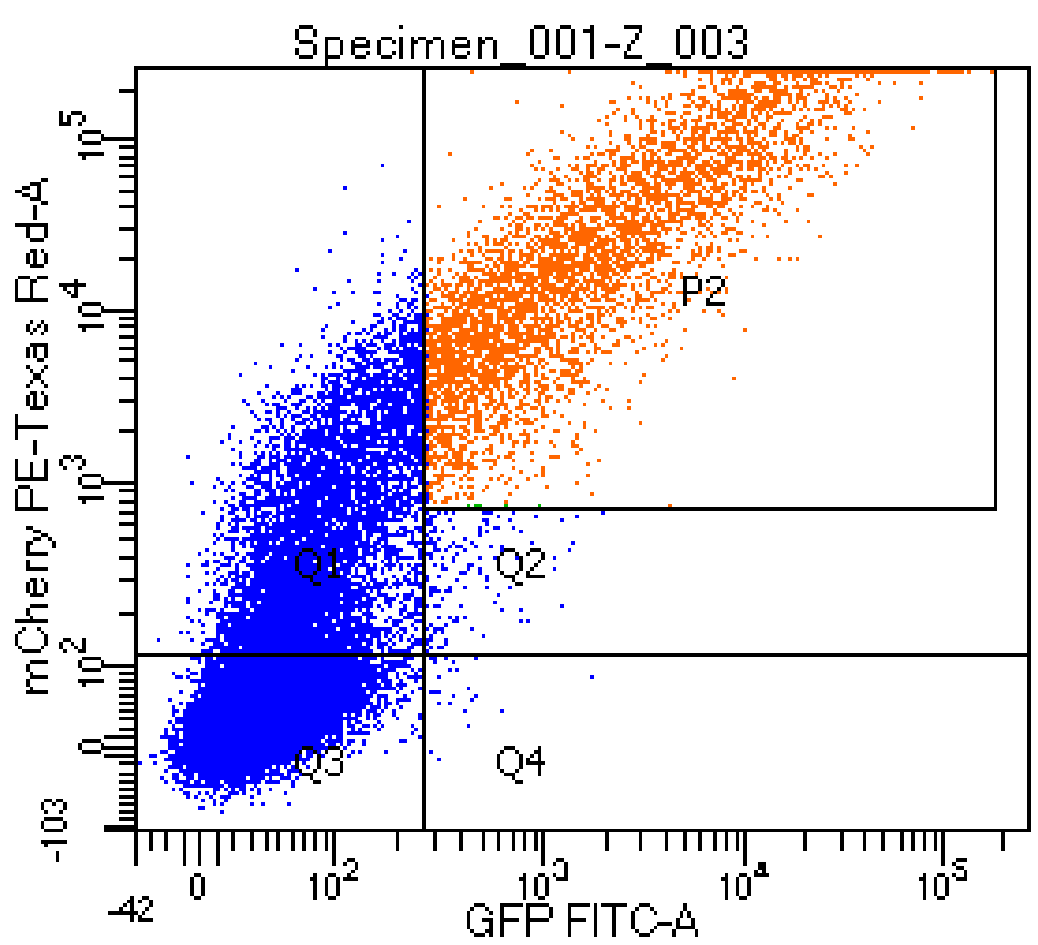

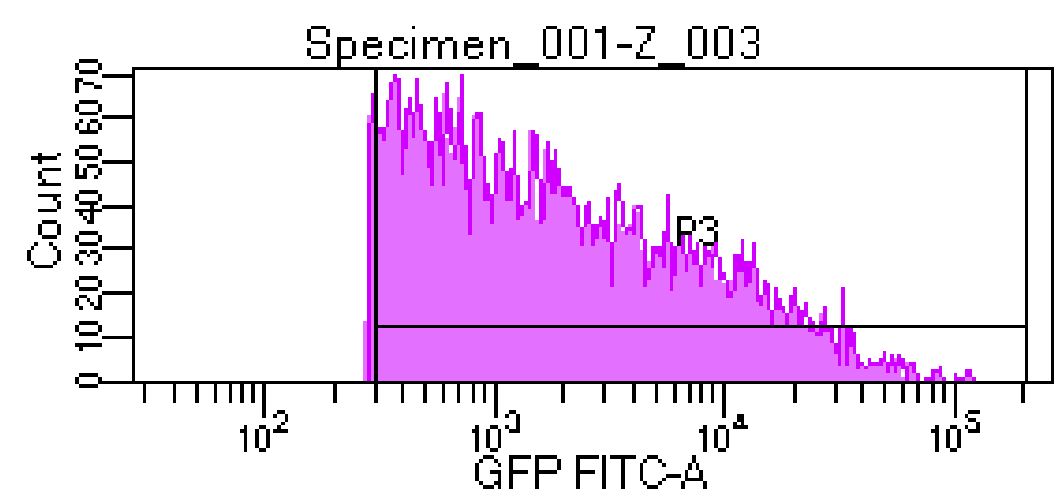

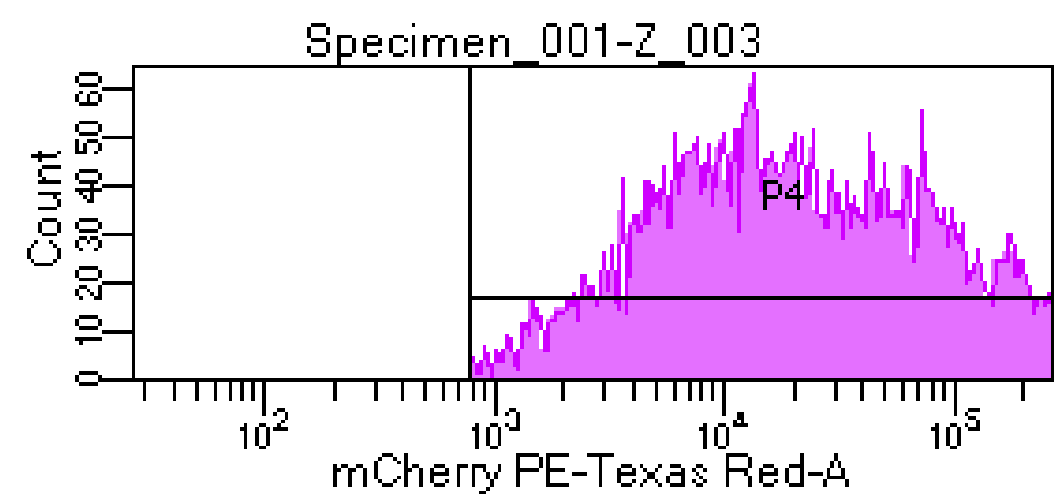

Tube: Z_003

Population	#Events	%Parent	%Total
All Events	70,387	####	100.0
P1	50,000	71.0	71.0
Single	43,928	87.9	62.4
P2	5,256	12.0	7.5
	5,046	96.0	7.2
	5,251	99.9	7.5
Q1	7,671	17.5	10.9
Q2	5,456	12.4	7.8
Q3	30,774	70.1	43.7
Q4	27	0.1	0.0

Experiment Name: Niehoff 303000583 GFP/mCh...
Specimen Name: Specimen_001

Population	#Events	%Parent	GFP FITC-A Mean	mCherry P... Mean
All Events	70,387	####	751	6,740
P1	50,000	71.0	729	7,088
Singlets	43,928	87.9	690	6,676
P2	5,256	12.0	5,349	53,835
P3	5,046	96.0	5,559	55,866
P4	5,251	99.9	5,353	53,886
Q1	7,671	17.5	95	1,141
Q2	5,456	12.4	5,202	51,948
Q3	30,774	70.1	38	35
Q4	27	0.1	405	72

ZFC3H1 guide 1, rep 1
+ ZFC3H1 cDNA

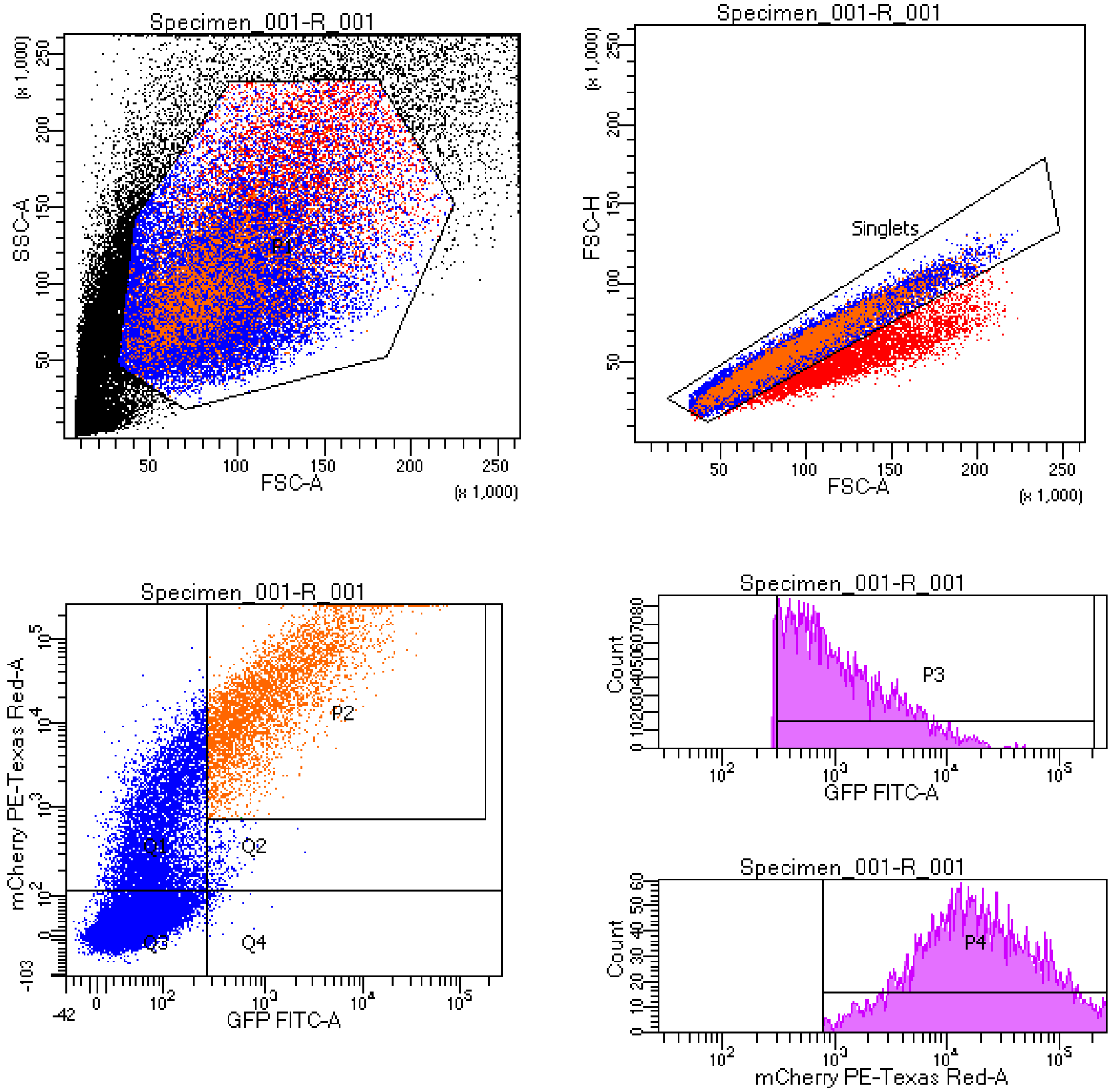

Tube: R_001

Population	#Events	%Parent	%Total
All Events	74,085	####	100.0
P1	50,000	67.5	67.5
Single	44,731	89.5	60.4
P2	4,579	10.2	6.2
	4,321	94.4	5.8
	4,578	100.0	6.2
Q1	7,004	15.7	9.5
Q2	4,817	10.8	6.5
Q3	32,850	73.4	44.3
Q4	60	0.1	0.1

Experiment Name: Niehoff 303000583 GFP/mCh...
Specimen Name: Specimen_001

Population	#Events	%Parent	GFP FITC-A Mean	mCherry P... Mean
All Events	74,085	####	350	4,943
P1	50,000	67.5	303	4,868
Singlets	44,731	89.5	286	4,604
P2	4,579	10.2	2,270	42,278
P3	4,321	94.4	2,389	44,416
P4	4,578	100.0	2,271	42,287
Q1	7,004	15.7	105	1,592
Q2	4,817	10.8	2,177	40,250
Q3	32,850	73.4	47	28
Q4	60	0.1	336	82

ZFC3H1 guide 1, rep 2
+ ZFC3H1 cDNA

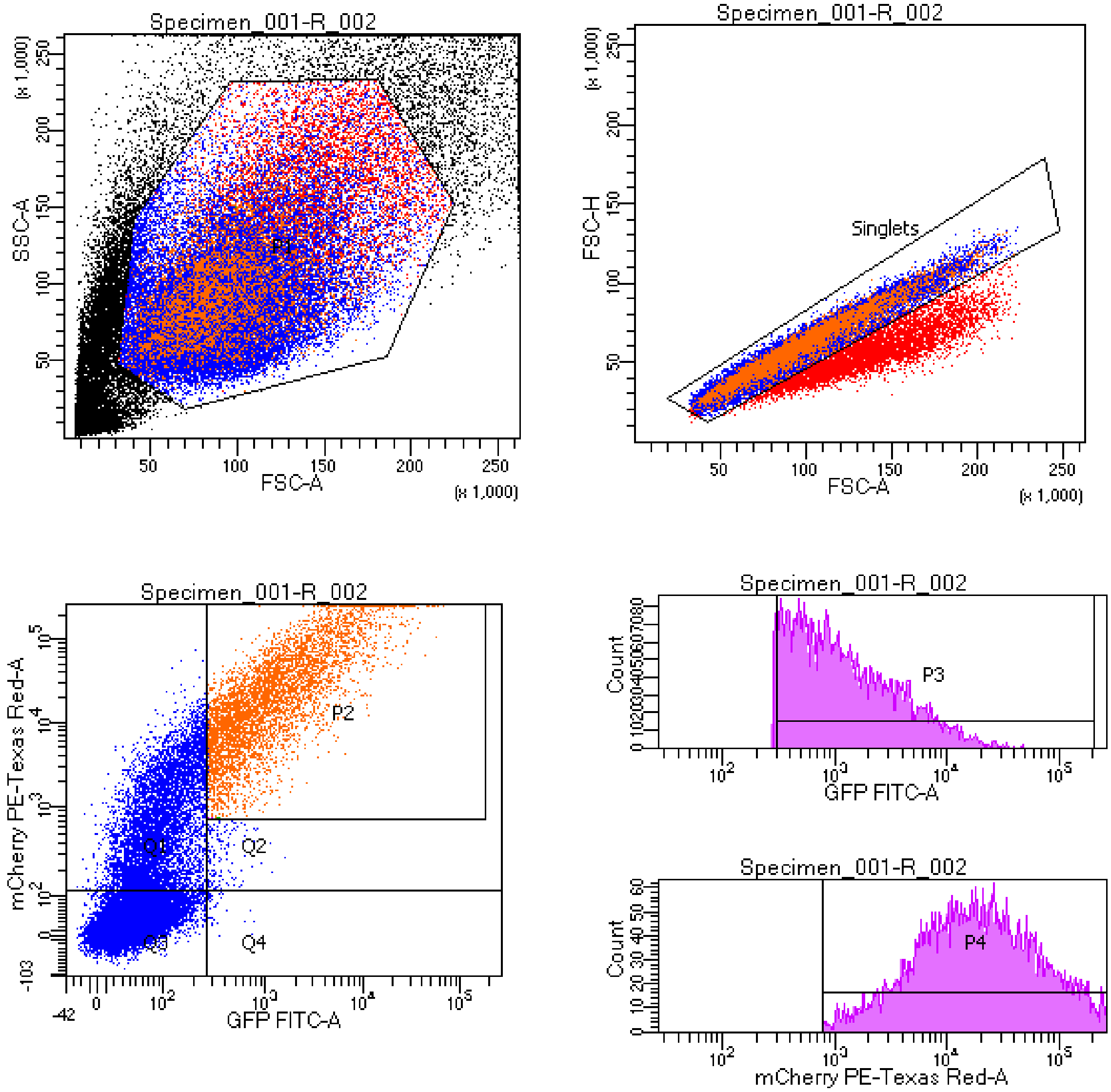

Tube: R_002

Population	#Events	%Parent	%Total
All Events	71,530	####	100.0
P1	50,000	69.9	69.9
Single	44,027	88.1	61.6
P2	4,924	11.2	6.9
	4,698	95.4	6.6
	4,918	99.9	6.9
Q1	6,786	15.4	9.5
Q2	5,108	11.6	7.1
Q3	32,091	72.9	44.9
Q4	42	0.1	0.1

Experiment Name: Niehoff 303000583 GFP/mCh...
Specimen Name: Specimen_001

Population	#Events	%Parent	GFP FITC-A Mean	mCherry P... Mean
All Events	71,530	####	400	5,559
P1	50,000	69.9	349	5,484
Singlets	44,027	88.1	320	5,046
P2	4,924	11.2	2,430	42,664
P3	4,698	95.4	2,533	44,400
P4	4,918	99.9	2,433	42,715
Q1	6,786	15.4	103	1,601
Q2	5,108	11.6	2,356	41,183
Q3	32,091	72.9	42	29
Q4	42	0.1	335	76

ZFC3H1 guide 2
+ ZFC3H1 cDNA

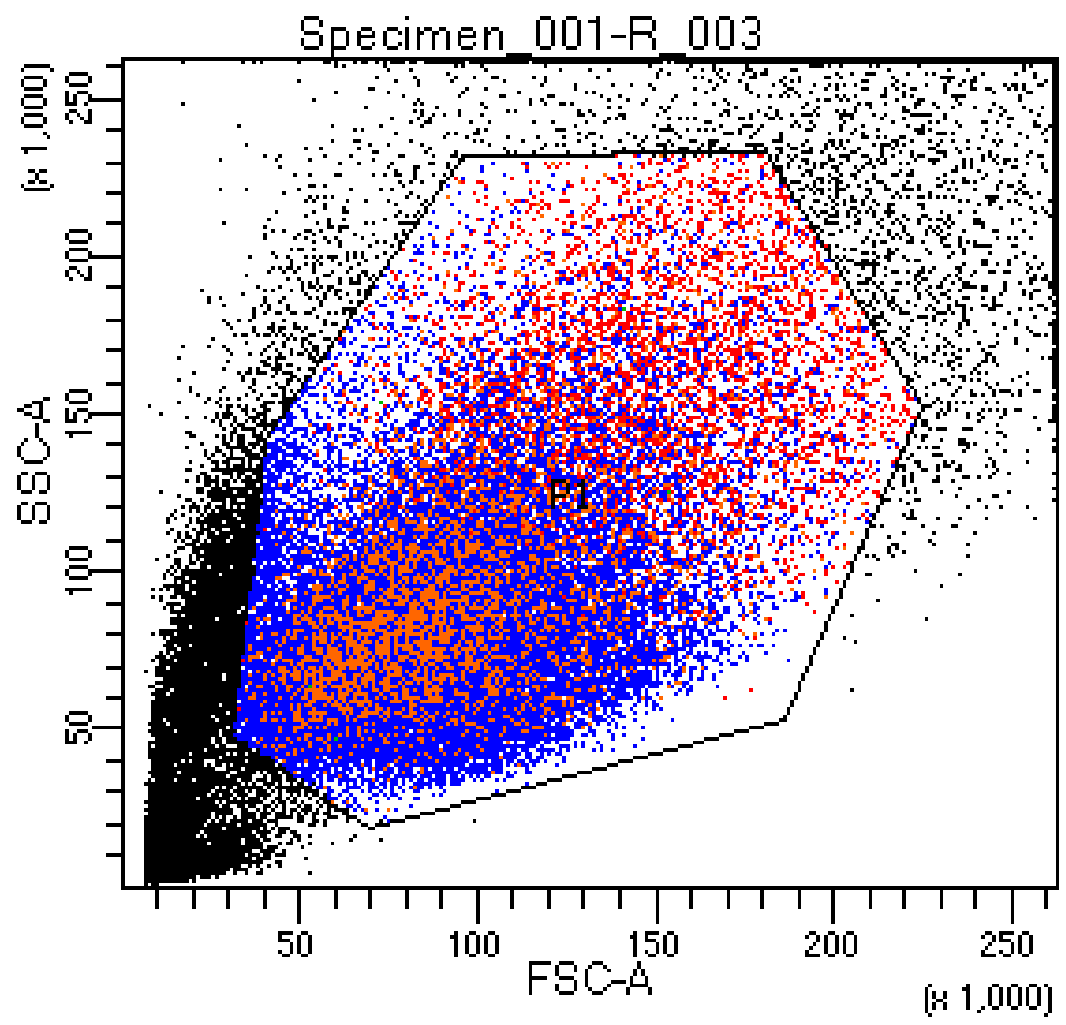

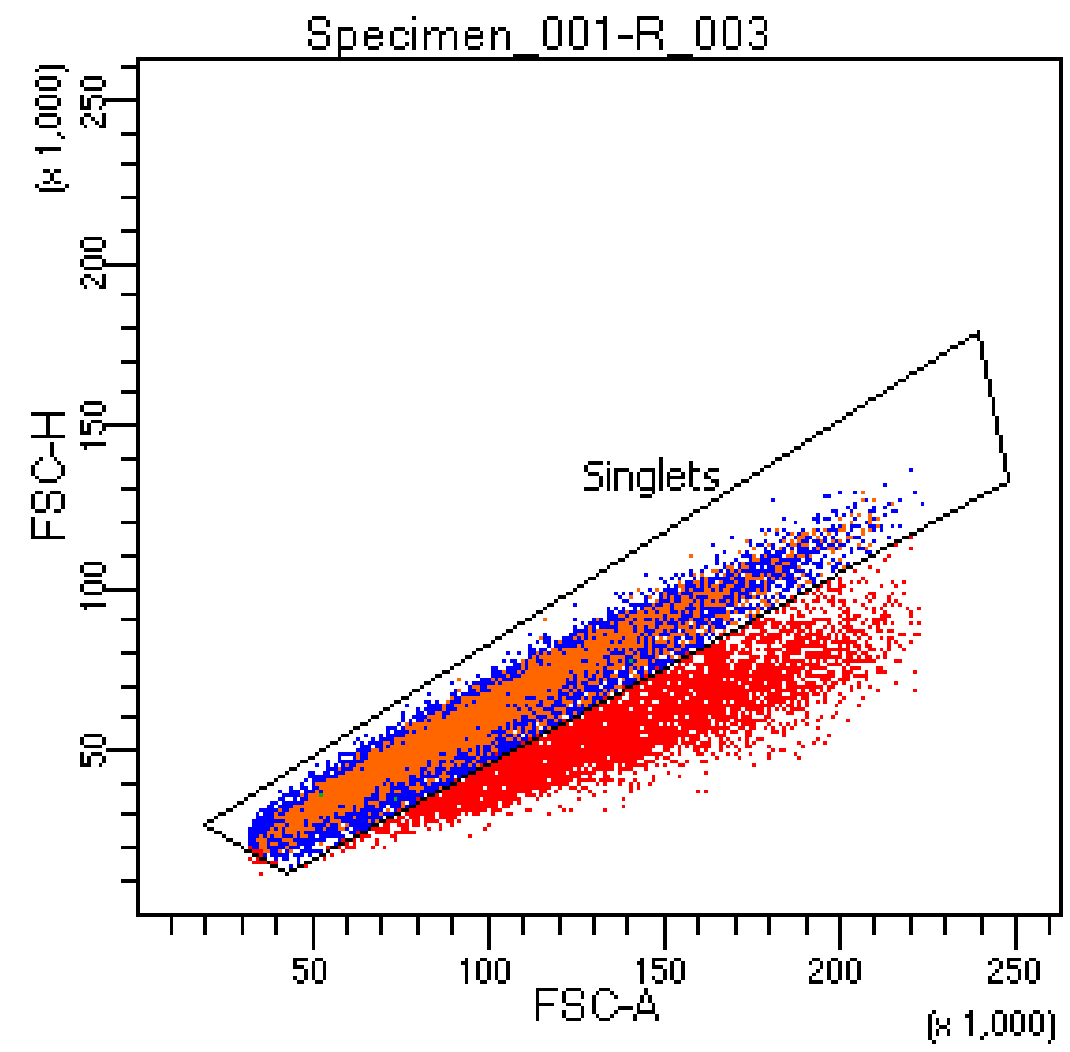

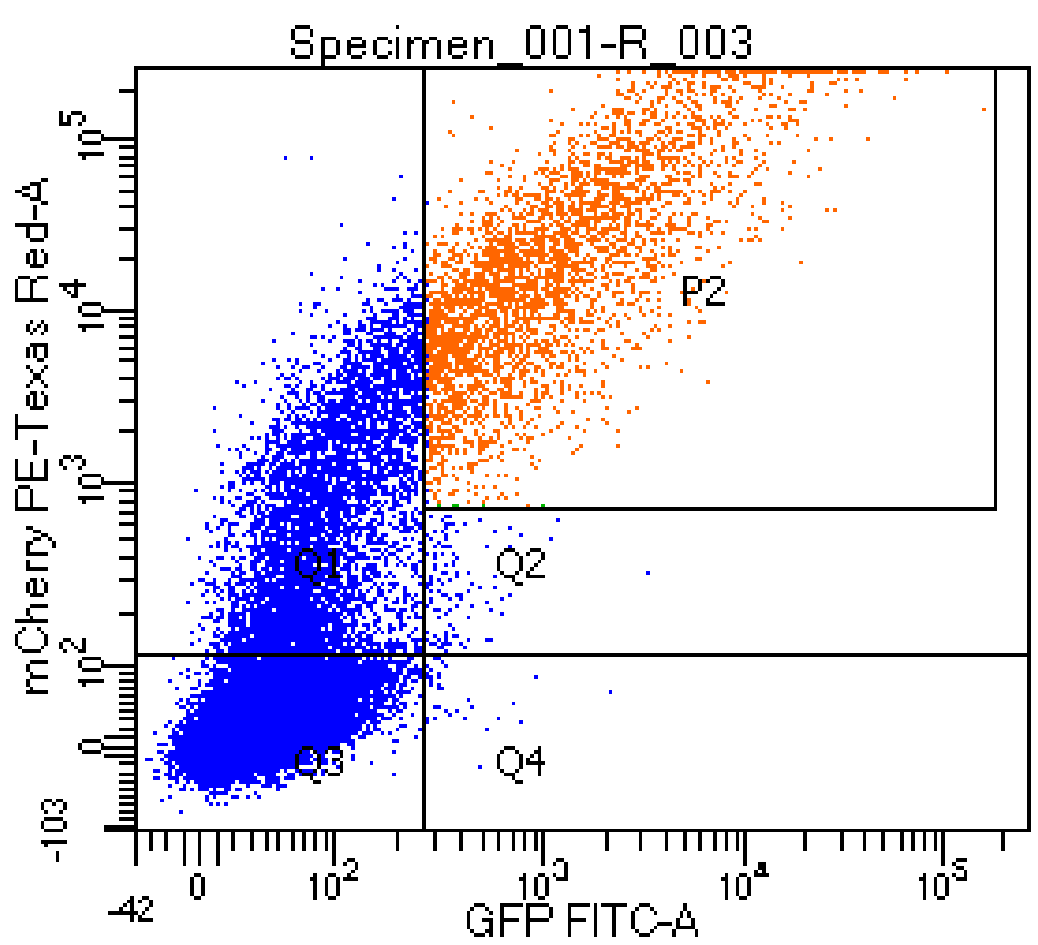

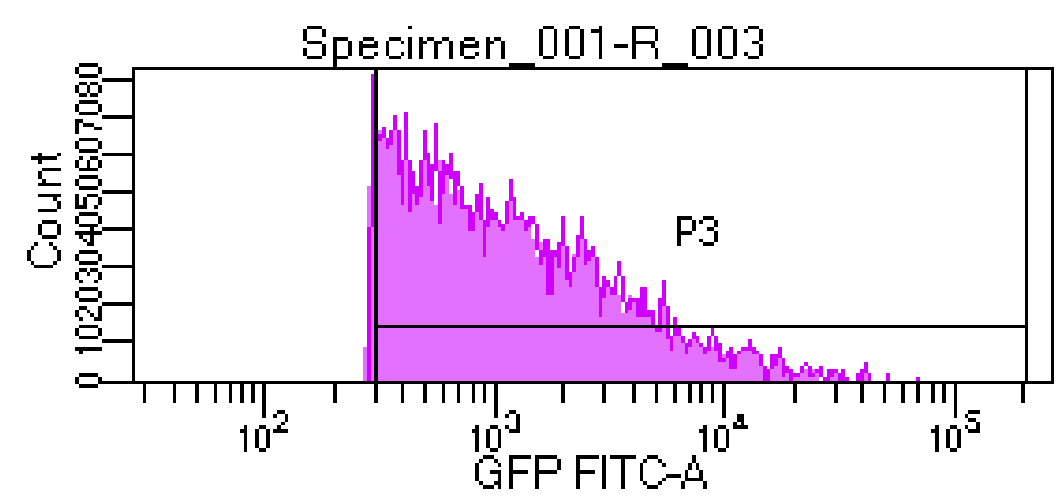

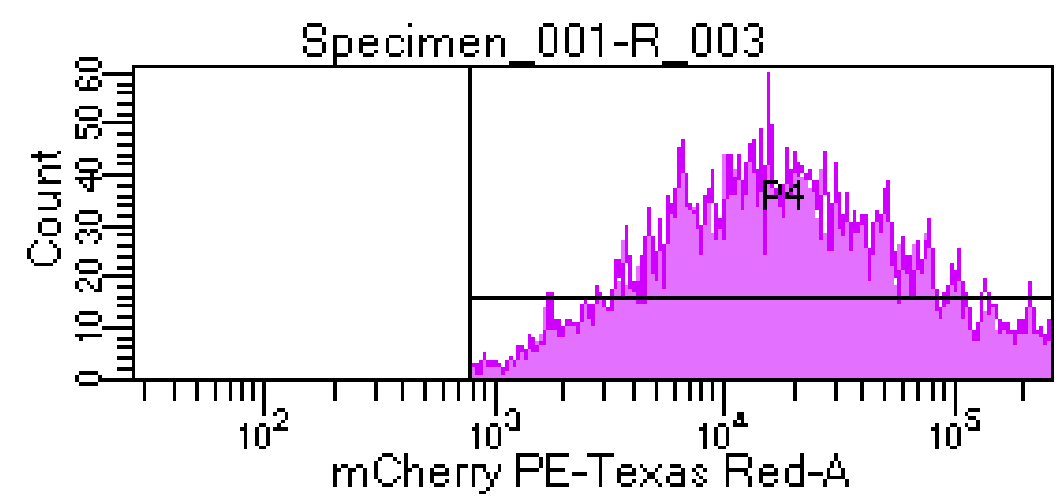

Tube: R_003

Population	#Events	%Parent	%Total
All Events	68,750	####	100.0
P1	50,000	72.7	72.7
Single	44,417	88.8	64.6
P2	3,887	8.8	5.7
	3,667	94.3	5.3
	3,882	99.9	5.6
Q1	5,892	13.3	8.6
Q2	4,047	9.1	5.9
Q3	34,440	77.5	50.1
Q4	38	0.1	0.1

Experiment Name: Niehoff 303000583 GFP/mCh...
Specimen Name: Specimen_001

Population	#Events	%Parent	GFP FITC-A Mean	mCherry P... Mean
All Events	68,750	####	311	4,133
P1	50,000	72.7	292	4,211
Singlets	44,417	88.8	271	3,876
P2	3,887	8.8	2,573	41,607
P3	3,667	94.3	2,710	43,718
P4	3,882	99.9	2,576	41,659
Q1	5,892	13.3	99	1,577
Q2	4,047	9.1	2,486	40,024
Q3	34,440	77.5	40	25
Q4	38	0.1	406	65
