# Supplementary material for: The PAXT connection is a modifier of rare codon-enriched protein expression
Source: Nat Commun. 2026 Sep 17;17:10928. doi: 10.1038/s41467-026-77792-7 (PMC13680609; doi:10.1038/s41467-026-77792-7)
Supplement: Supplementary file 6 [file 41467_2026_77792_MOESM7_ESM.pdf]

Uncropped Immunoblots – Niehoff *et al*

Substrate: BioRad Clarity and Clarity Max Western ECL Substrate

Protein Ladder: BioRad Precision Plus Protein Standards or Pageruler Ladder

Imager: BioRad ChemiDoc XRS+/GelDoc XR+

Accumulation Mode Settings:

First Image: 1 sec
Last Image: 60 sec
# of Images: 20

Loading controls (B-tubulin or GAPDH) for each blot included on same page (or it is specified which blot it corresponds to)

Figure 2A

ZFC3H1 (10.3 sec exposure – accumulation mode, max substrate)

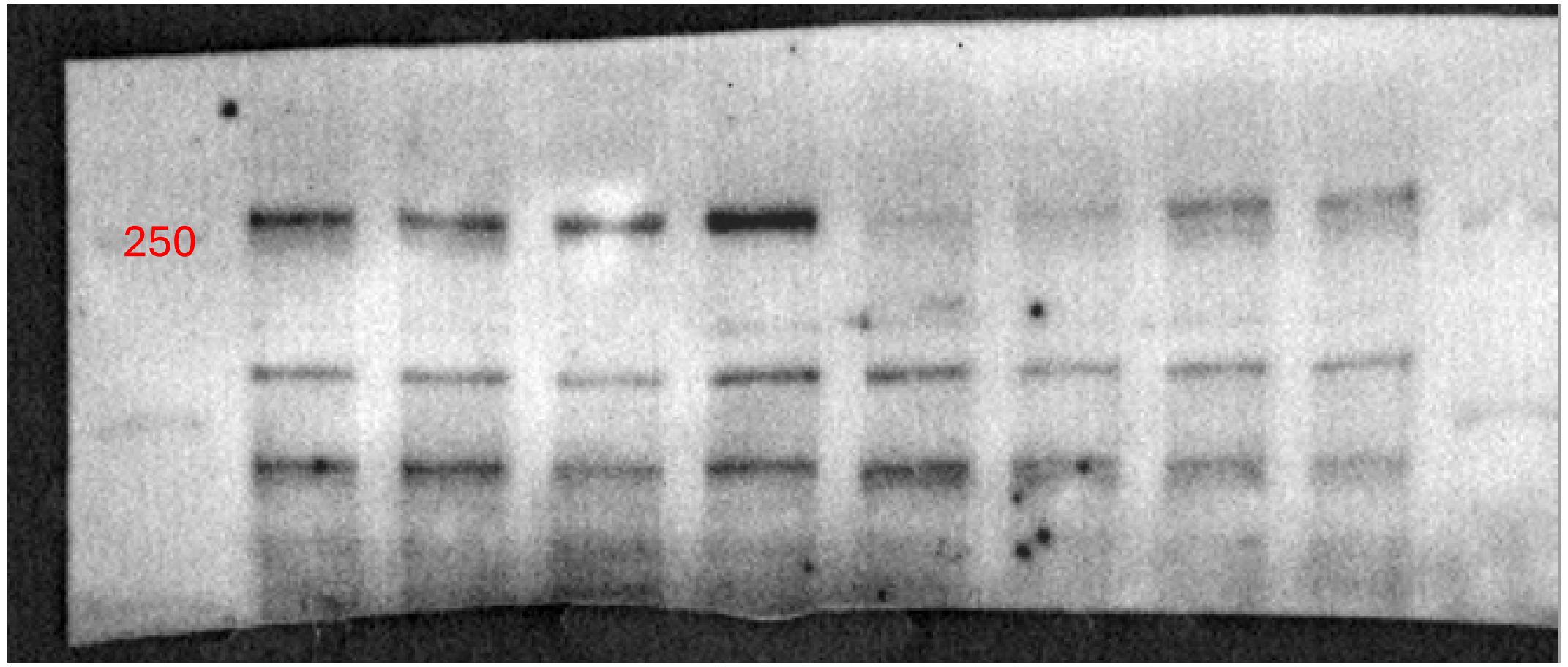

B-Tubulin for ZFC3H1 (1 sec exposure – accumulation mode)

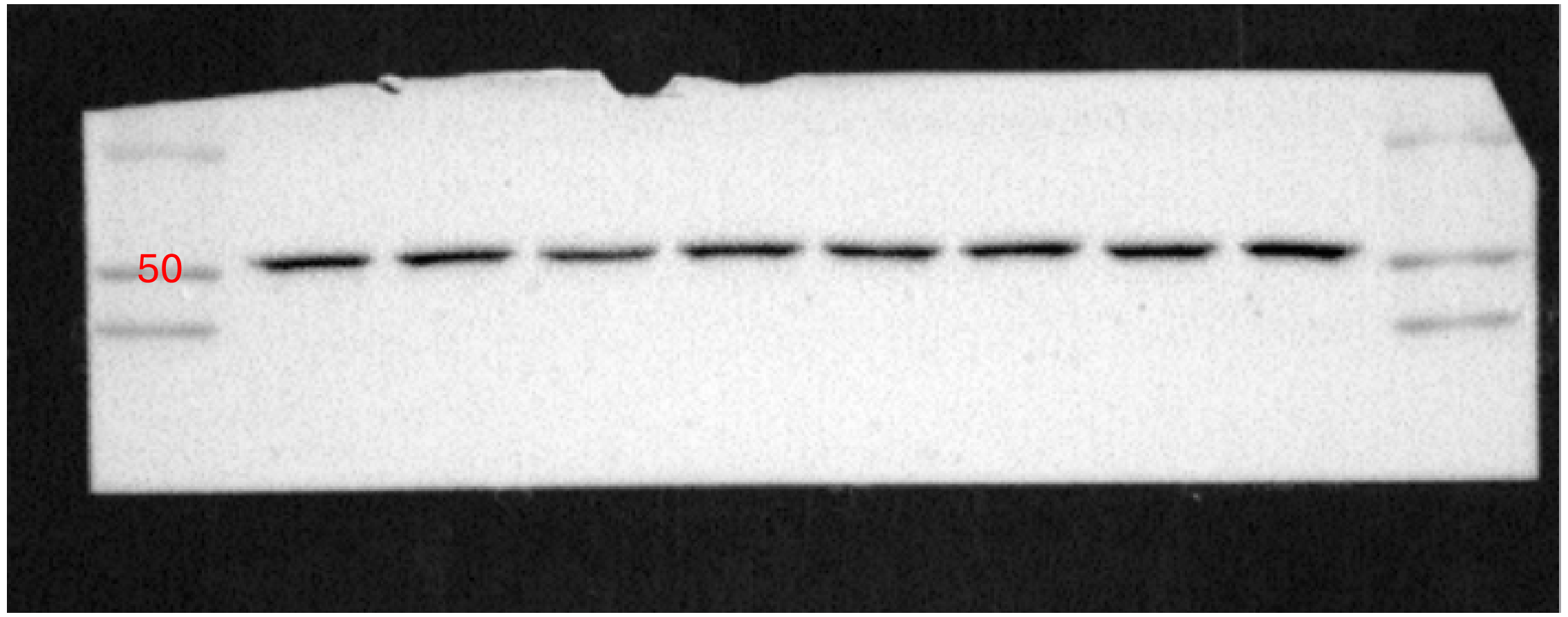

Lane	Sample
1	Ladder
2	Ctrl guide 1, Empty Vector
3	Ctrl guide 2, Empty Vector
4	Ctrl guide 1, FLAG-ZFC3H1
5	Ctrl guide 2, FLAG-ZFC3H1
6	ZFC3H1 guide 1, Empty Vector
7	ZFC3H1 guide 2, Empty Vector
8	ZFC3H1 guide 1, FLAG-ZFC3H1
9	ZFC3H1 guide 2, FLAG-ZFC3H1
10	Ladder

Figure 2A

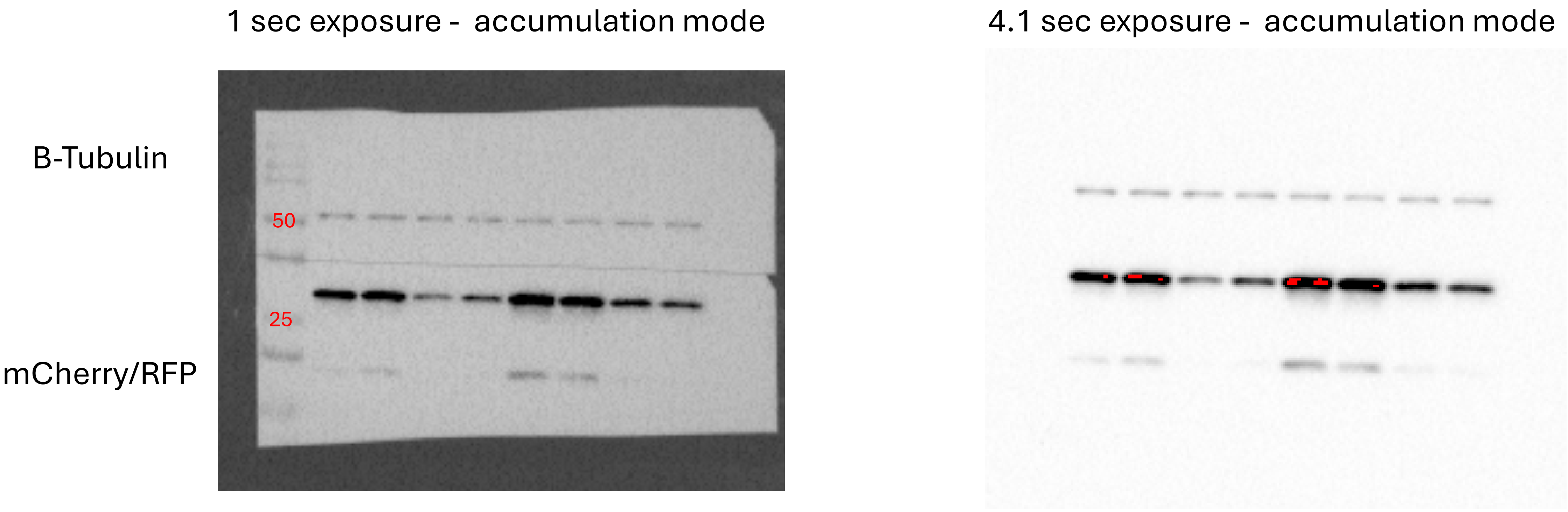
1 sec exposure - accumulation mode
4.1 sec exposure - accumulation mode
B-Tubulin
mCherry/RFP
50
25

Figure 2A

B-Tubulin for GFP (4.1 sec exposure – accumulation mode)

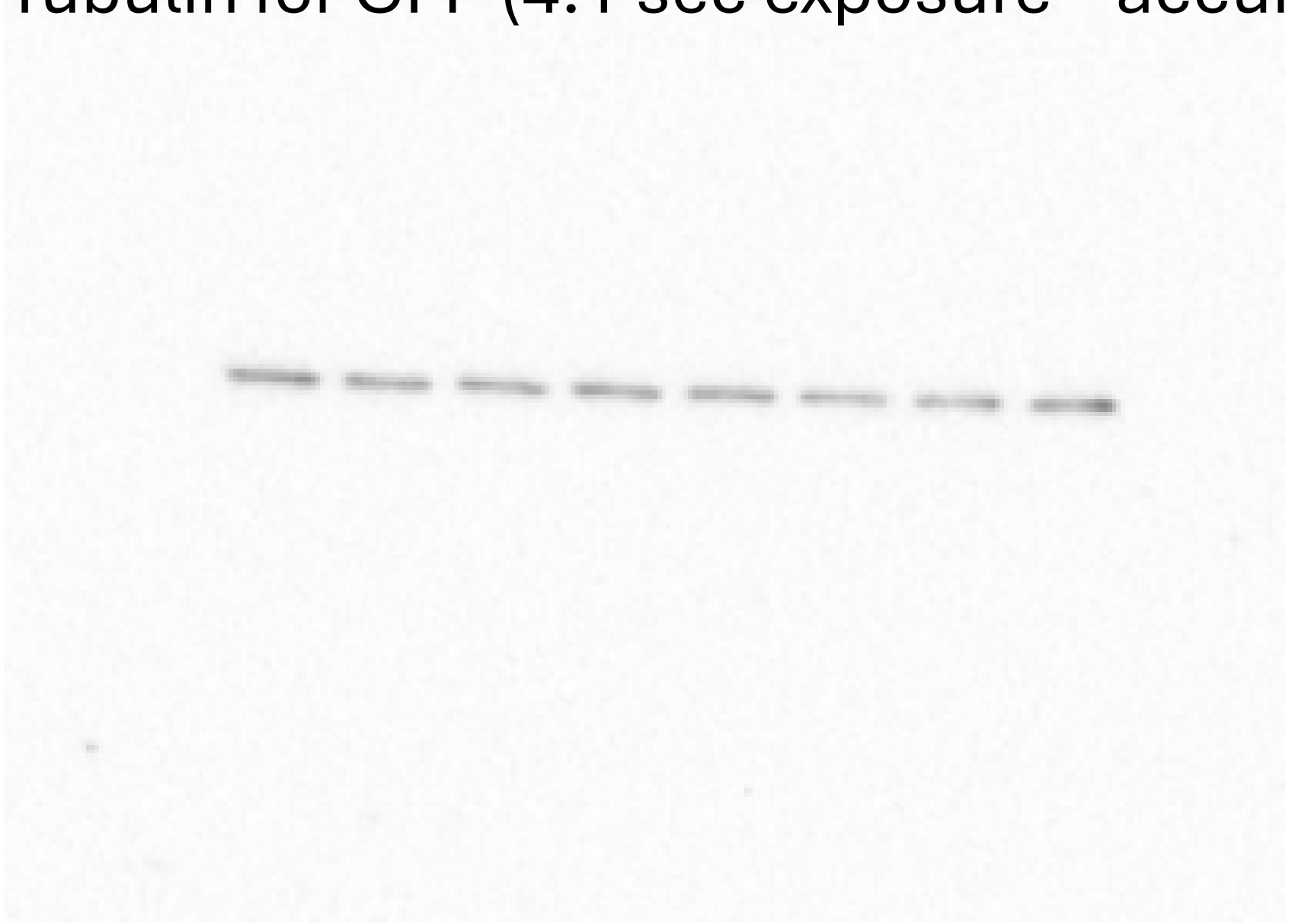

GFP (4.1 sec exposure – accumulation mode, max substrate)

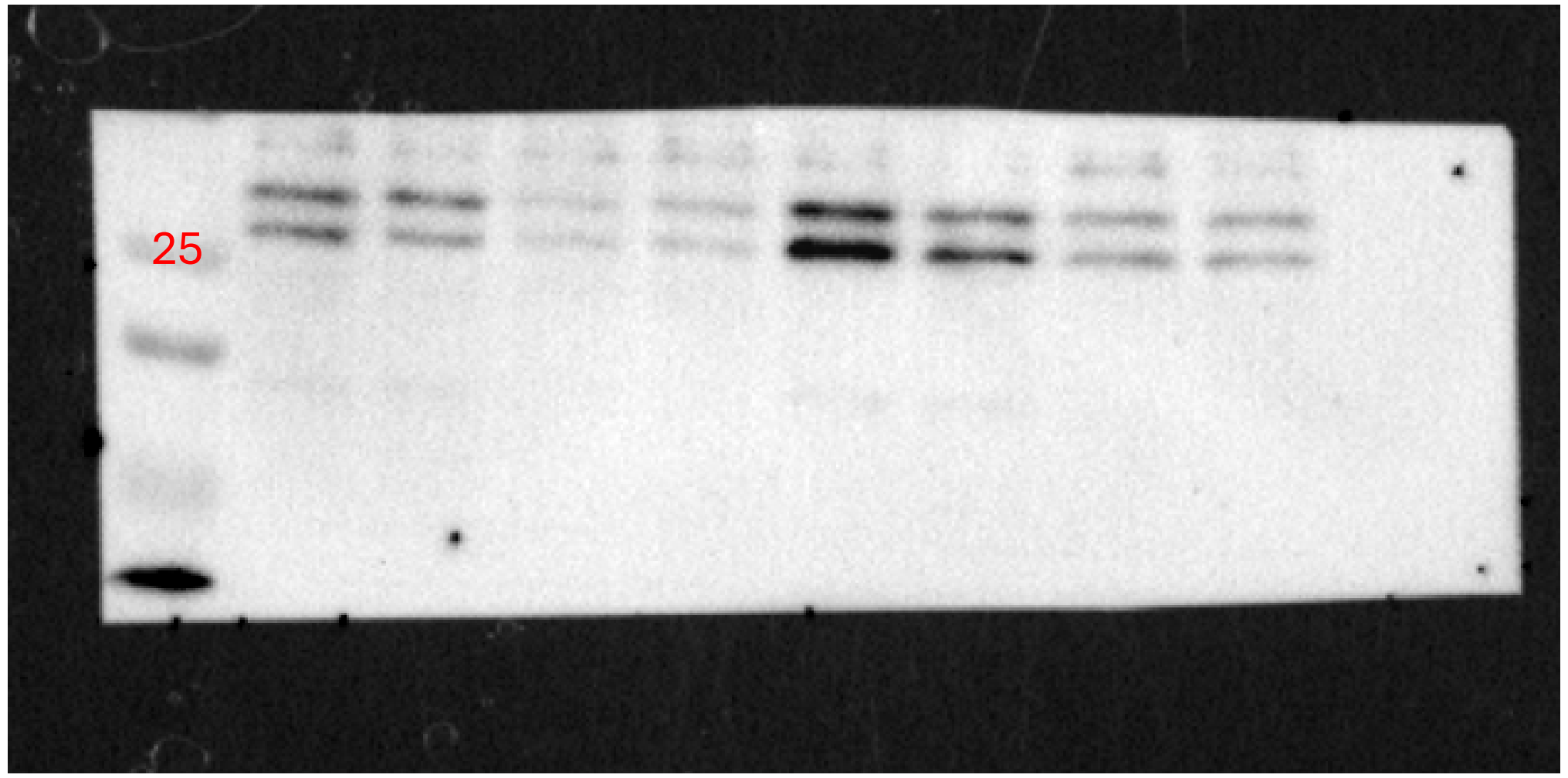

1 sec exposure – accumulation mode

B-Tubulin

GFP

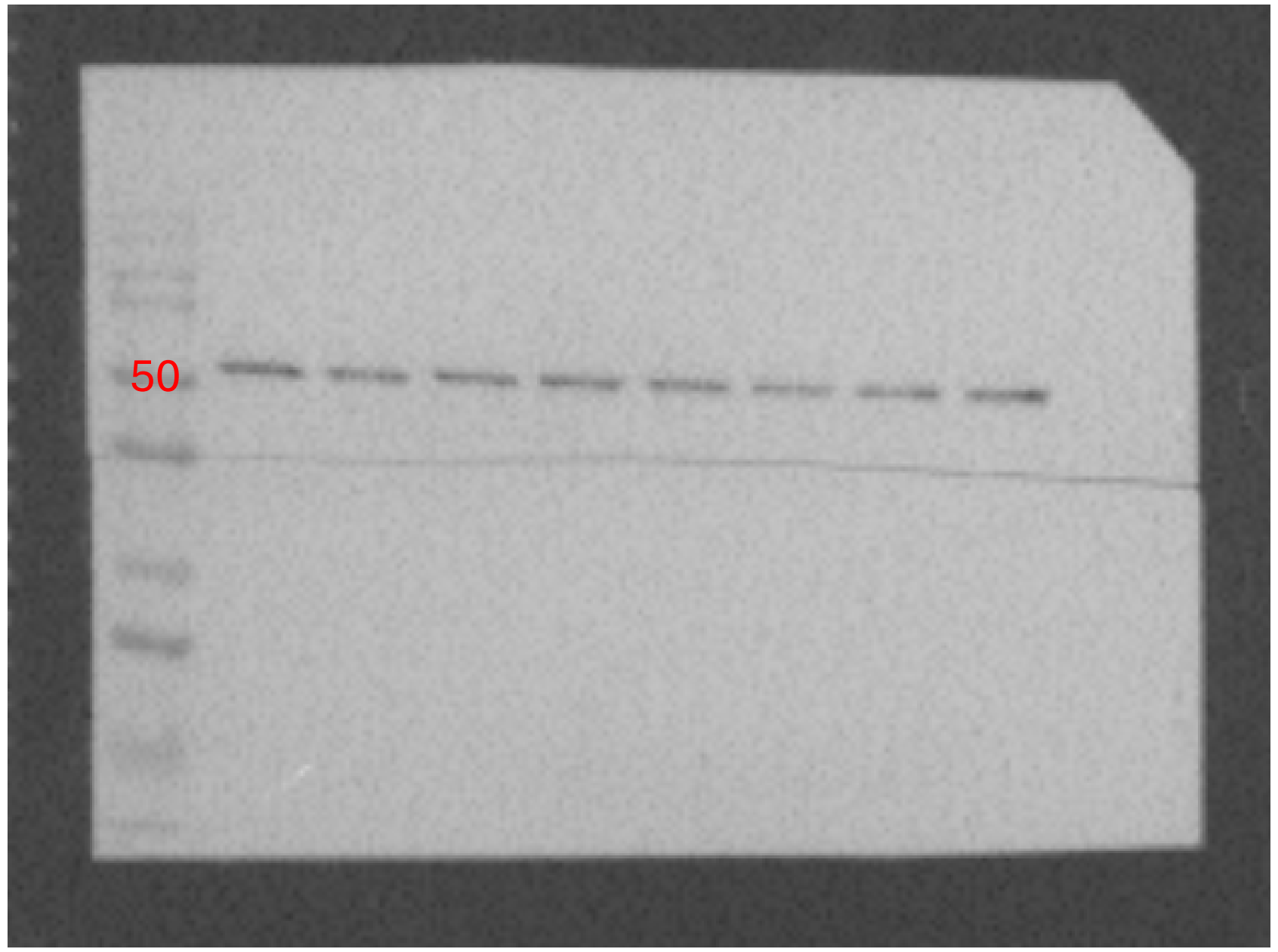

Figure 2C

ZFC3H1 (7.2 sec exposure – accumulation mode, max substrate)

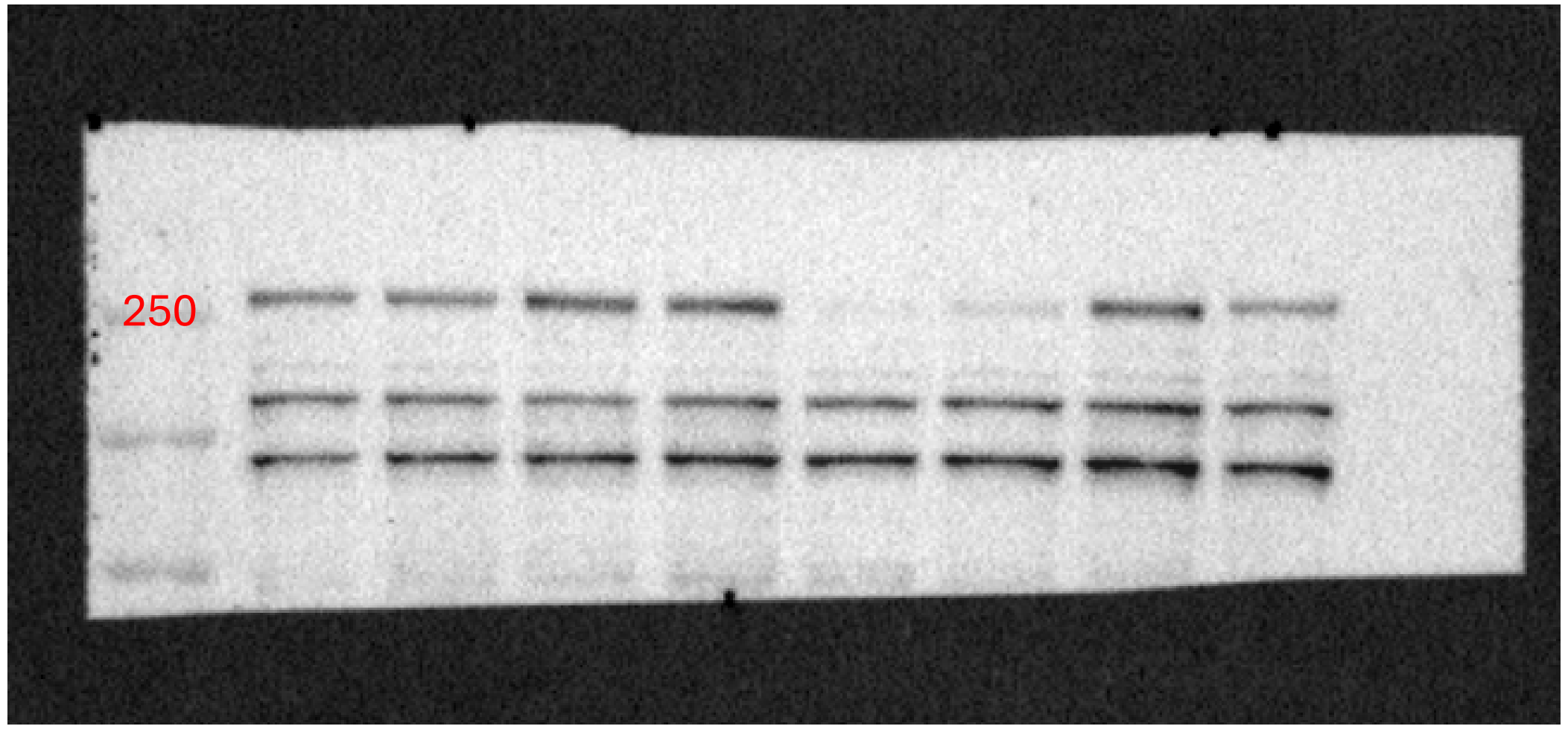

B-Tubulin for ZFC3H1 (1 sec exposure – accumulation mode)

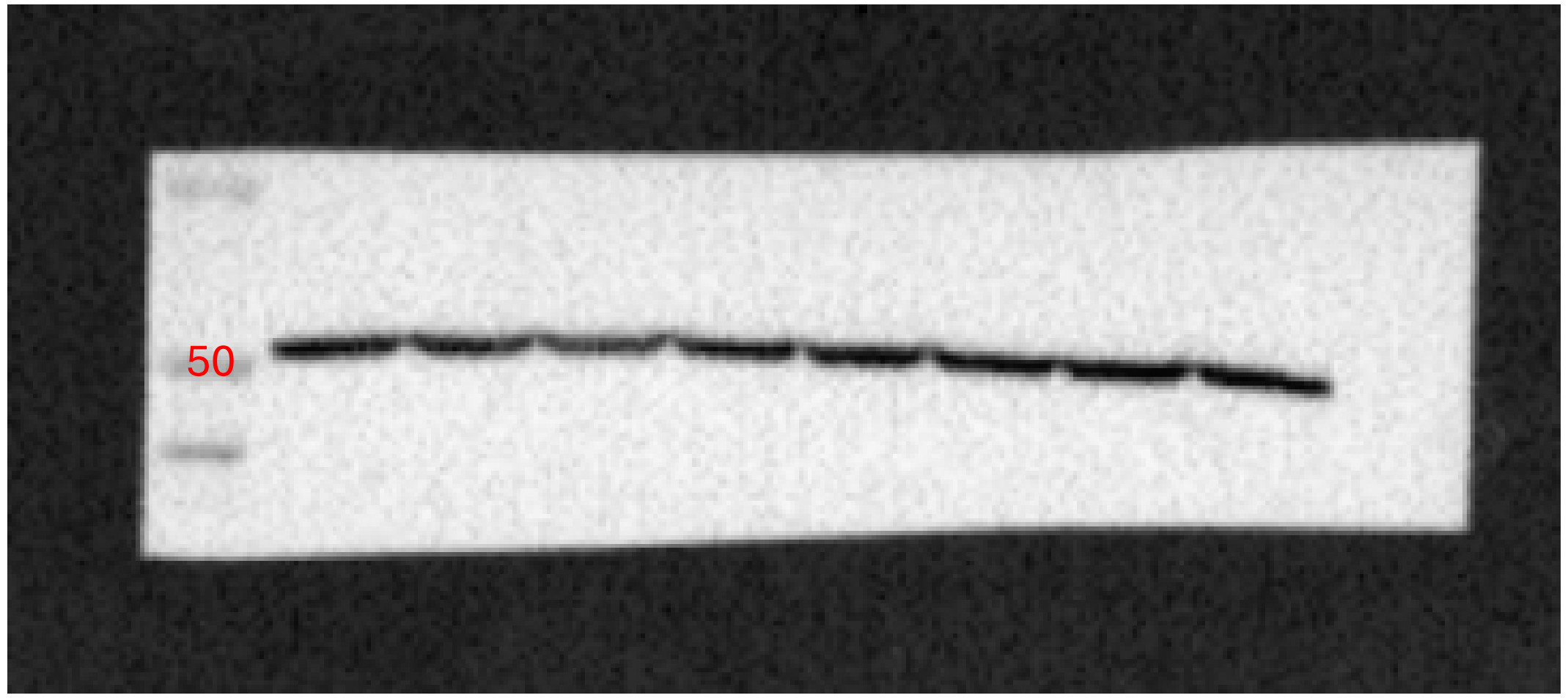

Lane	Sample
1	Ladder
2	Ctrl guide 1, Empty Vector
3	Ctrl guide 2, Empty Vector
4	Ctrl guide 1, FLAG-ZFC3H1
5	Ctrl guide 2, FLAG-ZFC3H1
6	ZFC3H1 guide 1, Empty Vector
7	ZFC3H1 guide 2, Empty Vector
8	ZFC3H1 guide 1, FLAG-ZFC3H1
9	ZFC3H1 guide 2, FLAG-ZFC3H1

Figure 2C

B-Tubulin for mCherry/RFP (1 sec exposure – accumulation mode)

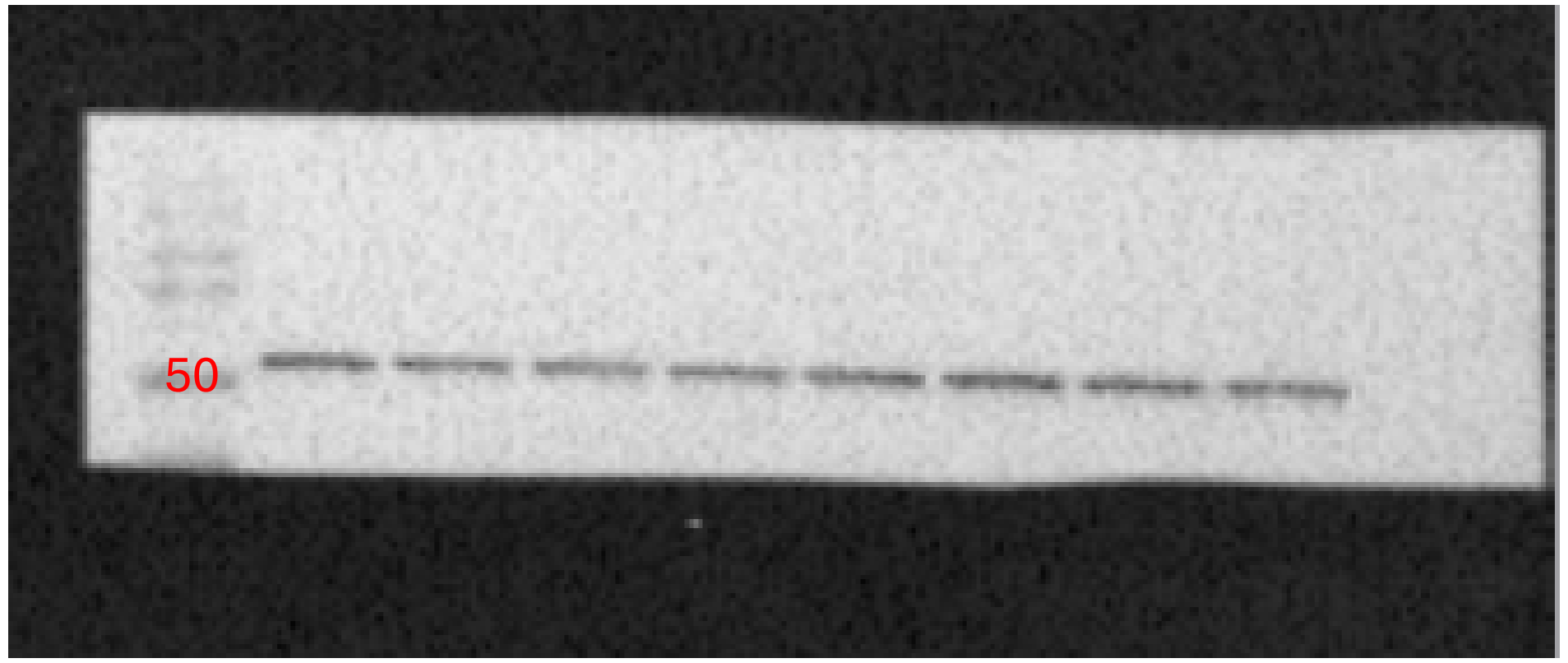

mCherry/RFP (4.1 sec exposure – accumulation mode, max substrate)

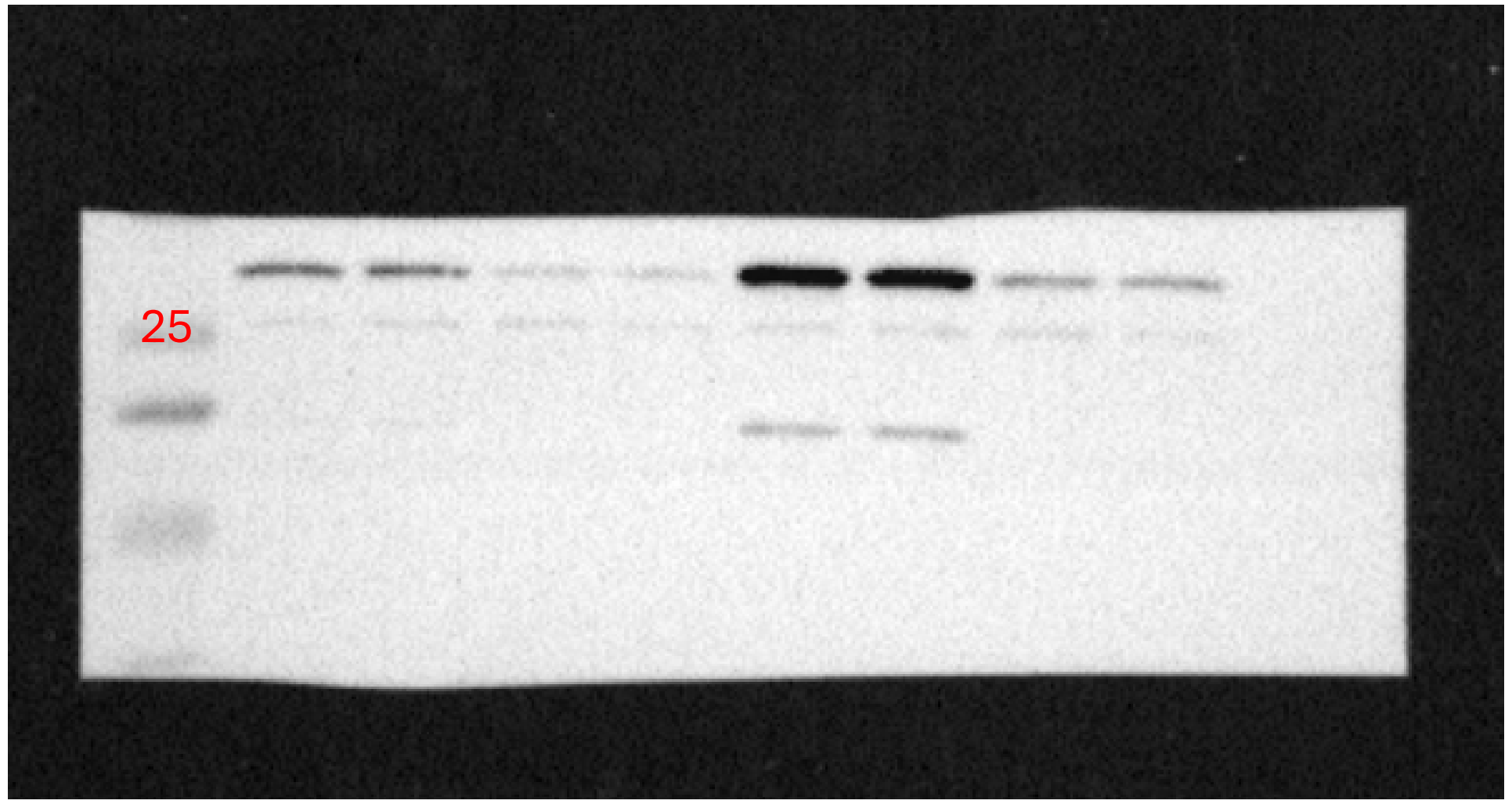

Figure 2C

B-Tubulin for GFP(1 sec exposure – accumulation mode)

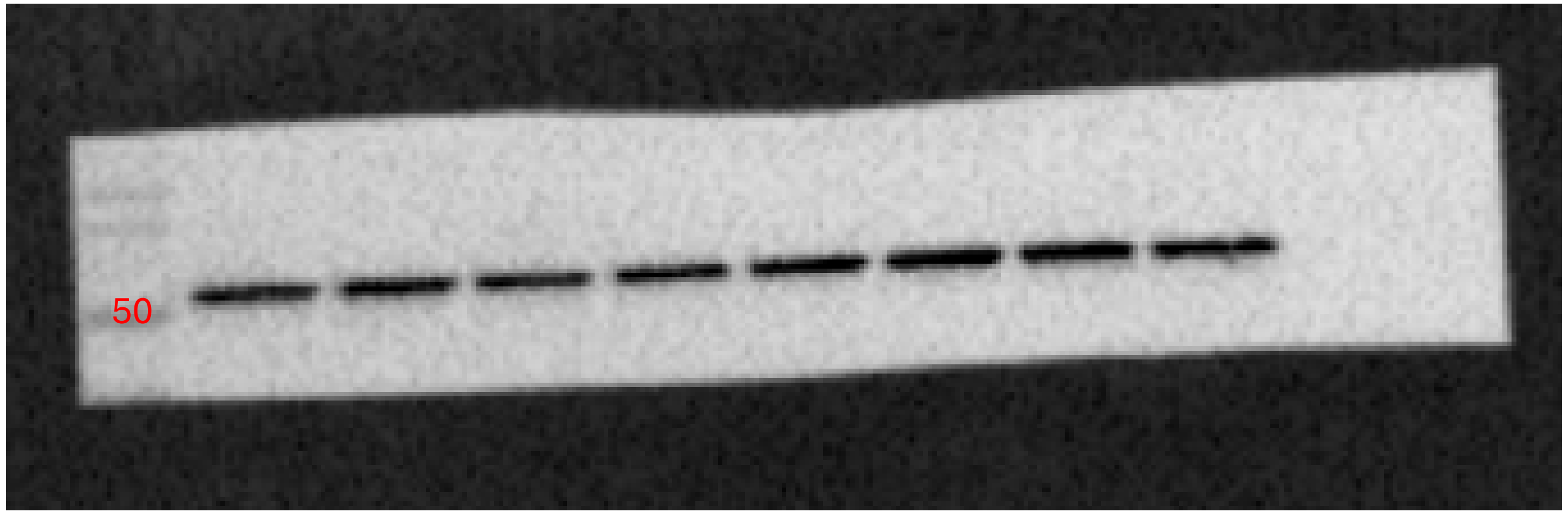

GFP(1 sec exposure – accumulation mode)

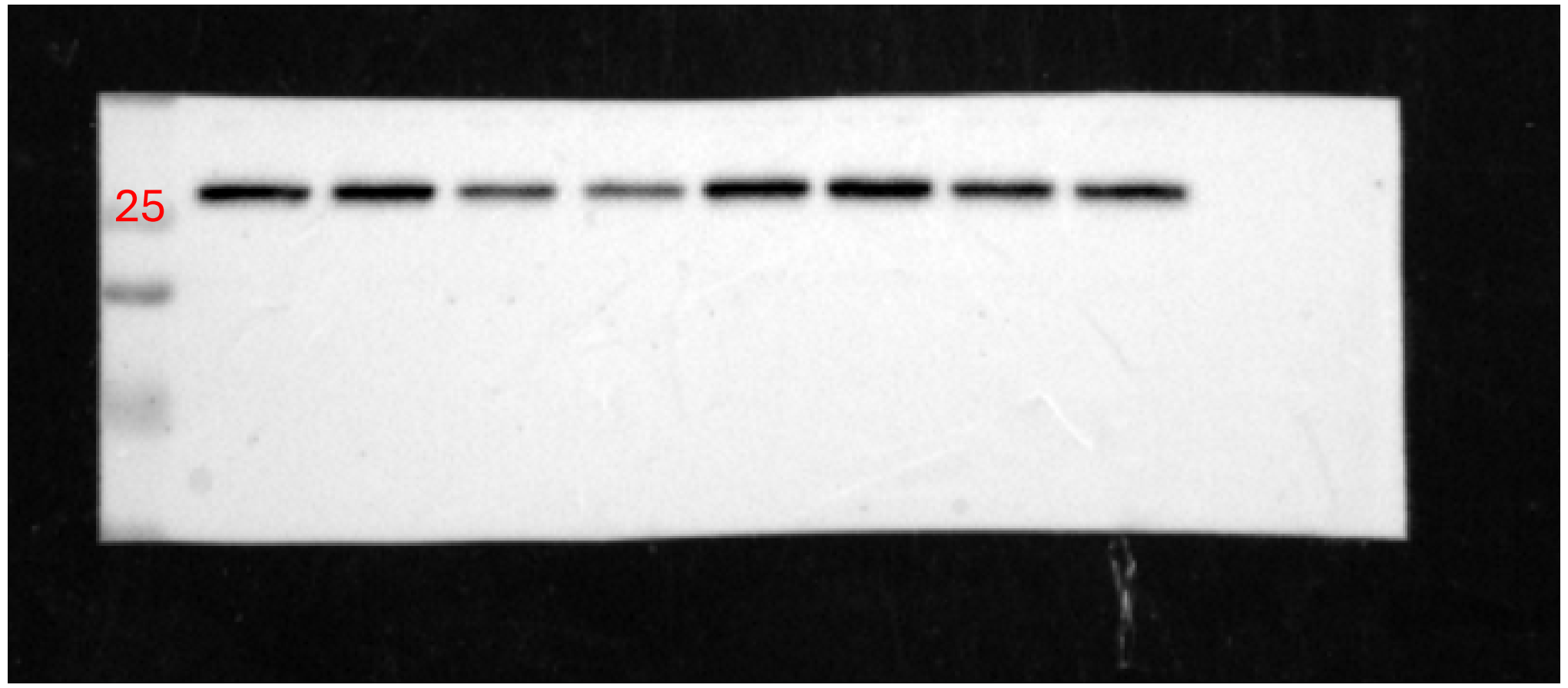

Figure 6A

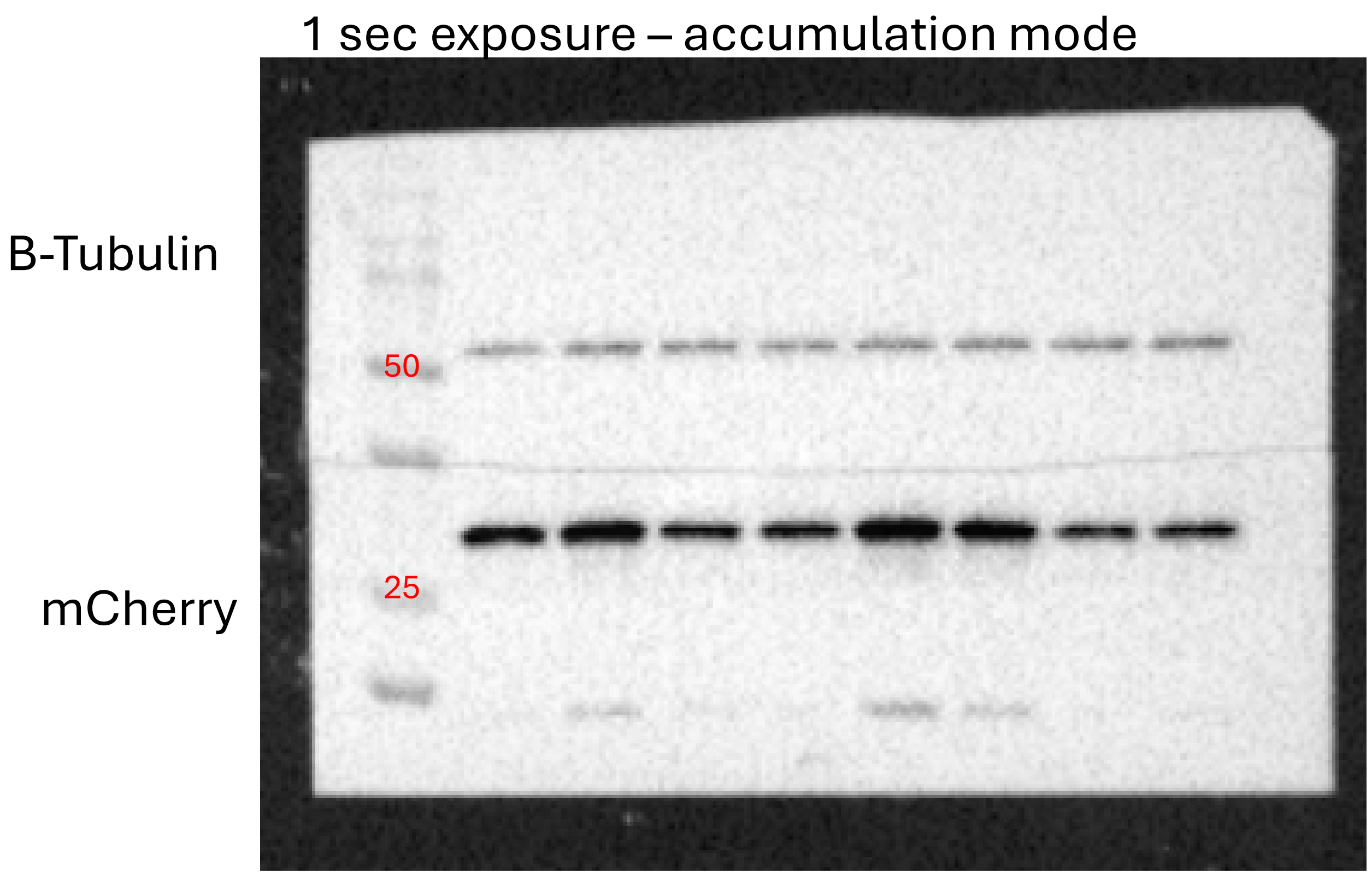

Lane	Sample
1	Ladder
2	Ctrl guide 1, Empty Vector
3	Ctrl guide 2, Empty Vector
4	Ctrl guide 1, FLAG-PABPN1
5	Ctrl guide 2, FLAG-PABPN1
6	PABPN1 guide 1, Empty Vector
7	PABPN1 guide 2, Empty Vector
8	PABPN1 guide 1, FLAG-PABPN1
9	PABPN1 guide 2, FLAG-PABPN1

Figure 6A

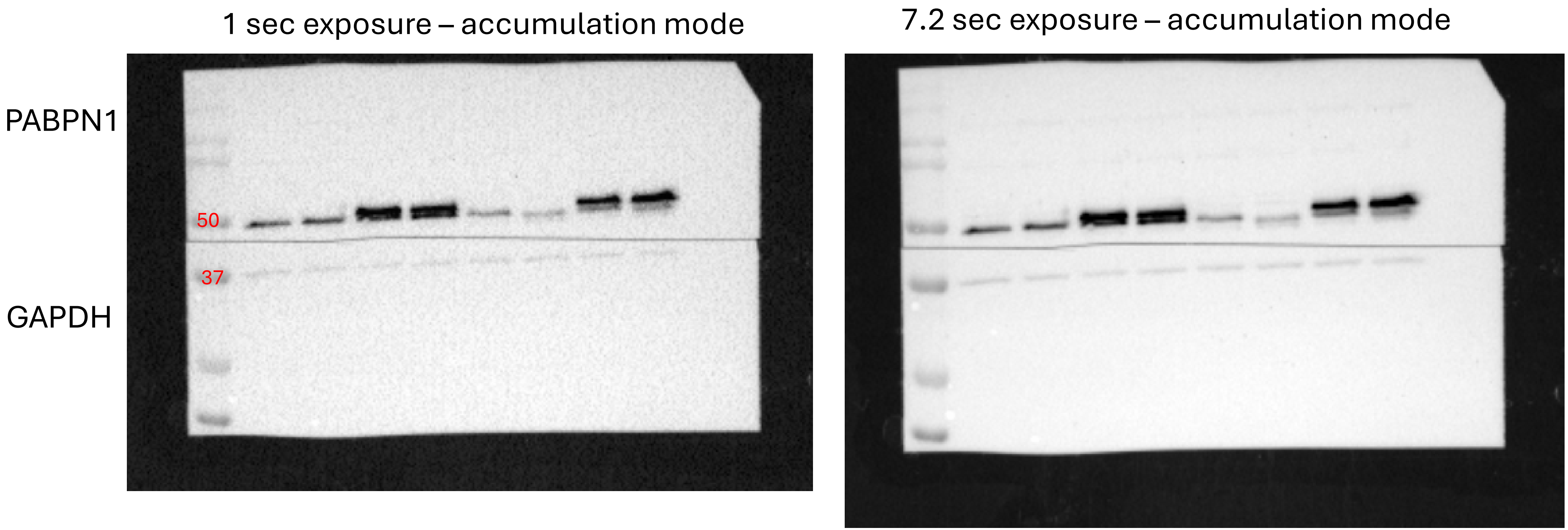

1 sec exposure – accumulation mode
7.2 sec exposure – accumulation mode
PABPN1
GAPDH
50
37

Figure 6A

GFP(1 sec exposure – accumulation mode, max substrate)

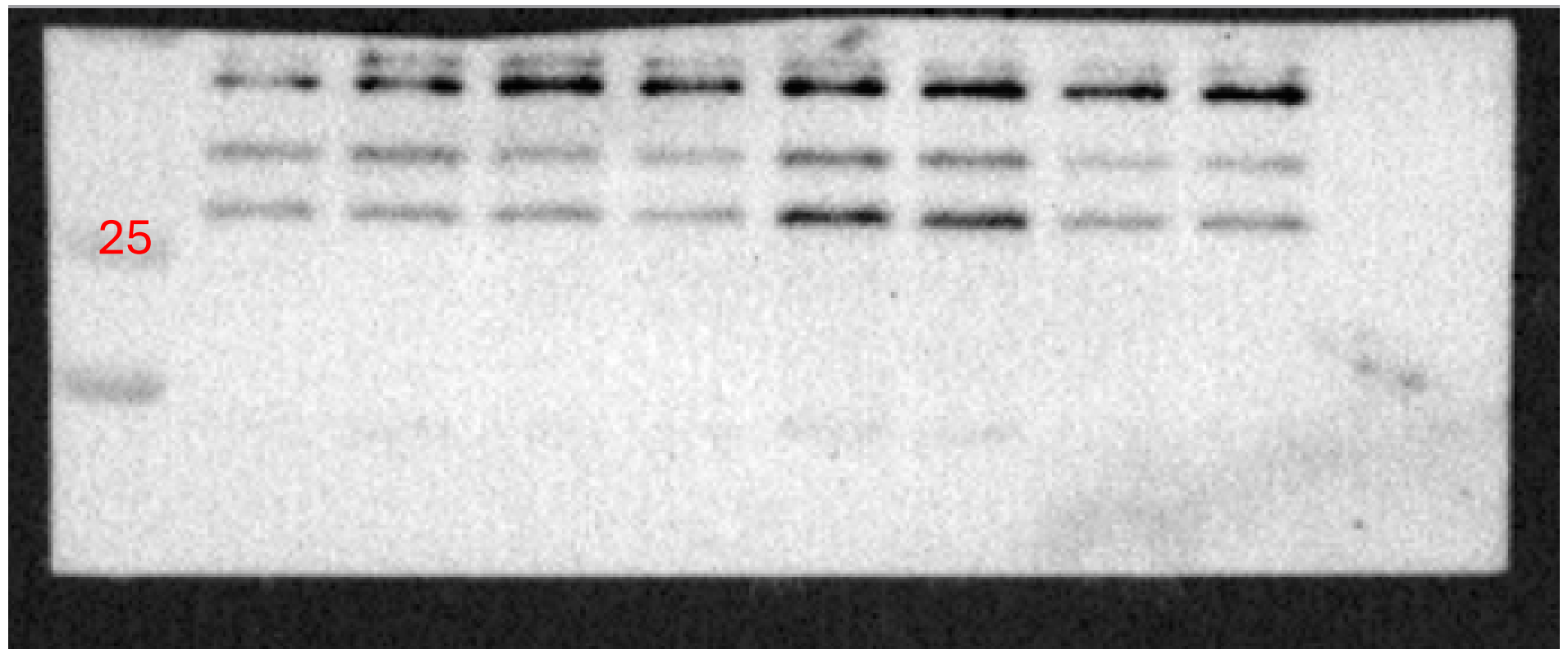

1 sec exposure – accumulation mode

B-Tubulin

GFP

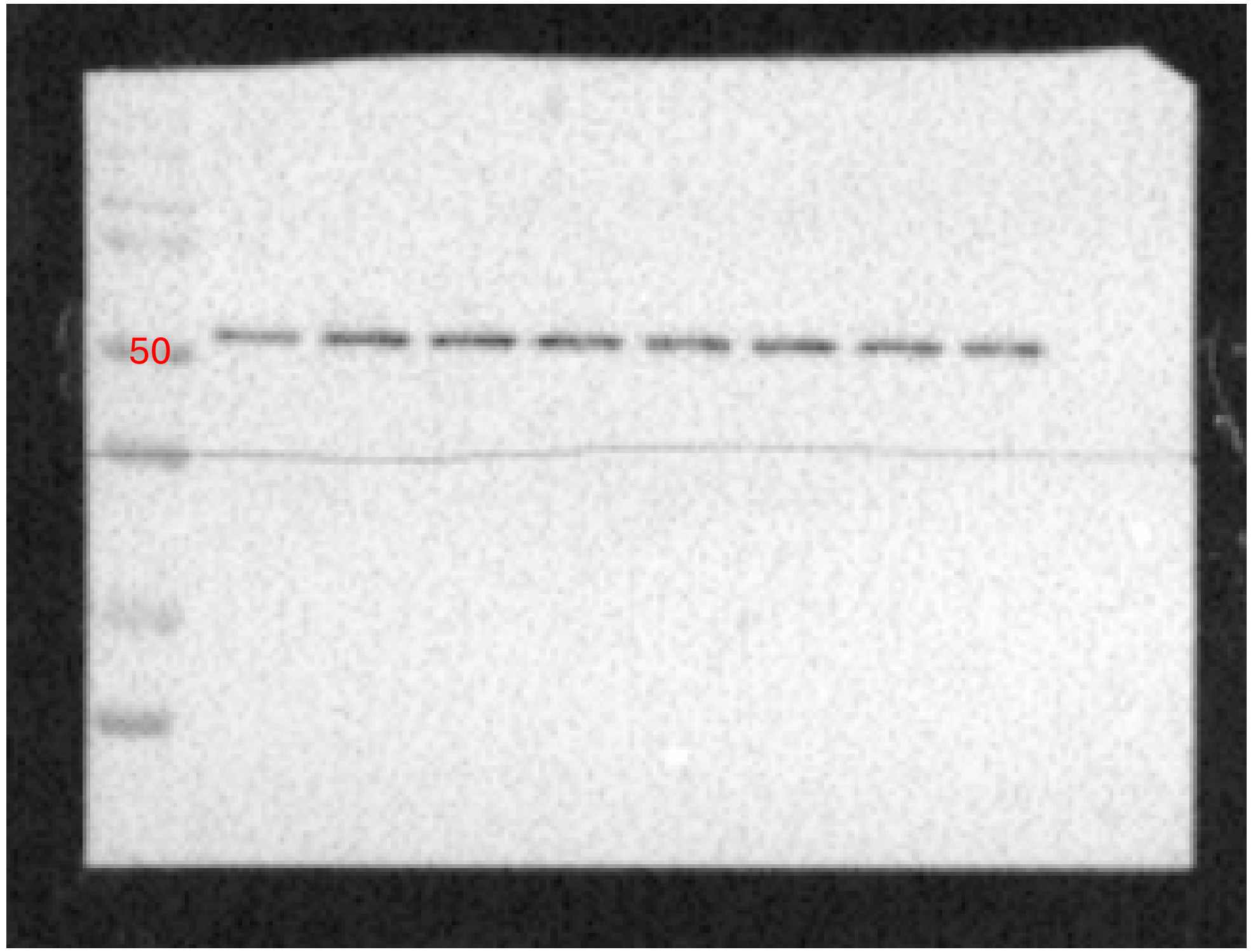

Figure 6B

Lane	Sample
1	Ladder
2	Ctrl guide 1, Empty Vector
3	Ctrl guide 2, Empty Vector
4	Ctrl guide 1, FLAG-PABPN1
5	Ctrl guide 2, FLAG-PABPN1
6	PABPN1 guide 1, Empty Vector
7	PABPN1 guide 2, Empty Vector
8	PABPN1 guide 1, FLAG-PABPN1
9	PABPN1 guide 2, FLAG-PABPN1

Figure 6B

4.1 sec exposure – accumulation mode

B-Tubulin

mCherry

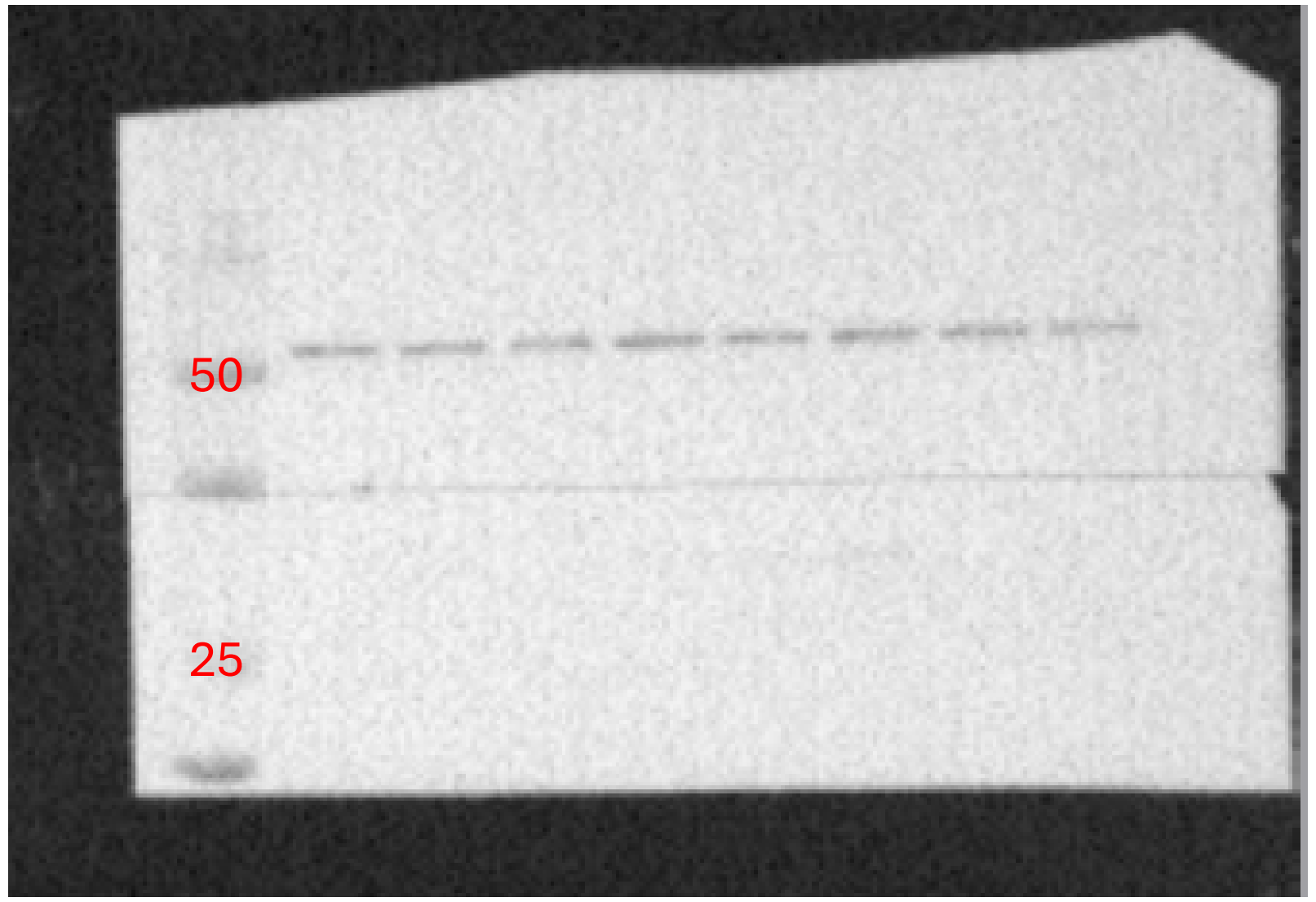

mCherry(4.1 sec exposure – accumulation mode, max substrate)

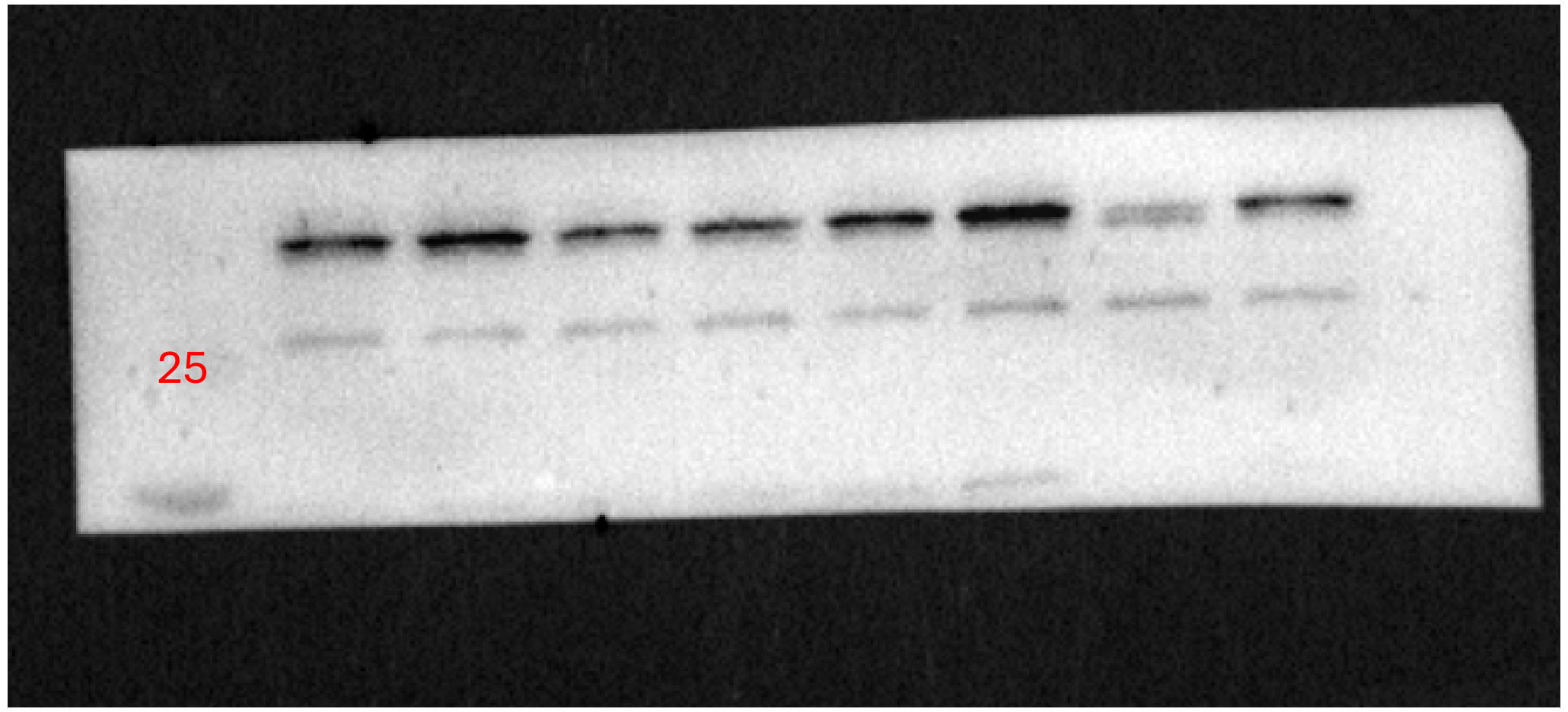

10.3 sec exposure – accumulation mode

B-Tubulin

mCherry

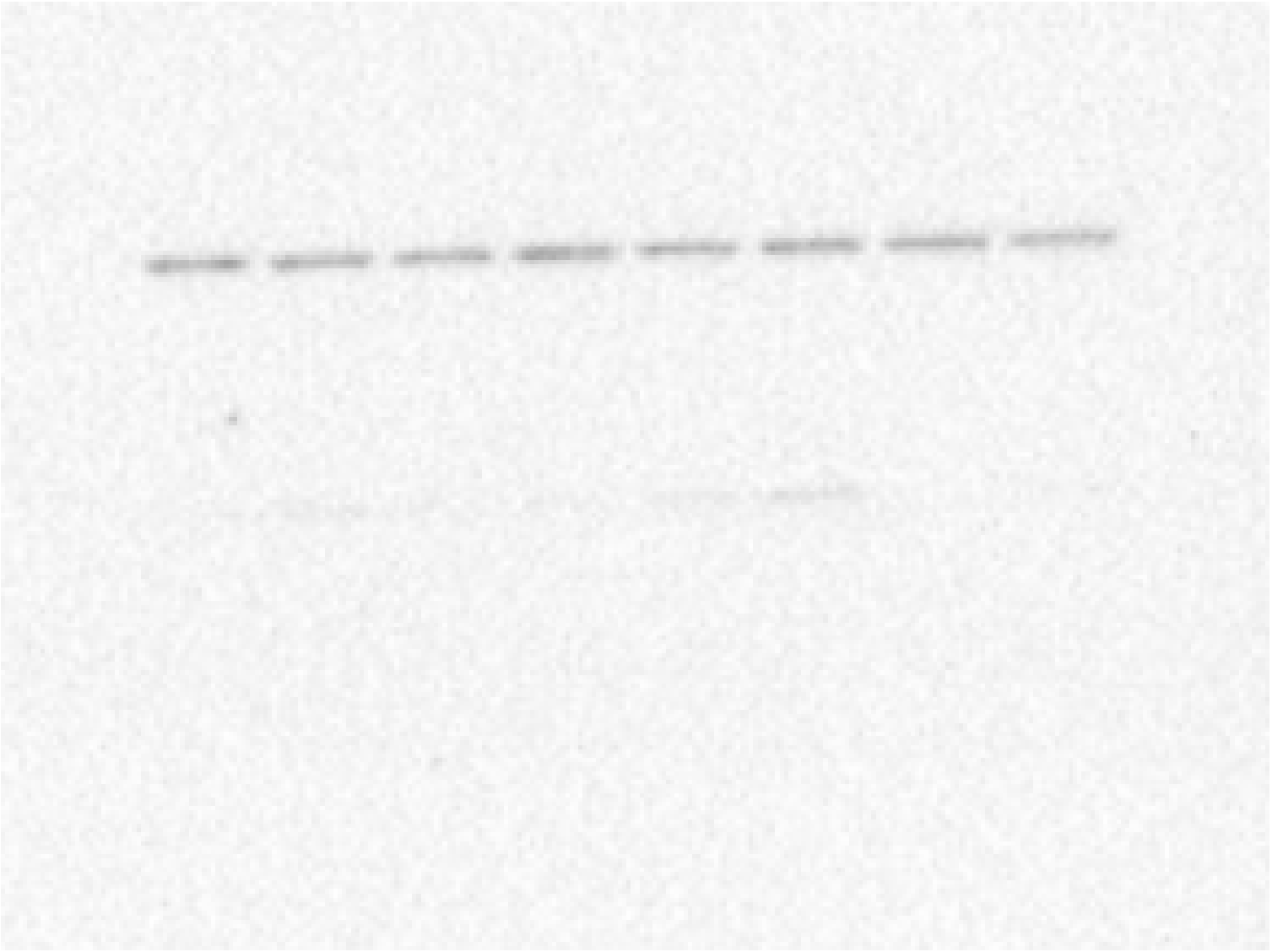

Figure 6B

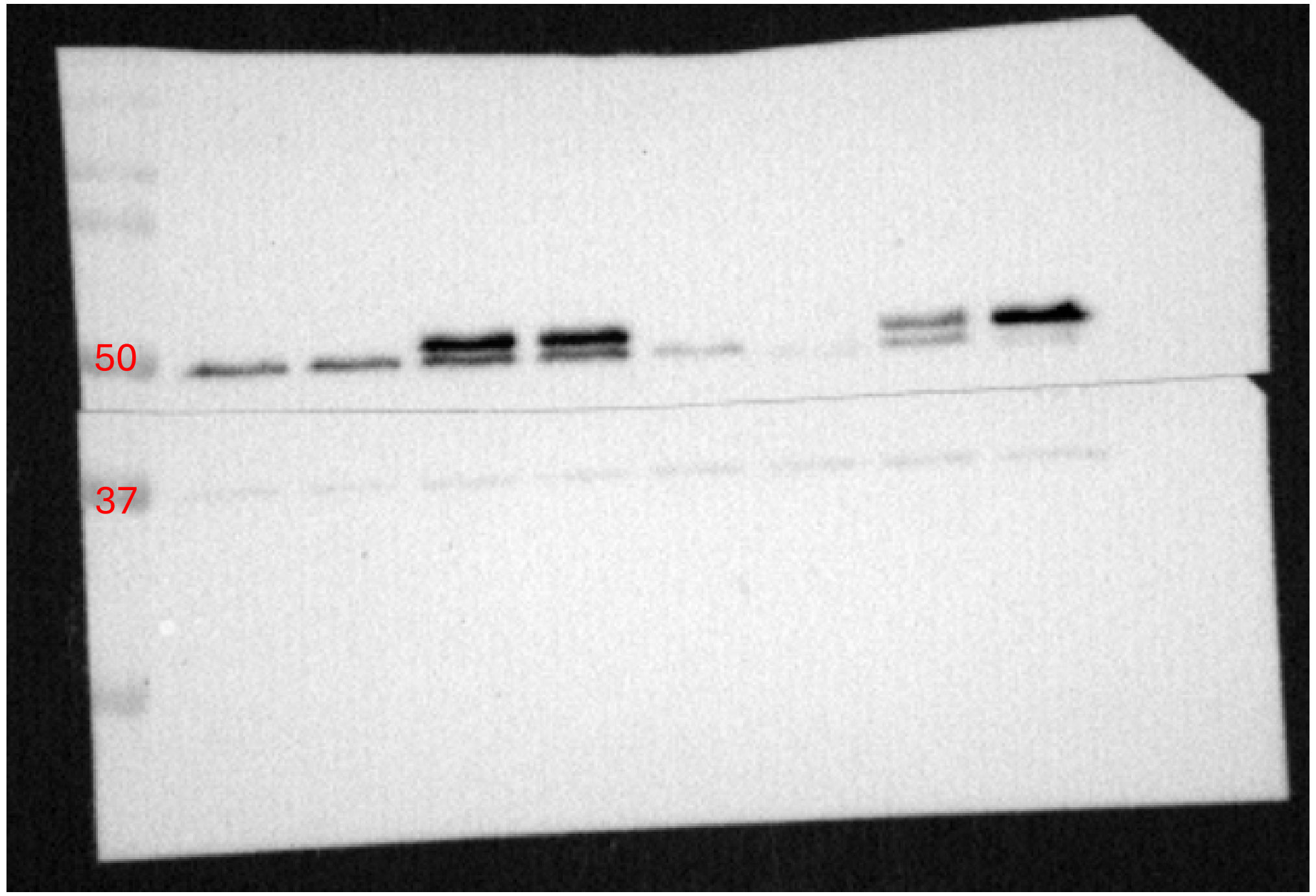
10.3 sec exposure – accumulation mode
PABPN1
GAPDH
50
37

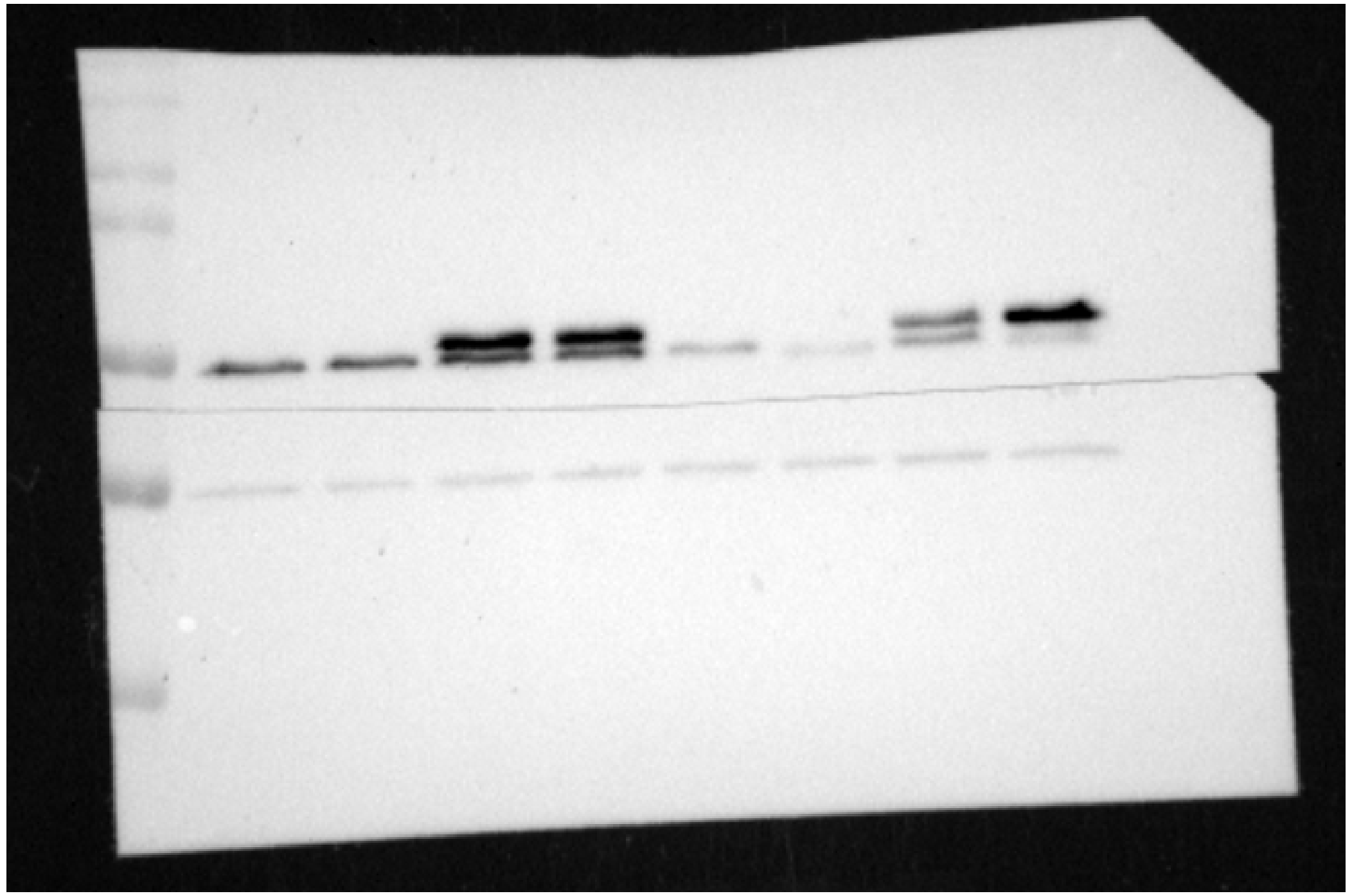
38.3 sec exposure – accumulation mode

Figure 6B

1 sec exposure – accumulation mode

B-Tubulin

GFP

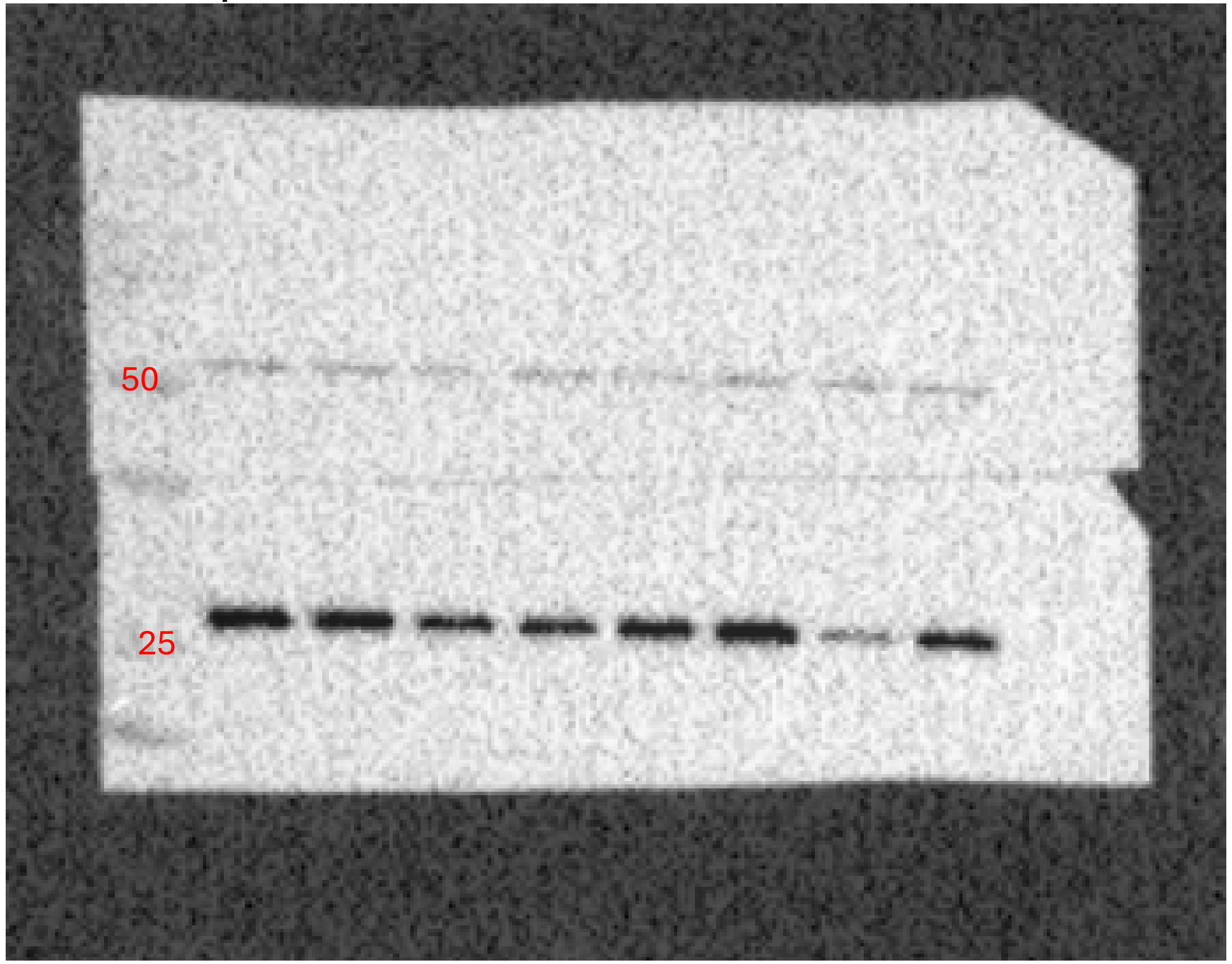

10.3 sec exposure – accumulation mode

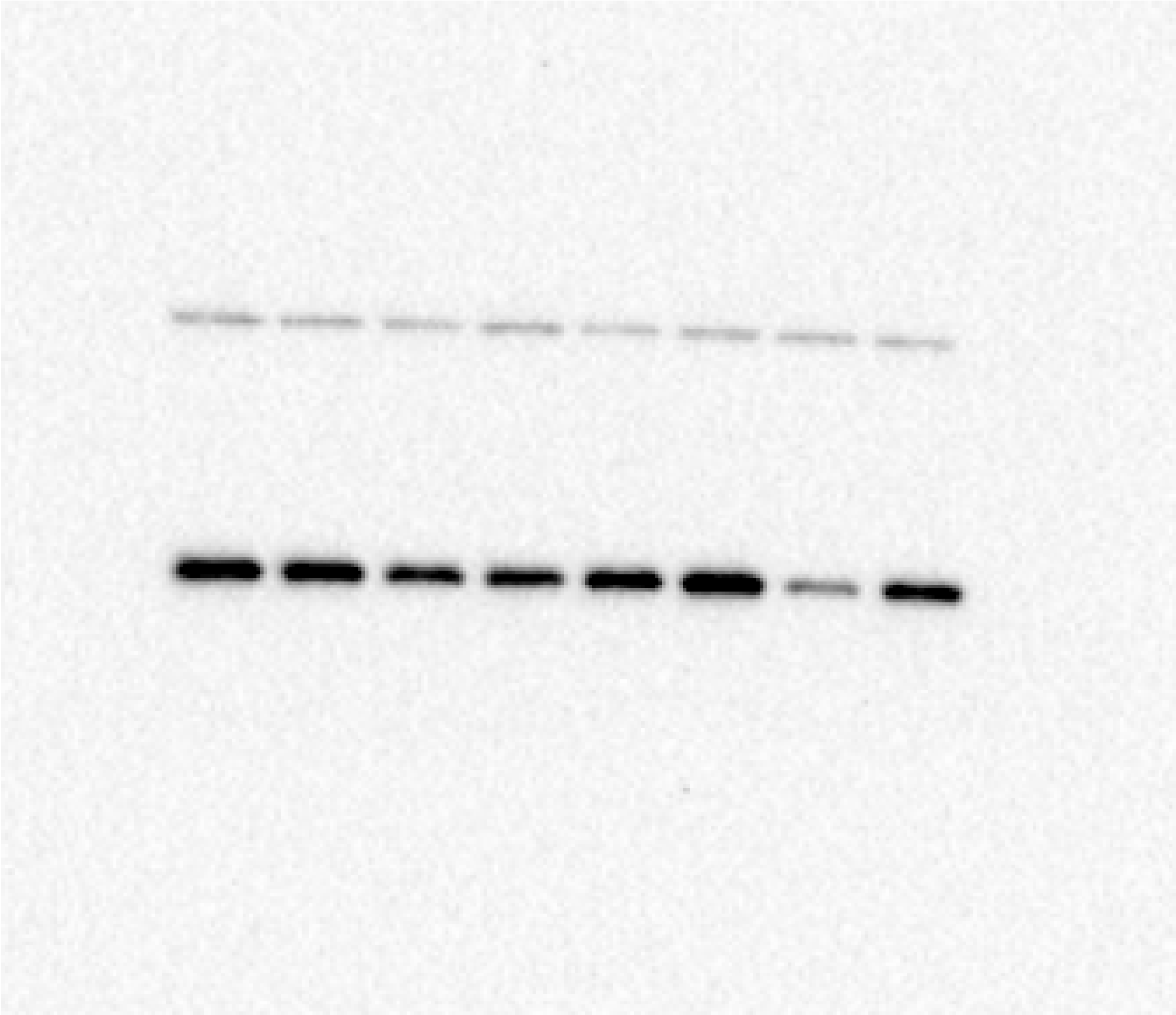

Supplementary Figure 2A

ZFC3H1 (16.5 sec exposure – accumulation mode, max substrate)

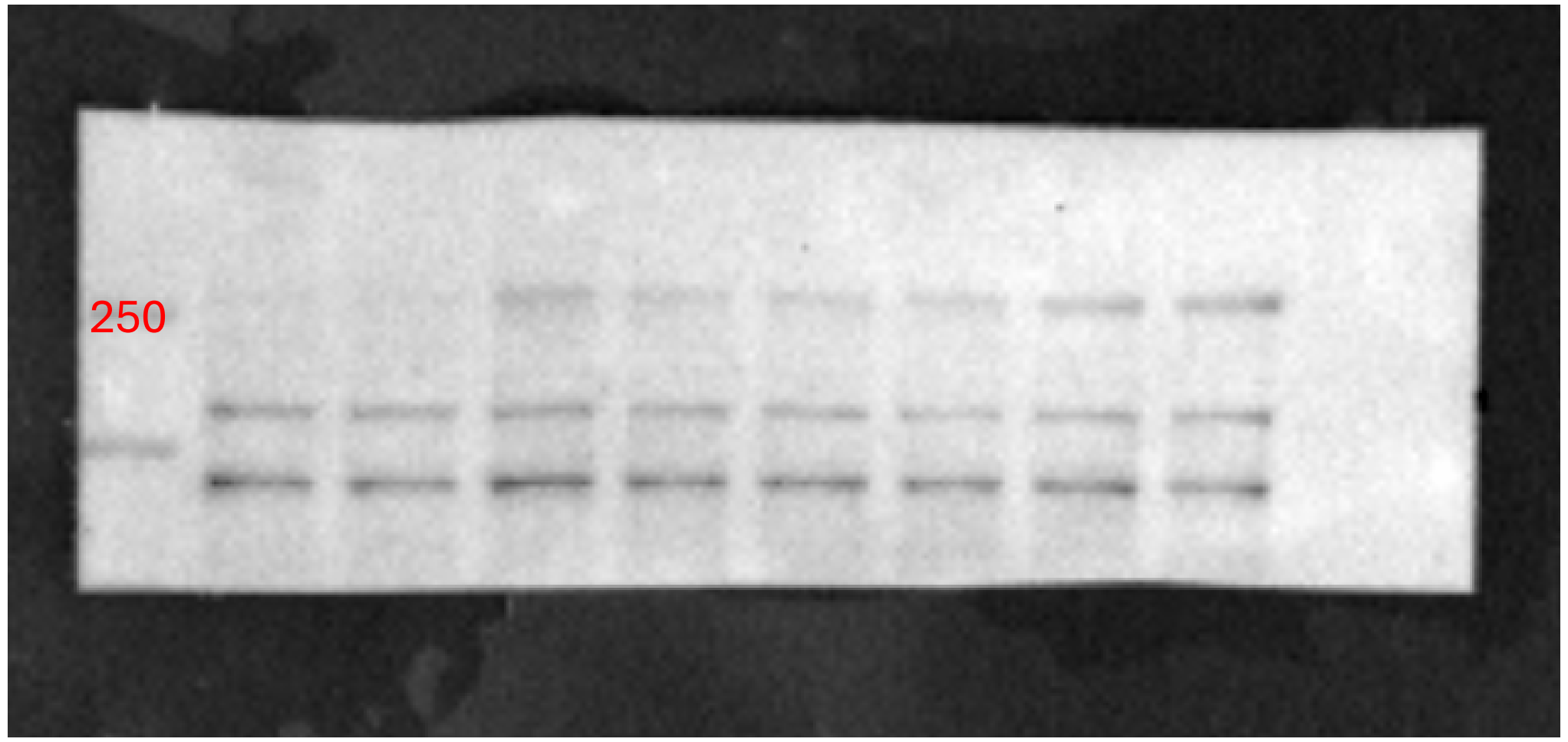

B-Tubulin for ZFC3H1 (4.1 sec exposure – accumulation mode)

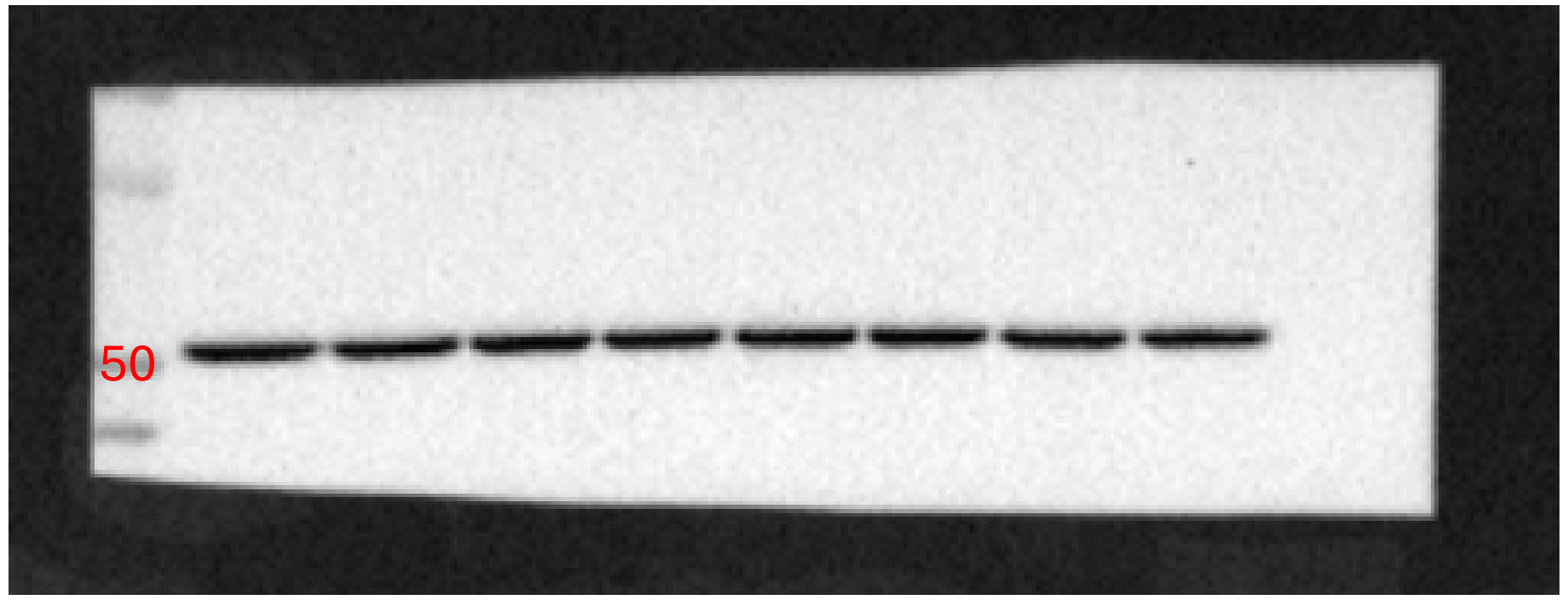

Lane	Sample
1	Ladder
2	ZFC3H1 guide 1, Empty Vector
3	ZFC3H1 guide 2, Empty Vector
4	ZFC3H1 guide 1, FLAG-ZFC3H1
5	ZFC3H1 guide 2, FLAG-ZFC3H1
6	Ctrl guide 1, Empty Vector
7	Ctrl guide 2, Empty Vector
8	Ctrl guide 1, FLAG-ZFC3H1
9	Ctrl guide 2, FLAG-ZFC3H1

Supplementary Figure 2A

B-Tubulin for mCherry/RFP (4.1 sec exposure – accumulation mode)

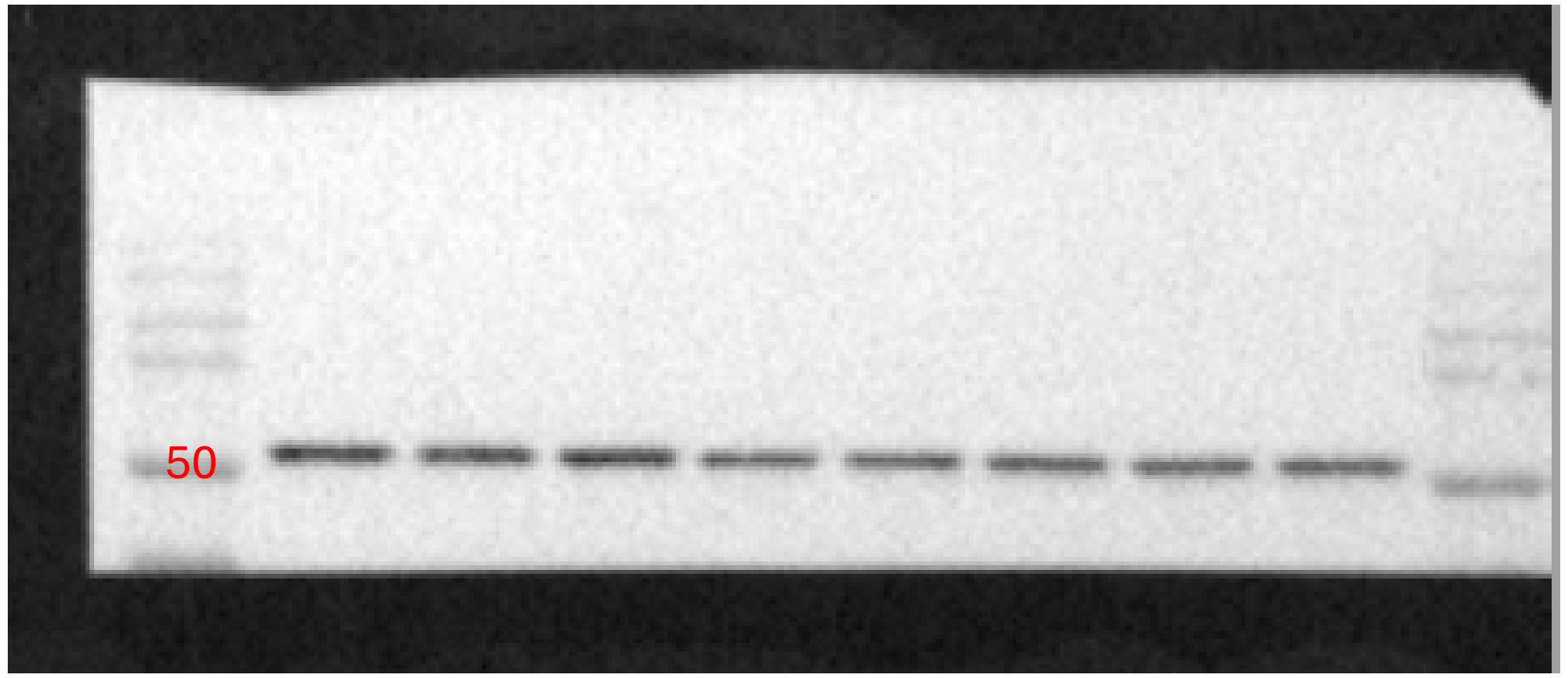

mCherry/RFP (0.5 sec exposure)

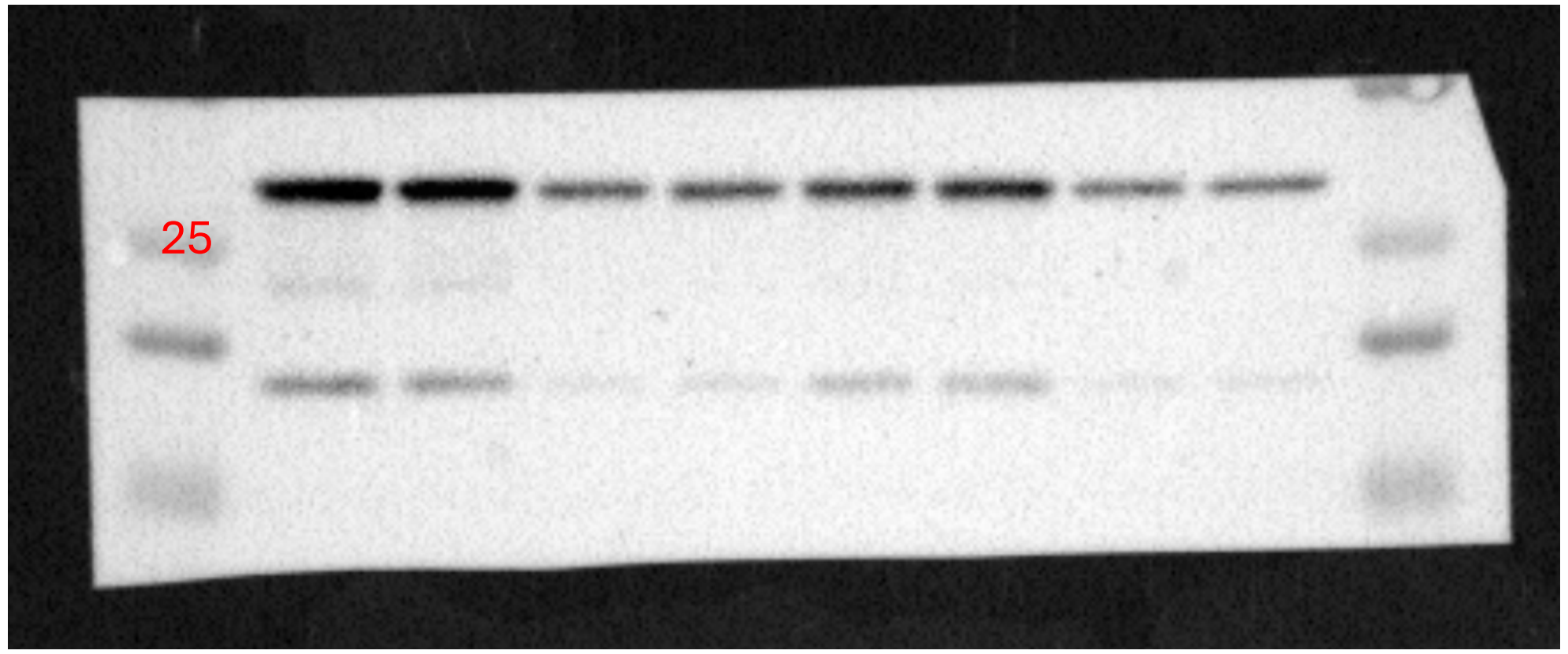

Supplementary Figure 2A

B-Tubulin for GFP (4.1 sec exposure – accumulation mode)

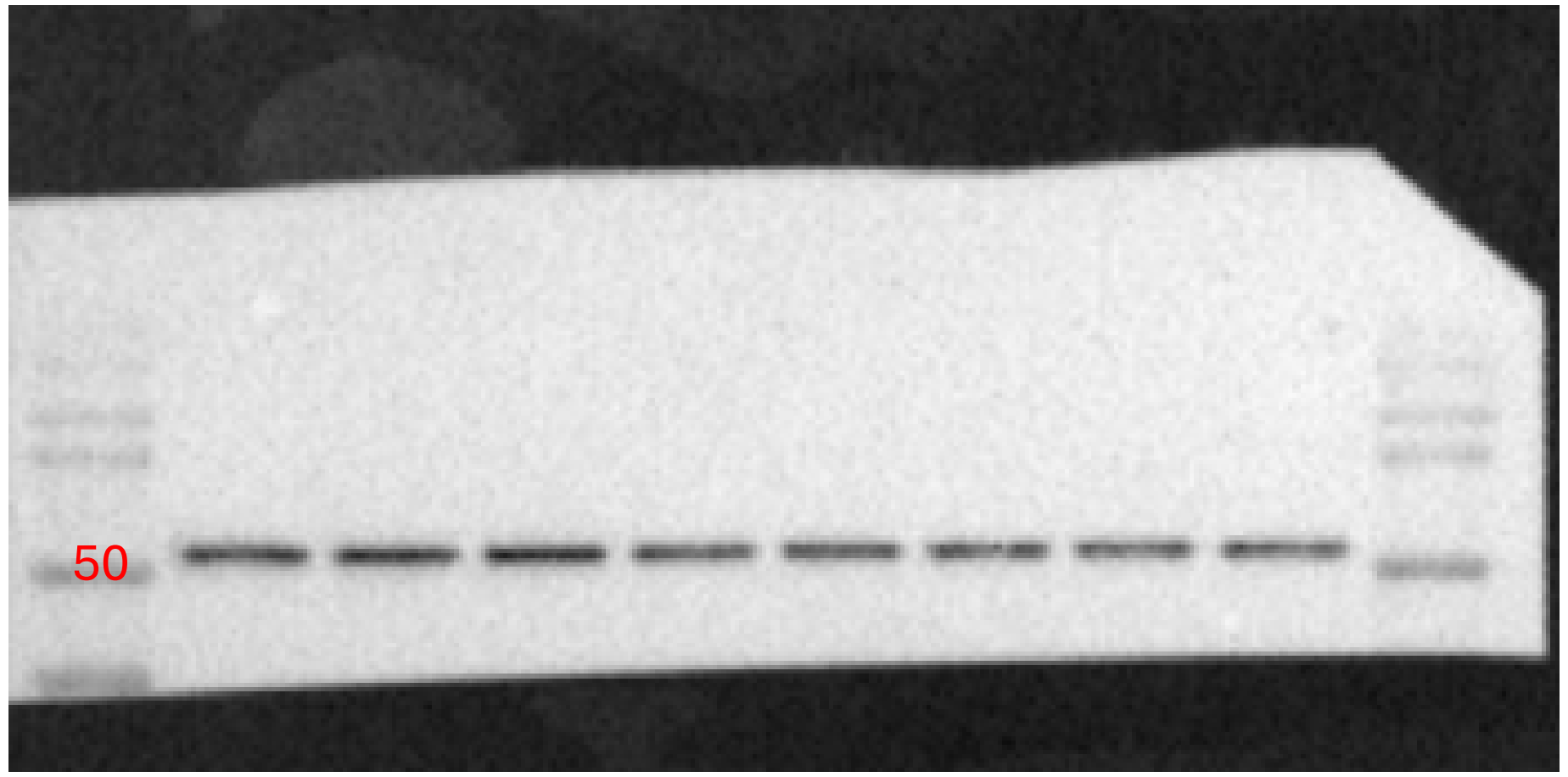

GFP (4.1 sec exposure – accumulation mode, max substrate)

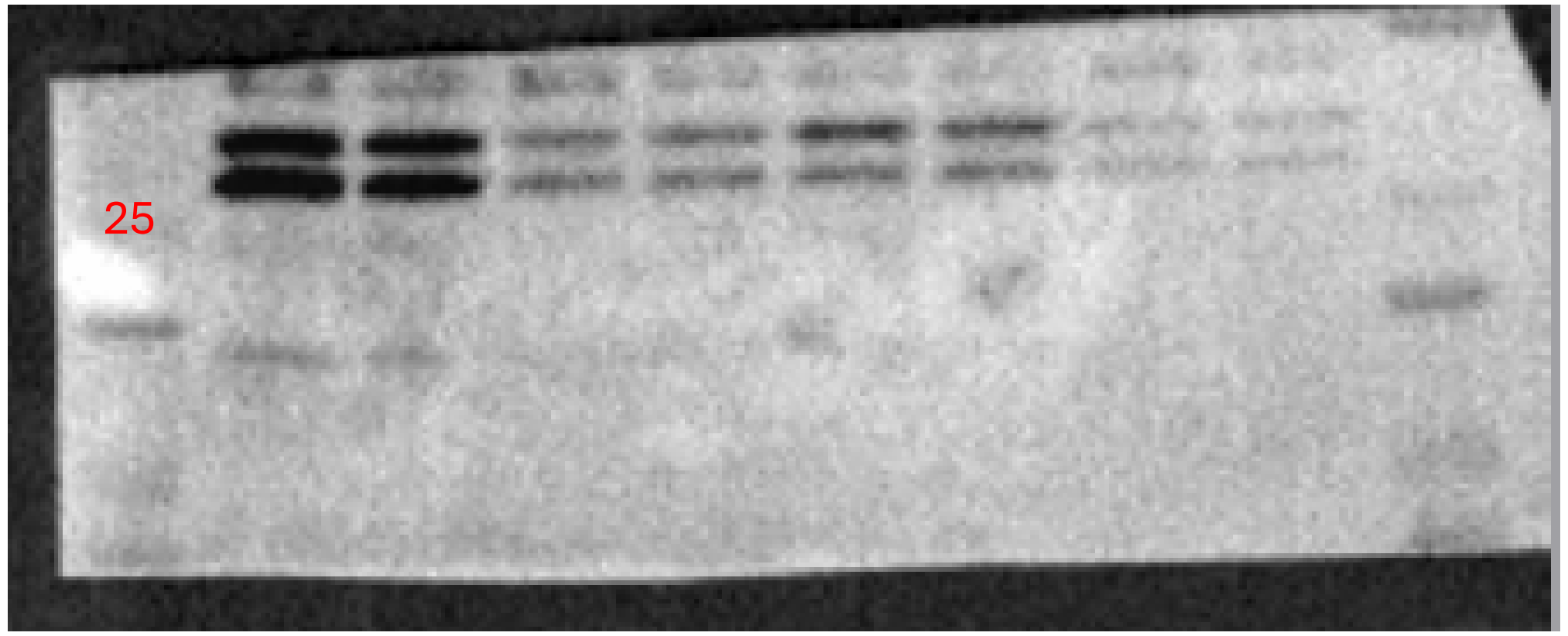

Supplementary Figure 2C

ZFC3H1 (19.6 sec exposure – accumulation mode, max substrate)

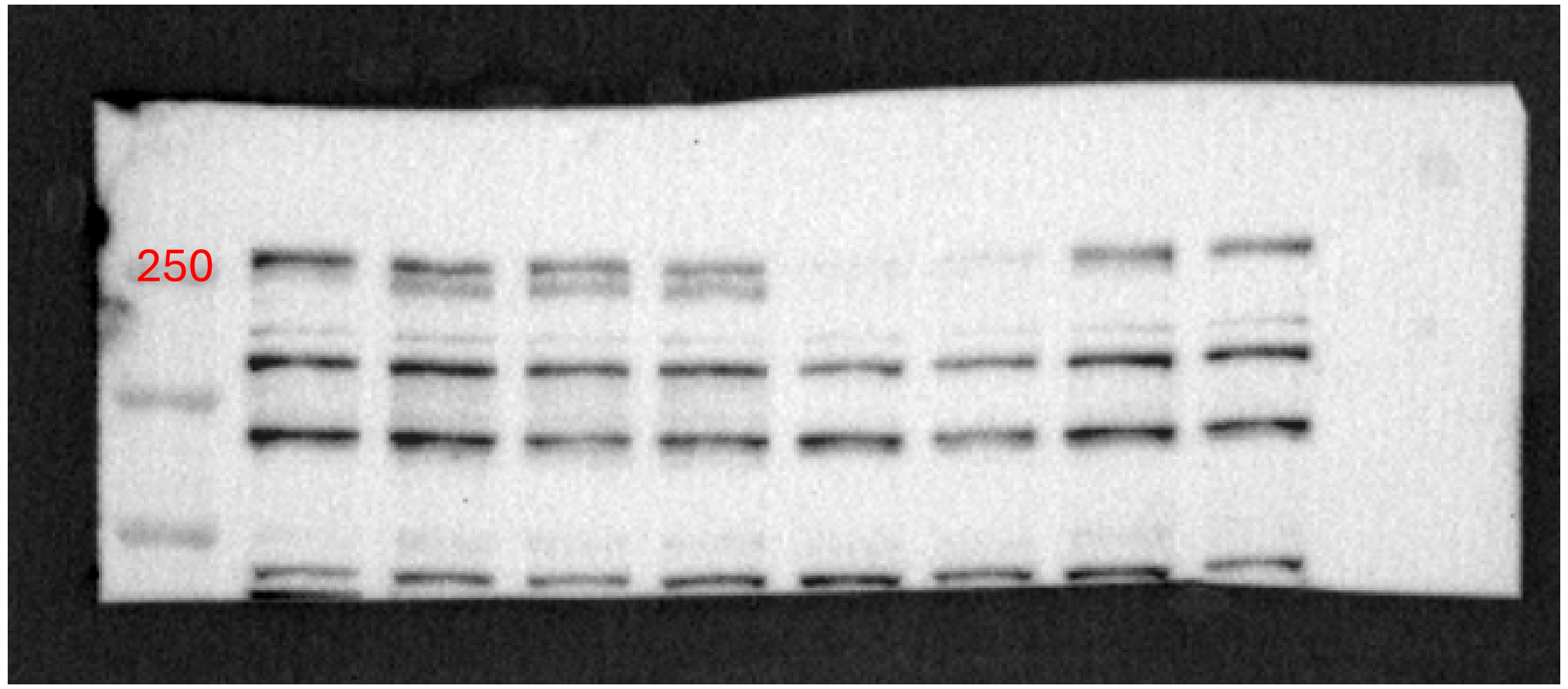

B-tubulin for ZFC3H1 (4.1 sec exposure – accumulation mode)

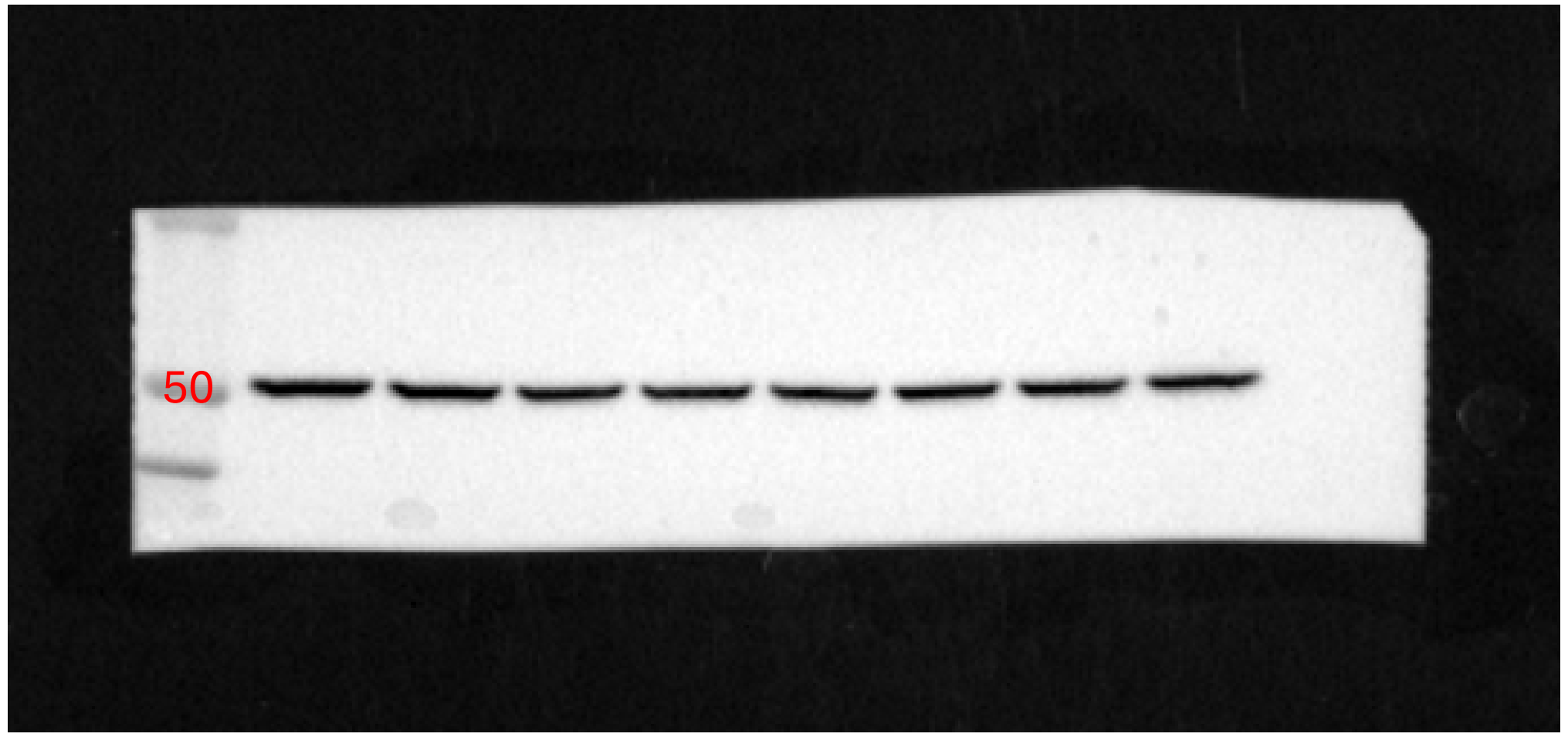

Lane	Sample
1	Ladder
2	Ctrl guide 1, Empty Vector
3	Ctrl guide 2, Empty Vector
4	Ctrl guide 1, FLAG-ZFC3H1
5	Ctrl guide 2, FLAG-ZFC3H1
6	ZFC3H1 guide 1, Empty Vector
7	ZFC3H1 guide 2, Empty Vector
8	ZFC3H1 guide 1, FLAG-ZFC3H1
9	ZFC3H1 guide 2, FLAG-ZFC3H1

Supplementary Figure 2C

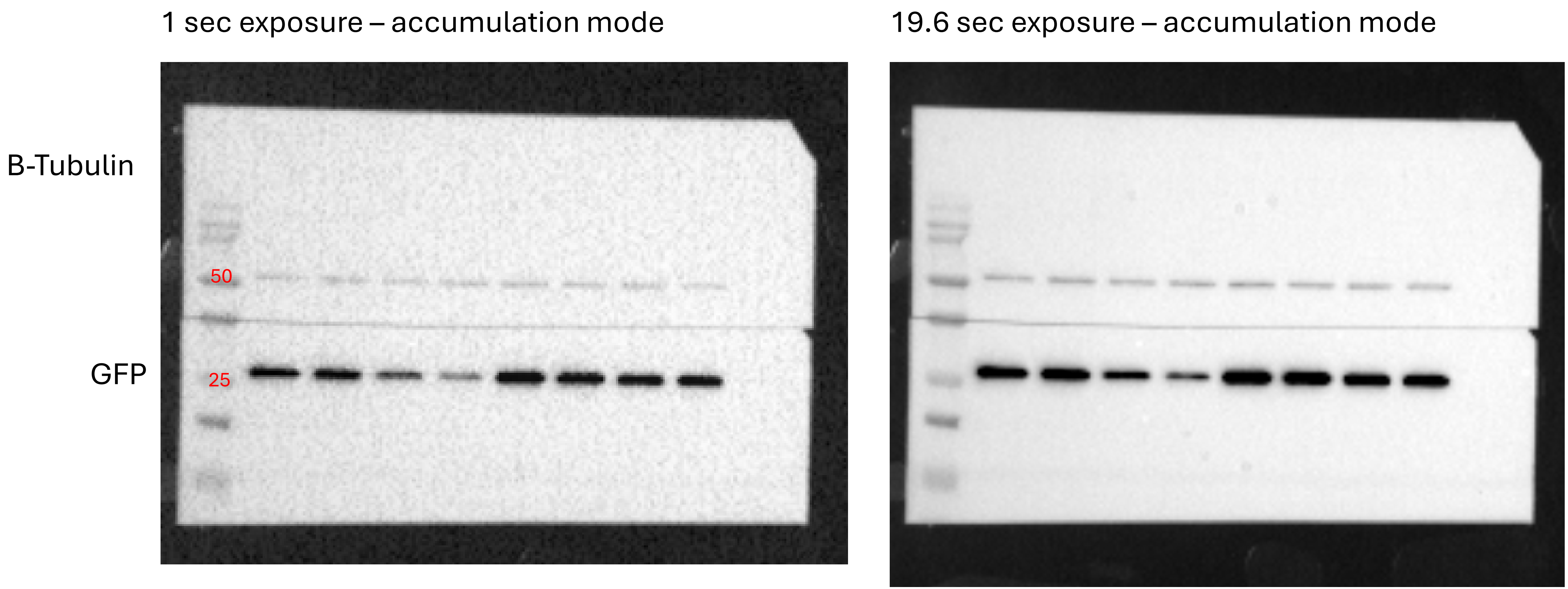

Supplementary Figure 2C

19.6 sec exposure – accumulation mode

B-Tubulin

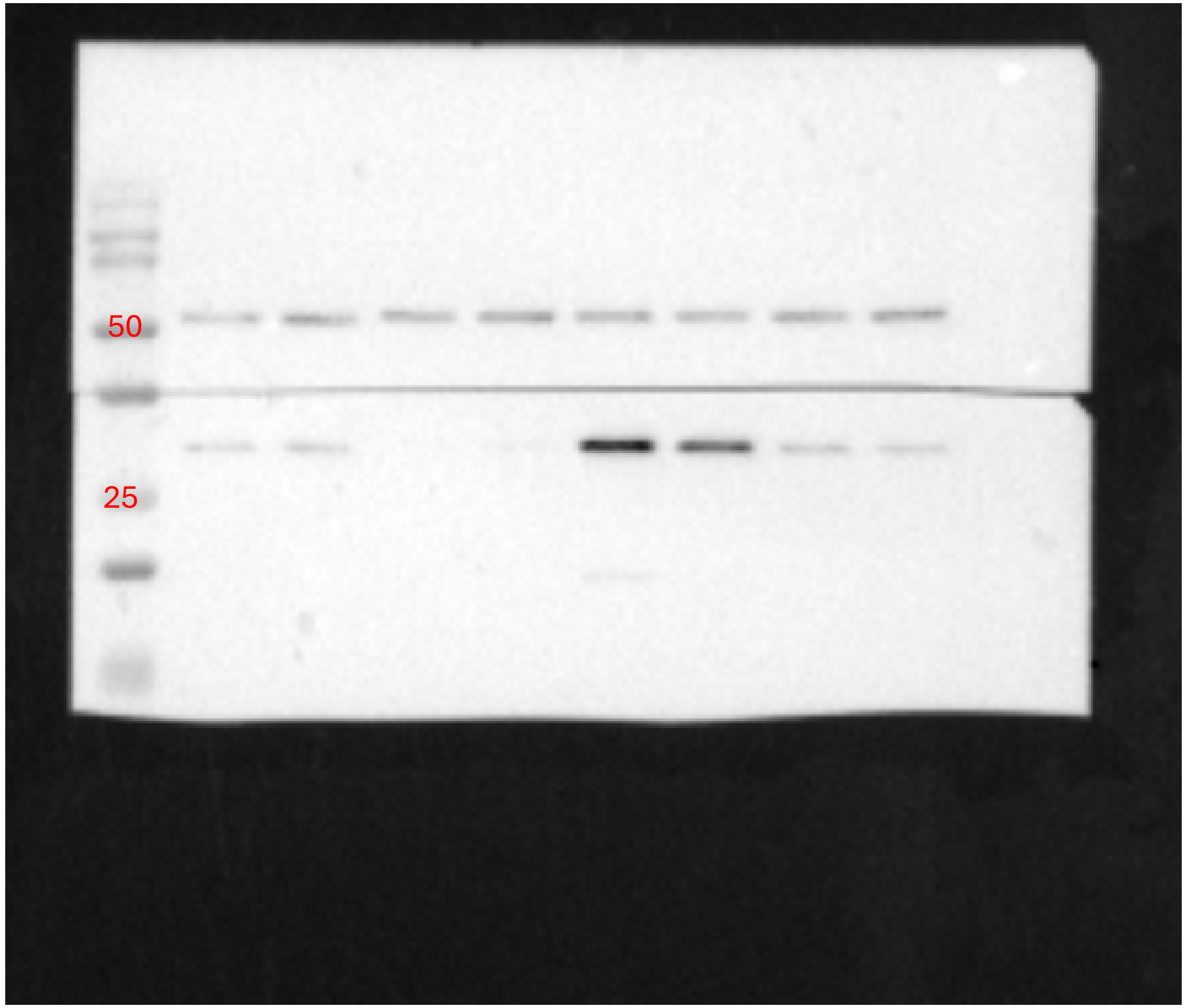

mCherry

Supplementary Figure 3A

ZFC3H1 (60 sec exposure – accumulation mode, max substrate)

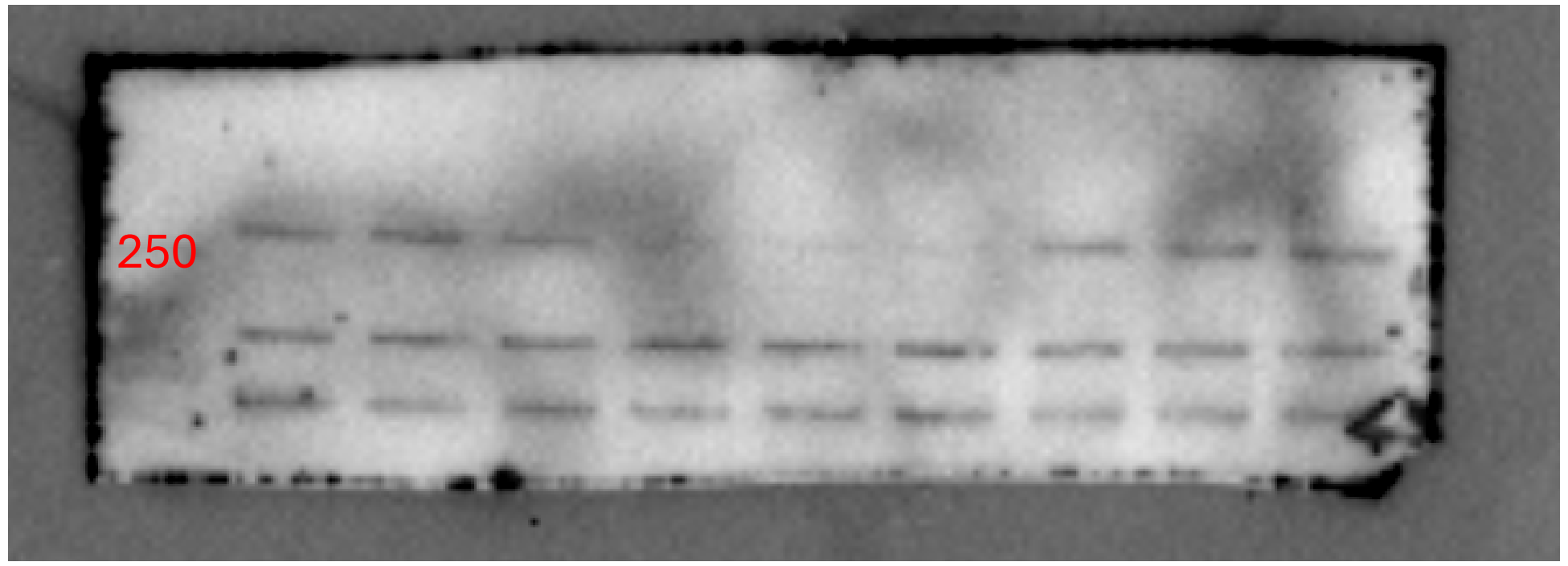

B-tubulin (16.5 sec exposure – accumulation mode)

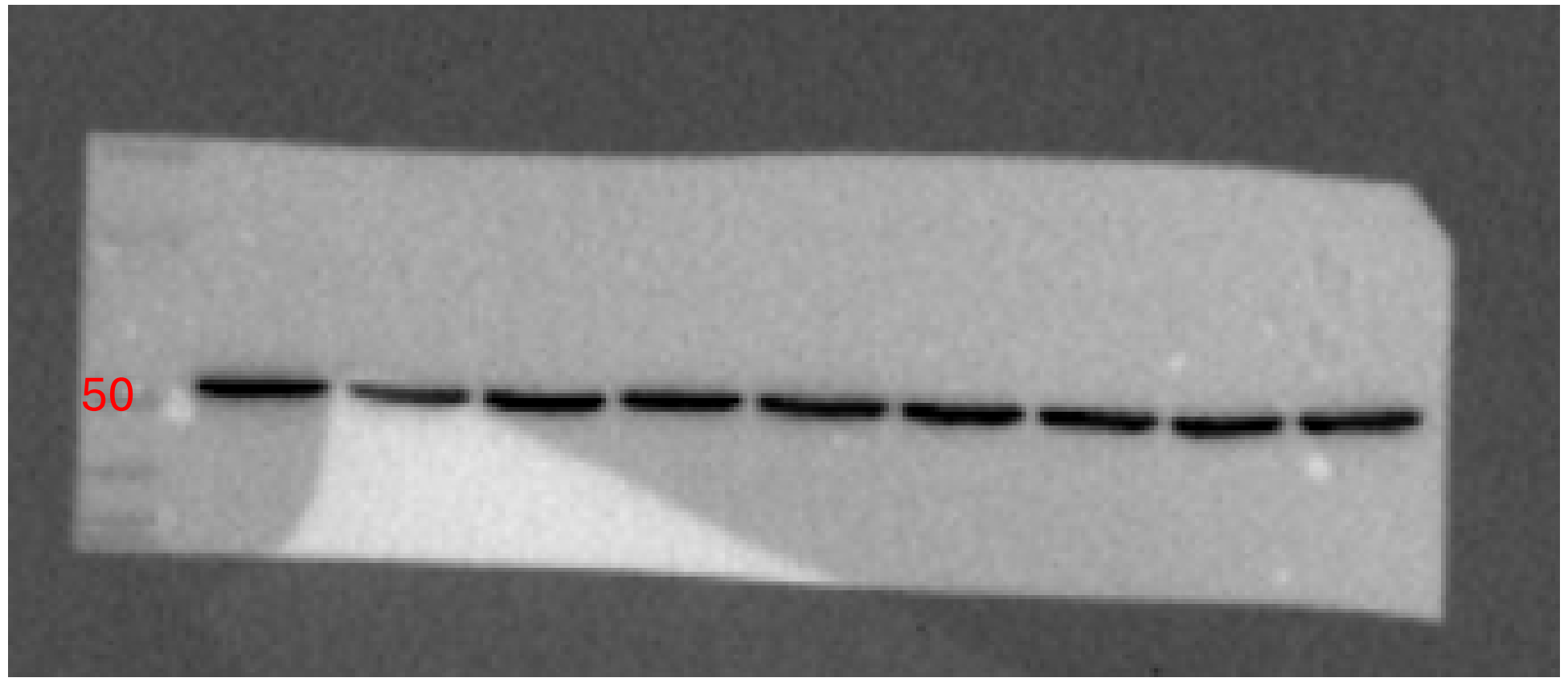

Lane	Sample
1	Ladder
2	Ctrl guide 1, Empty Vector
2	Ctrl guide 1, Empty Vector, rep 2
3	Ctrl guide 2, Empty Vector
6	ZFC3H1 guide 1, Empty Vector
6	ZFC3H1 guide 1, Empty Vector, rep 2
7	ZFC3H1 guide 2, Empty Vector
8	ZFC3H1 guide 1, FLAG-ZFC3H1
8	ZFC3H1 guide 1, FLAG-ZFC3H1, rep 2
9	ZFC3H1 guide 2, FLAG-ZFC3H1

Supplementary Figure 3A

GFP (19.6 sec exposure – accumulation mode, max substrate)

2.5 µg protein

5 µg protein

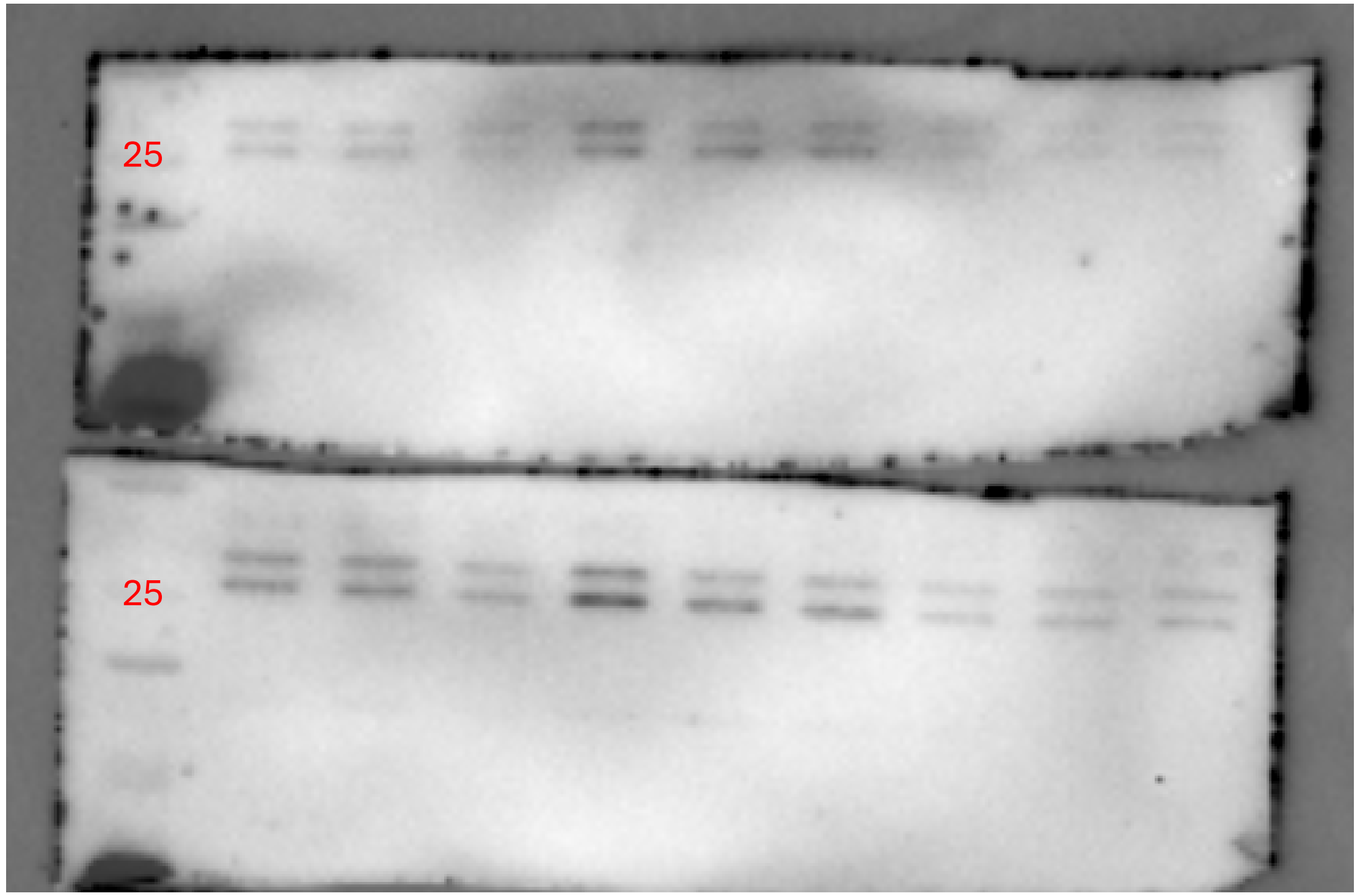

B-tubulin for GFP (16.5 sec exposure – accumulation mo

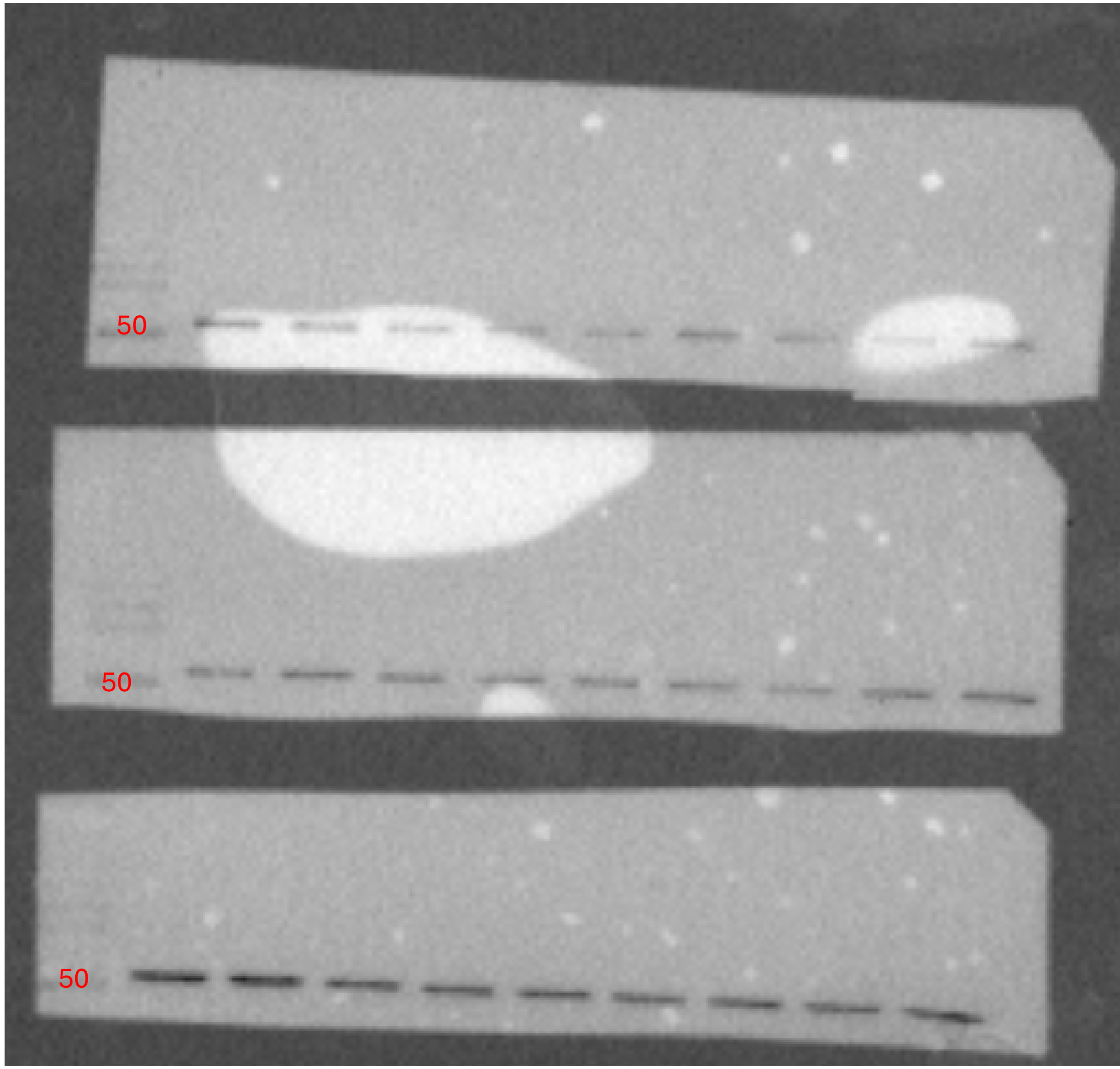

GFP (7.2 sec exposure – accumulation mode, max substrate)

10 µg protein

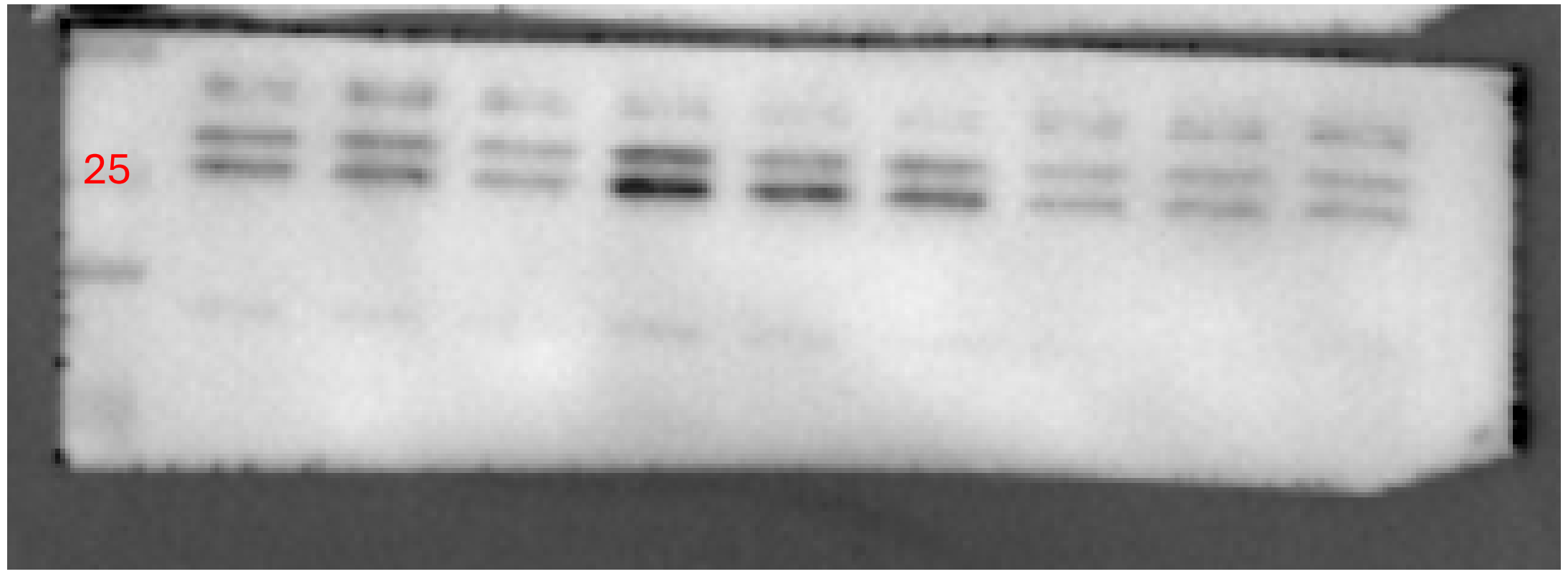

Supplementary Figure 3A

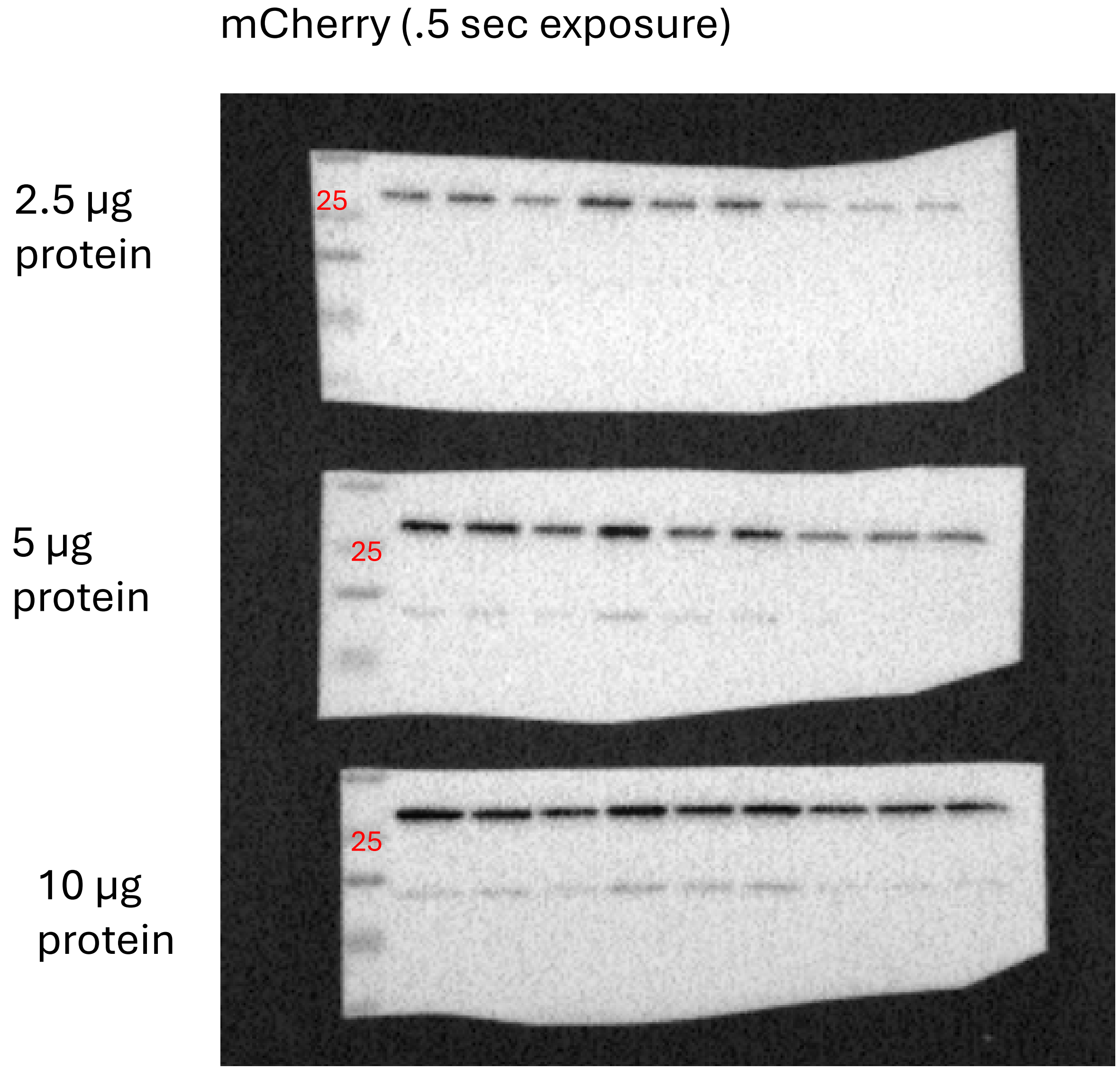

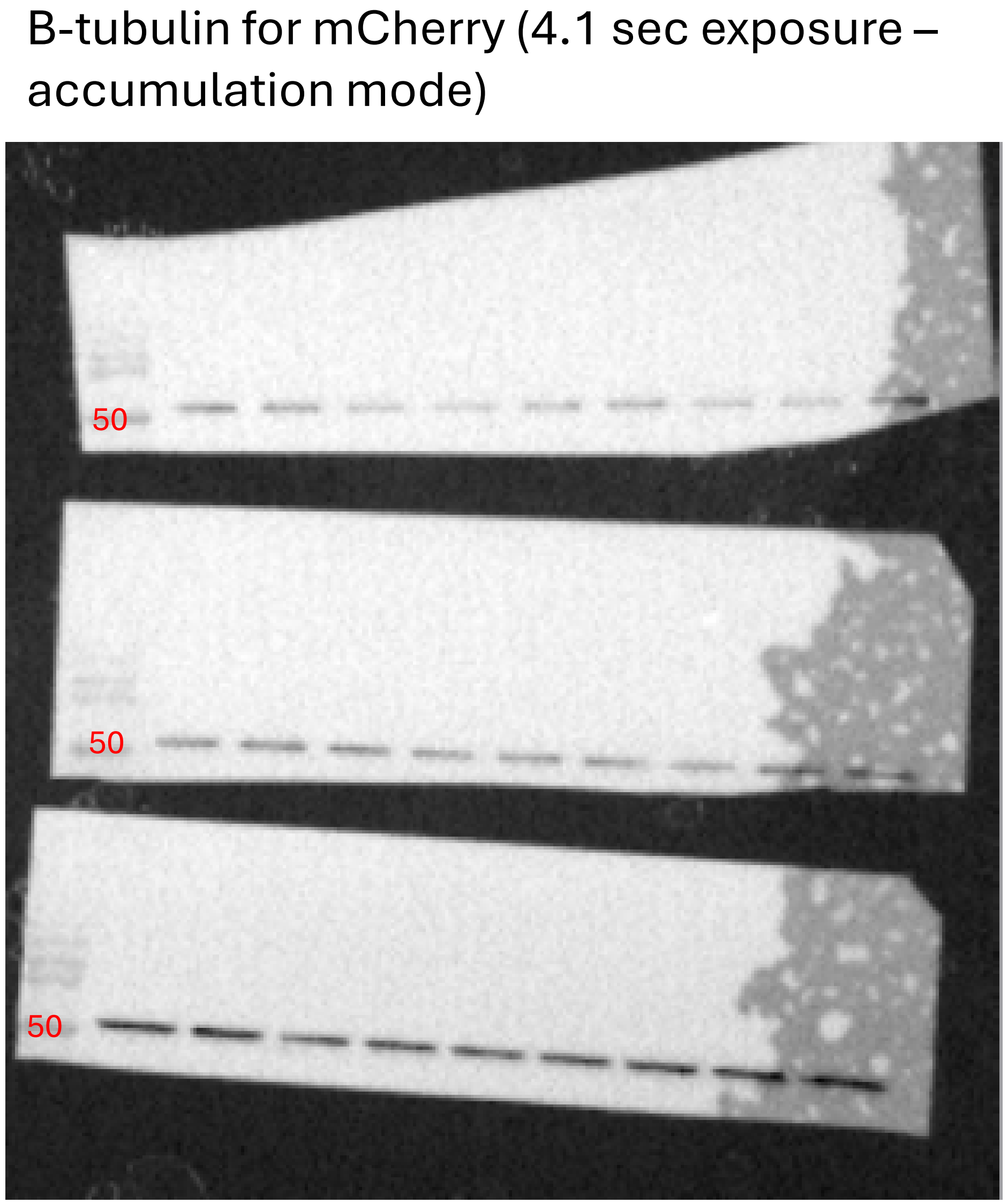

Supplementary Figure 4A

ponceau

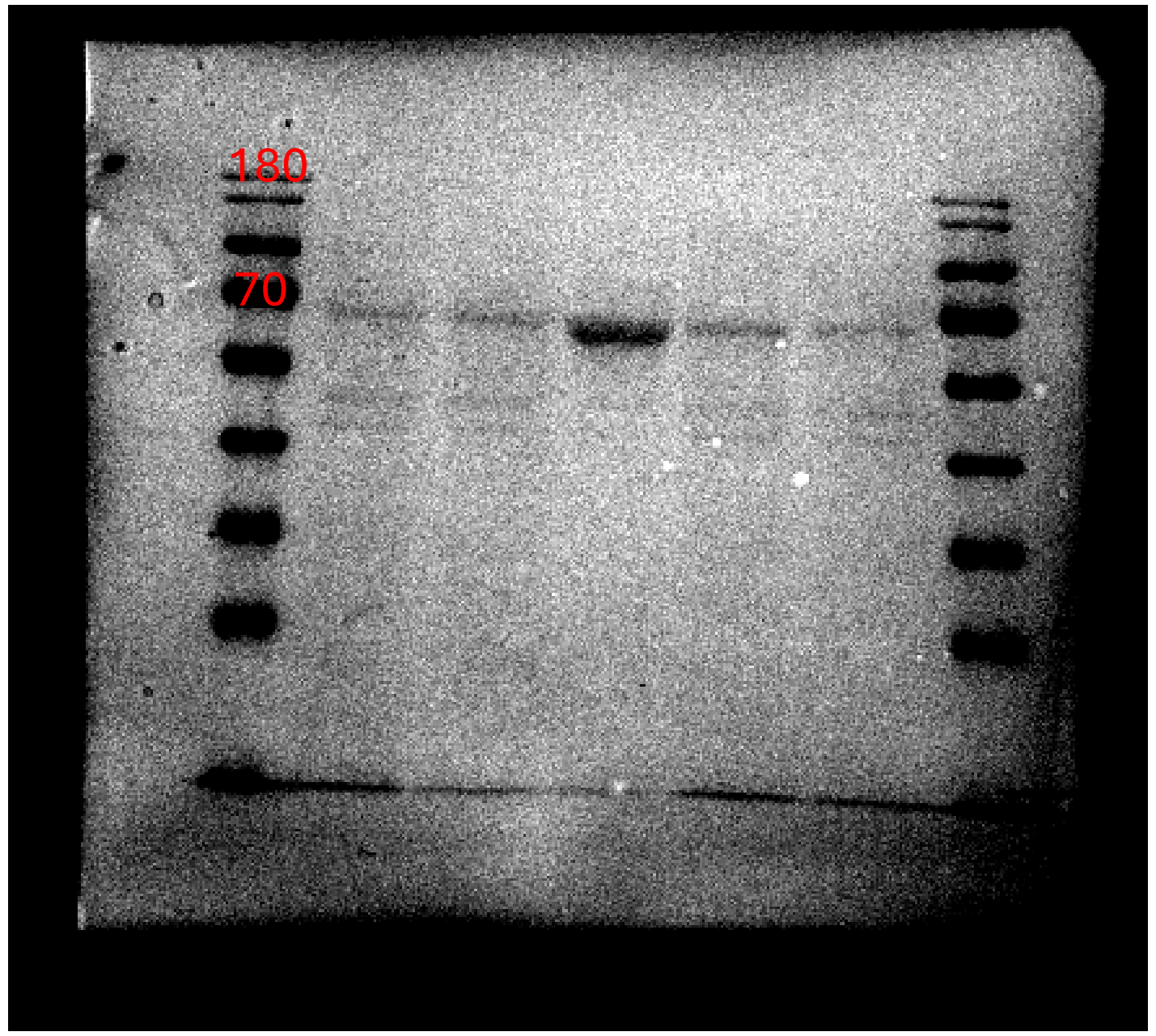

Puromycin (35.2 sec accum)

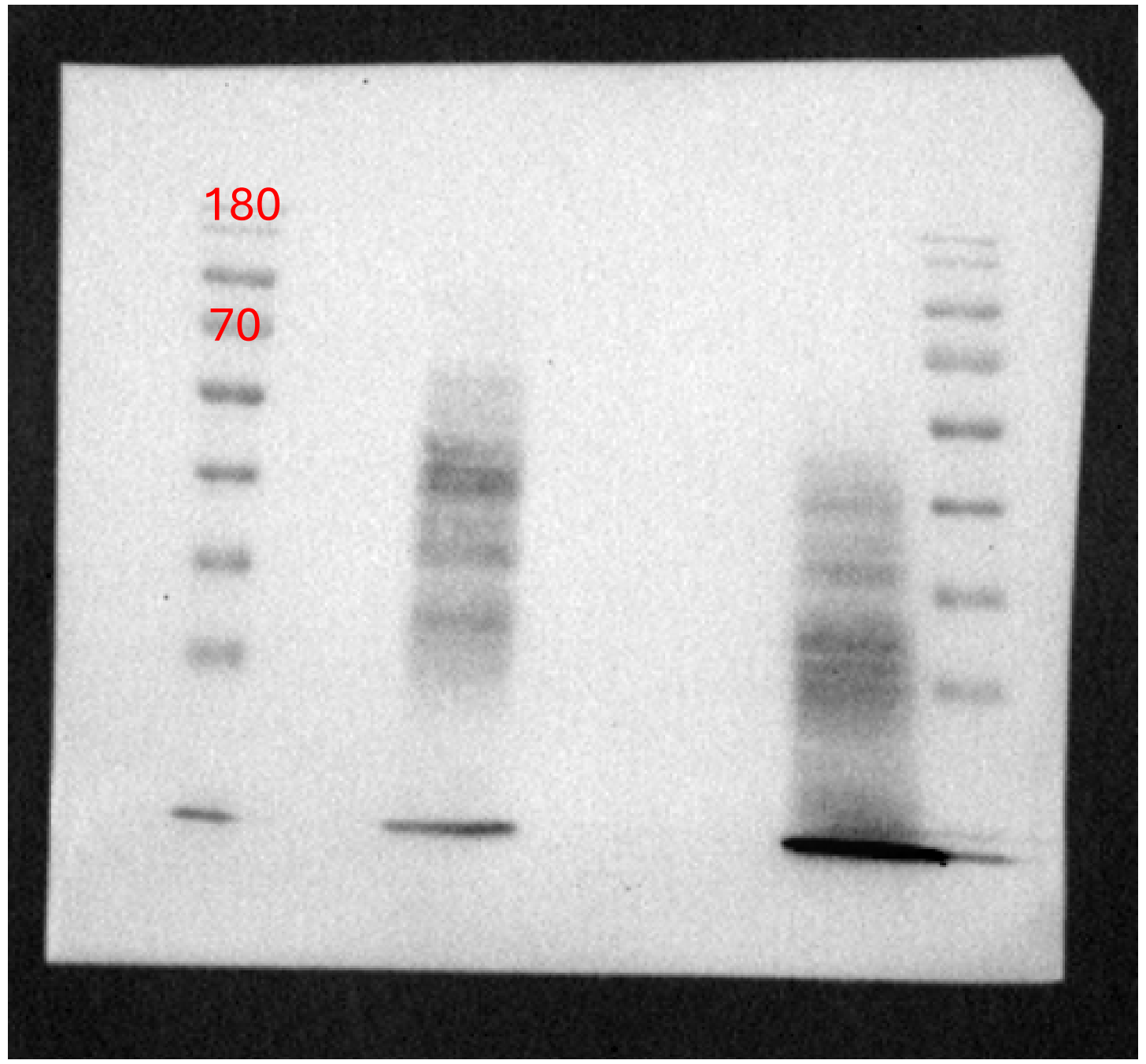

Lane	Sample
1	Ladder
2	Ctrl guide, no puro
3	Ctrl guide, puro
4	Ctrl guide, puro plus chx
5	Ctrl siRNA, no puro
6	Ctrl siRNA, puro
7	Ladder

Supplementary Figure 4B

ZFC3H1 (44.5 sec accum mode, max substrate)

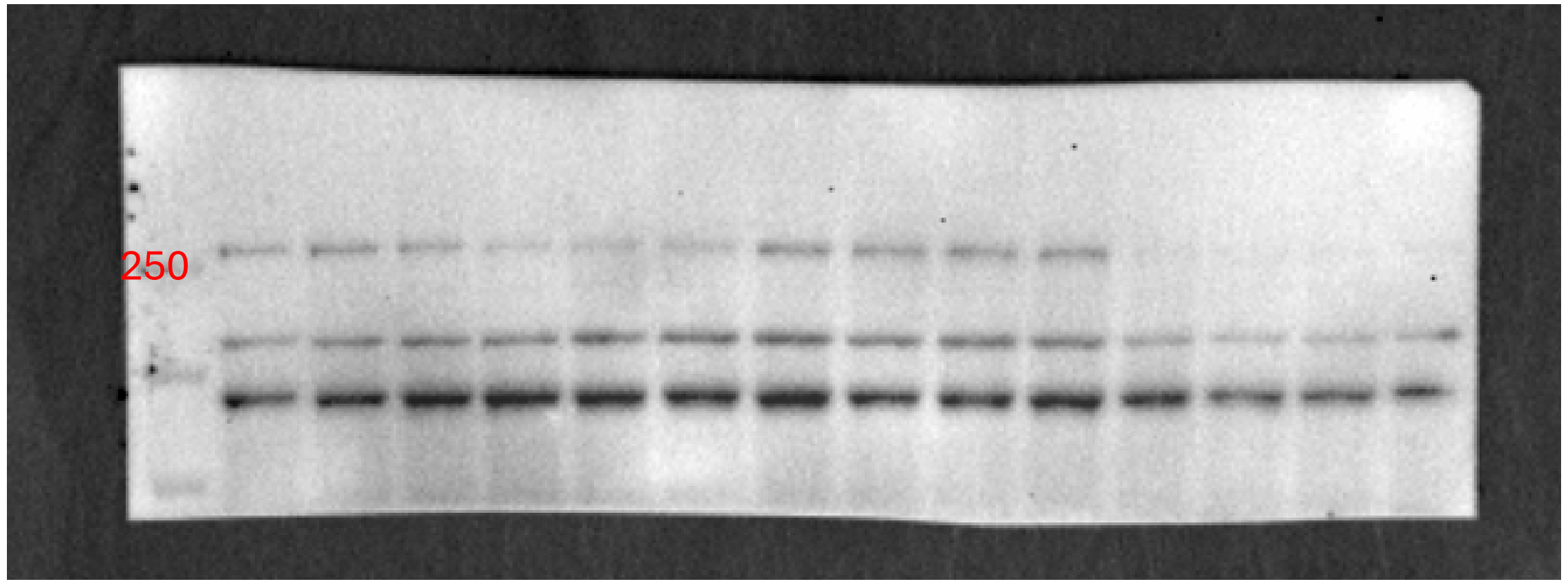

B-tubulin (4.1 sec accum mode)

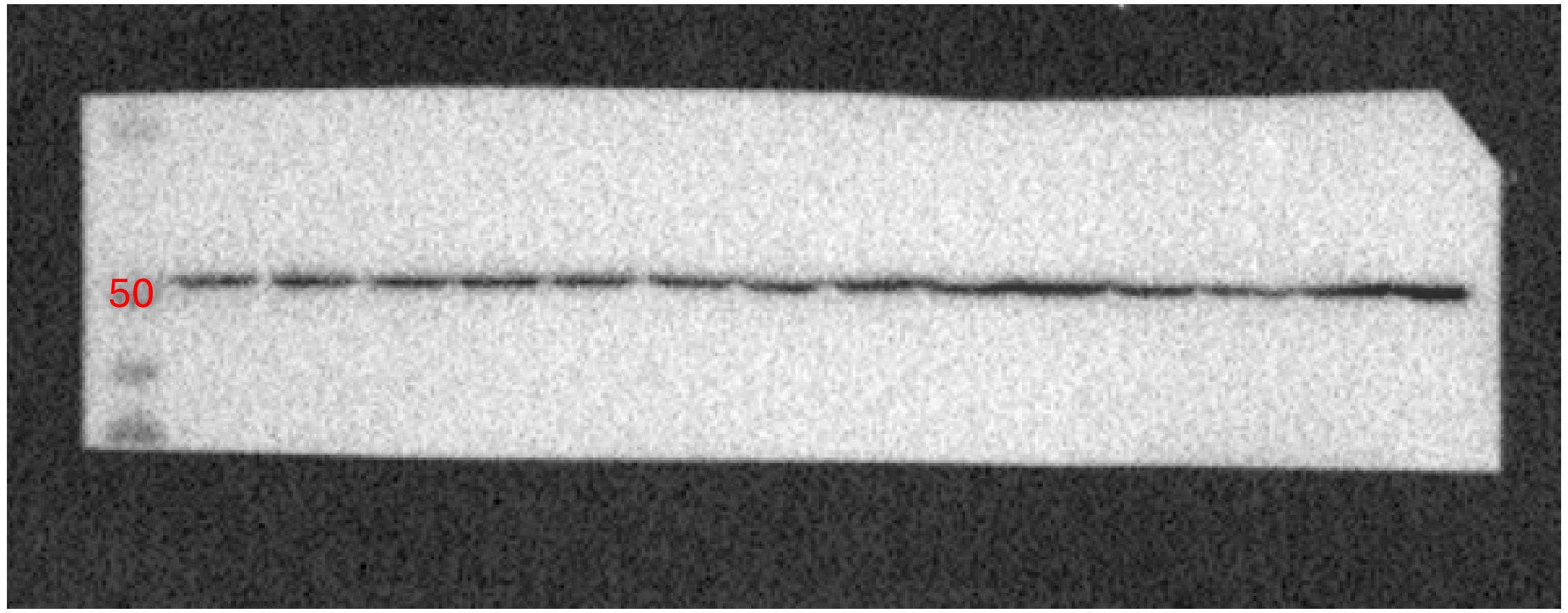

Lane	Sample
1	Ladder
2-4	Ctrl siRNA
5-7	ZFC3H1 siRNA
8-9	Ctrl guide 1
10-11	Ctrl guide 2
12-13	ZFC3H1 guide 1
14-15	ZFC3H1 guide 2

Supplementary Figure 4B

ponceau

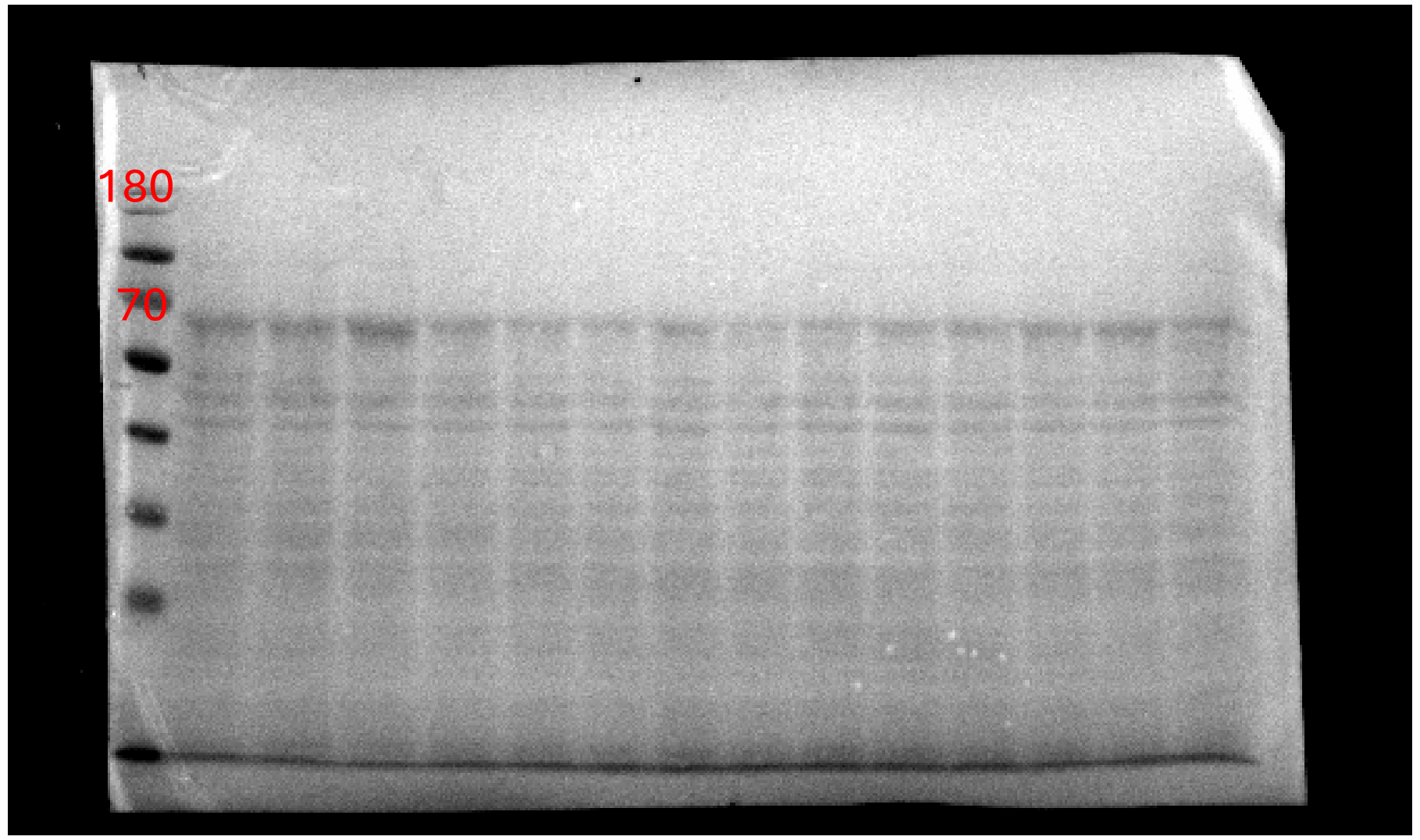

Puromycin (22.7 sec accum)

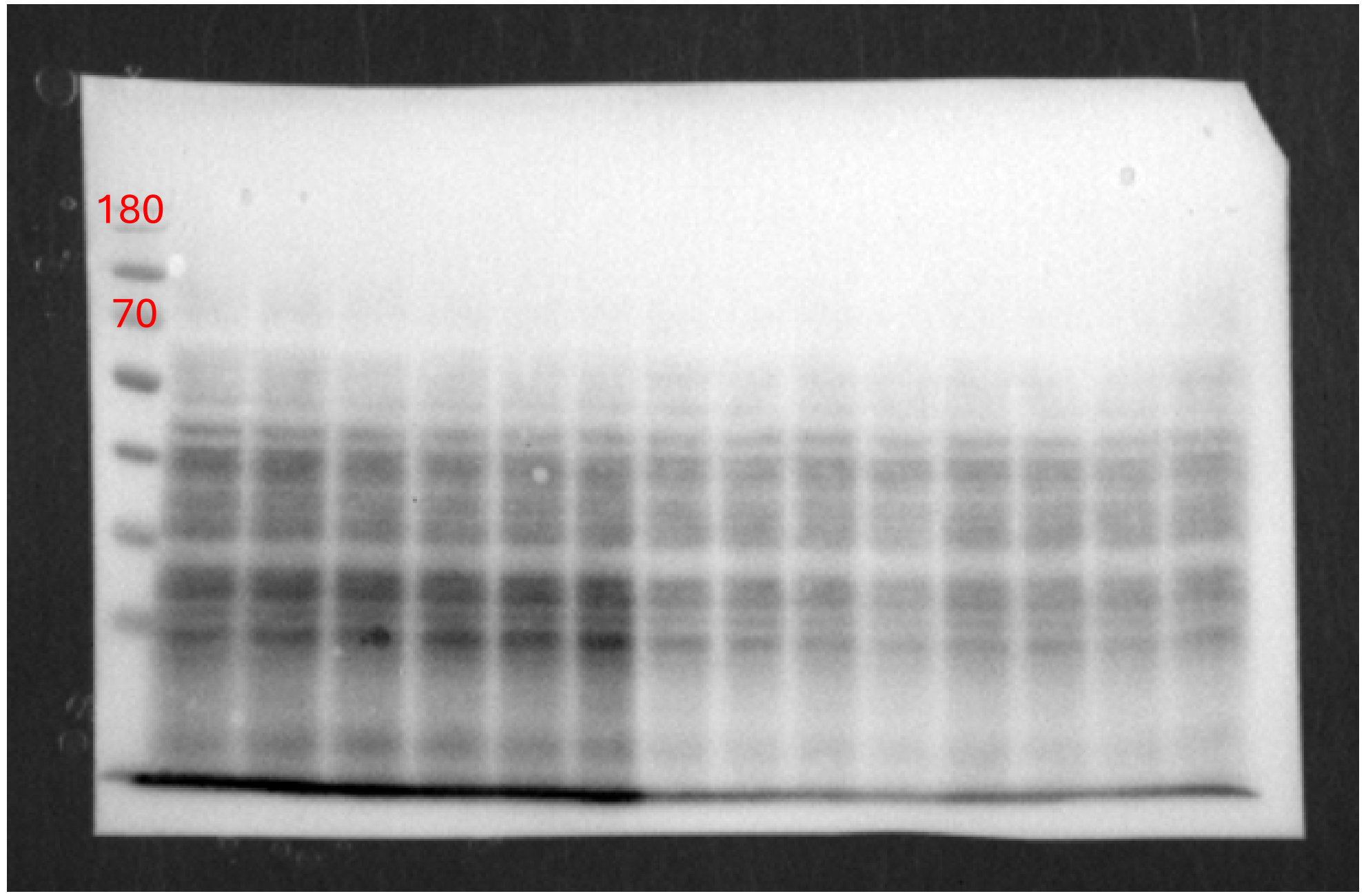

Supplementary Figure 5A

ZFC3H1 13.1 sec exposure – accumulation mode (max substrate)

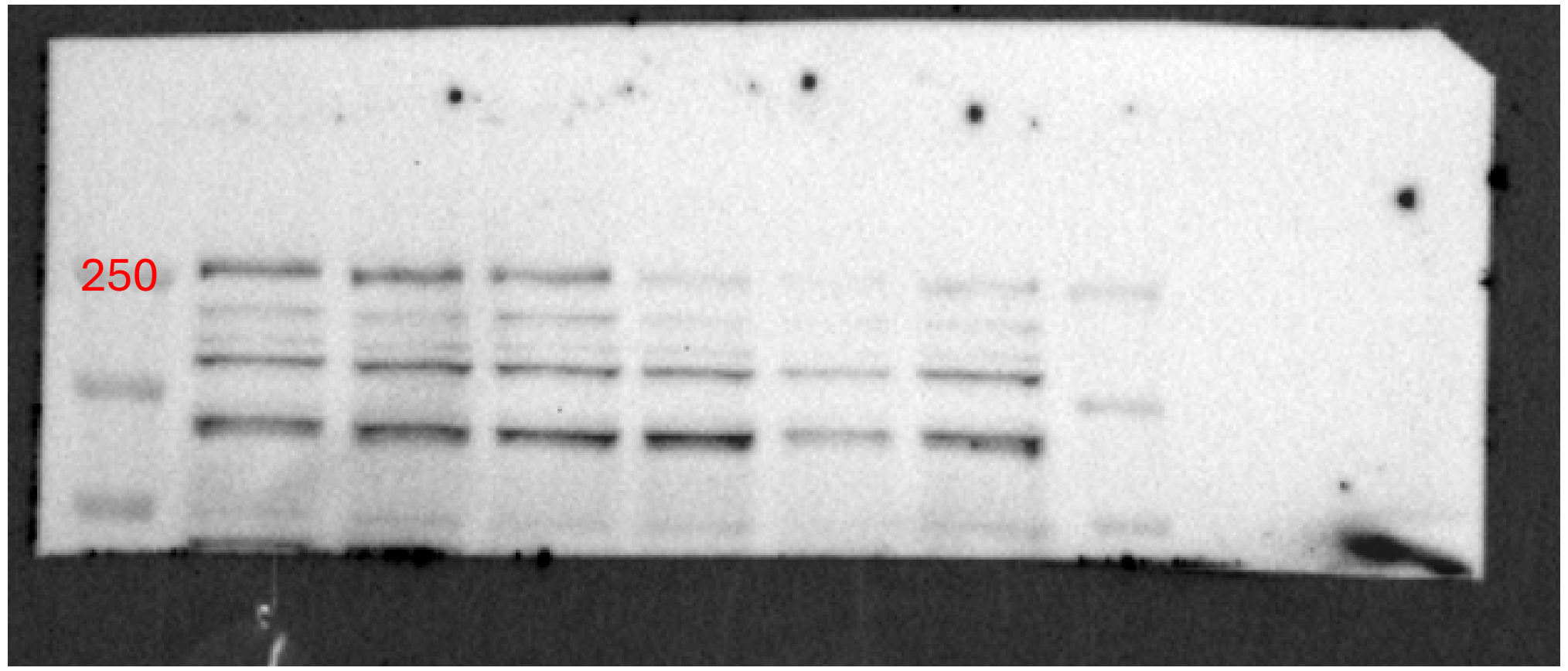

B-tubulin 4.1 sec exposure – accumulation mode

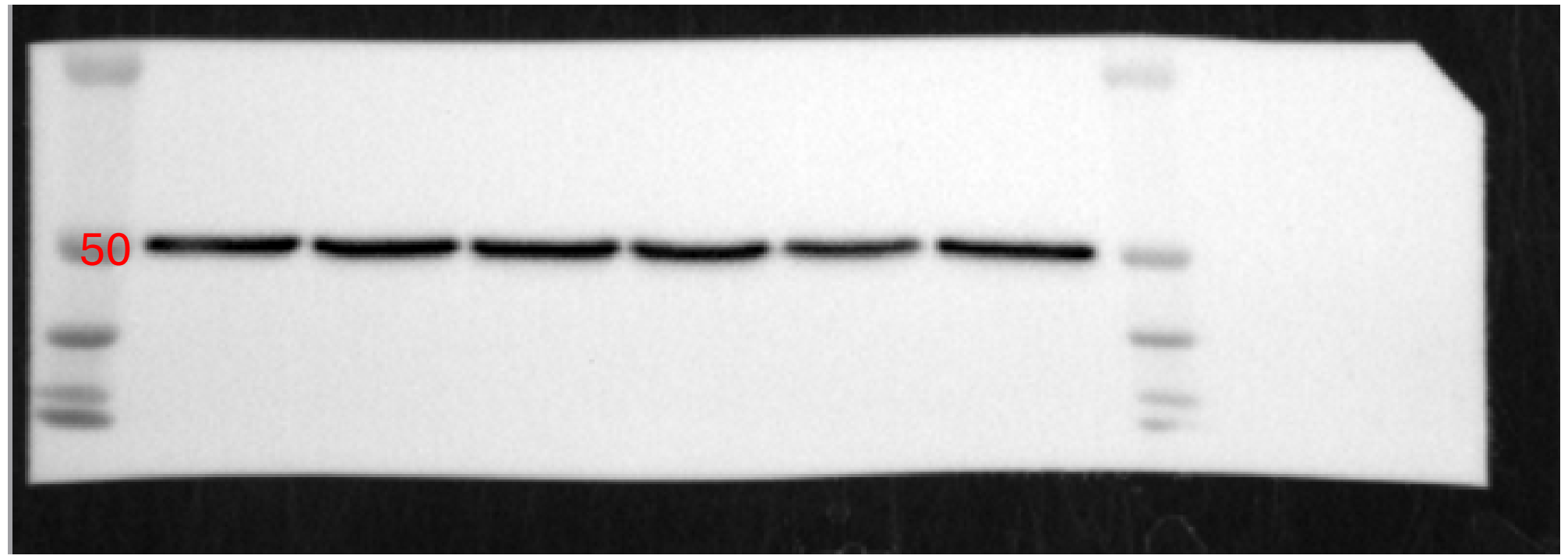

Lane	Sample
1	Ladder
2	Ctrl guide 1
3	Ctrl guide 1
4	Ctrl guide 2
5	ZFC3H1 guide 1
6	ZFC3H1 guide 1
7	ZFC3H1 guide 2
8	ladder

Supplementary Figure -6A

ZFC3H1 13.4 sec exposure – accumulation mode (max substrate)

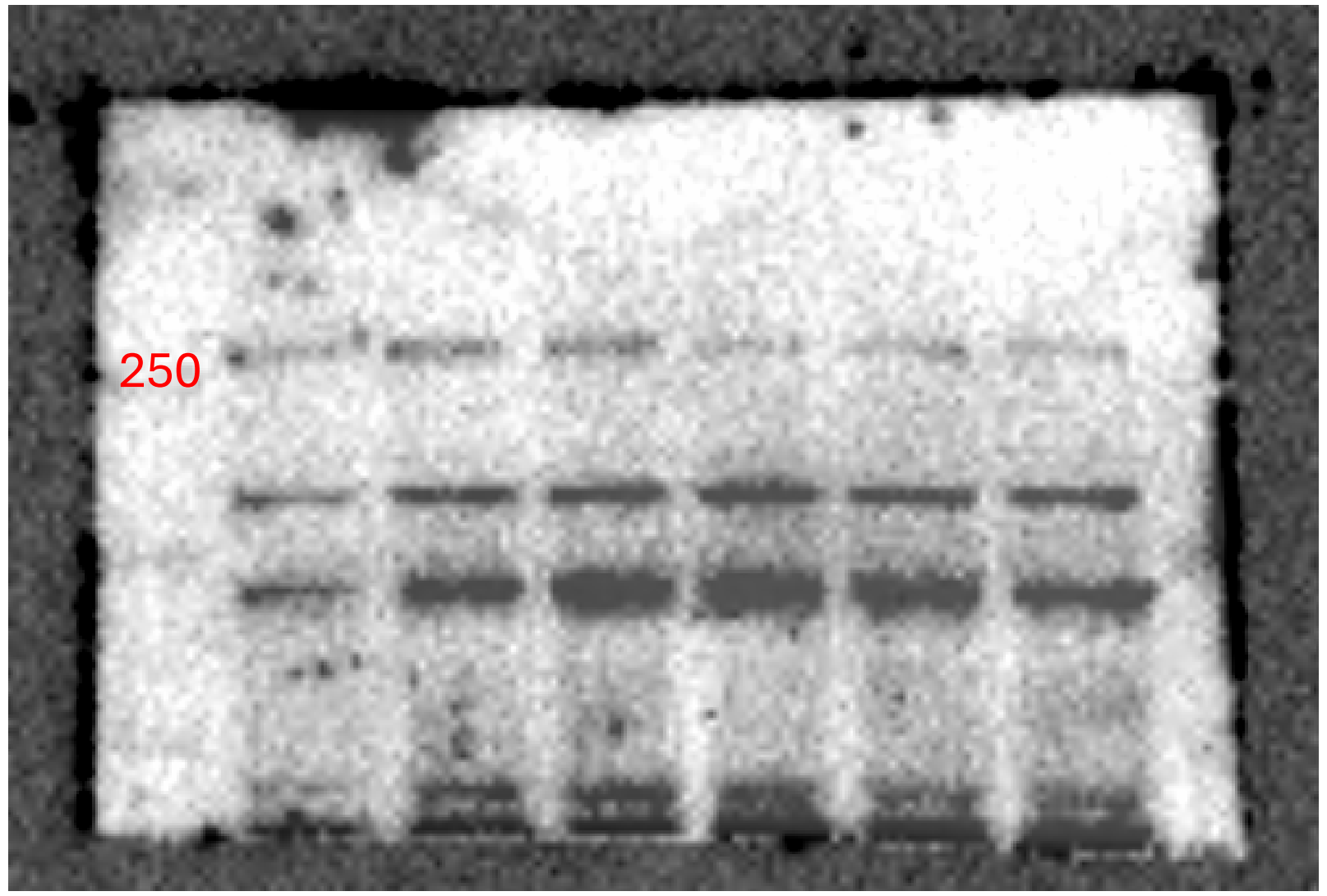

B-tubulin 1 sec exposure – accumulation mode

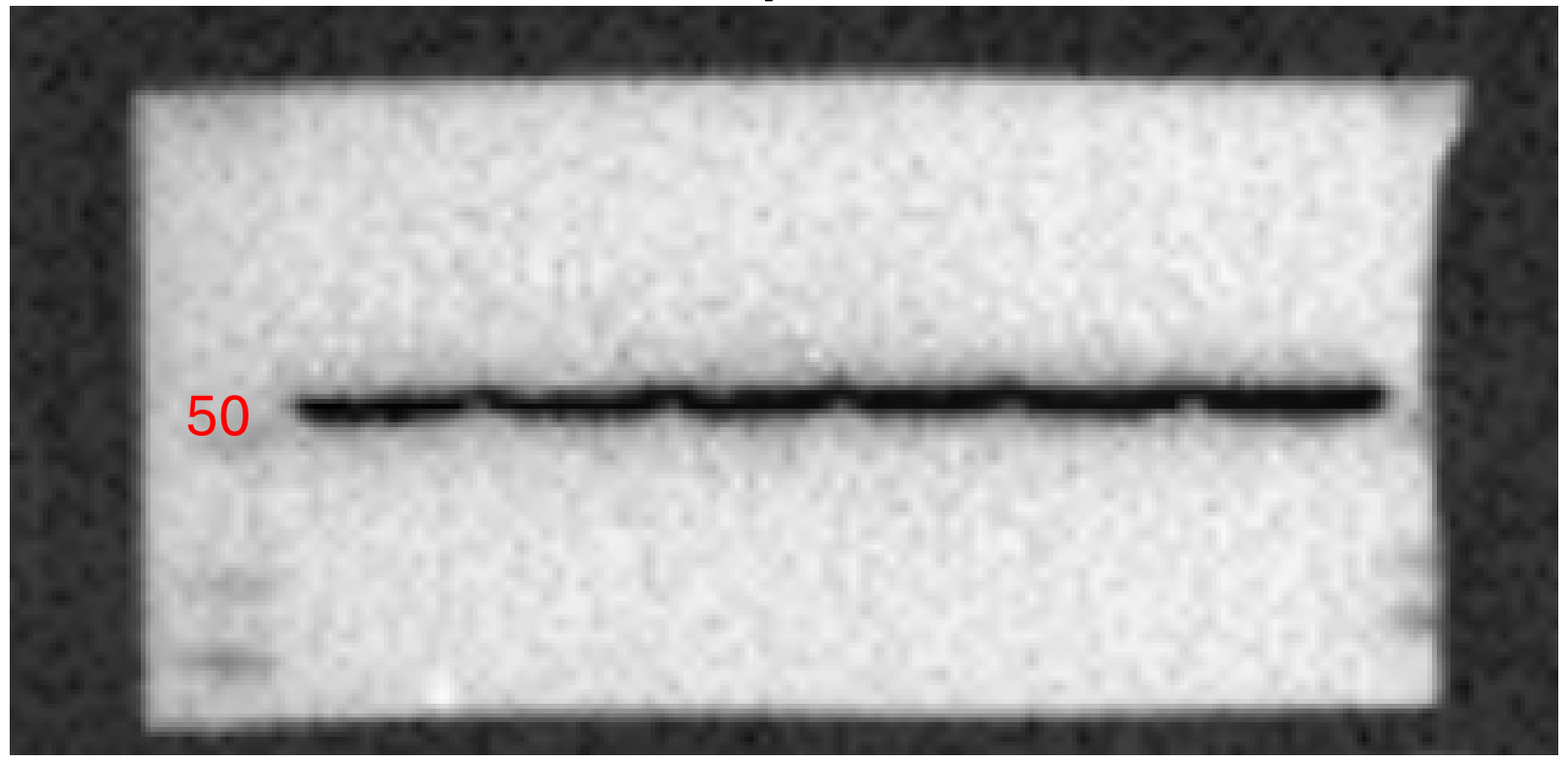

Lane	Sample
1	Ladder
2	Ctrl siRNA
3	Ctrl siRNA
4	Ctrl siRNA
5	ZFC3H1 siRNA
6	ZFC3H1 siRNA
7	ZFC3H1 siRNA

Supplementary Figure 6C

B-Tubulin for GFP (1 sec exposure – accumulation mode)

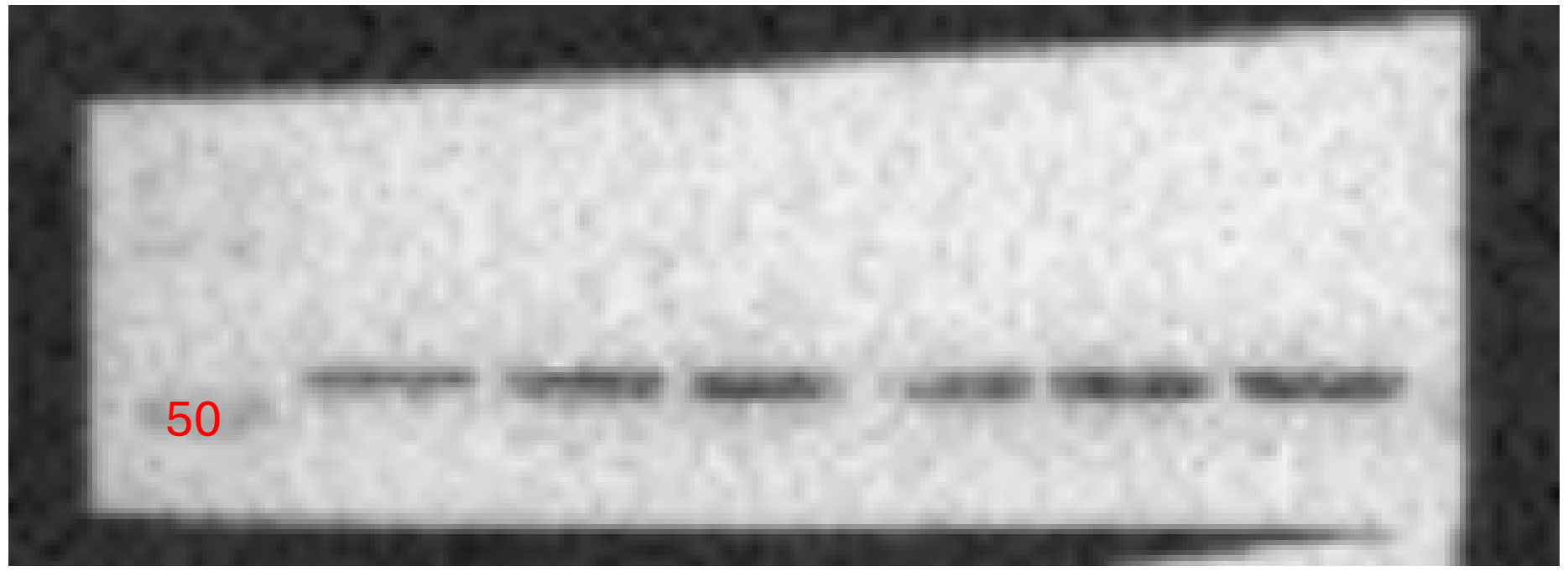

B-Tubulin for mCherry (1 sec exposure – accumulation mode)

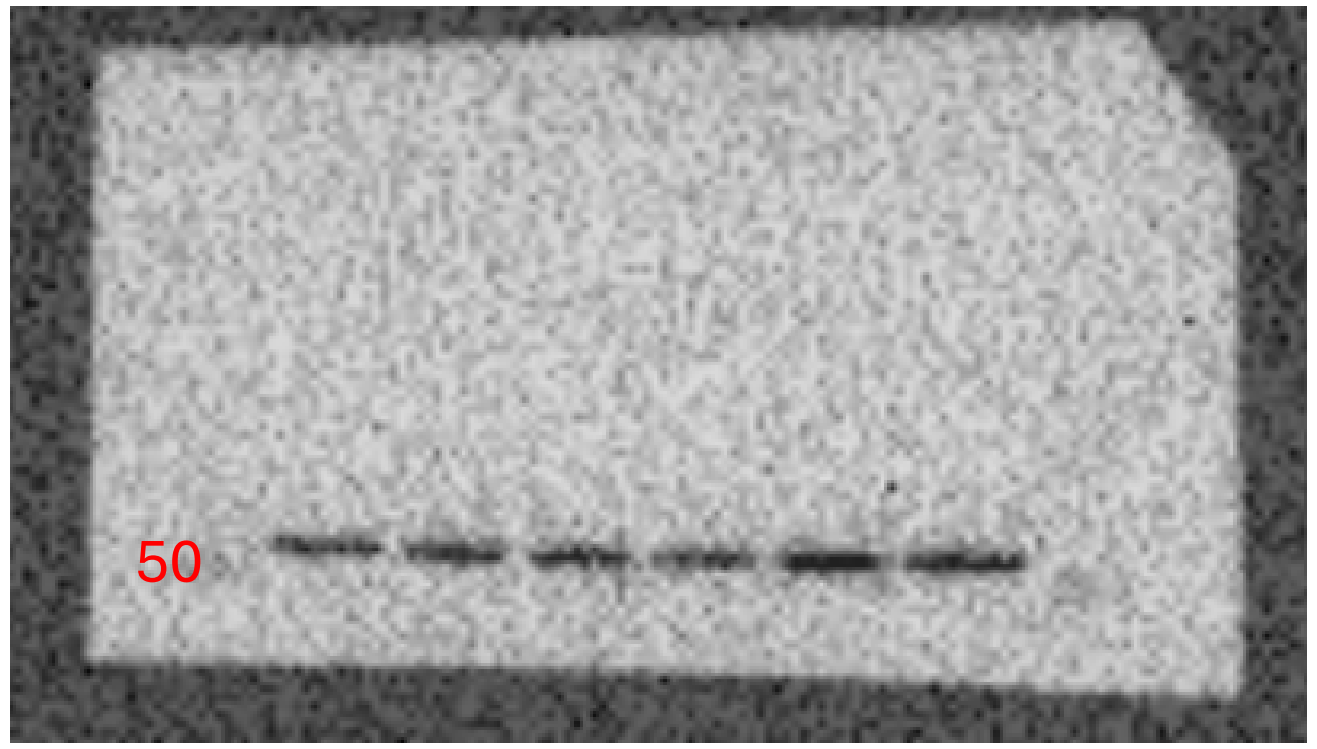

GFP (25.8 sec exposure – accumulation mode), max substrate

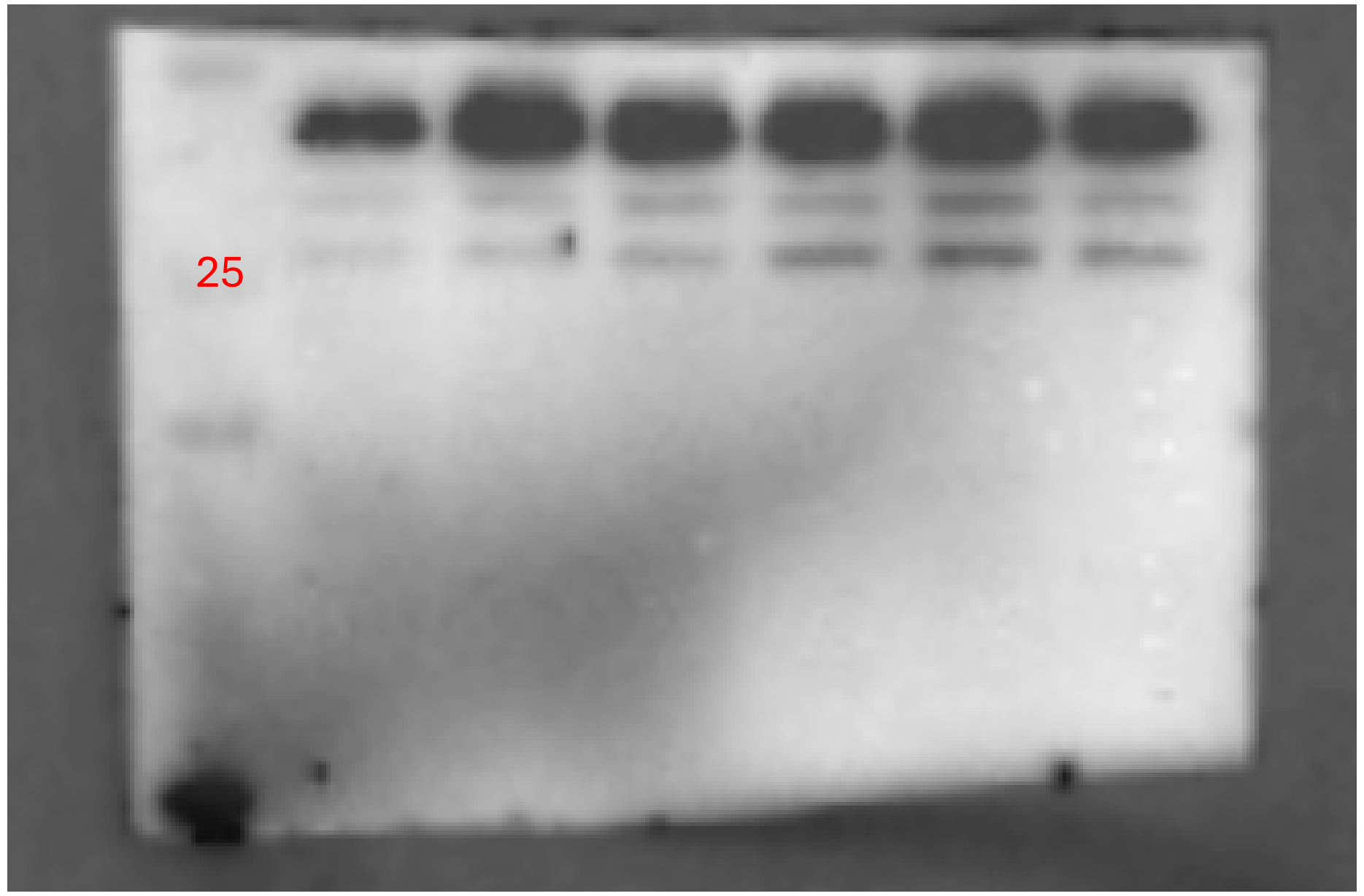

mCherry (1 sec exposure)

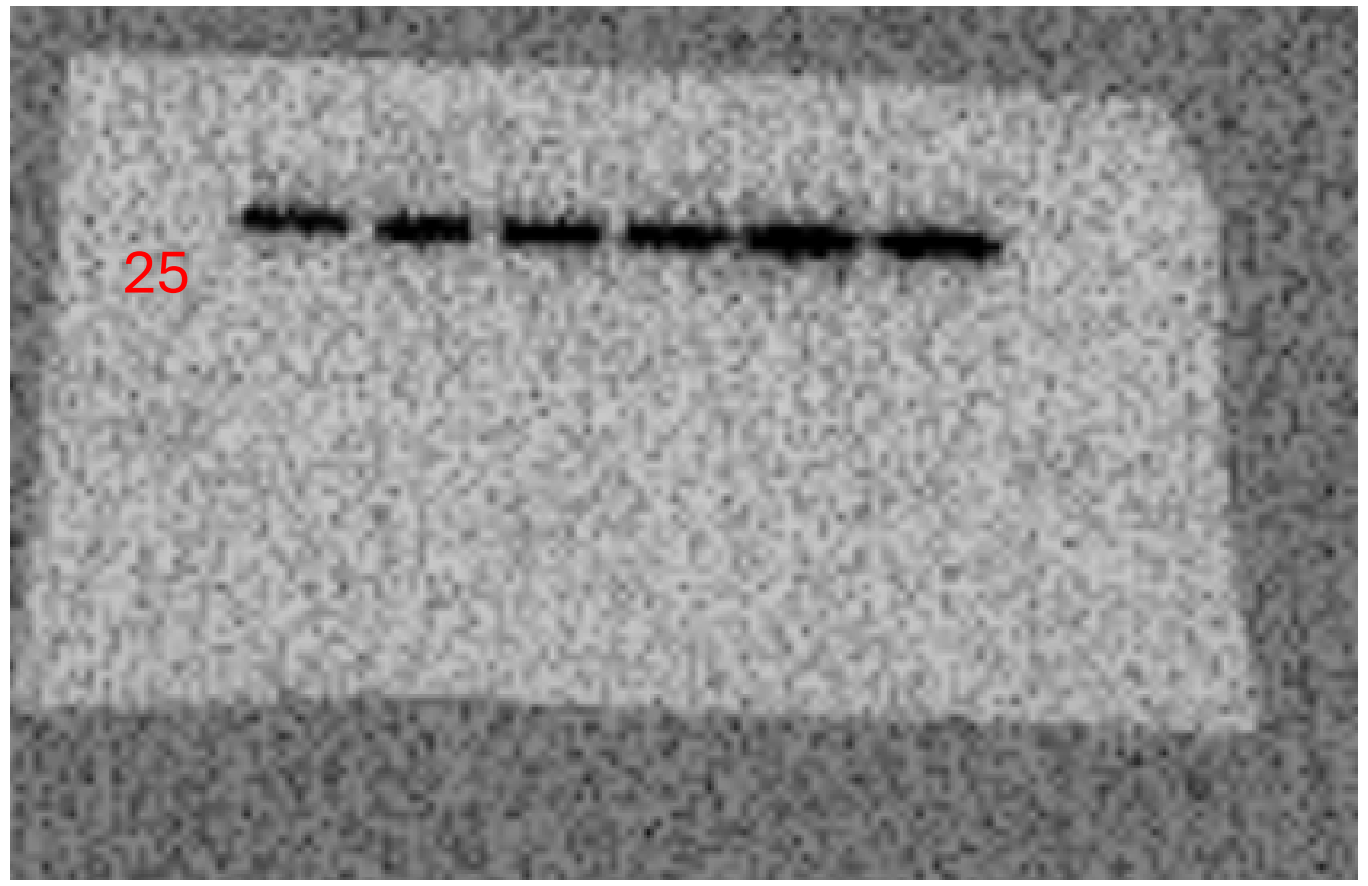

Supplementary Figure 6D

B-Tubulin for mCherry (1 sec exposure – accumulation mode)

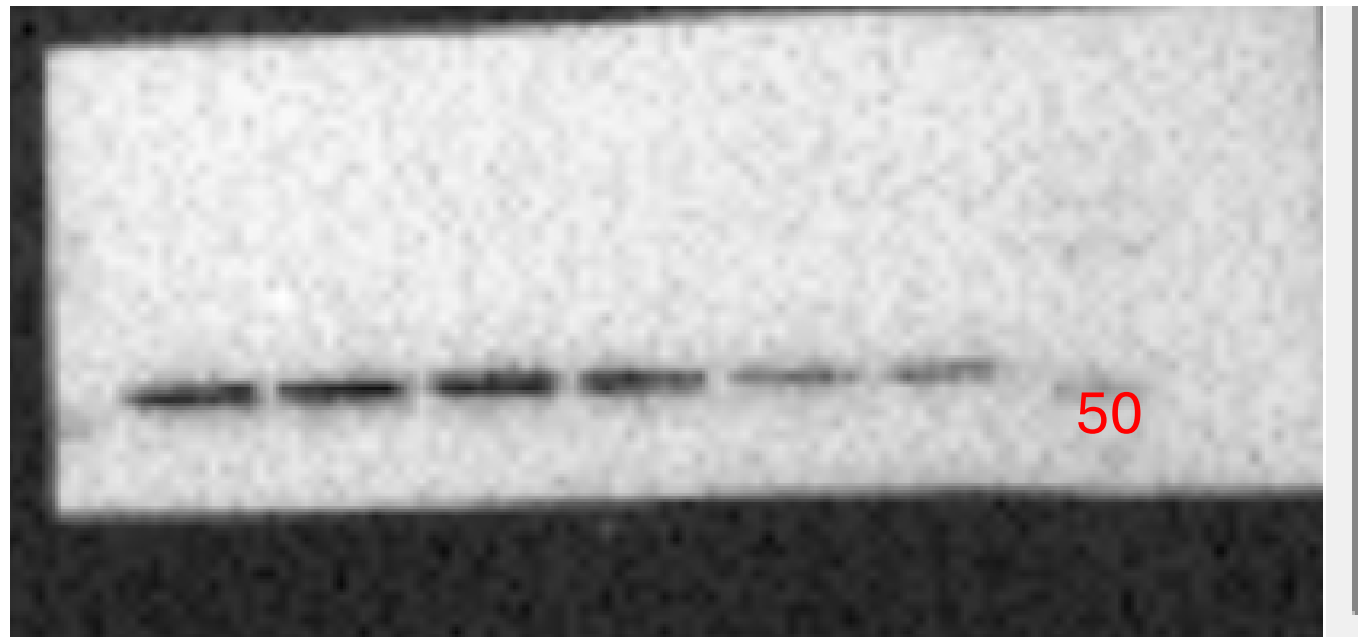

B-Tubulin for GFP (1 sec exposure – accumulation mode)

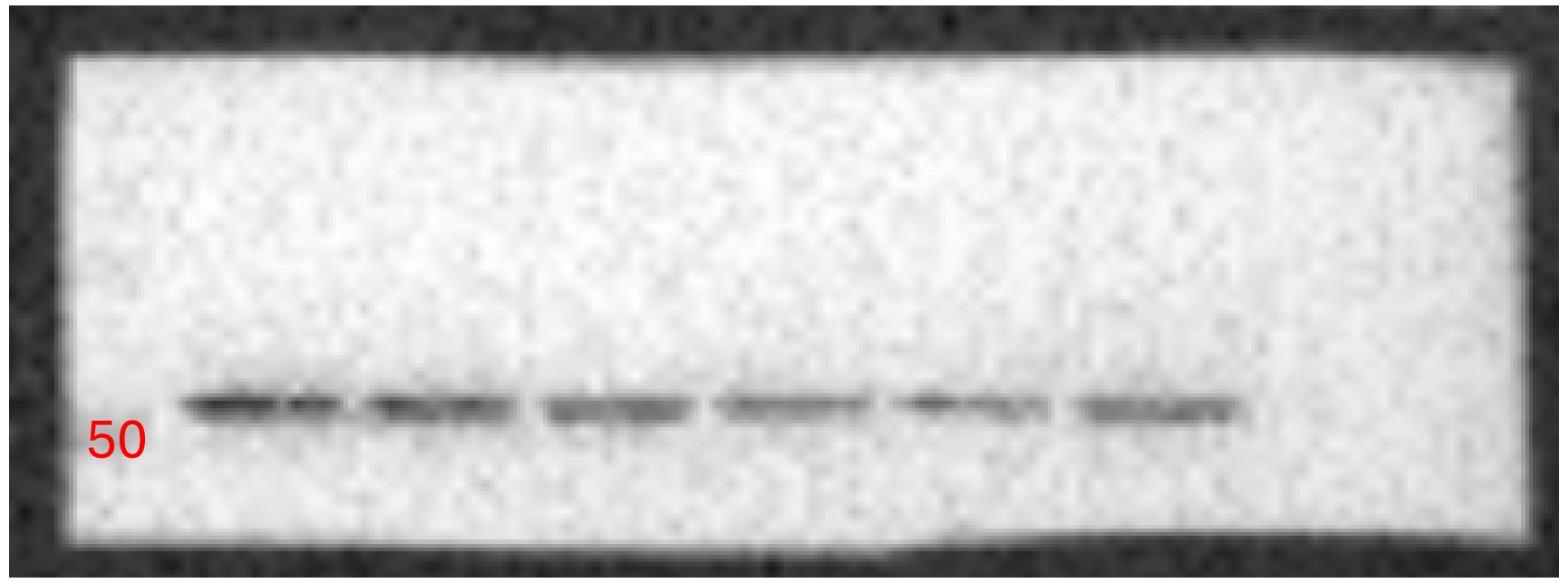

mCherry (25.8 sec exposure – accumulation mode), max substrate

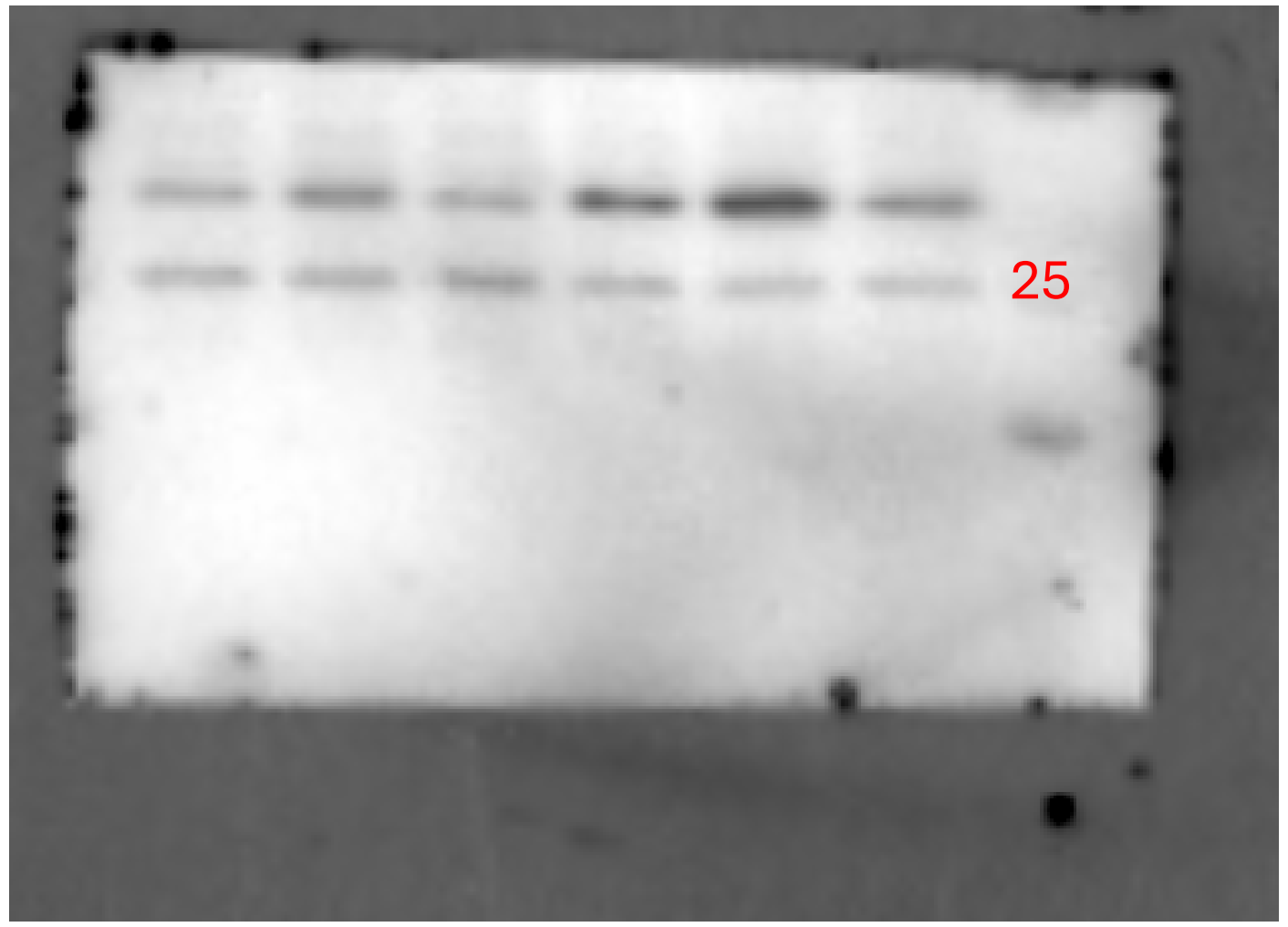

GFP (1 sec exposure – accumulation mode)

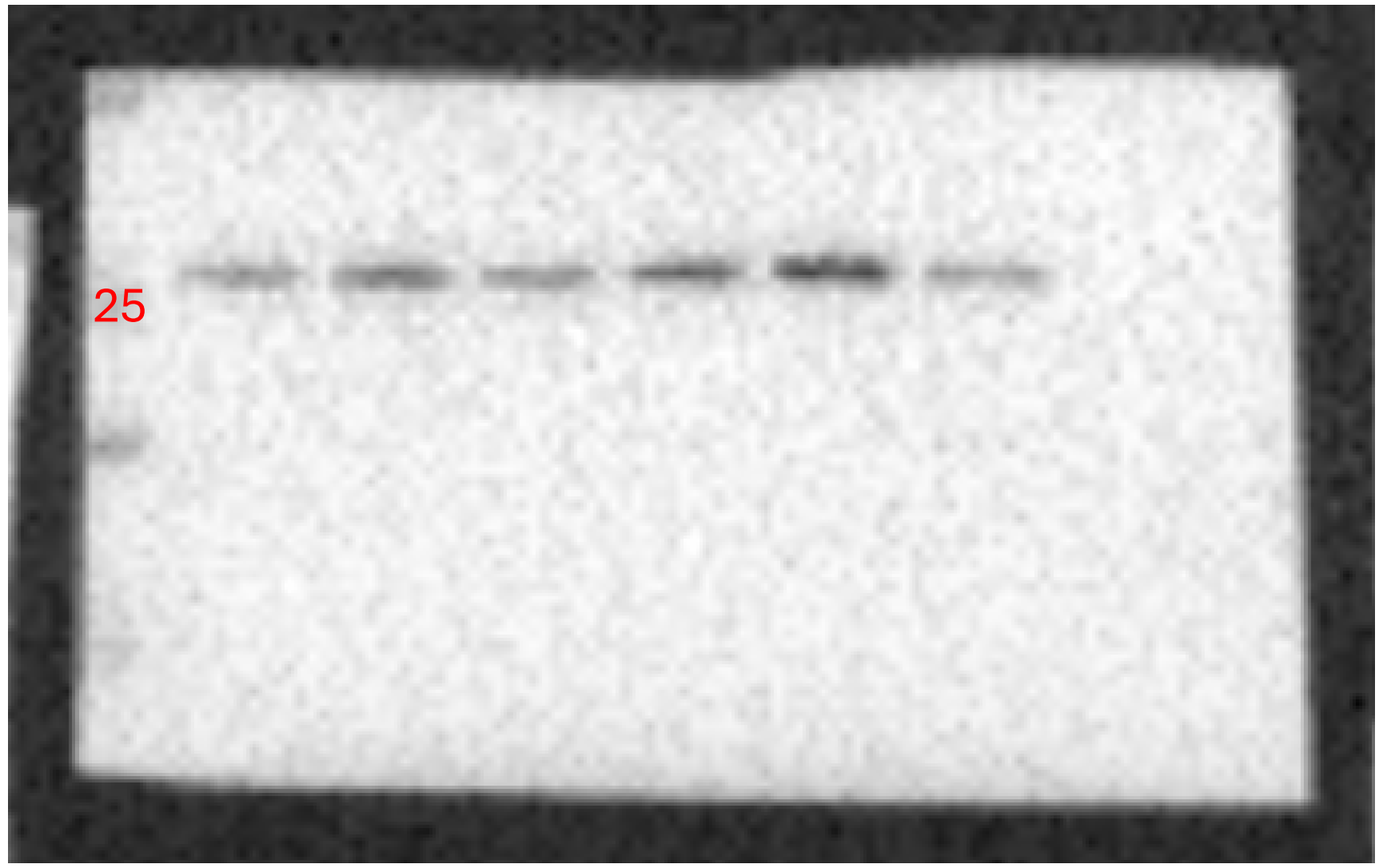

Supplementary Figure 7A

ZFC3H1 4.1 sec exposure – accumulation mode (max substrate)

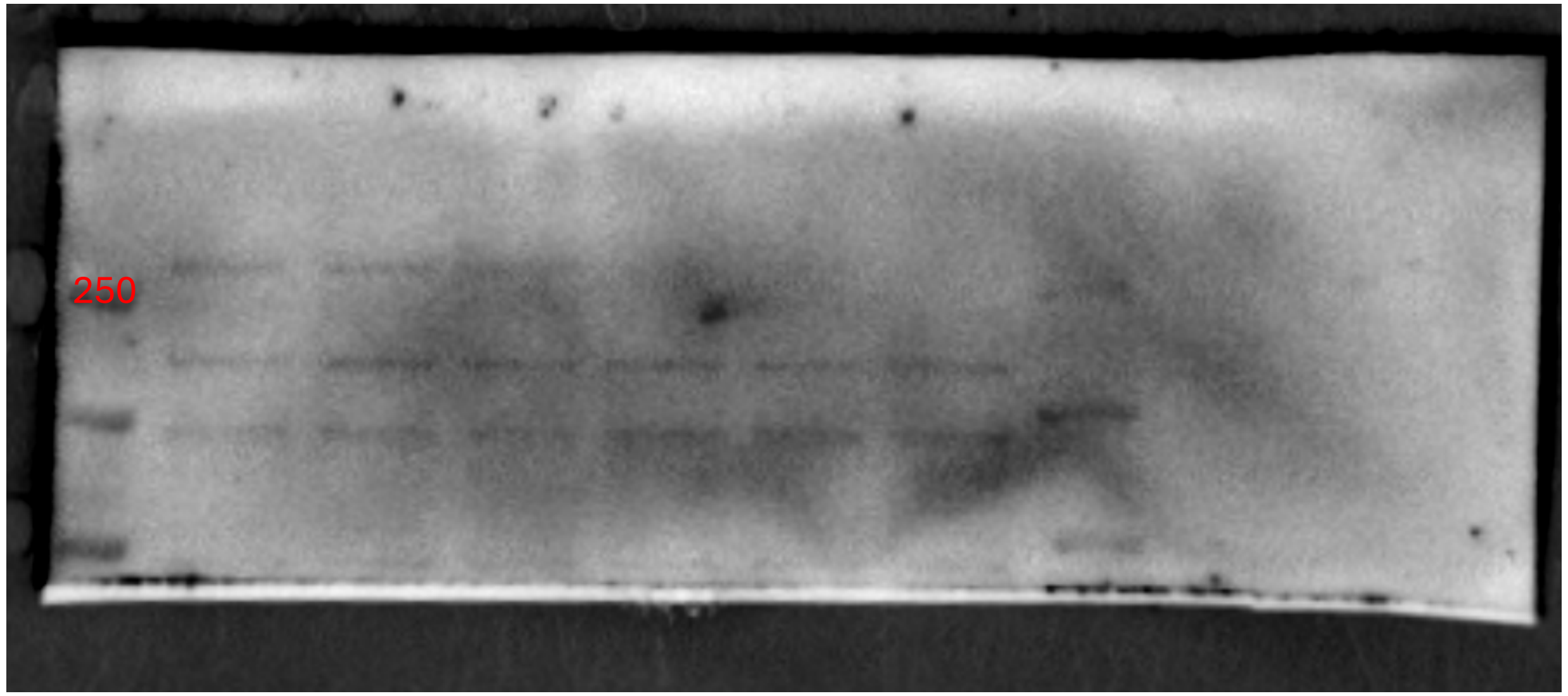

B-tubulin 7.2 sec exposure – accumulation mode

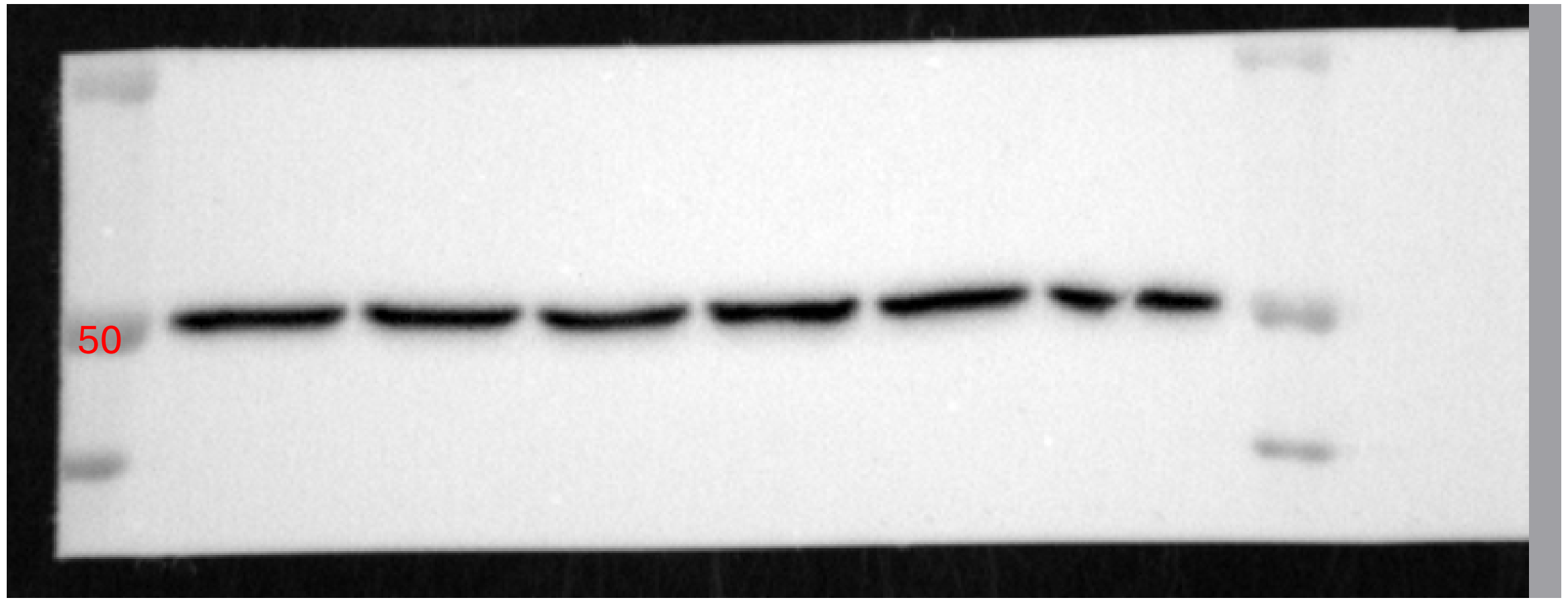

Lane	Sample
1	Ladder
2	Ctrl guide 1
3	Ctrl guide 1
4	Ctrl guide 2
5	ZFC3H1 guide 1
6	ZFC3H1 guide 1
7	ZFC3H1 guide 2
8	ladder

Supplementary Figure 8A

ZFC3H1 60 sec exposure – accumulation mode (max substrate)

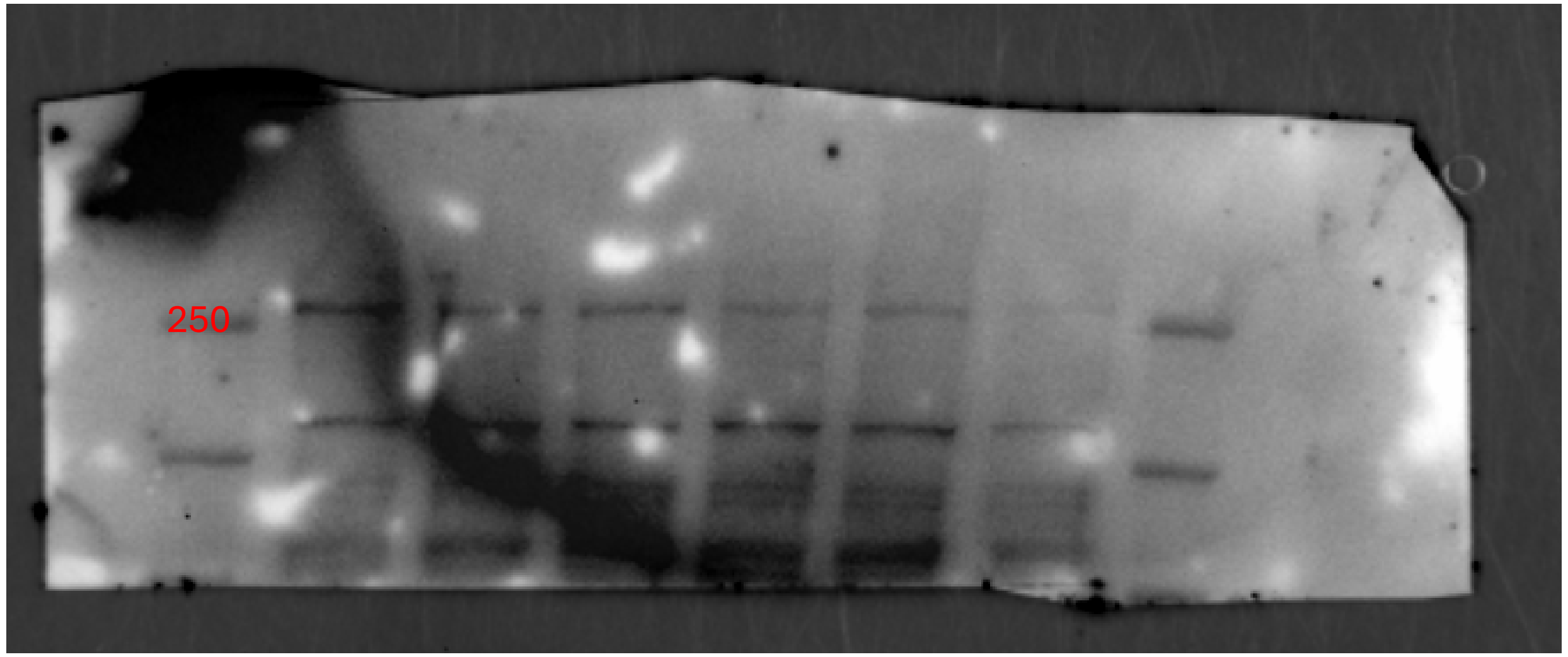

B-tubulin 4.1 sec exposure – accumulation mode

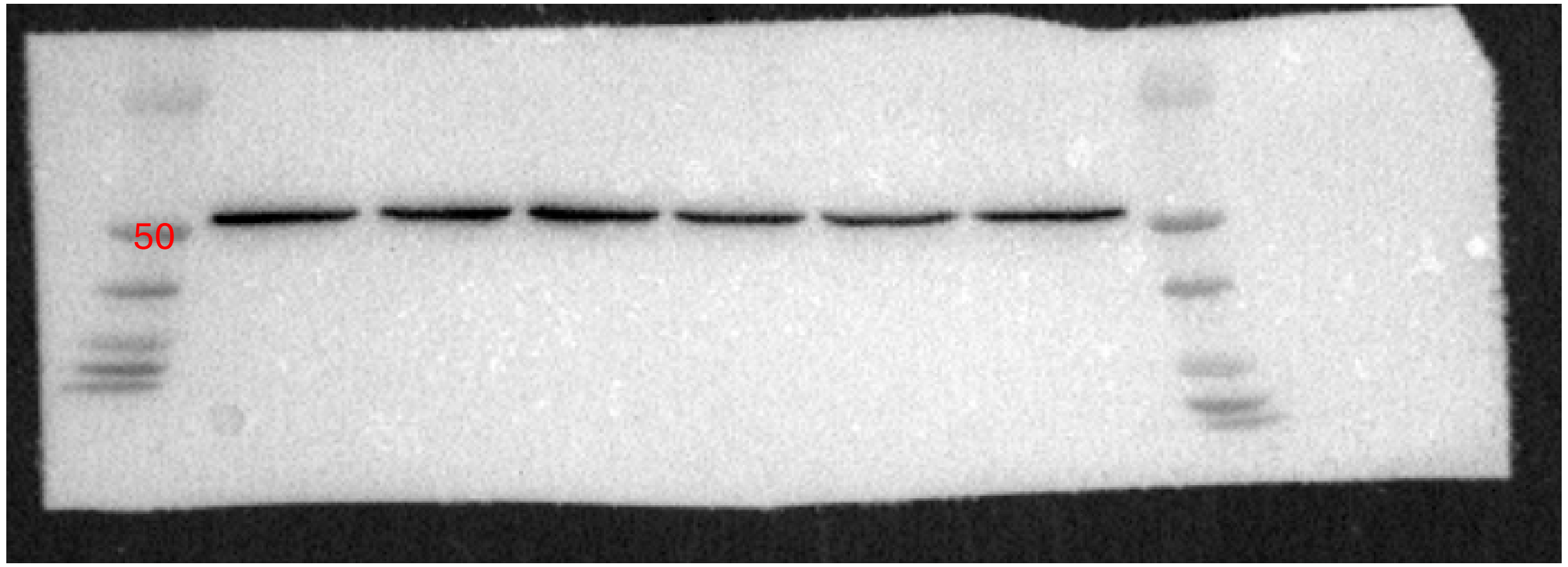

Lane	Sample
1	Ladder
2	Ctrl guide 1
3	Ctrl guide 1
4	Ctrl guide 2
5	ZFC3H1 guide 1
6	ZFC3H1 guide 1
7	ZFC3H1 guide 2
8	ladder

Supplementary Figure 9A

ZFC3H1 (41.4 sec exposure – accumulation mode, max substrate)

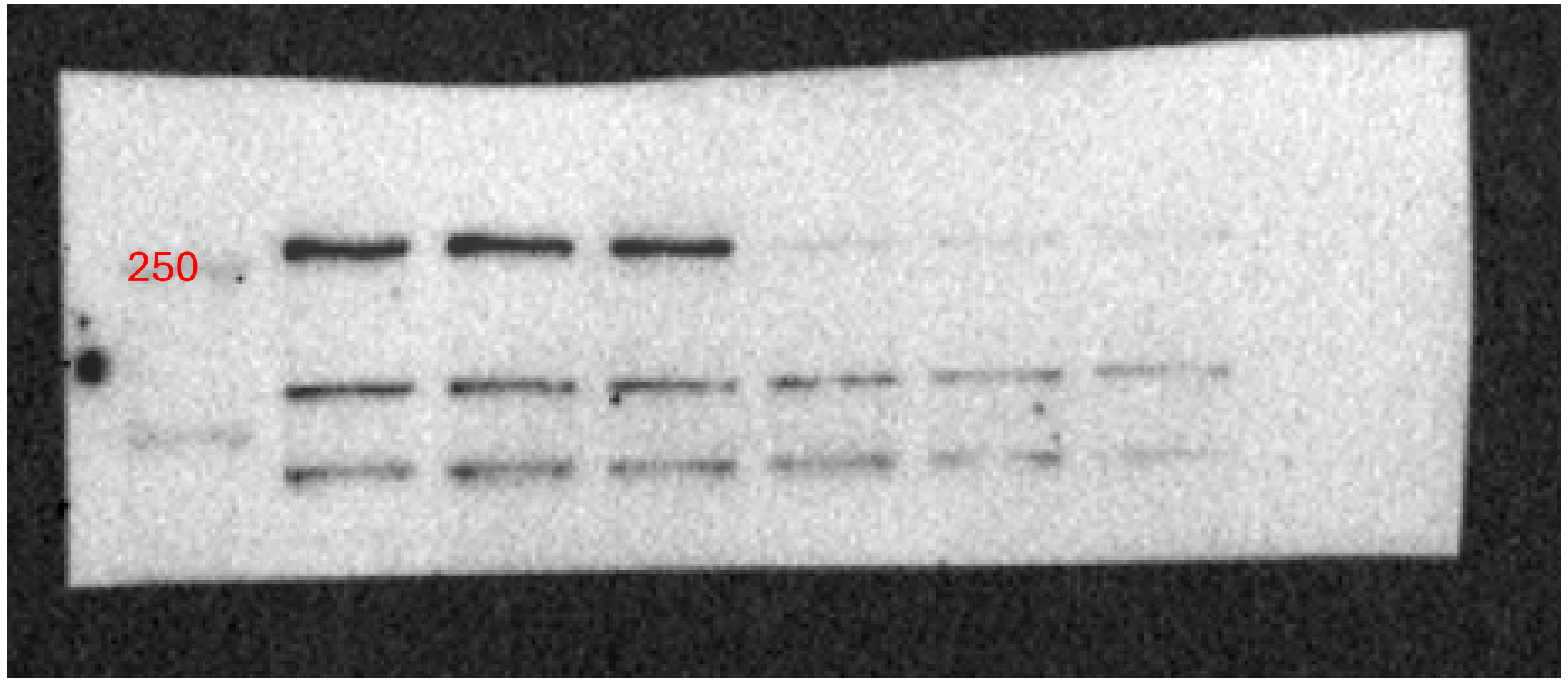

Lane	Sample
1	Ladder
2	Ctrl guide 1
3	Ctrl guide 1
4	Ctrl guide 2
5	ZFC3H1 guide 1
6	ZFC3H1 guide 1
7	ZFC3H1 guide 2

B-tubulin for ZFC3H1 (13.4 sec exposure – accumulation mode)

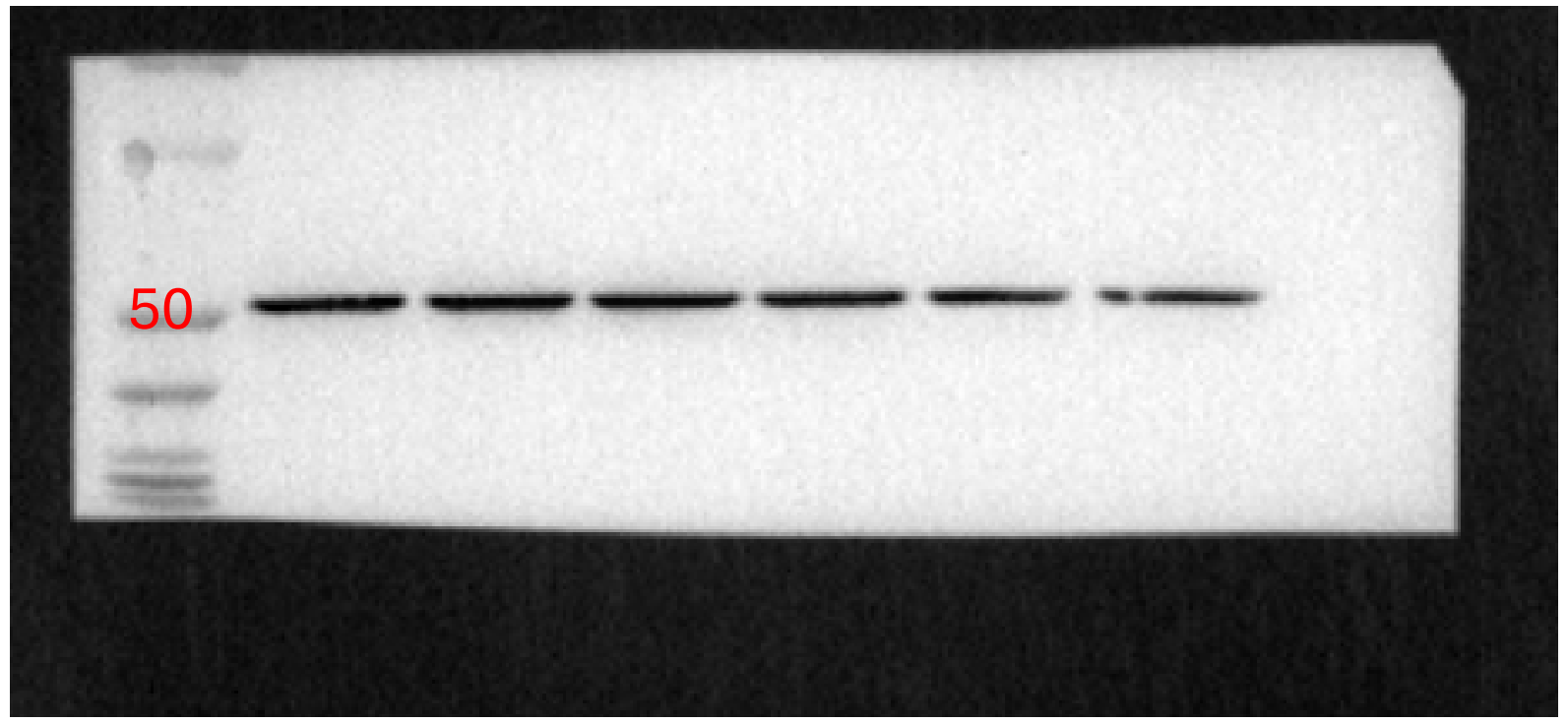

Supplementary Figure 9B – Ctrl sgRNA

1 sec exposure – max substrate

LaminB1

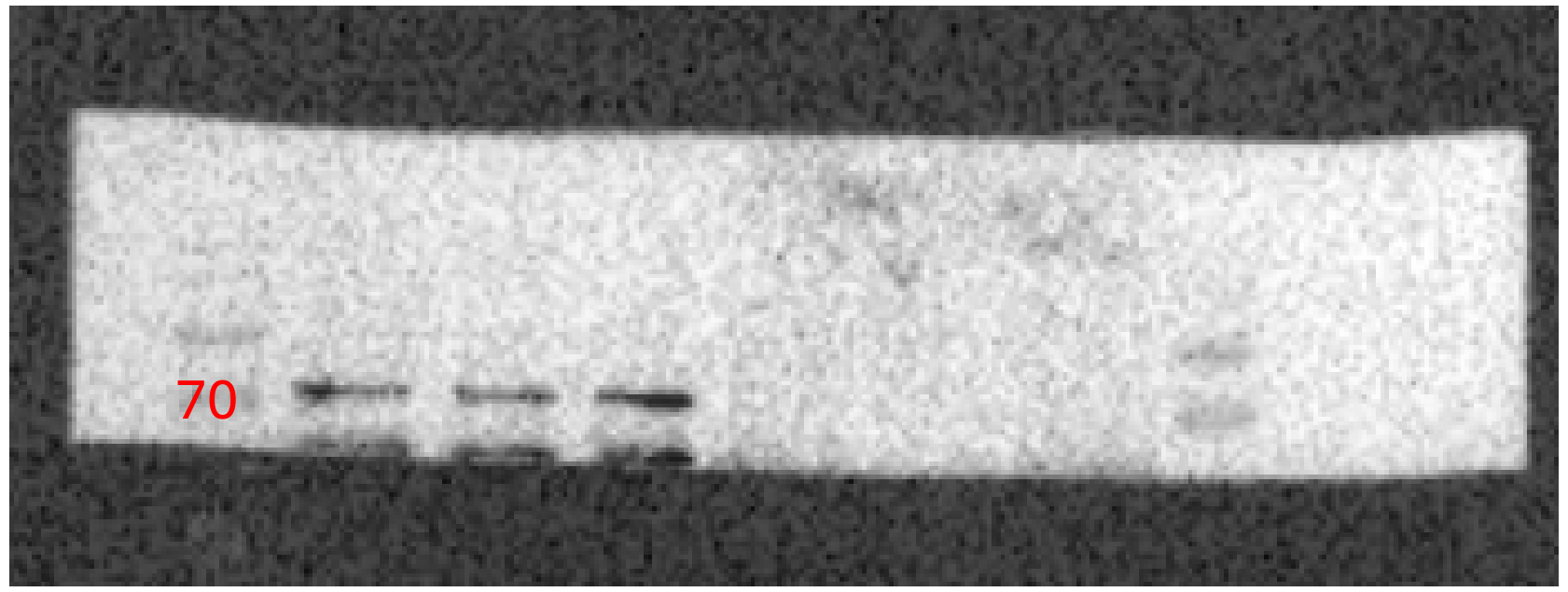

10.3 sec exposure – accumulation mode

B-Tubulin

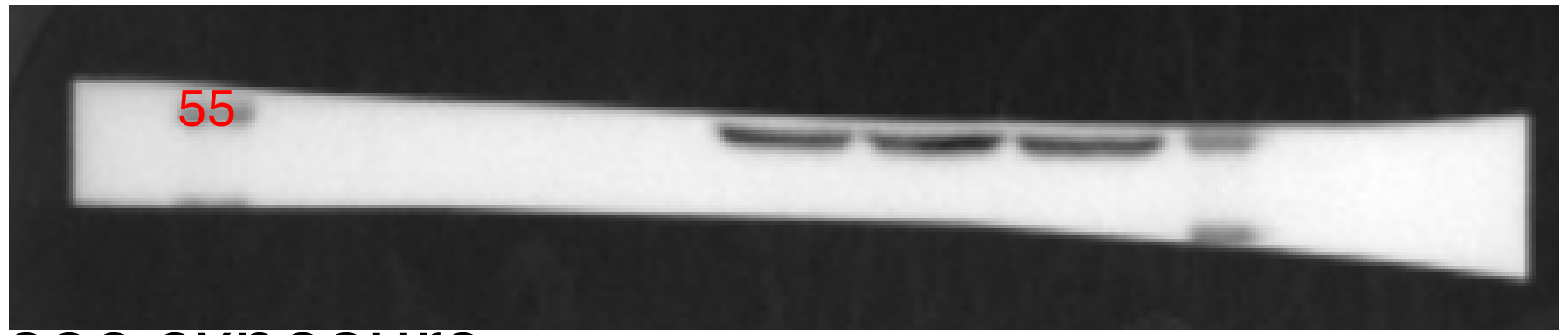

0.5 sec exposure

SSR1/TRAPA

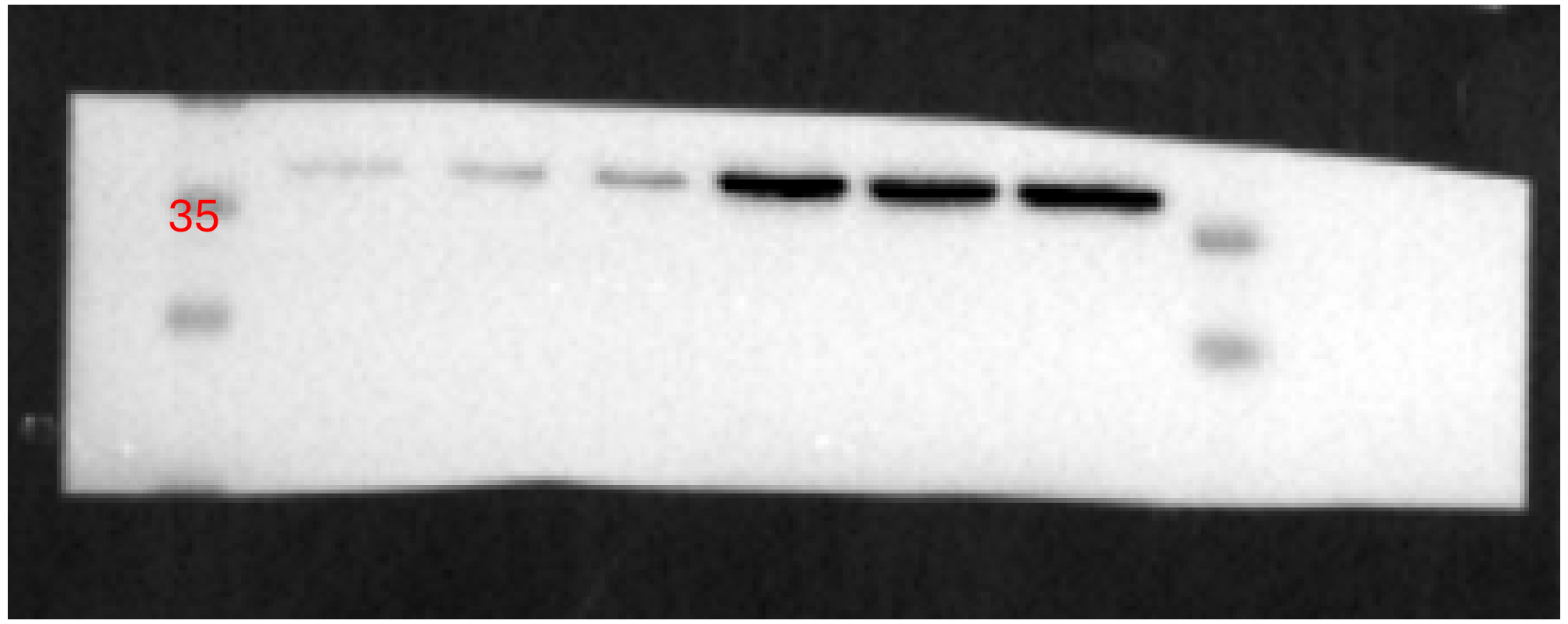

Lane	Sample
1	Ladder
2	Nuc rep 1
3	Nuc rep 2
4	Nuc rep 3
5	EN rep 1
6	EN rep 2
7	EN rep 3
8	ladder

Supplementary Figure 9B – ZFC3H1 sgRNA

1 sec exposure – max substrate

LaminB1

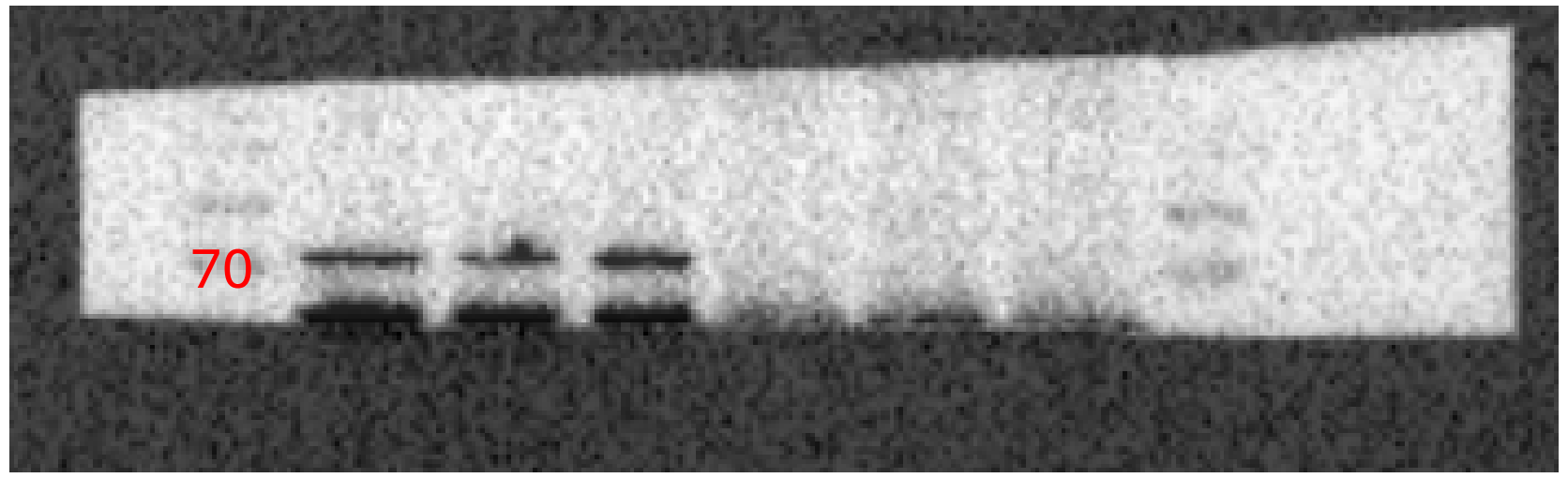

10.3 sec exposure – accumulation mode

B-Tubulin

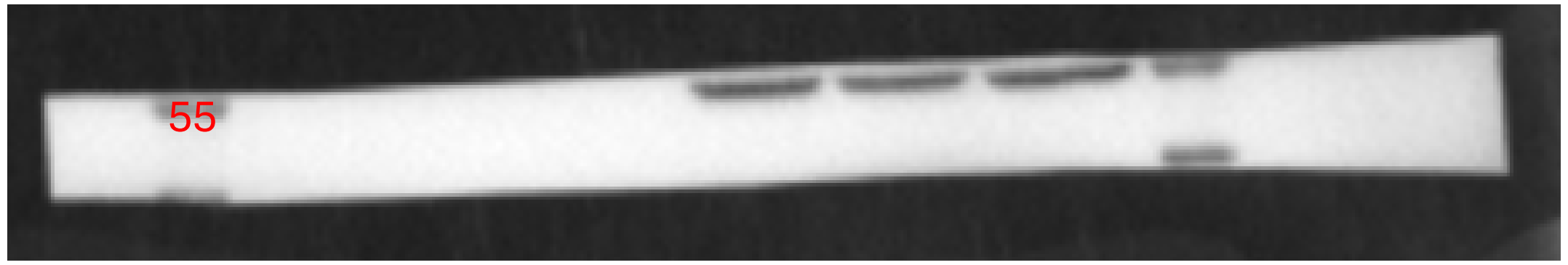

.5 sec exposure

SSR1/TRAPA

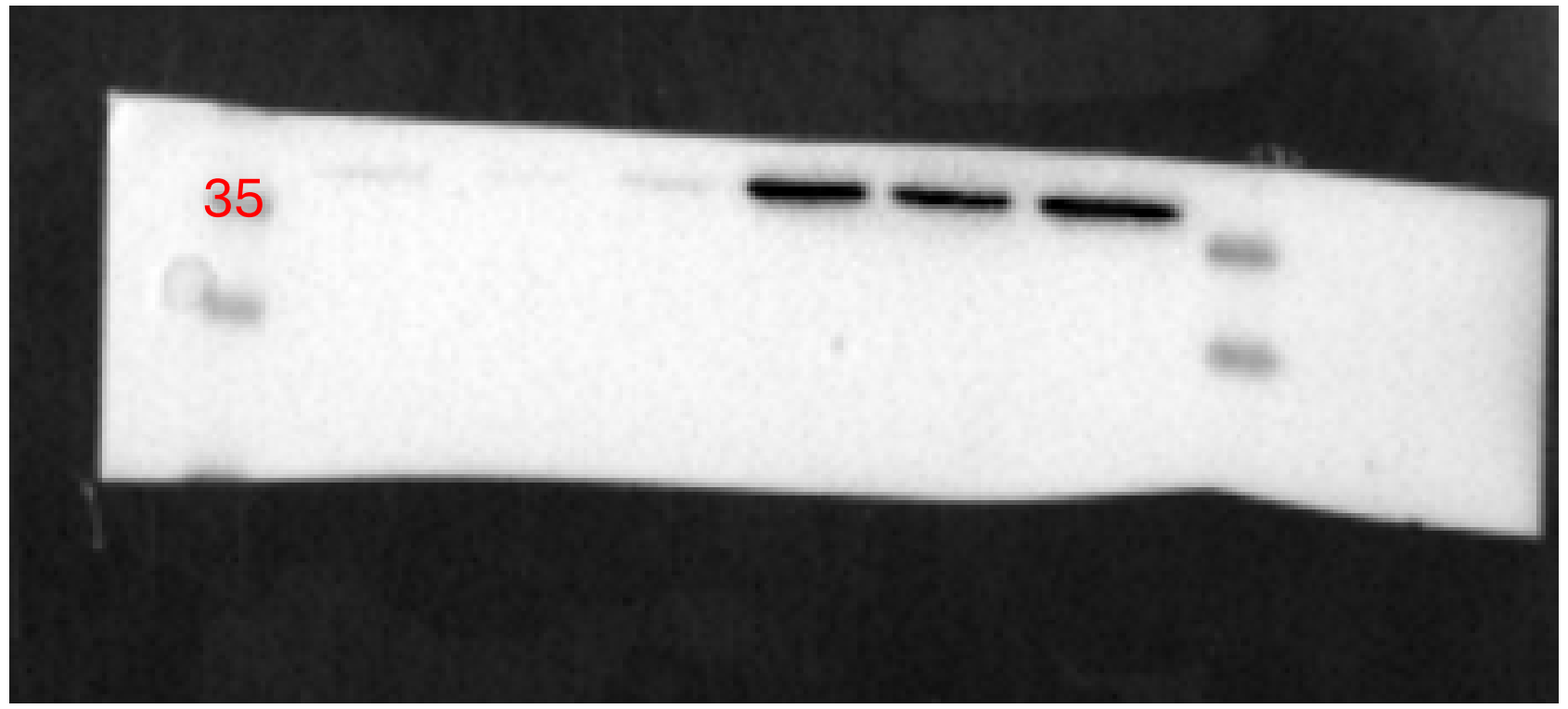

Lane	Sample
1	Ladder
2	Nuc rep 1
3	Nuc rep 2
4	Nuc rep 3
5	EN rep 1
6	EN rep 2
7	EN rep 3
8	ladder

Supplementary Figure 9C

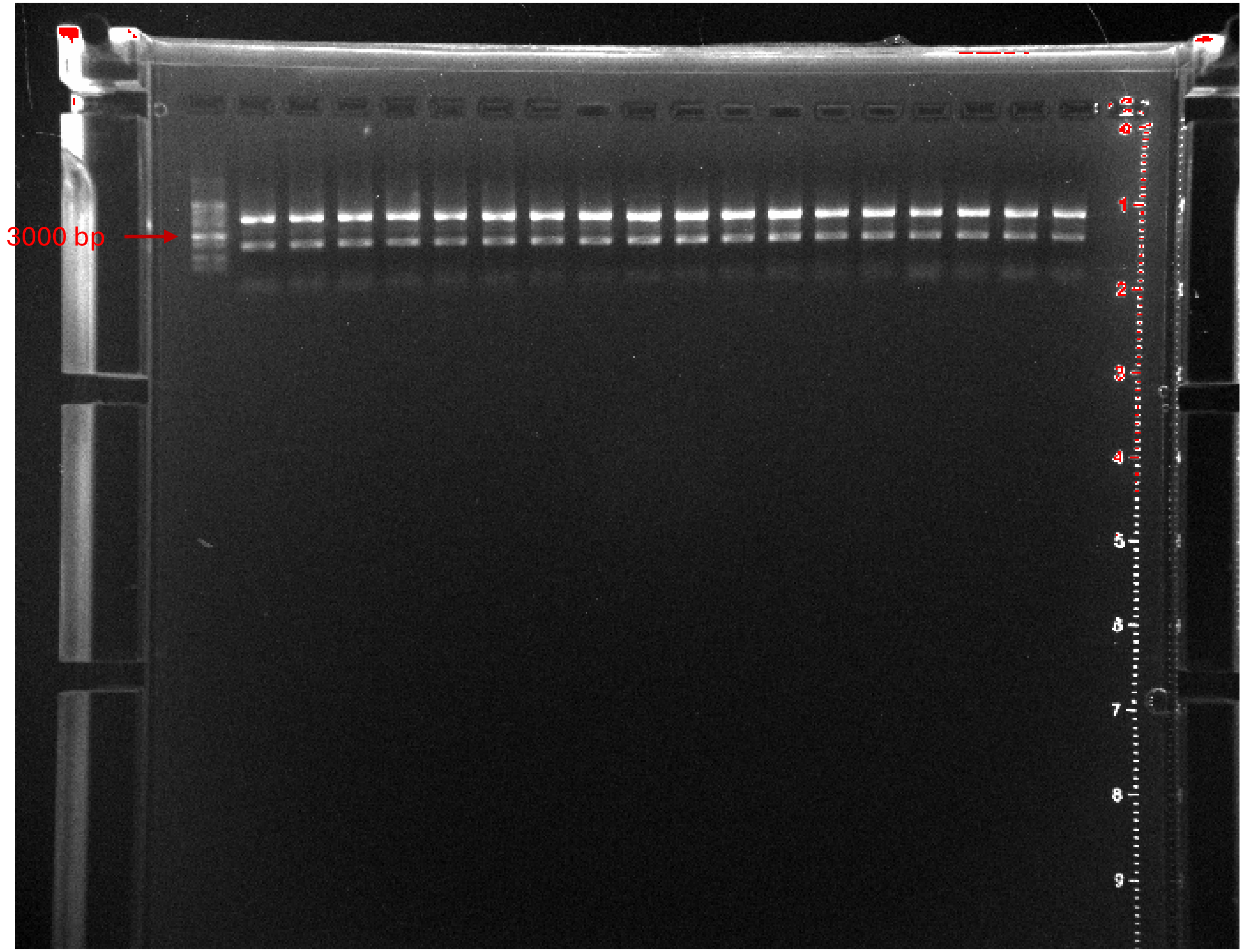

Lane	Sample
1	NEB ssRNA ladder
2-7	Whole cell samples (ZFC3H1 guide replicates and then Ctrl guide replicates)
8-13	Nuclear samples (ZFC3H1 guide replicates and then Ctrl guide replicates)
14-19	Extranuclear samples (ZFC3H1 guide replicates and then Ctrl guide replicates)

Supplementary Figure 11A –rep 1

ZFC3H1 (7.2 sec exposure – accumulation mode, max substrate)

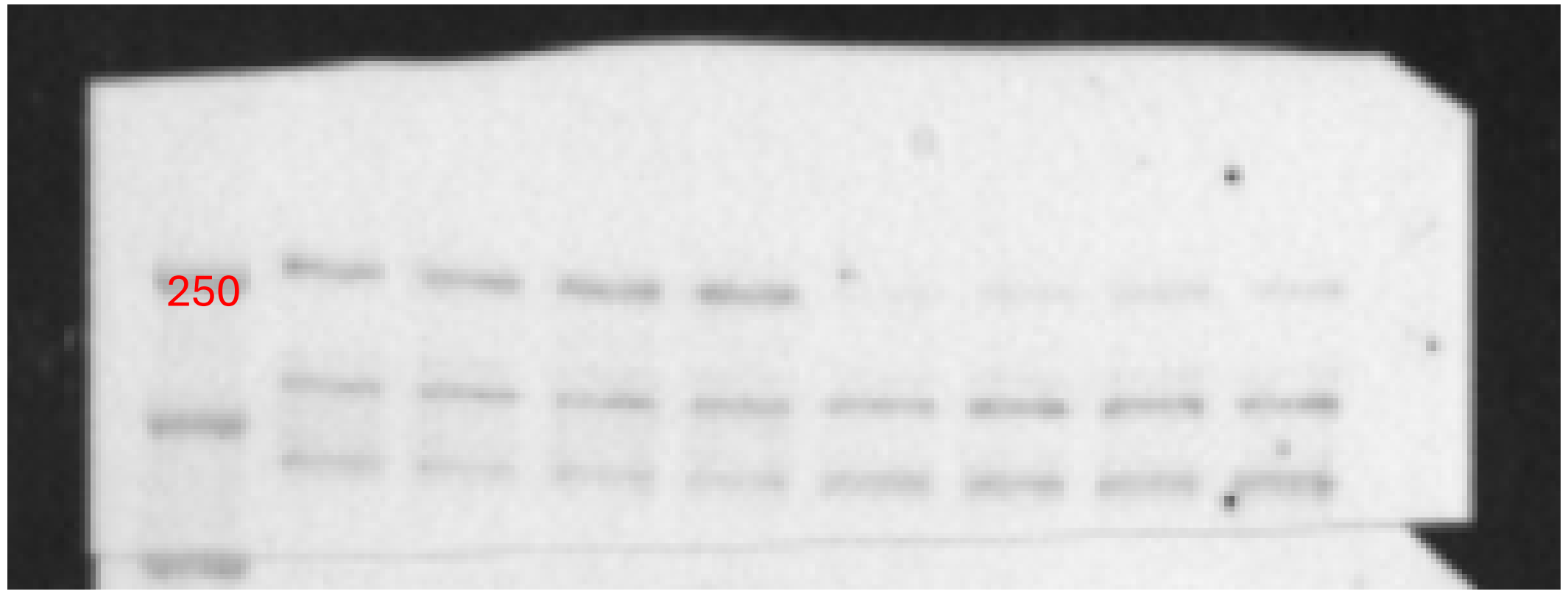

B-tubulin (0.5 sec exposure – accumulation mode)

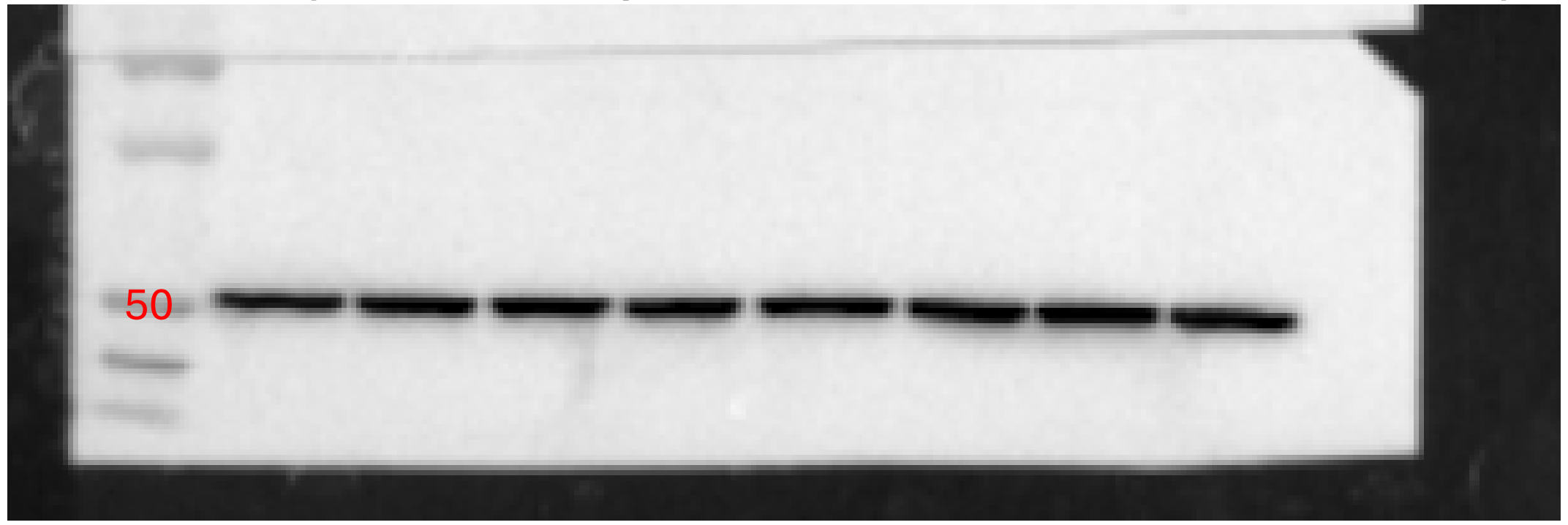

Lane	Sample
1	Ladder
2	Ctrl guide 1, Low reporter
3	Ctrl guide 1, Medium Reporter
4	Ctrl guide 2, Low Reporter
5	Ctrl guide 2, Medium Reporter
6	ZFC3H1 guide 1, Low reporter
7	ZFC3H1 guide 1, Medium Reporter
8	ZFC3H1 guide 2, Low reporter
9	ZFC3H1 guide 2, Medium Reporter

Supplementary Figure 11A – rep 1

B-Tubulin for GFP (10.3 sec accumulation mode)

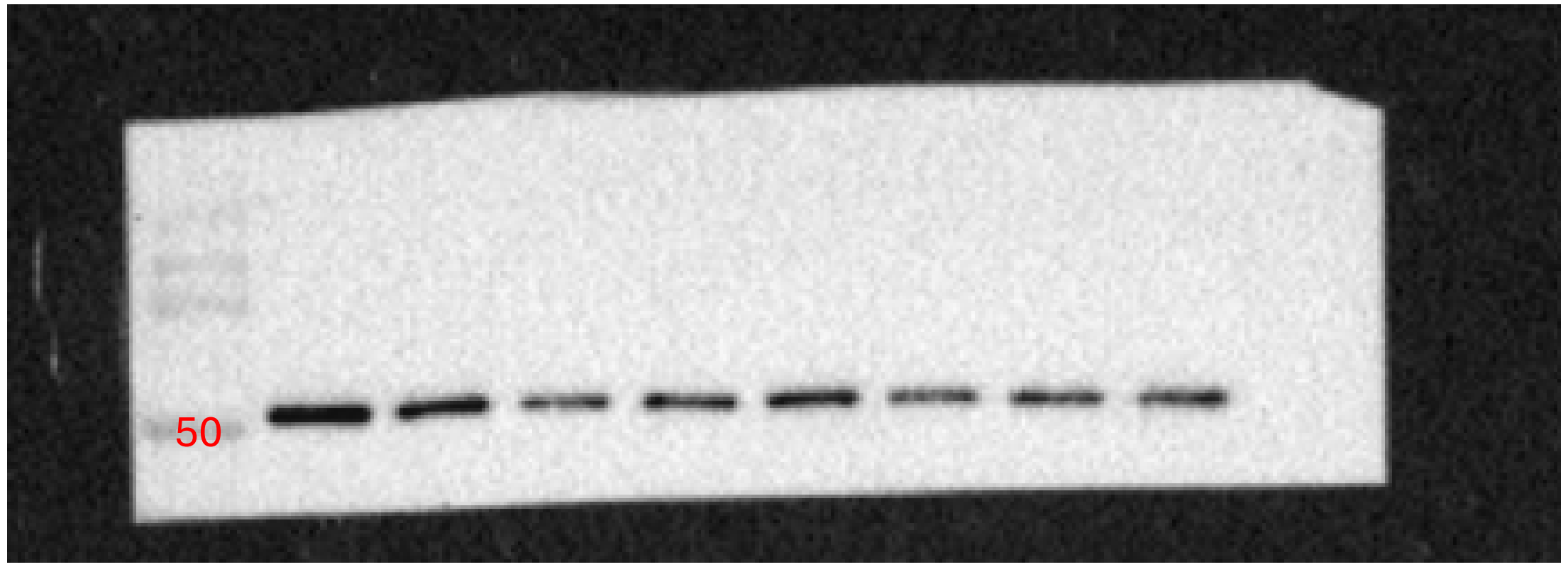

GFP (13.4 sec accumulation mode, max substrate)

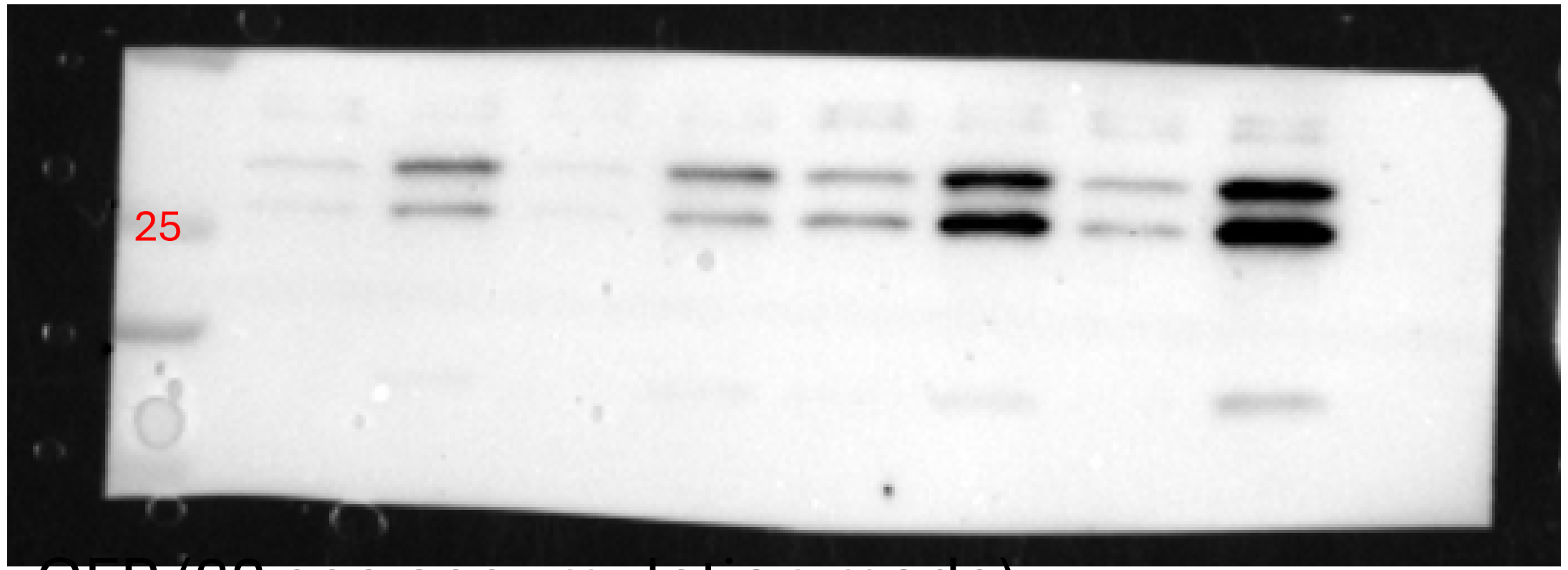

GFP (32 sec accumulation mode)

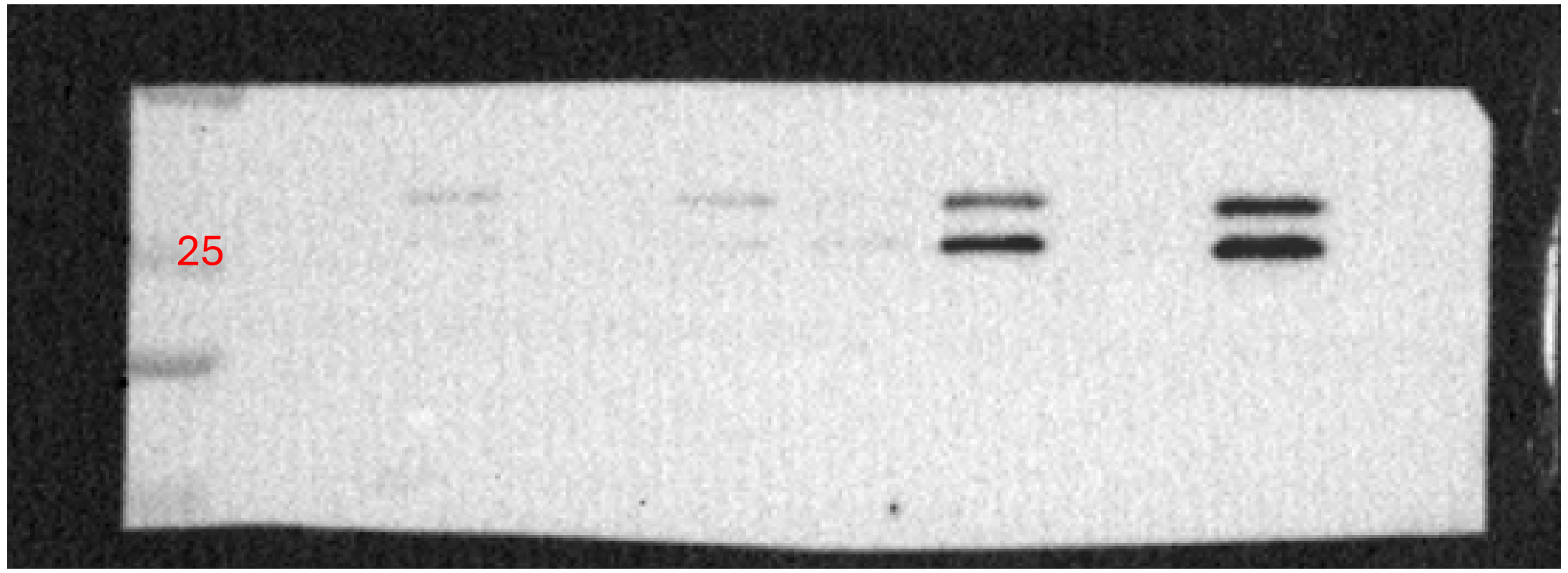

B-Tubulin for mCherry (10.3 sec accumulation mode)

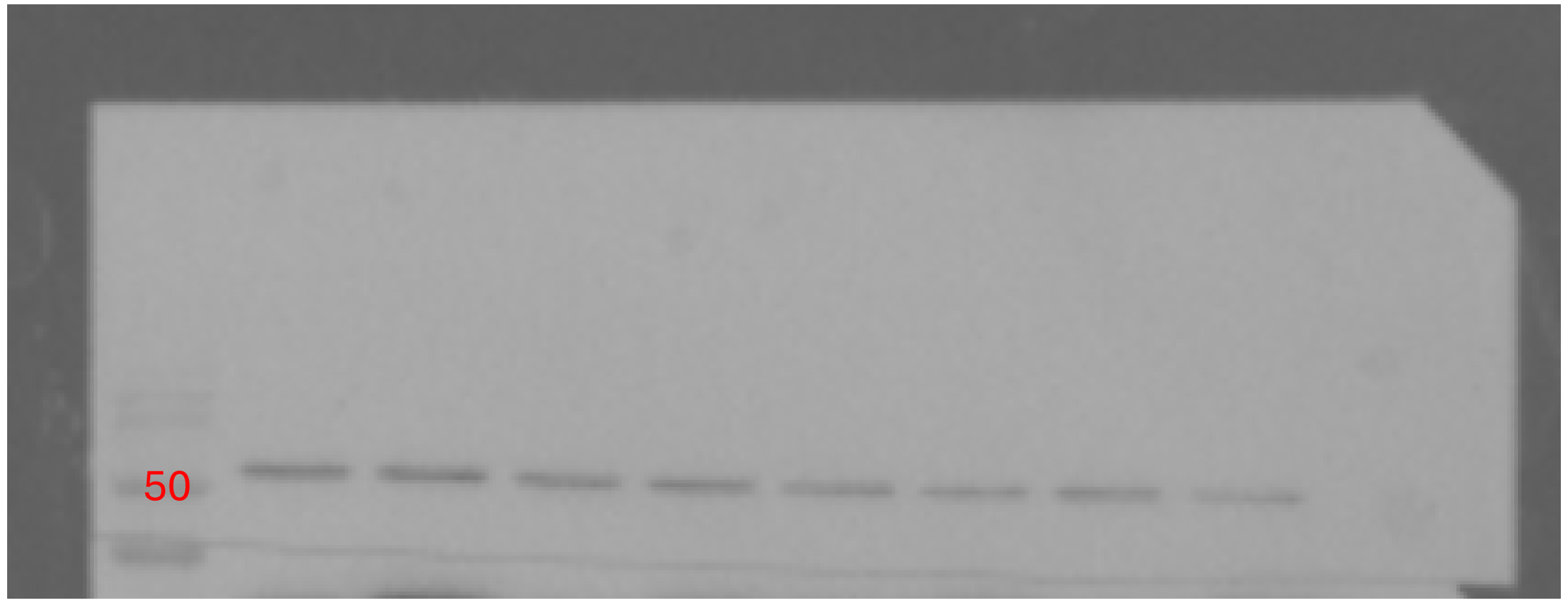

mCherry (.25 sec)

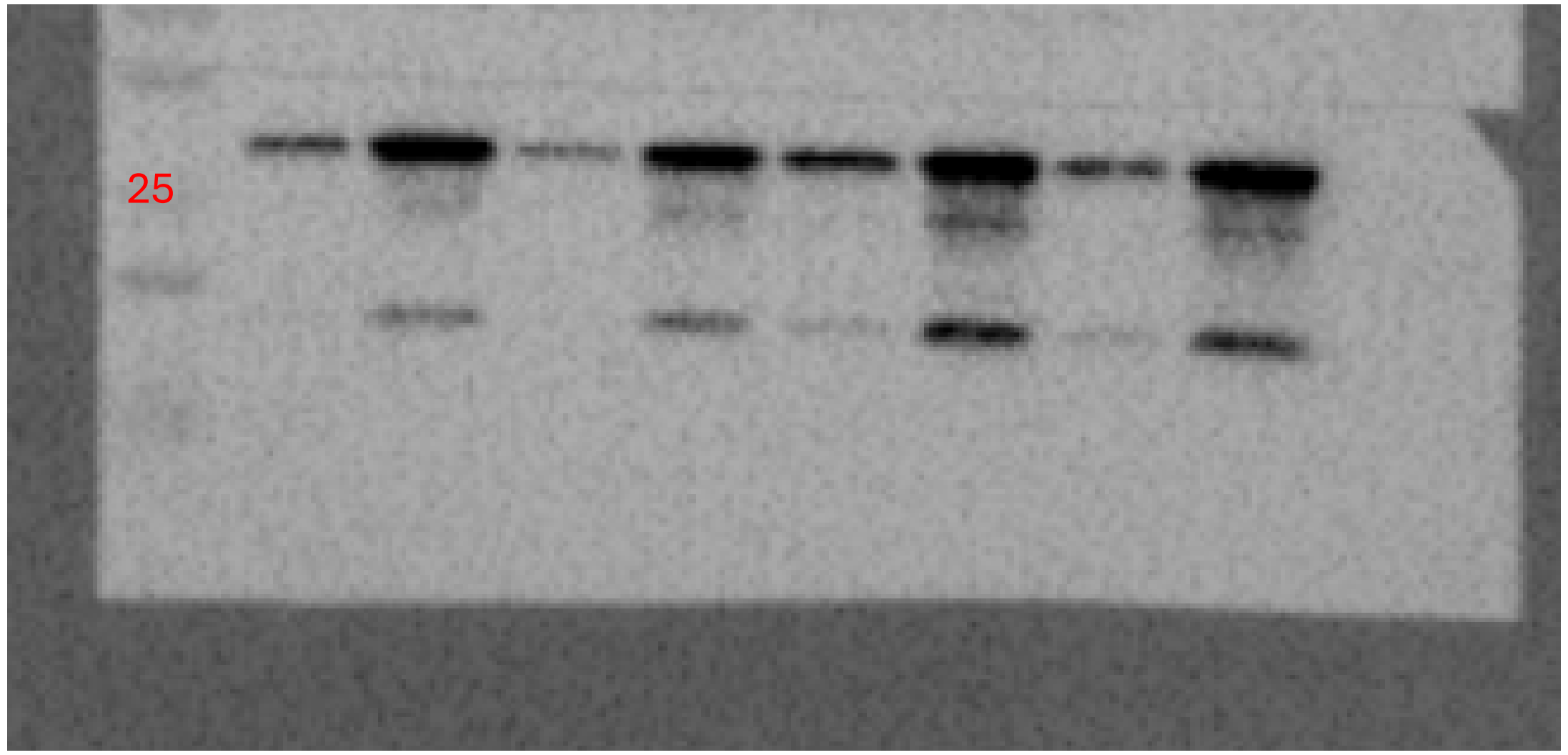

Supplementary Figure 11A –rep 2

ZFC3H1 (22.7 sec exposure – accumulation mode, max substrate)

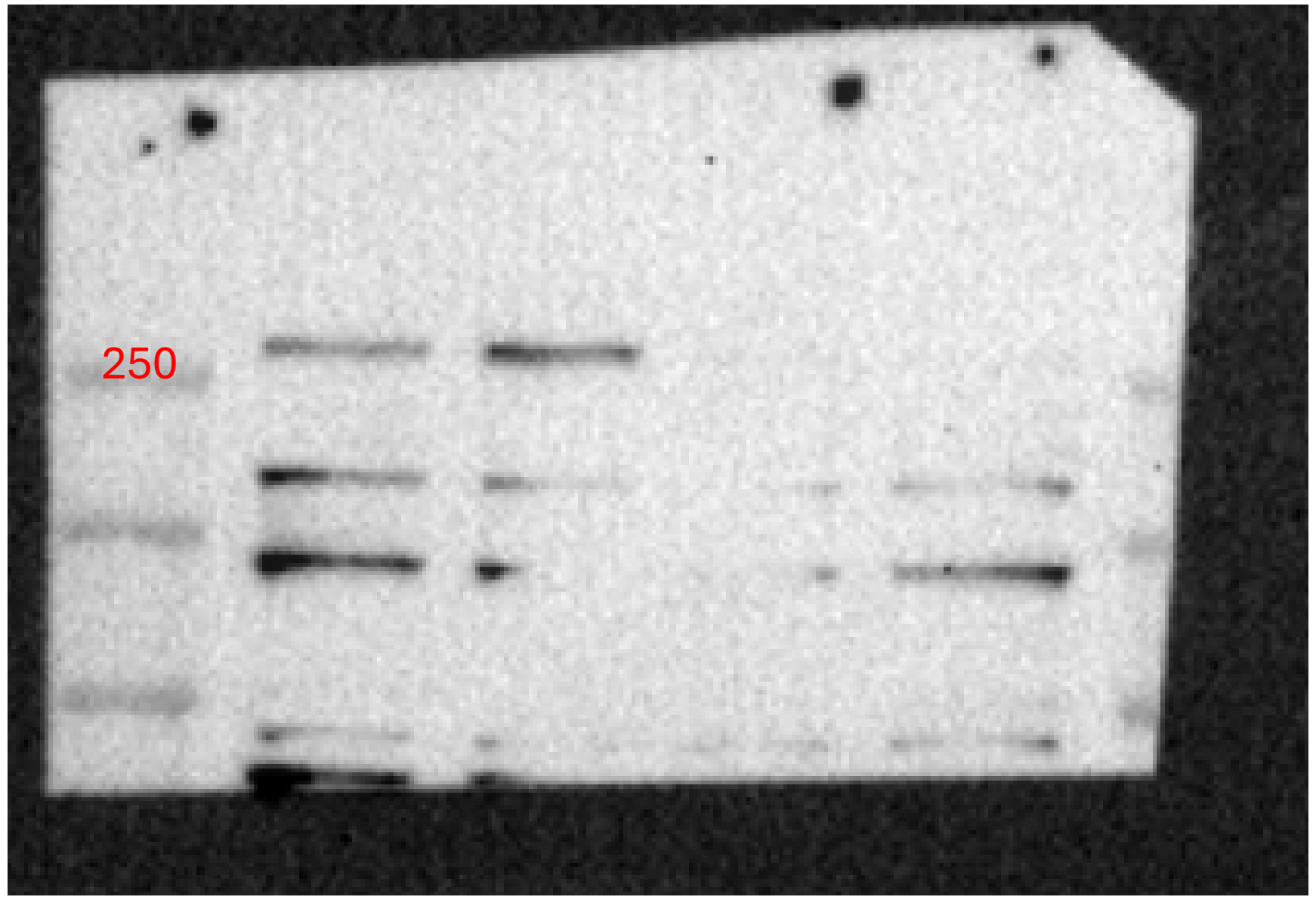

B-tubulin (10.3 sec exposure – accumulation mode)

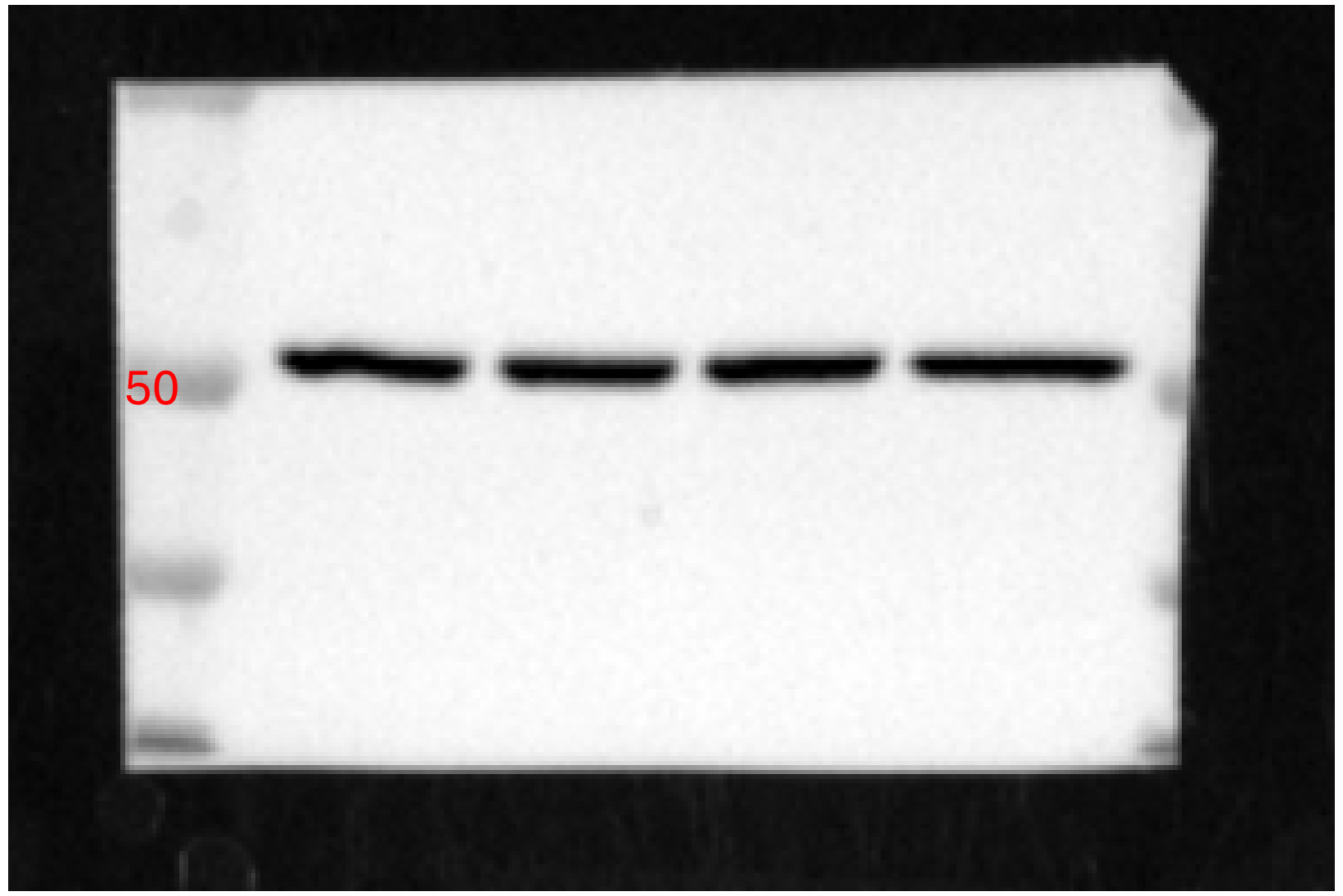

Lane	Sample
1	Ladder
2	Ctrl guide 1, Low reporter
3	Ctrl guide 1, Medium Reporter
6	ZFC3H1 guide 1, Low reporter
7	ZFC3H1 guide 1, Medium Reporter

Supplementary Figure 11A –rep 2

B-Tubulin for GFP (10.3 sec accumulation mode)

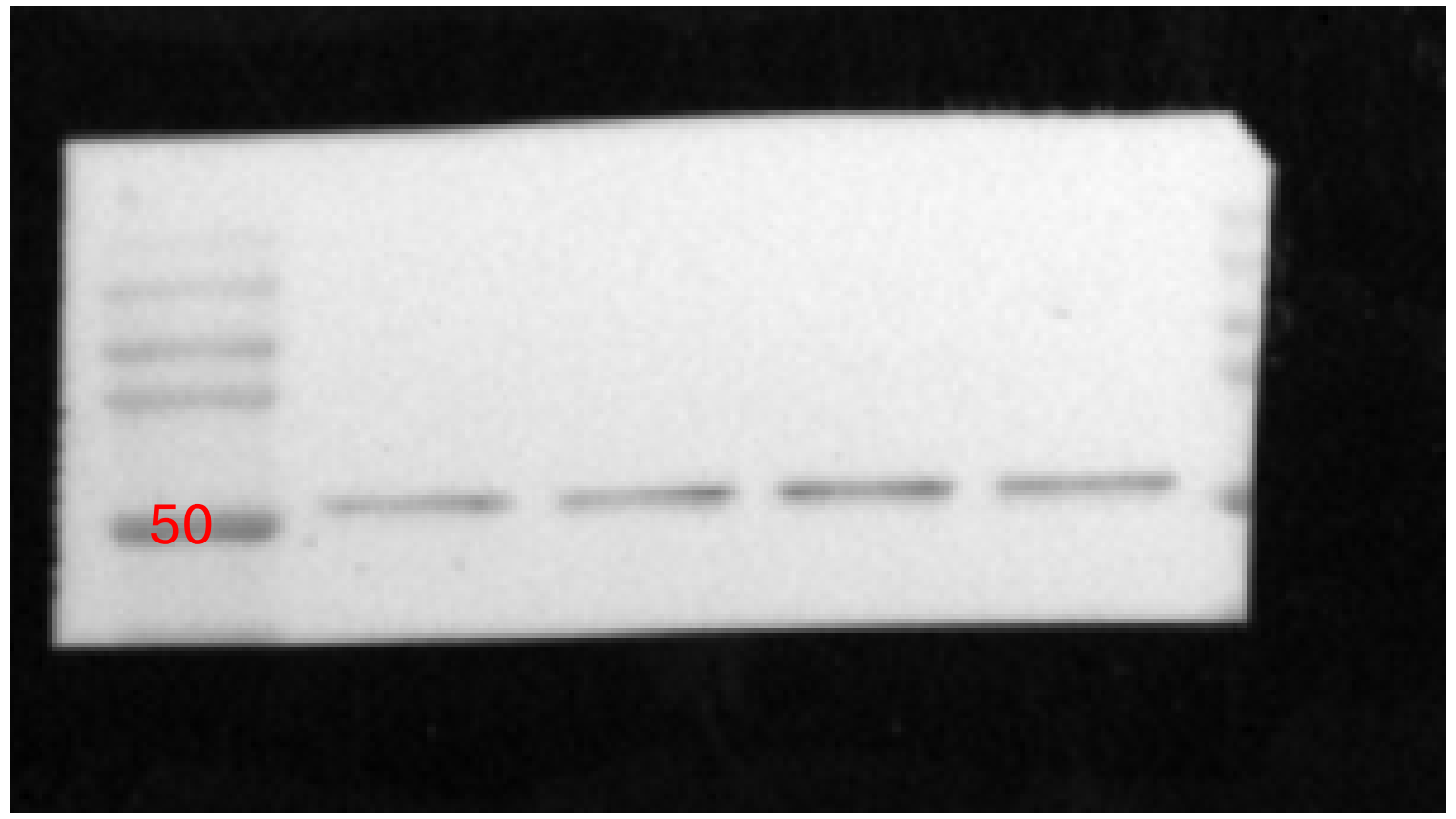

GFP (4.1 sec accumulation mode, max subtrate)

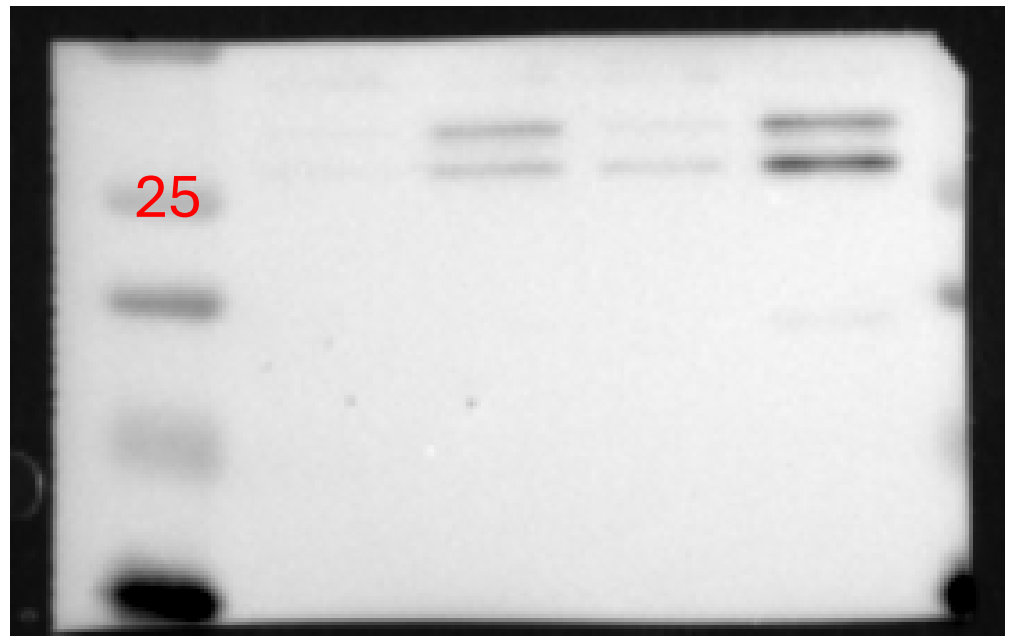

GFP (16.5 sec accumulation mode, max subtrate)

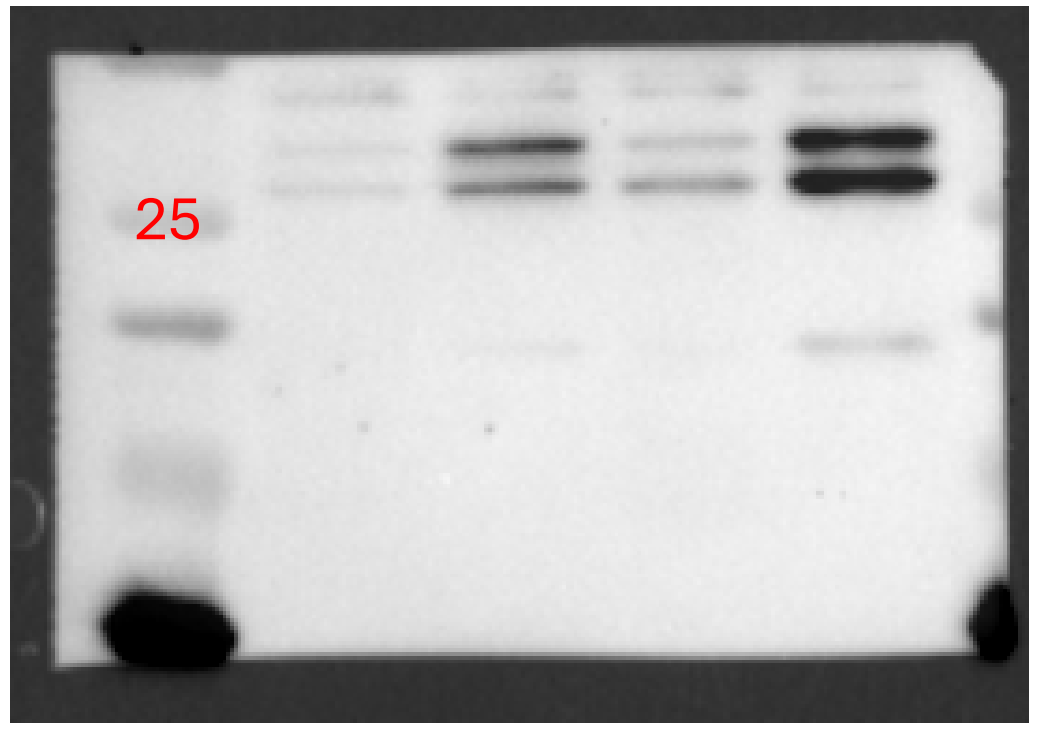

B-Tubulin for mCherry (10.3 sec accumulation mode)

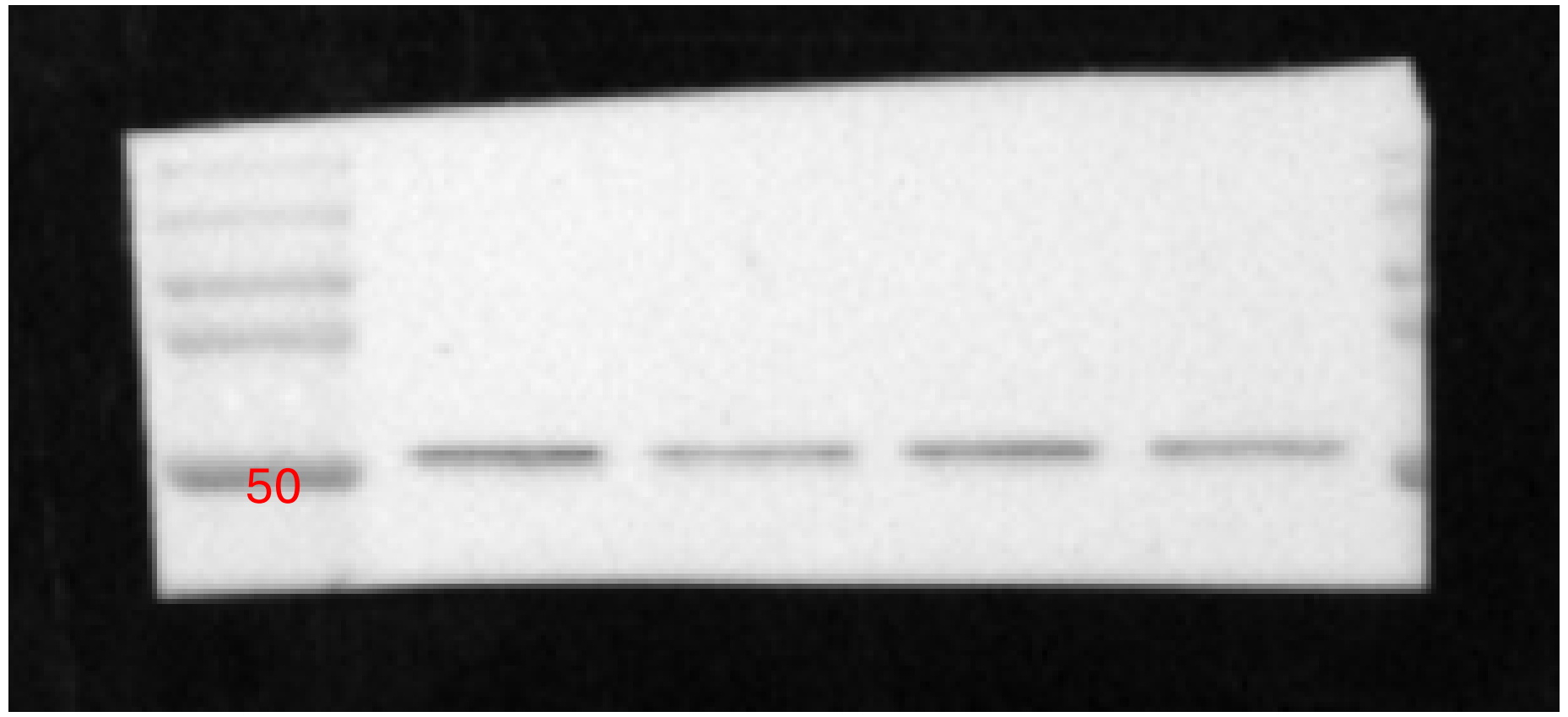

mCherry (1 sec accumulation mode)

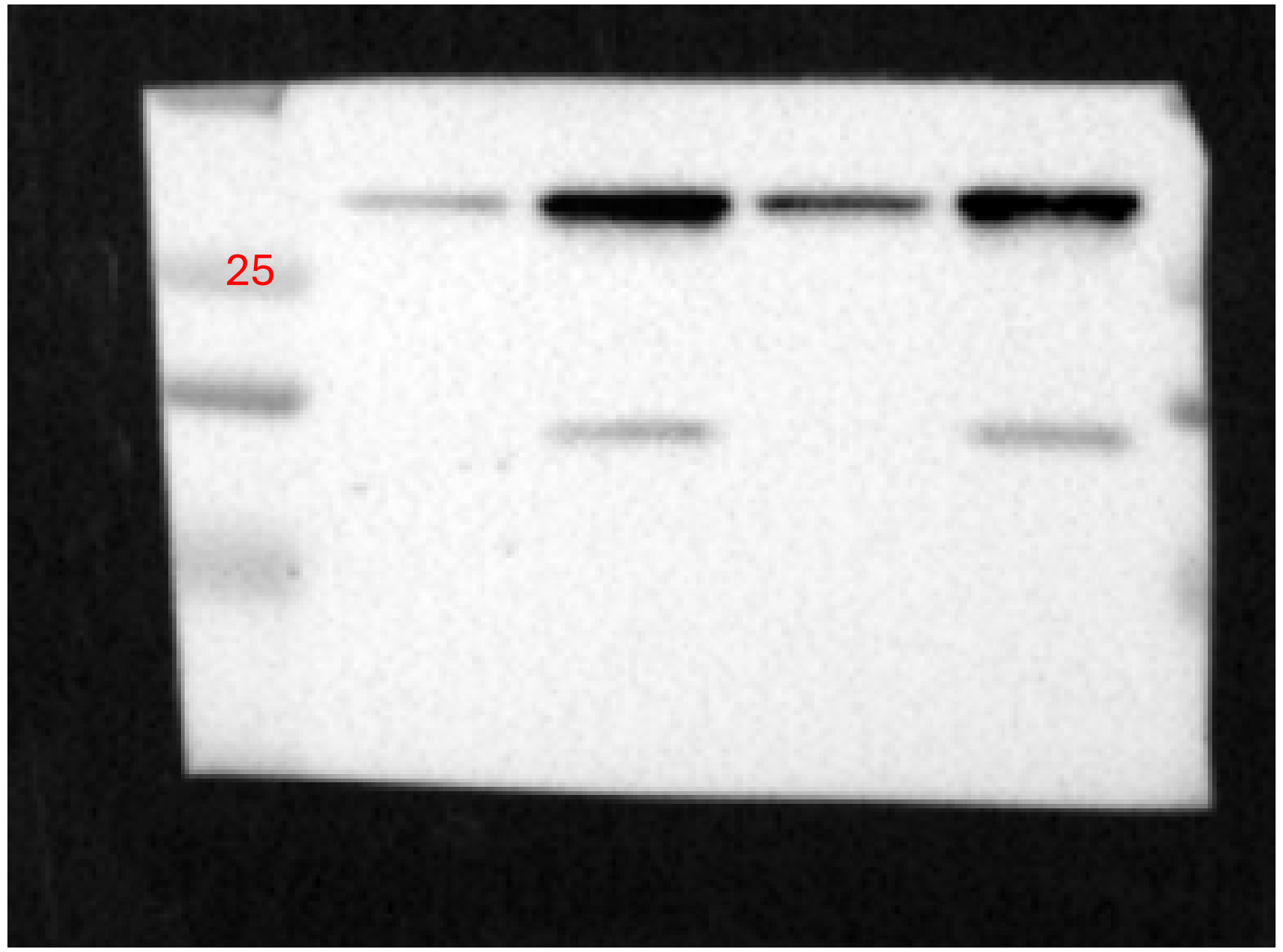

Supplementary Figure 11B

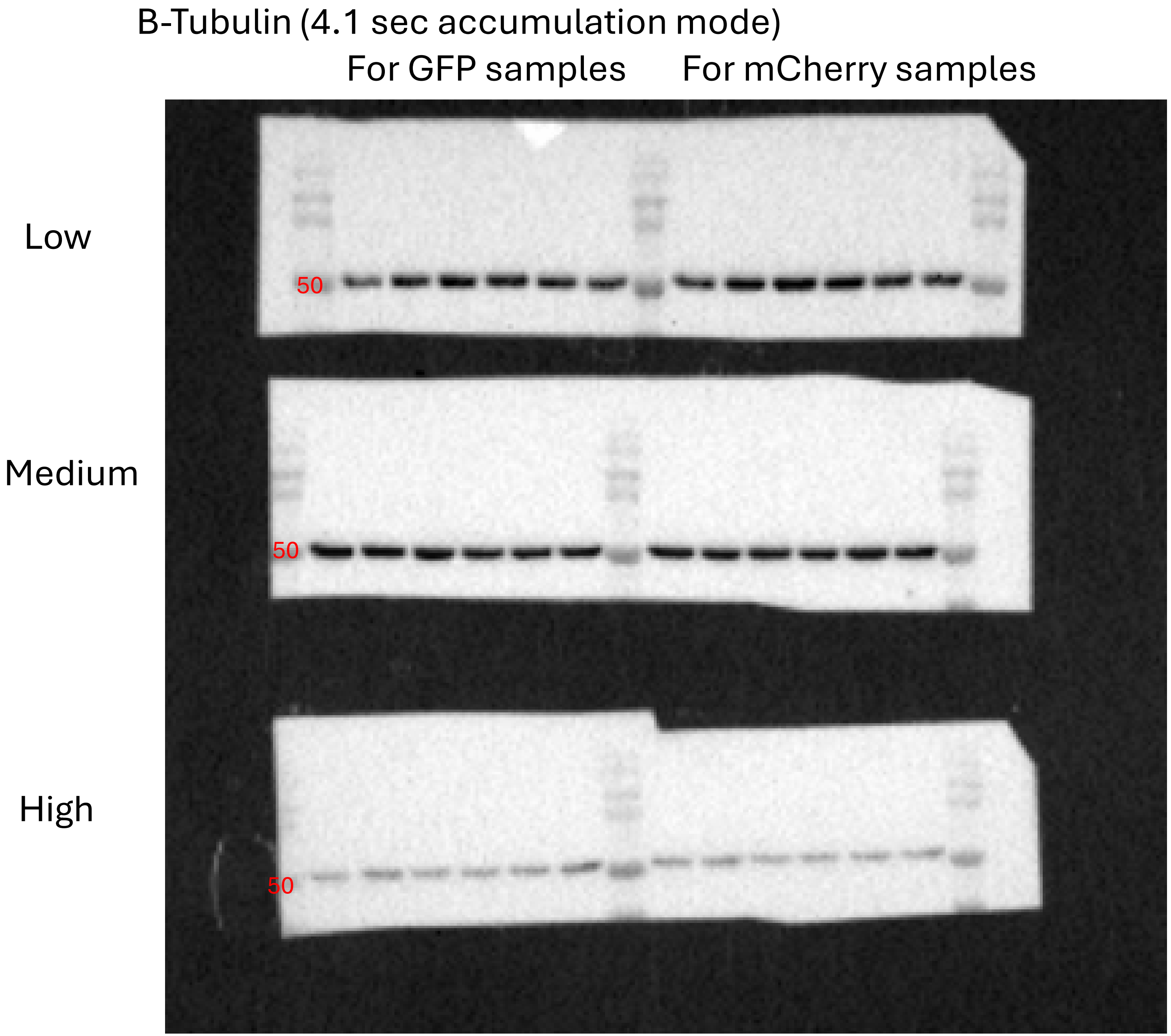

Lane	Sample
1	Ladder
2	Ctrl guide 1
3	Ctrl guide 1
4	Ctrl guide 2
5	ZFC3H1 guide 1
6	ZFC3H1 guide 1
7	ZFC3H1 guide 2
8	ladder
9	Ctrl guide 1
10	Ctrl guide 1
11	Ctrl guide 2
12	ZFC3H1 guide 1
13	ZFC3H1 guide 1
14	ZFC3H1 guide 2
15	ladder

Supplementary Figure 11B

mCherry (25.8 sec accumulation mode)

Low

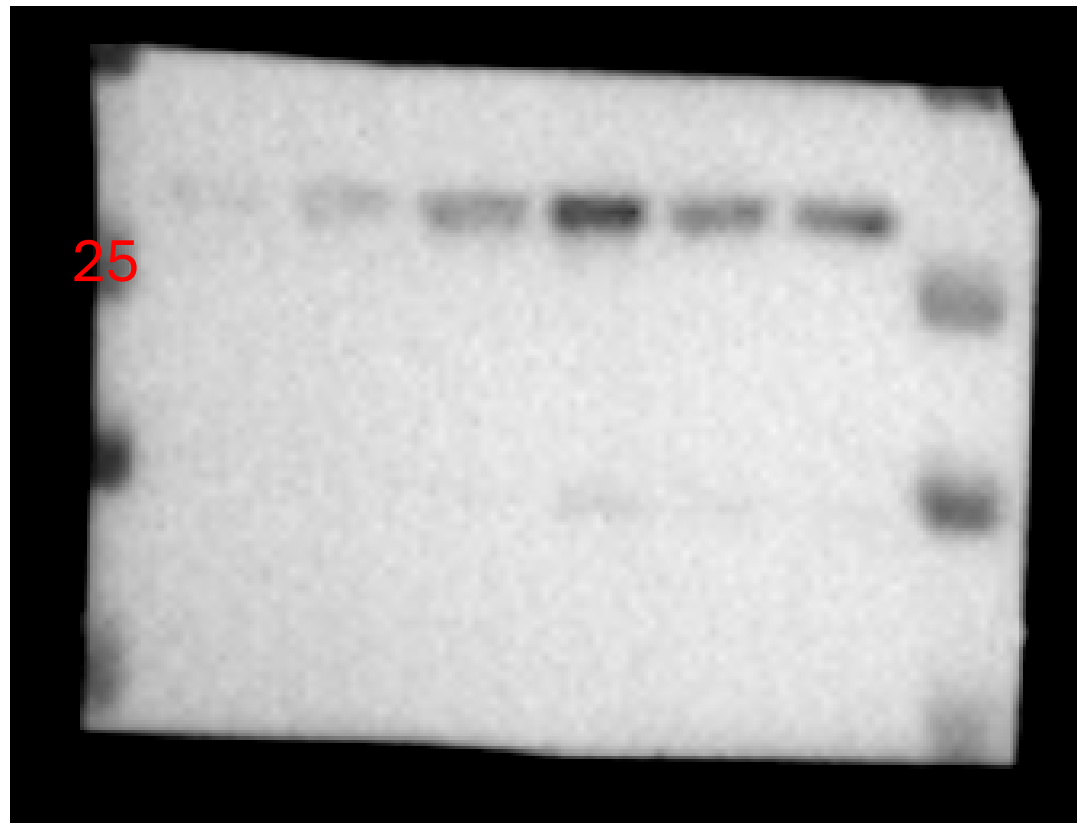

mCherry (.25 sec accumulation mode)

Medium High

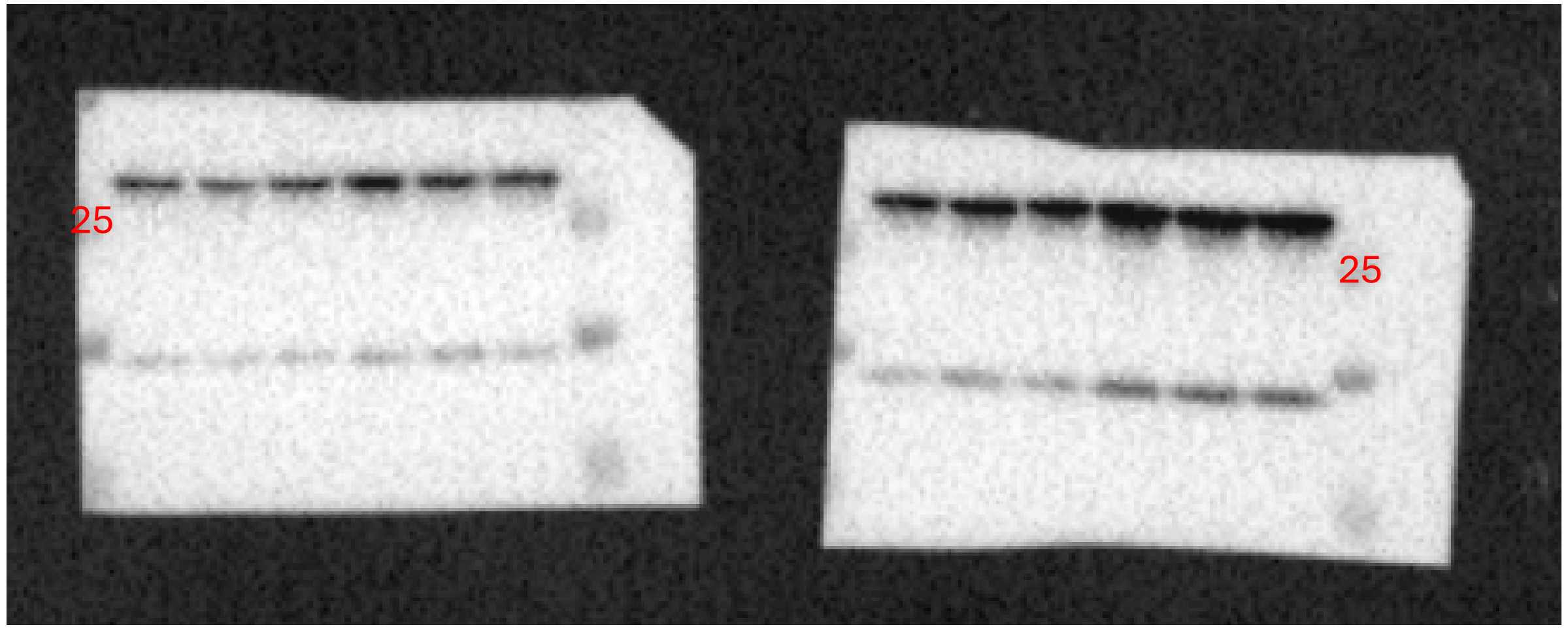

Lane	Sample
1	Ladder
2	Ctrl guide 1
3	Ctrl guide 1
4	Ctrl guide 2
5	ZFC3H1 guide 1
6	ZFC3H1 guide 1
7	ZFC3H1 guide 2
8	ladder

Supplementary Figure 11B

GFP (32 sec accumulation mode, max substrate)

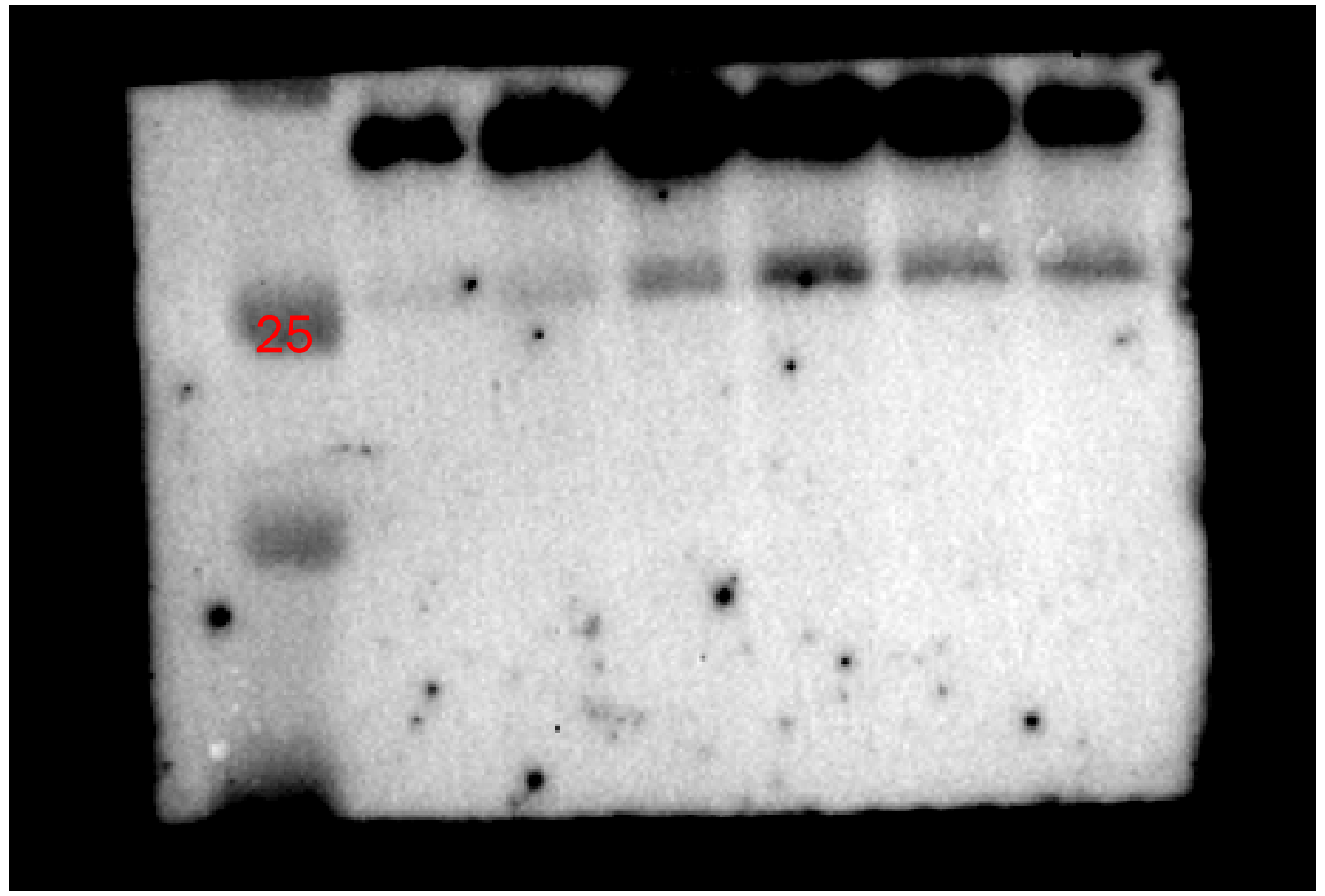

Low

Lane	Sample
1	Ladder
2	Ctrl guide 1
3	Ctrl guide 1
4	Ctrl guide 2
5	ZFC3H1 guide 1
6	ZFC3H1 guide 1
7	ZFC3H1 guide 2
8	ladder

GFP (7.2 sec accumulation mode, max substrate)

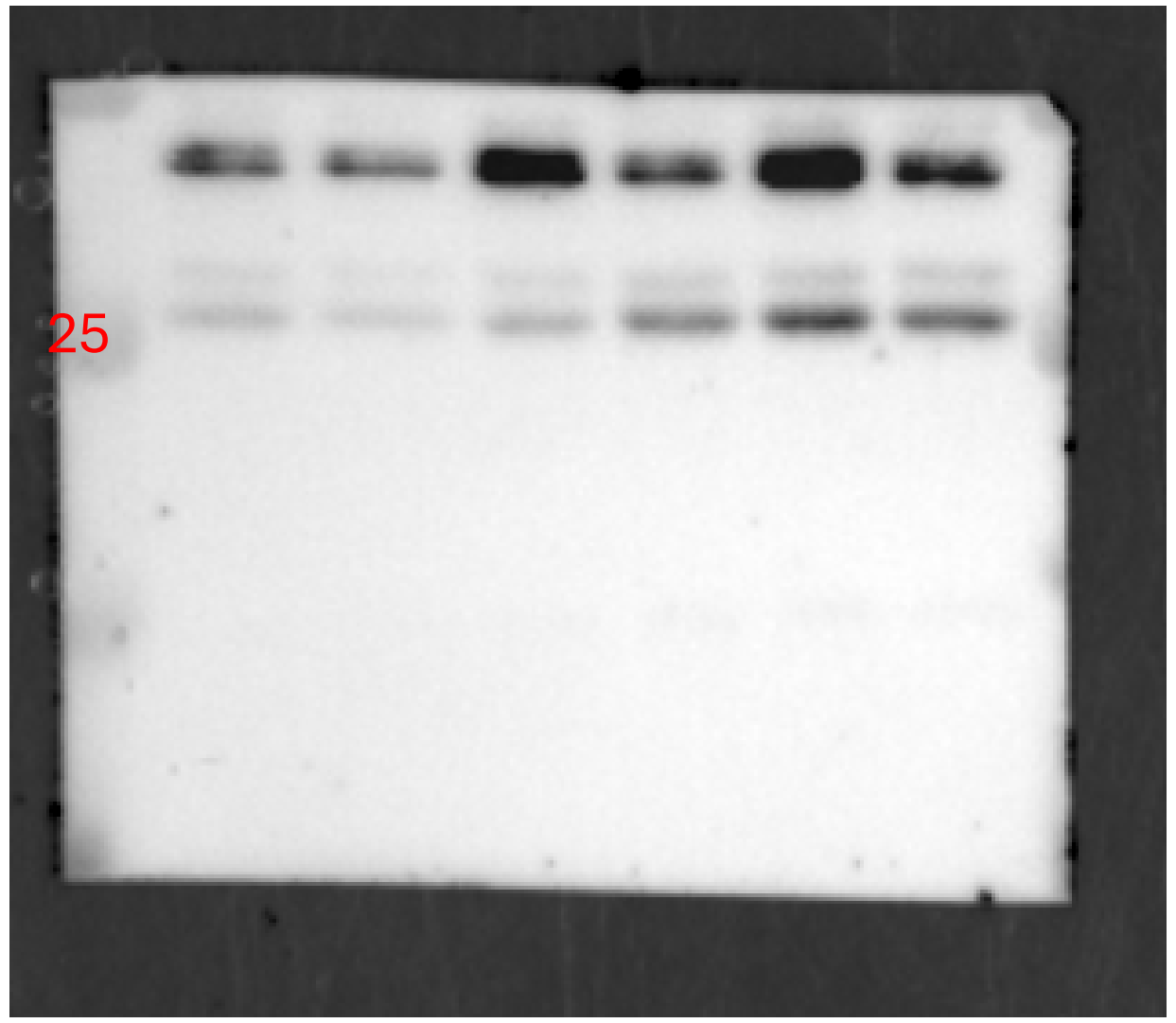

Medium

GFP (1 sec accumulation mode, max substrate)

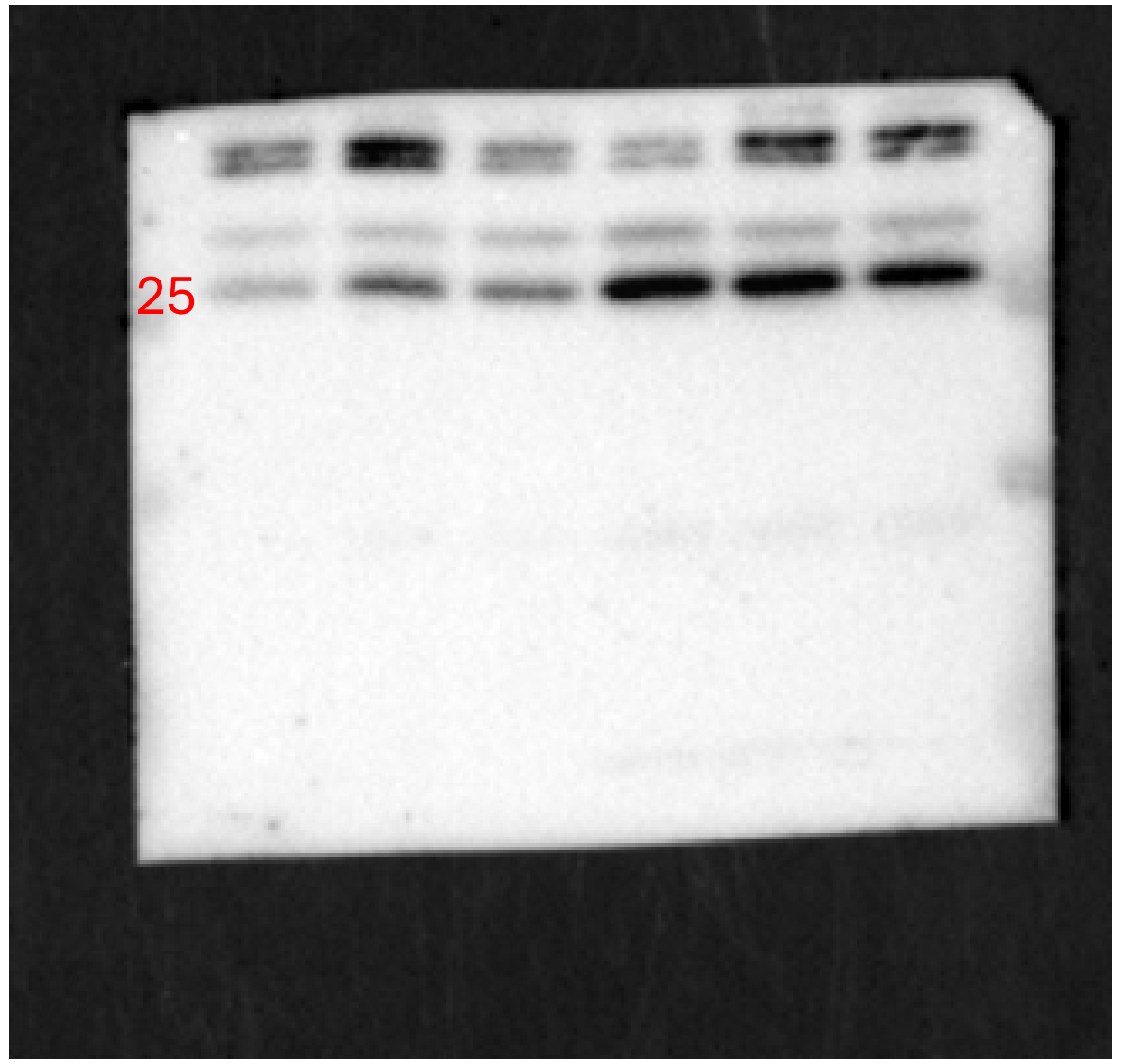

High

Supplementary Figure 11C – rep 1

ZFC3H1 (7.2 sec exposure – accumulation mode, max substrate)

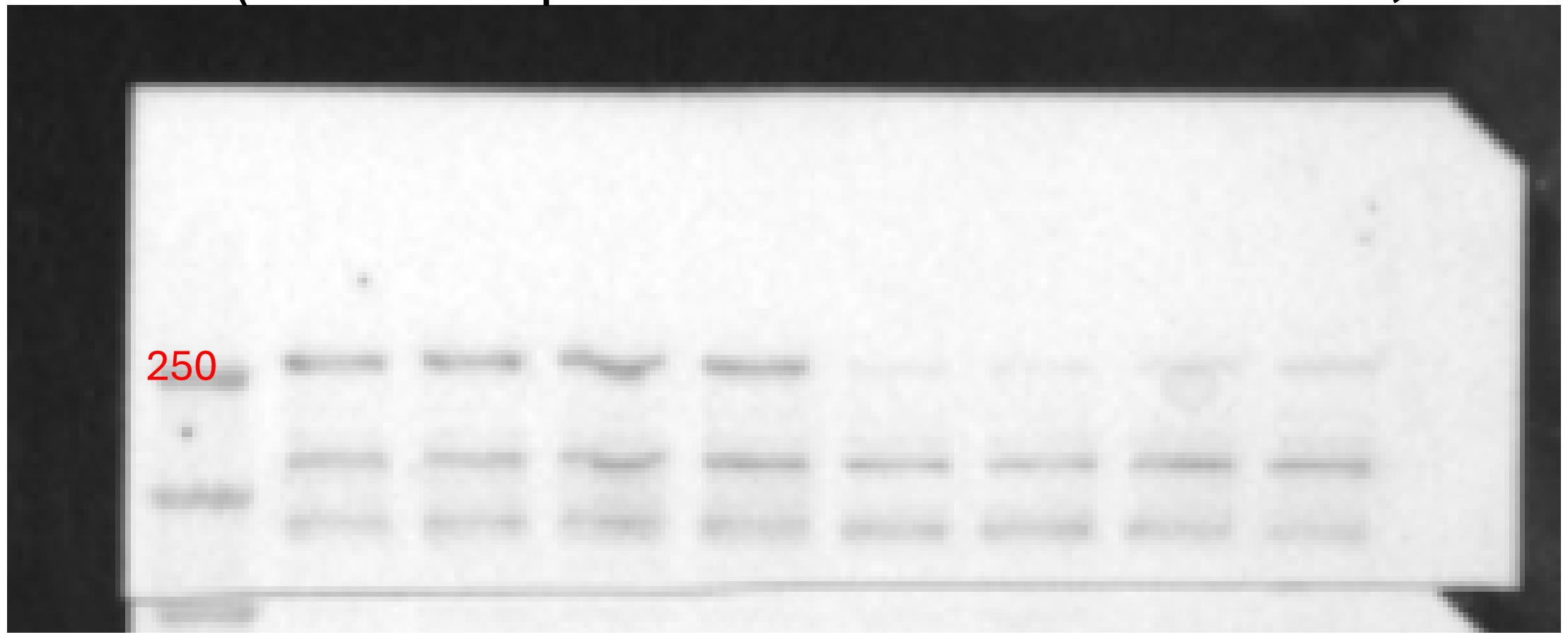

B-tubulin (0.5 sec exposure – accumulation mode)

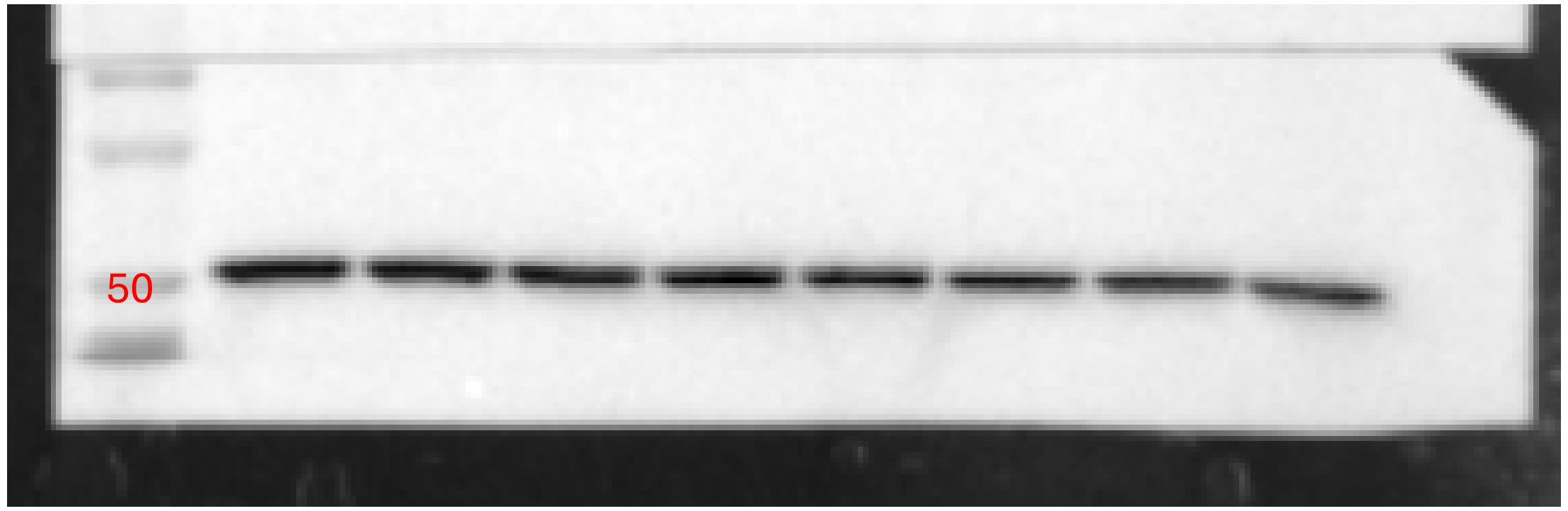

Lane	Sample
1	Ladder
2	Ctrl guide 1, Low reporter
3	Ctrl guide 1, Medium Reporter
4	Ctrl guide 2, Low Reporter
5	Ctrl guide 2, Medium Reporter
6	ZFC3H1 guide 1, Low reporter
7	ZFC3H1 guide 1, Medium Reporter
8	ZFC3H1 guide 2, Low reporter
9	ZFC3H1 guide 2, Medium Reporter

Supplementary Figure 11C – rep 1

B-Tubulin for mCherry (10.3 sec accumulation mode)

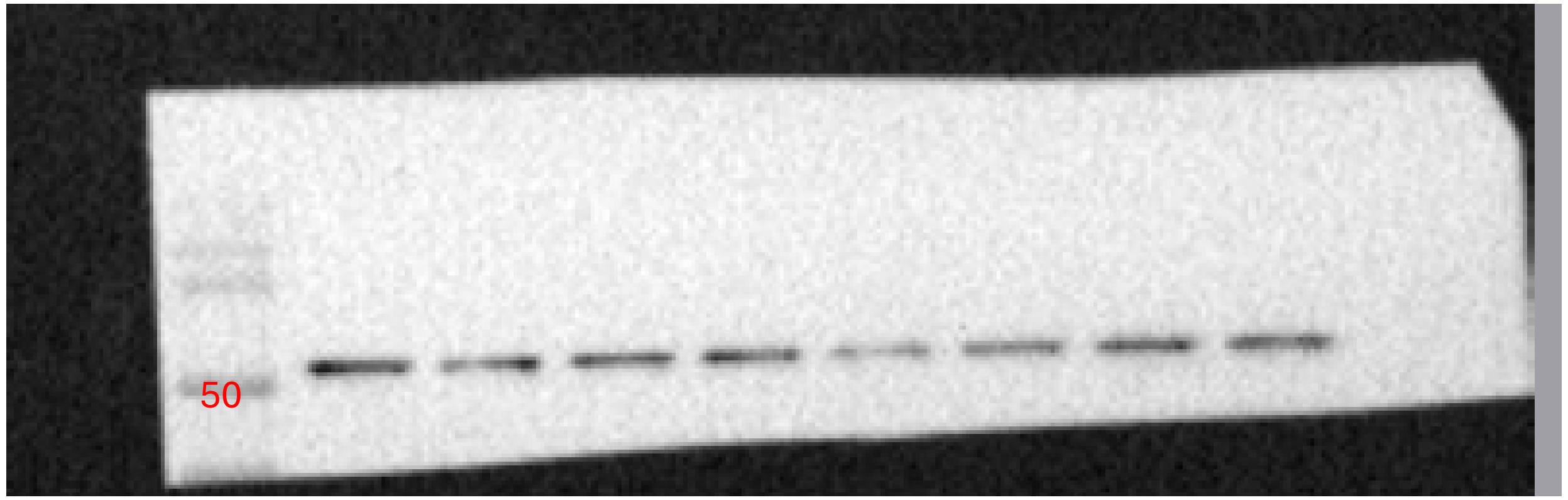

mCherry (4.1 sec accumulation mode)

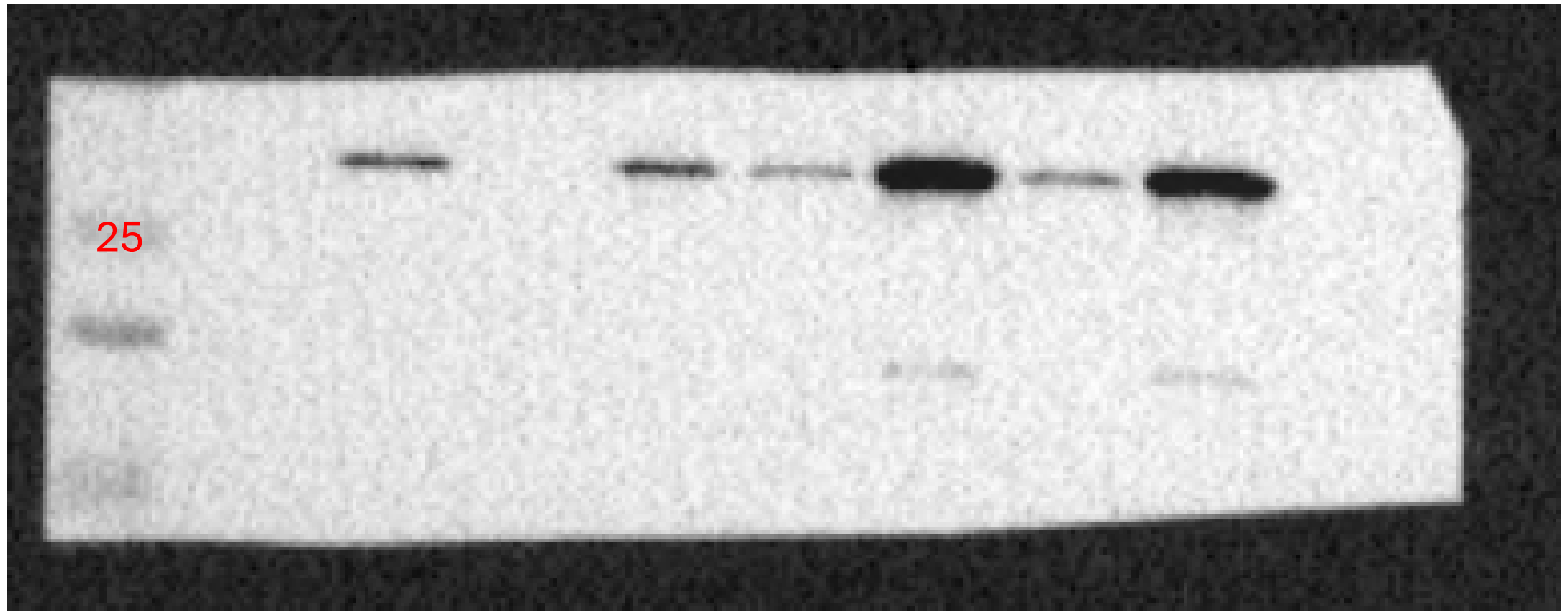

mCherry (4.1 sec accumulation mode, max substrate)

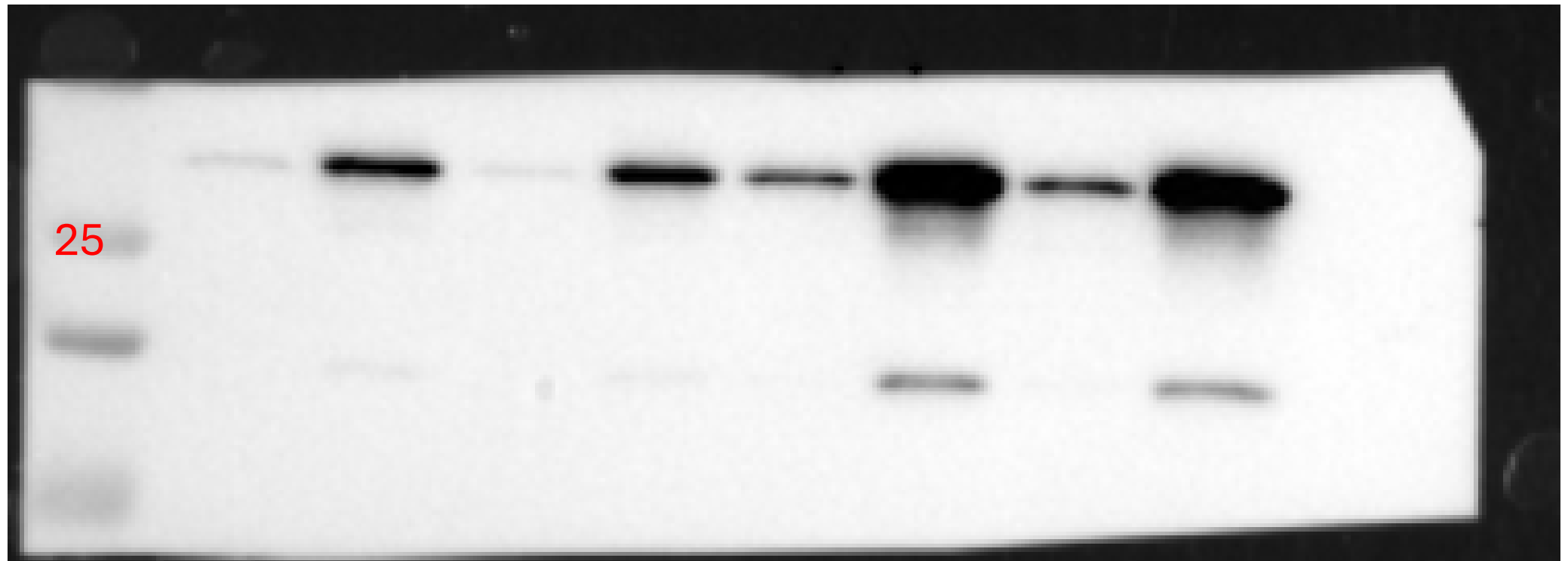

B-Tubulin for GFP (10.3 sec accumulation mode)

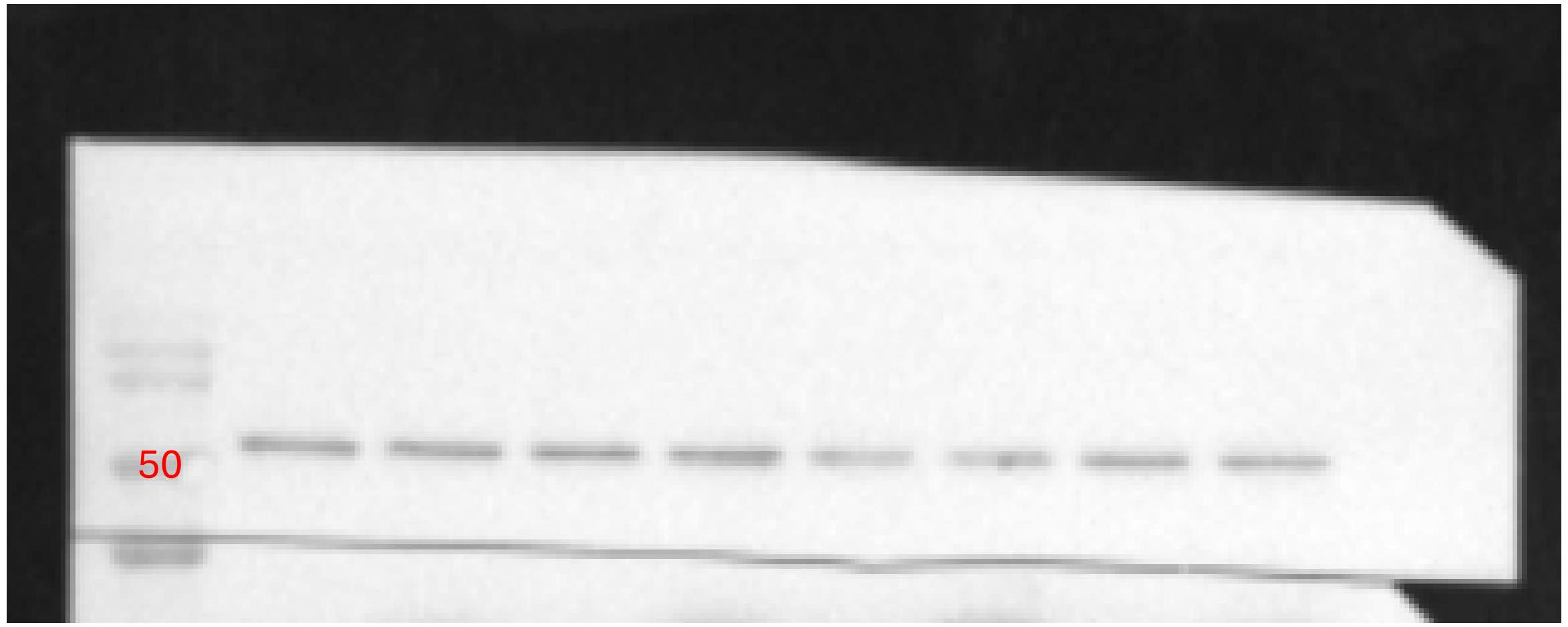

GFP (.2 sec)

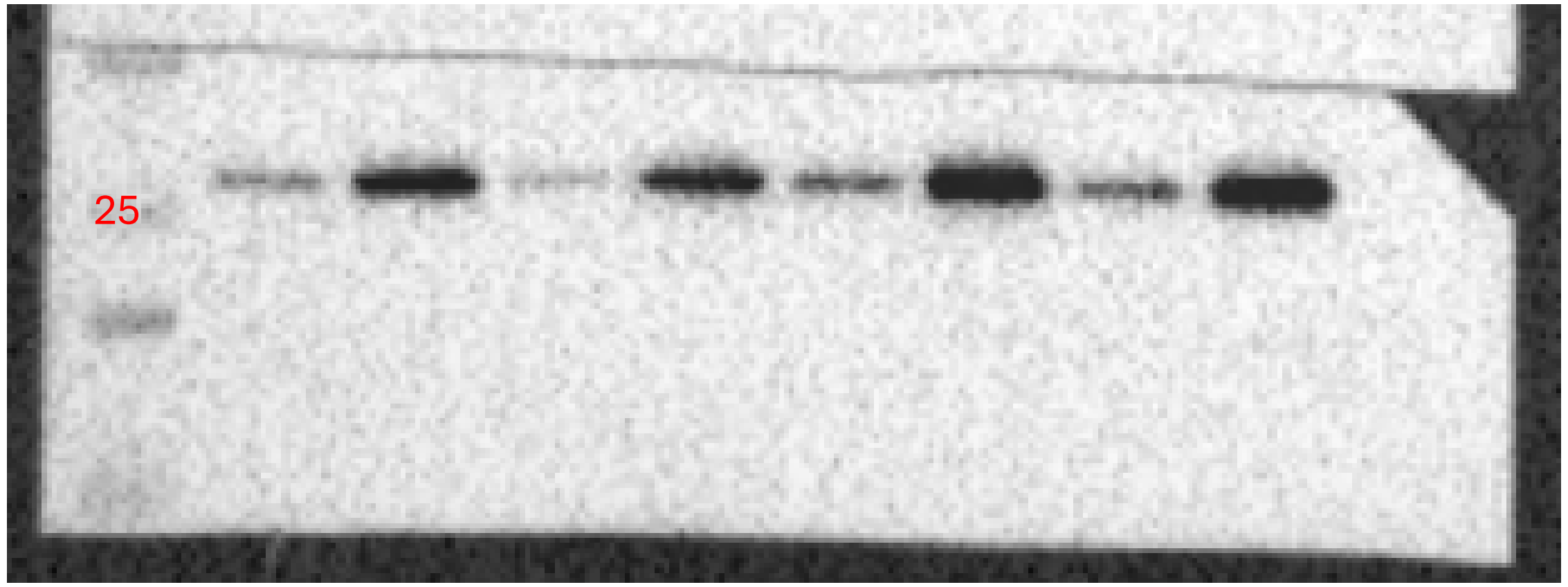

Supplementary Figure 11C – rep 2

ZFC3H1 (22.7 sec exposure – accumulation mode, max substrate)

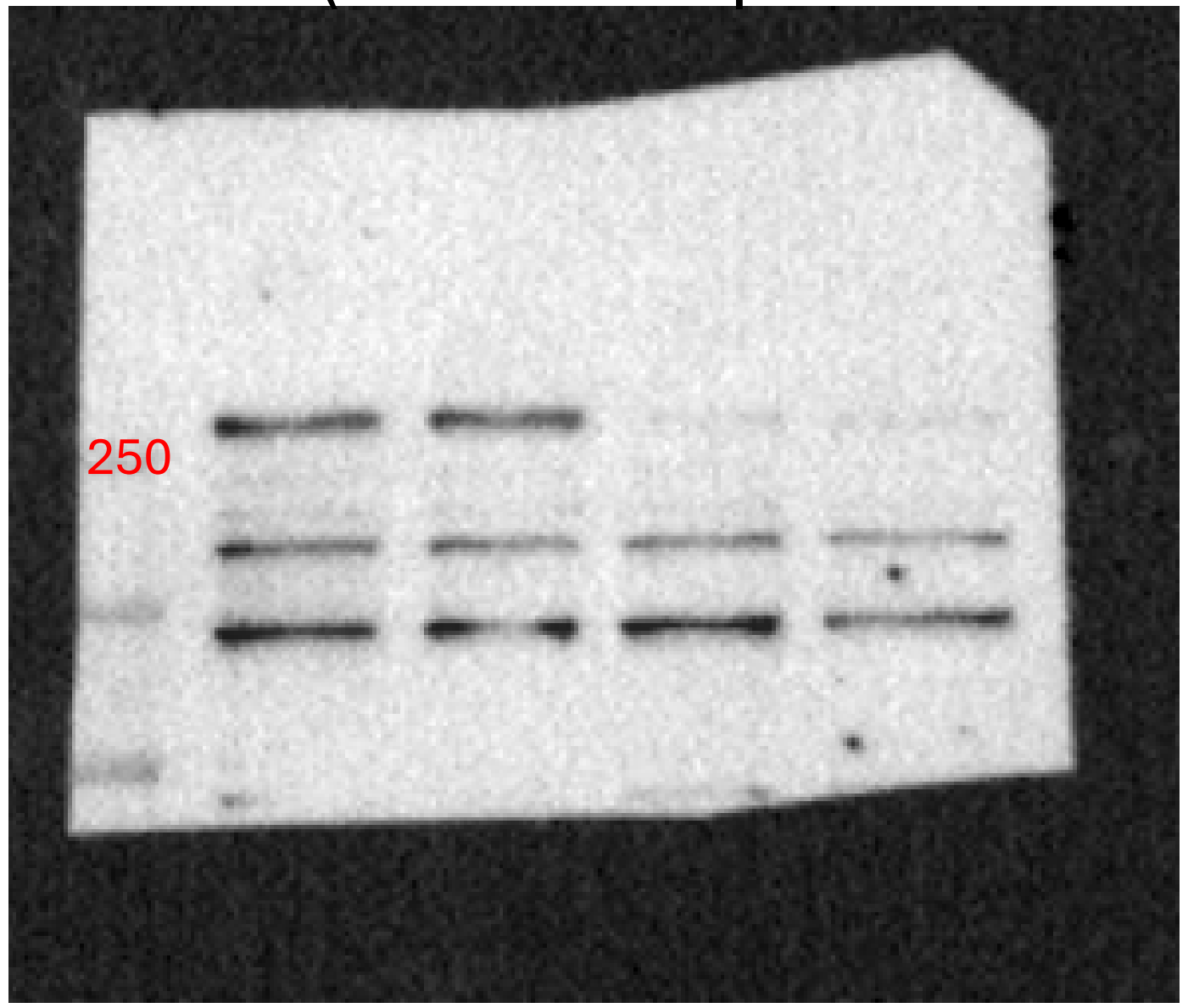

B-tubulin (7.2 sec exposure – accumulation mode)

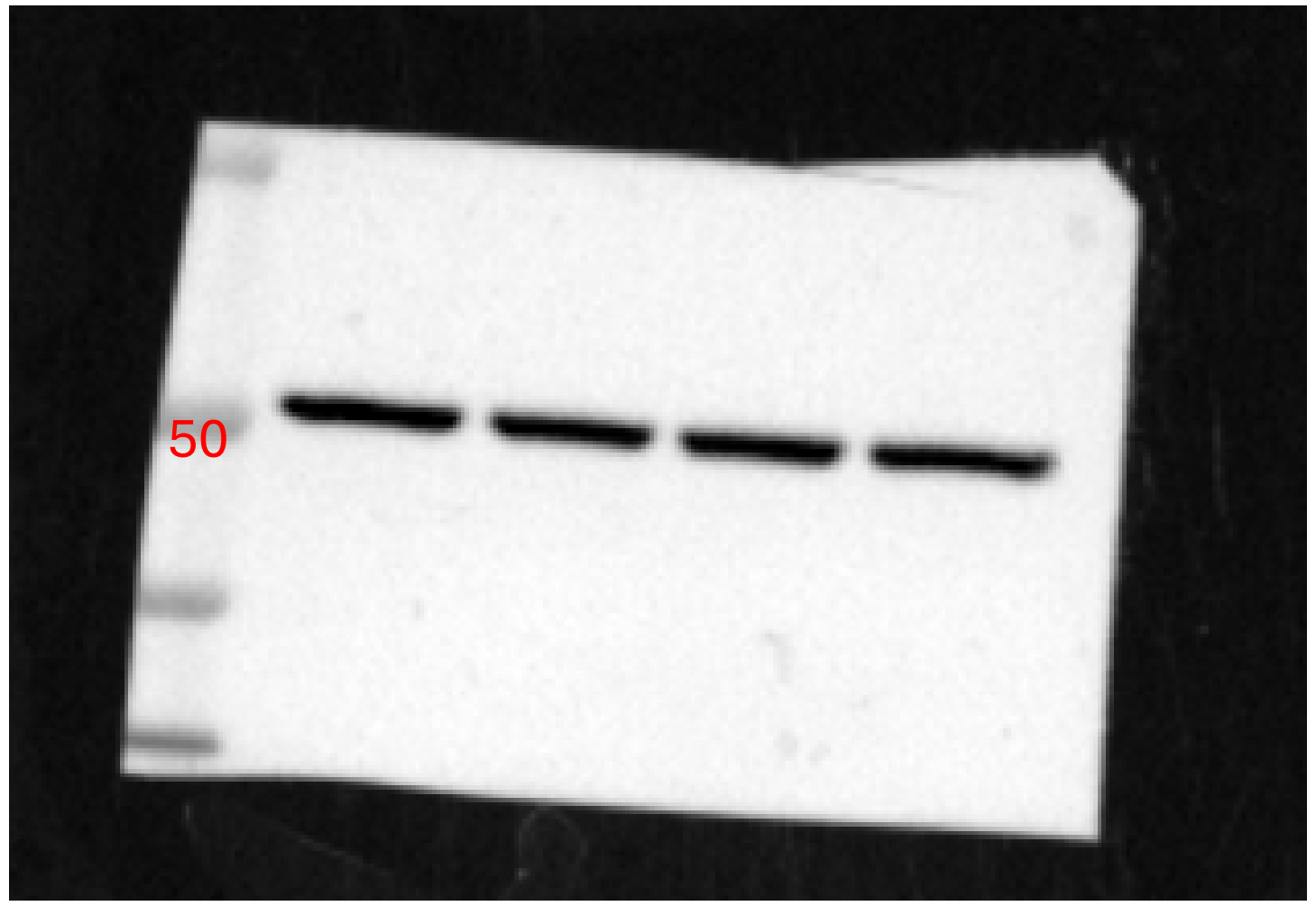

Lane	Sample
1	Ladder
2	Ctrl guide 2, Low reporter
3	Ctrl guide 2, Medium Reporter
6	ZFC3H1 guide 2, Low reporter
7	ZFC3H1 guide 2, Medium Reporter

Supplementary Figure 11C – rep 2

B-tubulin for GFP (7.2 sec exposure – accumulation mode)

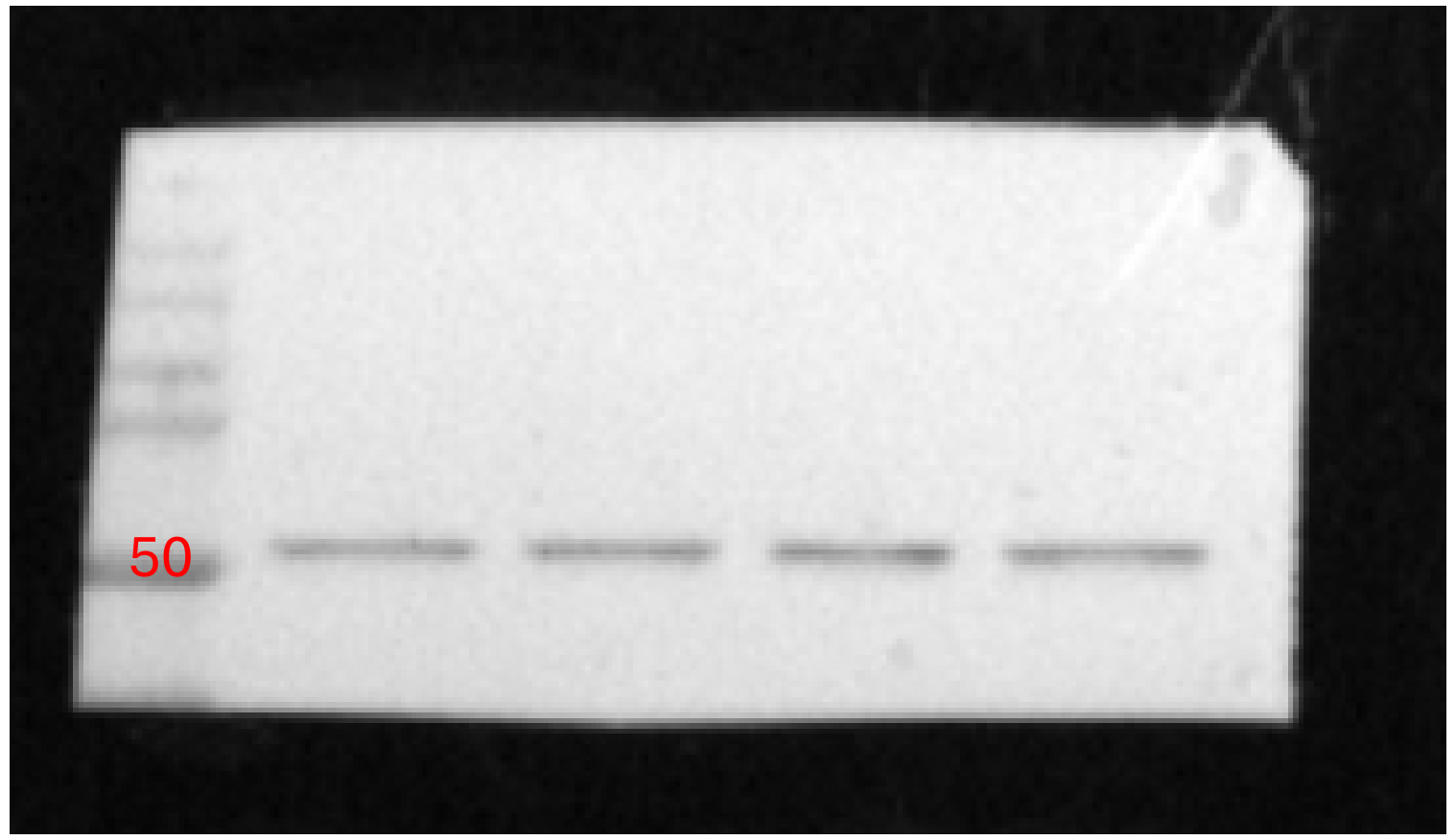

GFP (1 sec exposure – accumulation mode)

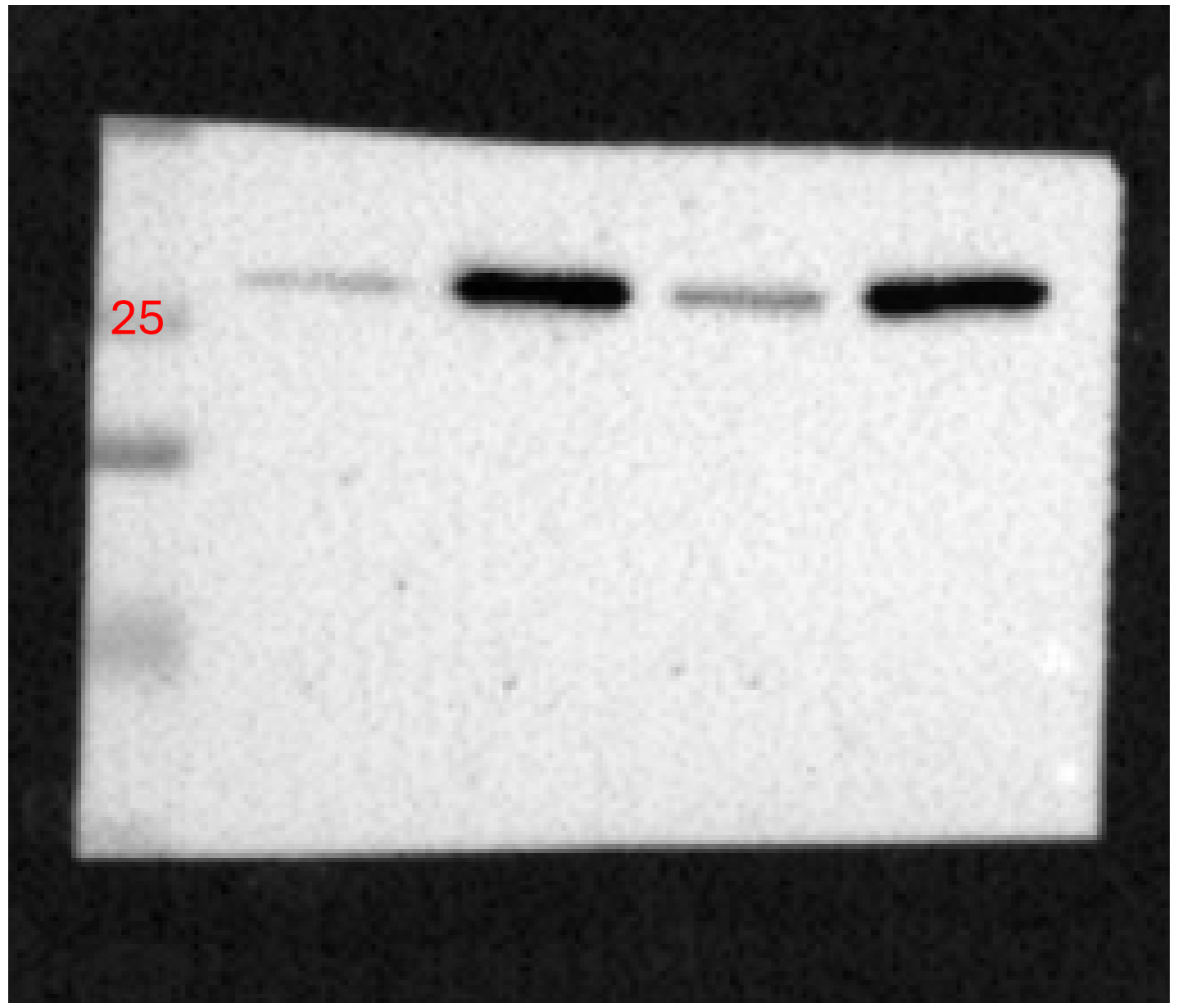

B-tubulin for mCherry (10.3 sec exposure – accumulation mode)

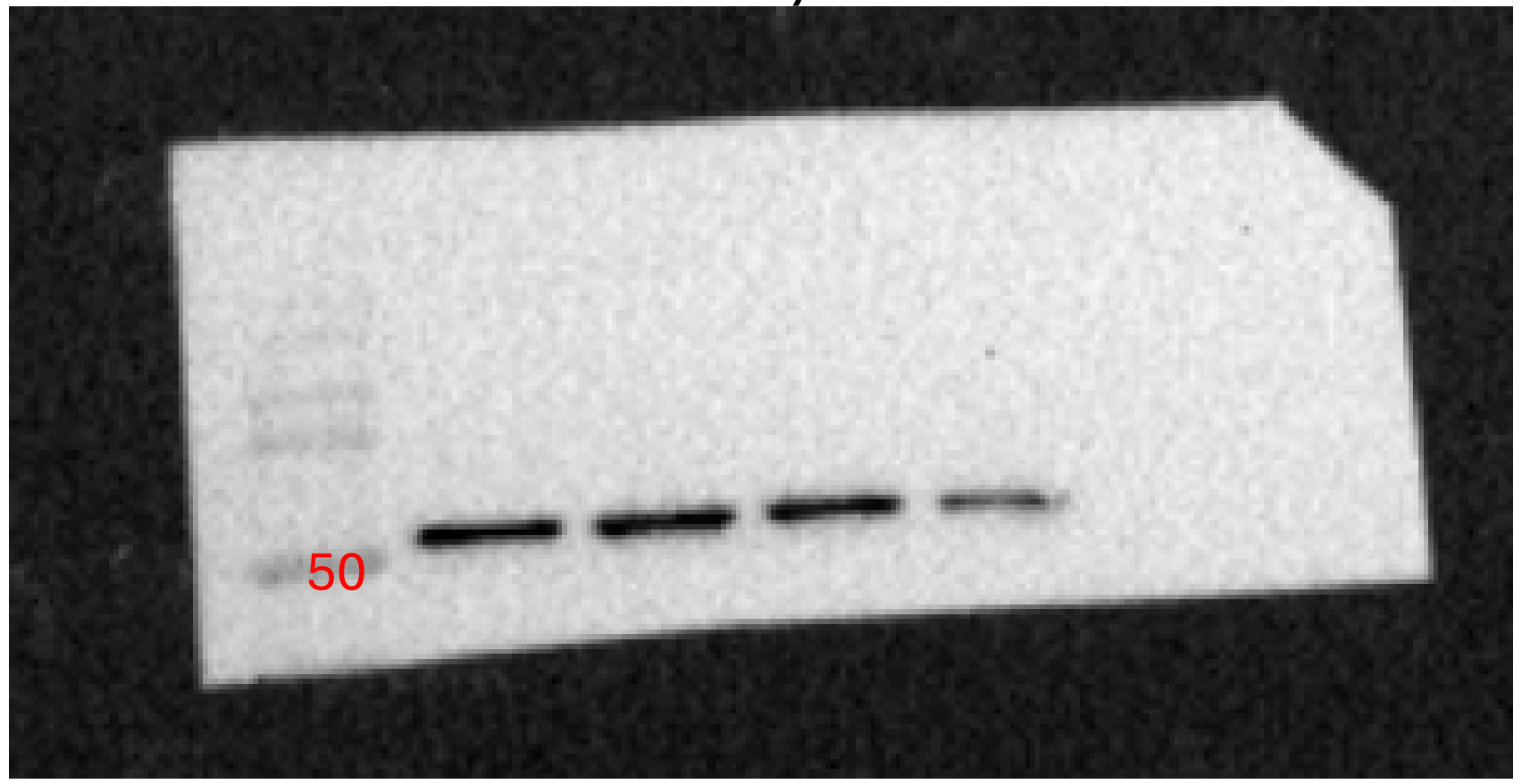

mCherry (4.1 sec exposure – accumulation mode)

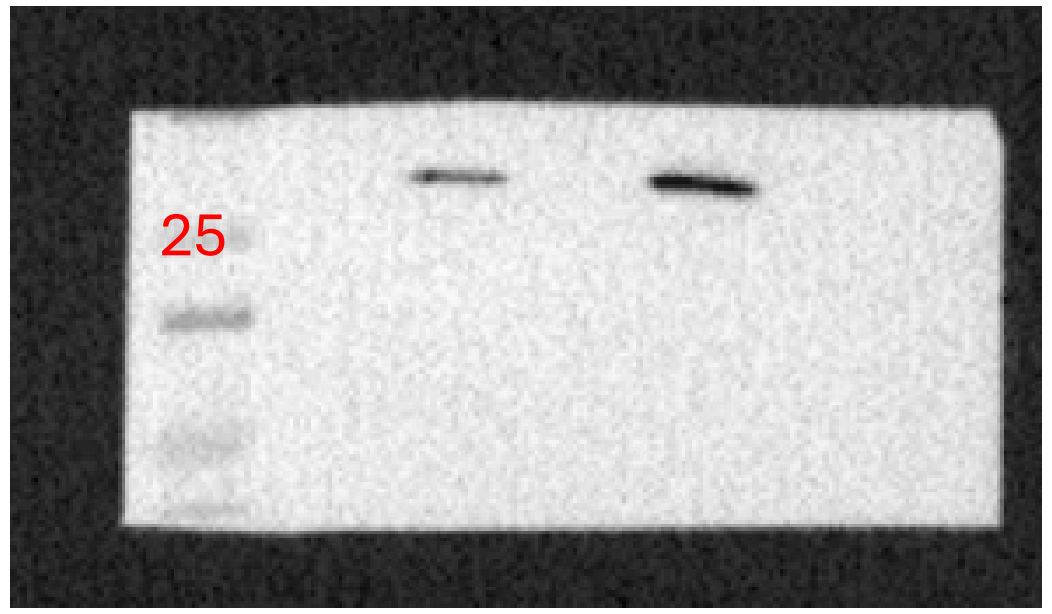

mCherry (13.1 sec exposure – accumulation mode, max substrate)

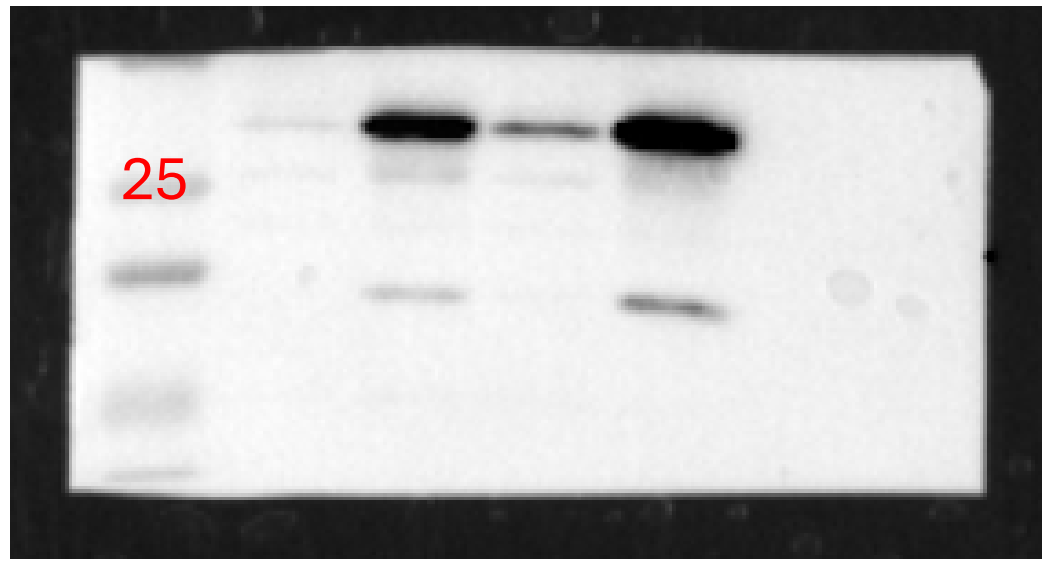

Supplementary Figure 13A – rep 1

PABPN1 (7.2 sec exposure –accumulation mode)

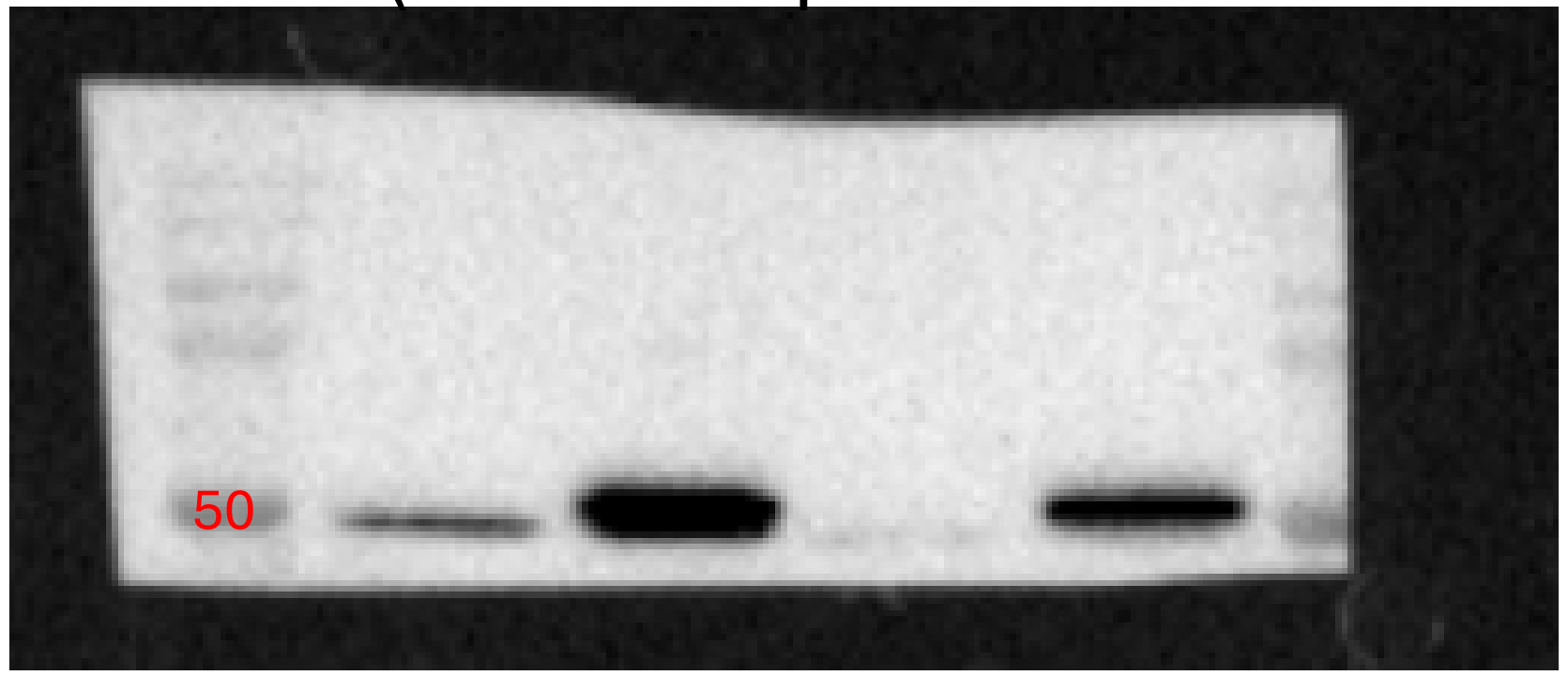

GAPDH (13.4 sec exposure – accumulation mode, max substrate)

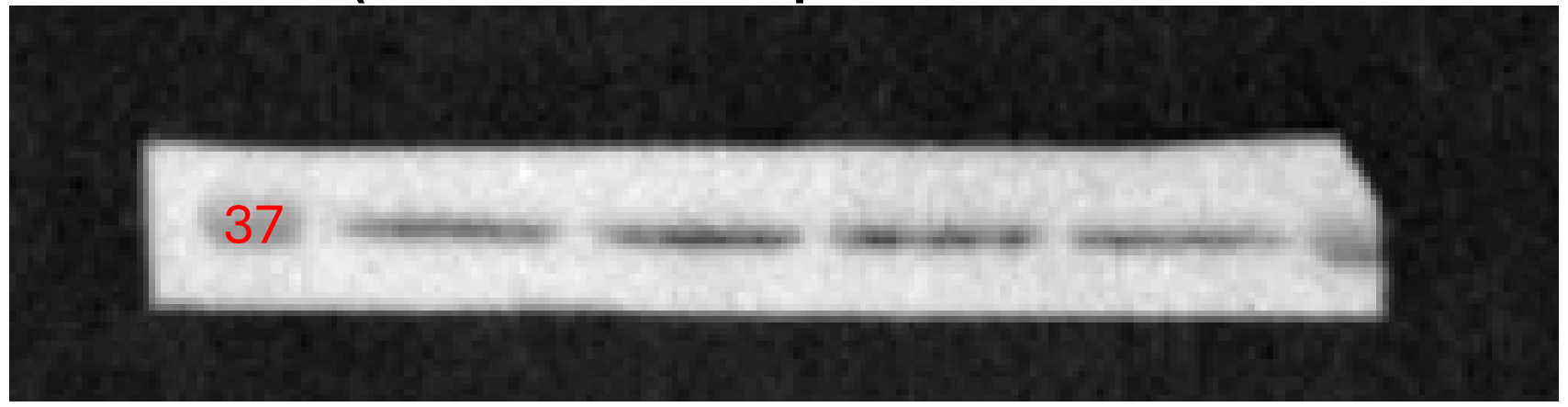

mCherry (7.2 sec exposure – accumulation mode)

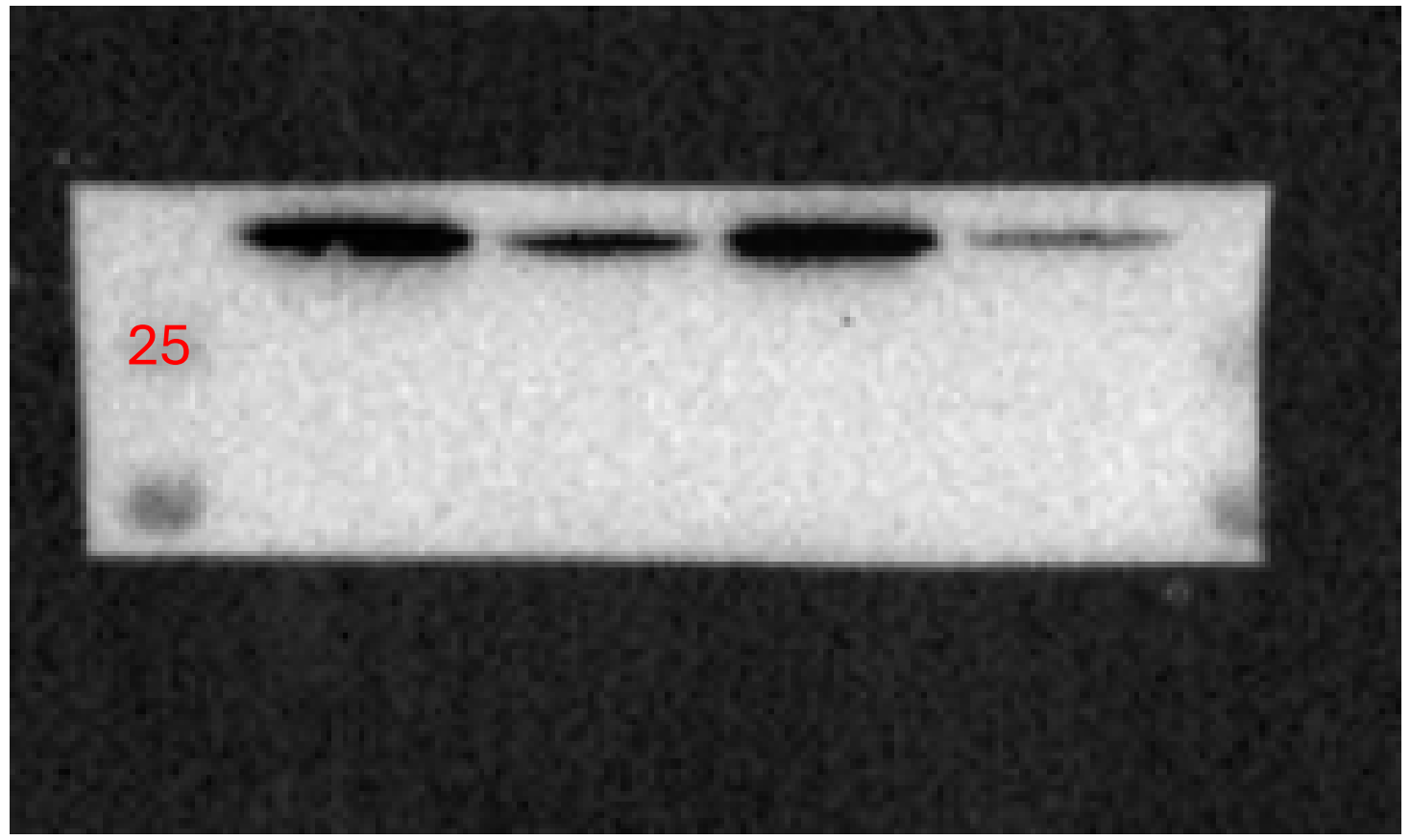

Lane	Sample
1	Ladder
2	Ctrl guide 2, Empty Vector
3	Ctrl guide 2, FLAG-PABPN1
4	PABPN1 guide 2, Empty Vector
5	PABPN1 guide 2, FLAG-PABPN1

Supplementary Figure 13A – rep 1

B-tubulin (13.4 sec exposure – accumulation mode, max substrate)

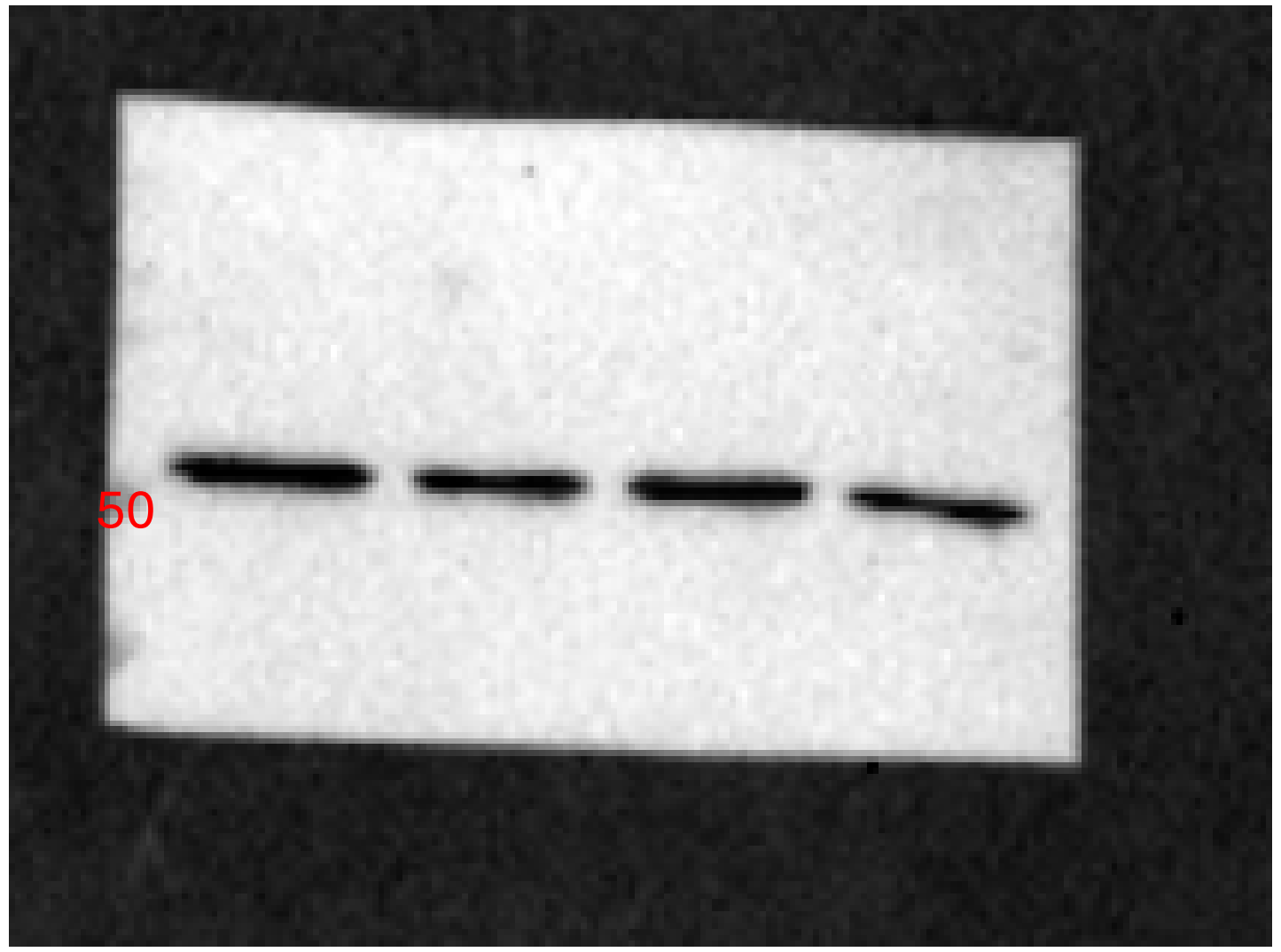

GFP (25.8 sec exposure – accumulation mode, max substrate)

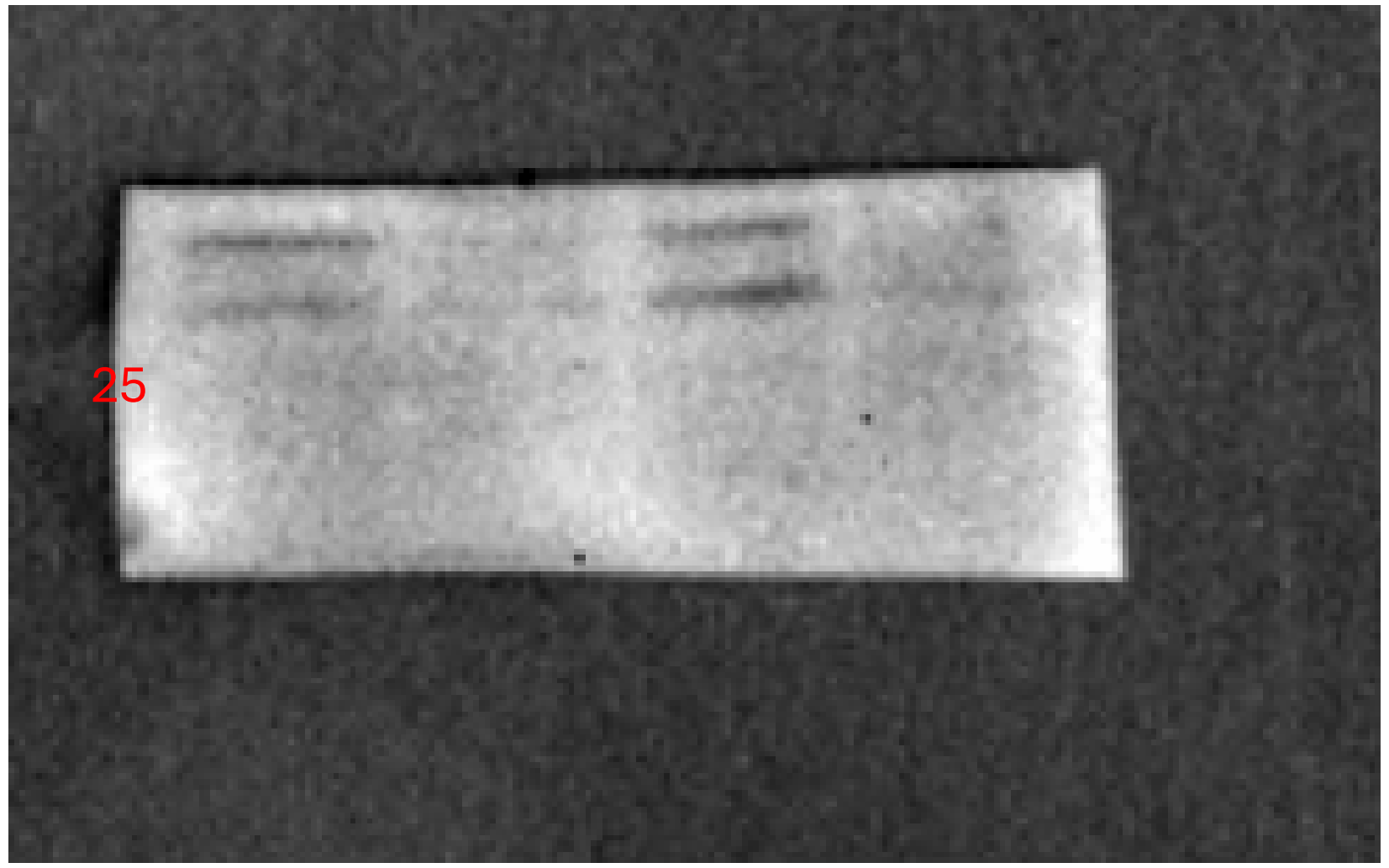

Supplementary Figure 13A – rep 2

PABPN1 (1 sec exposure –accumulation mode)

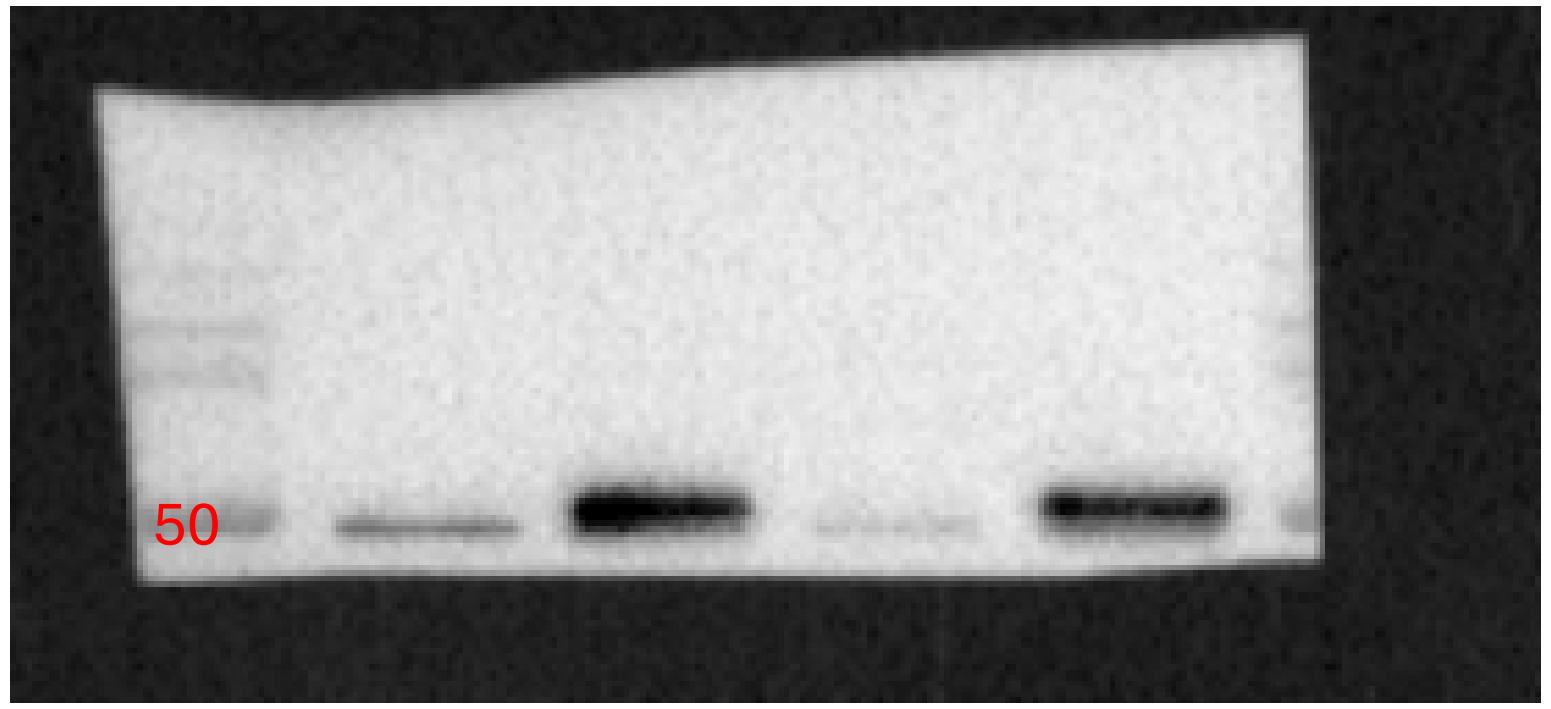

GAPDH (13.4 sec exposure – accumulation mode)

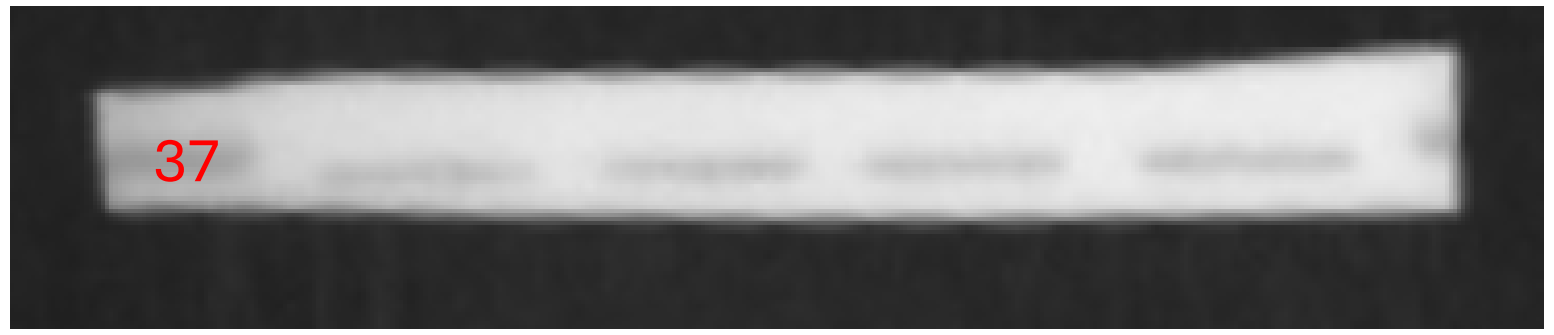

mCherry (.25 sec exposure)

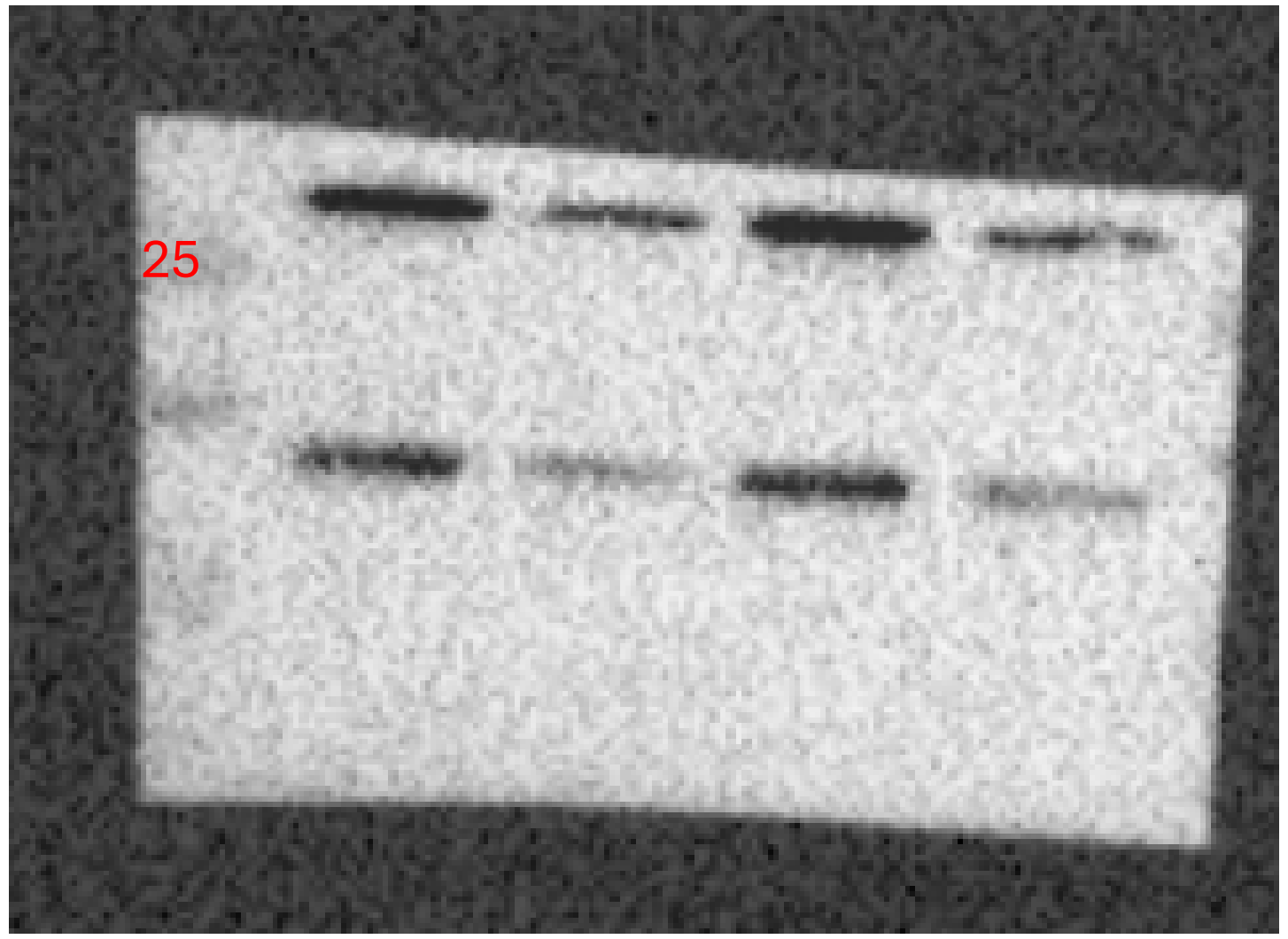

Lane	Sample
1	Ladder
2	Ctrl guide 2, Empty Vector
3	Ctrl guide 2, FLAG-PABPN1
4	PABPN1 guide 2, Empty Vector
5	PABPN1 guide 2, FLAG-PABPN1

Supplementary Figure 13A- rep 2

B-tubulin (4.1 sec exposure – accumulation mode)

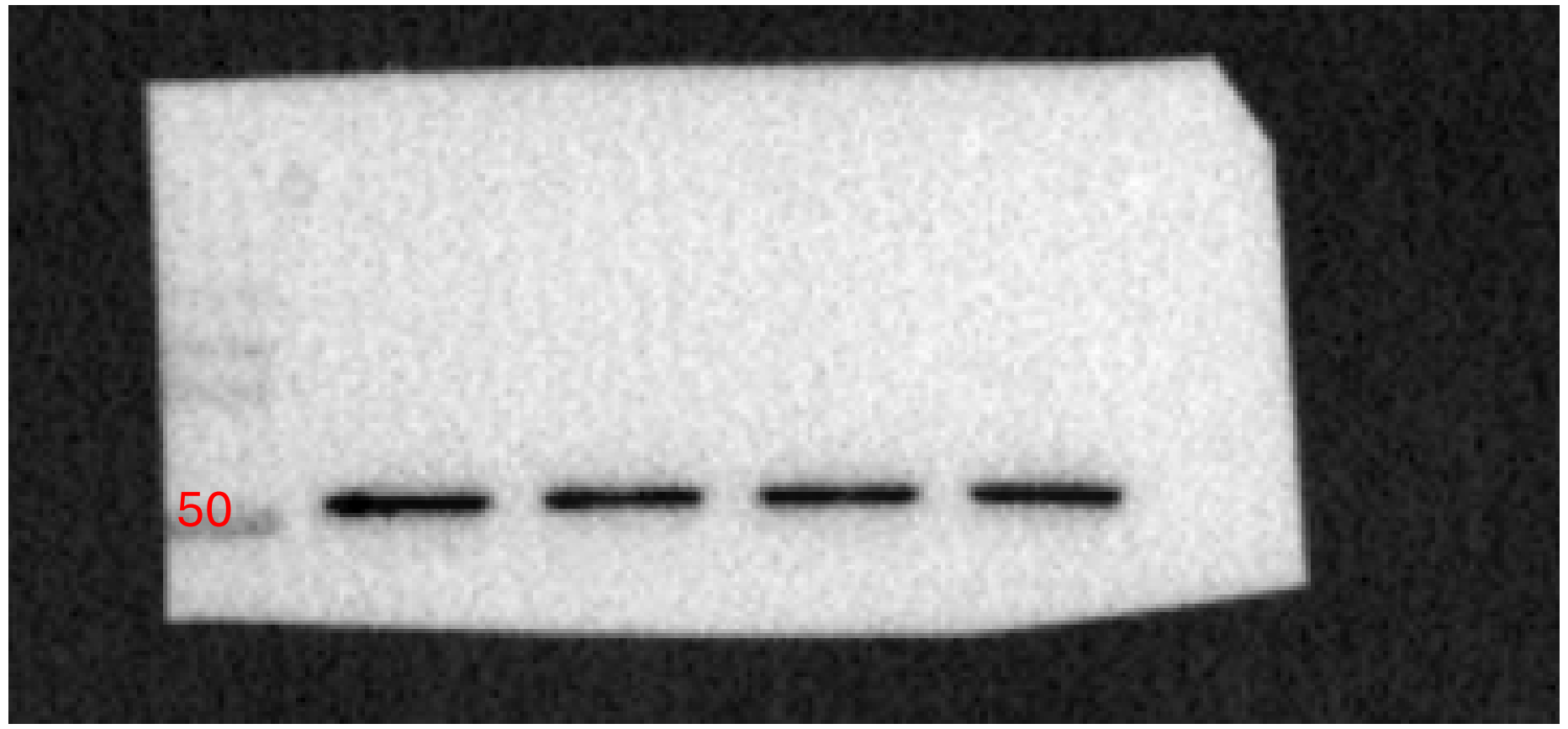

GFP (7.2 sec exposure – accumulation mode, max substrate)

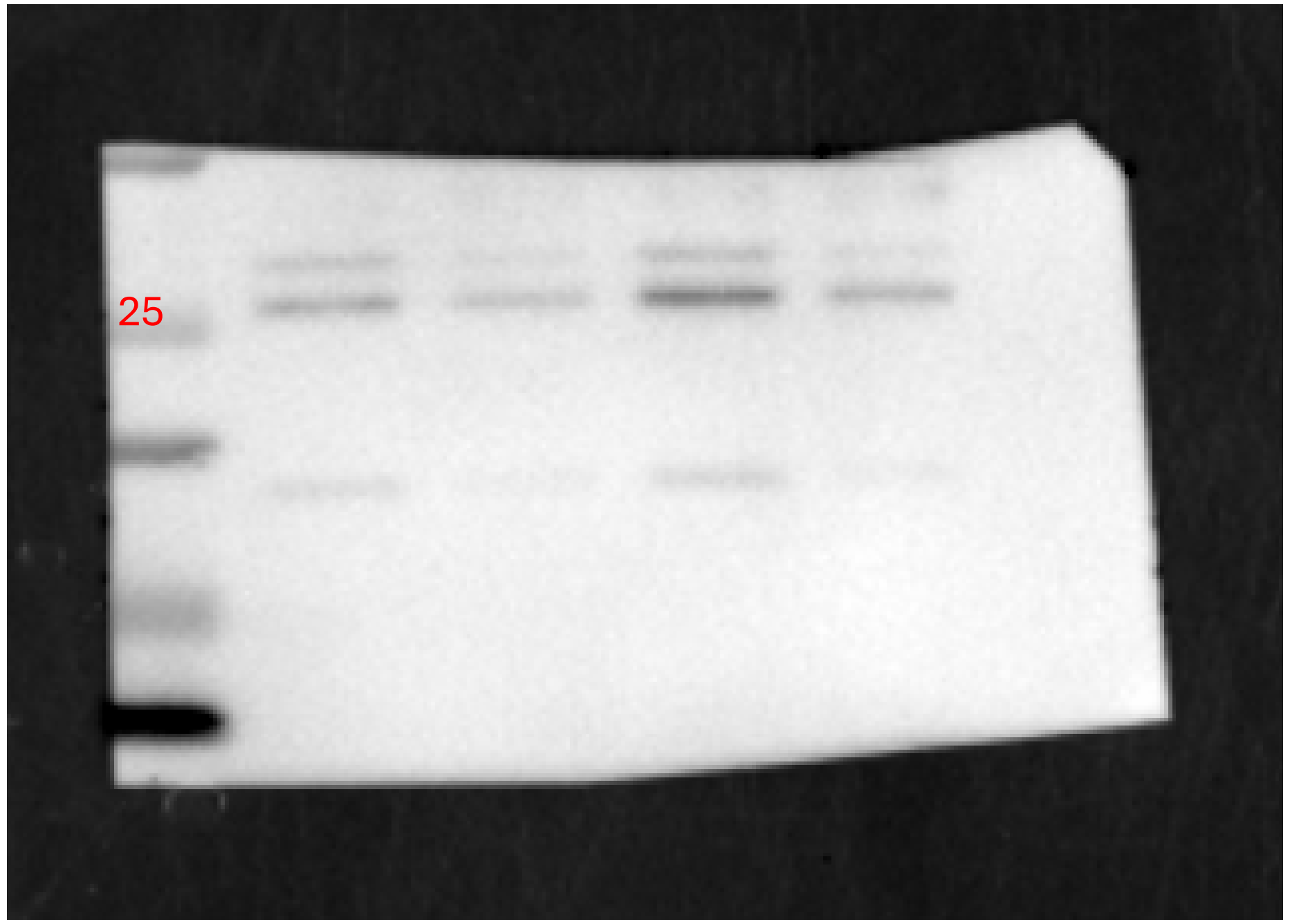

Supplementary Figure 13B – rep 1

PABPN1 (7.2 sec exposure –accumulation mode)

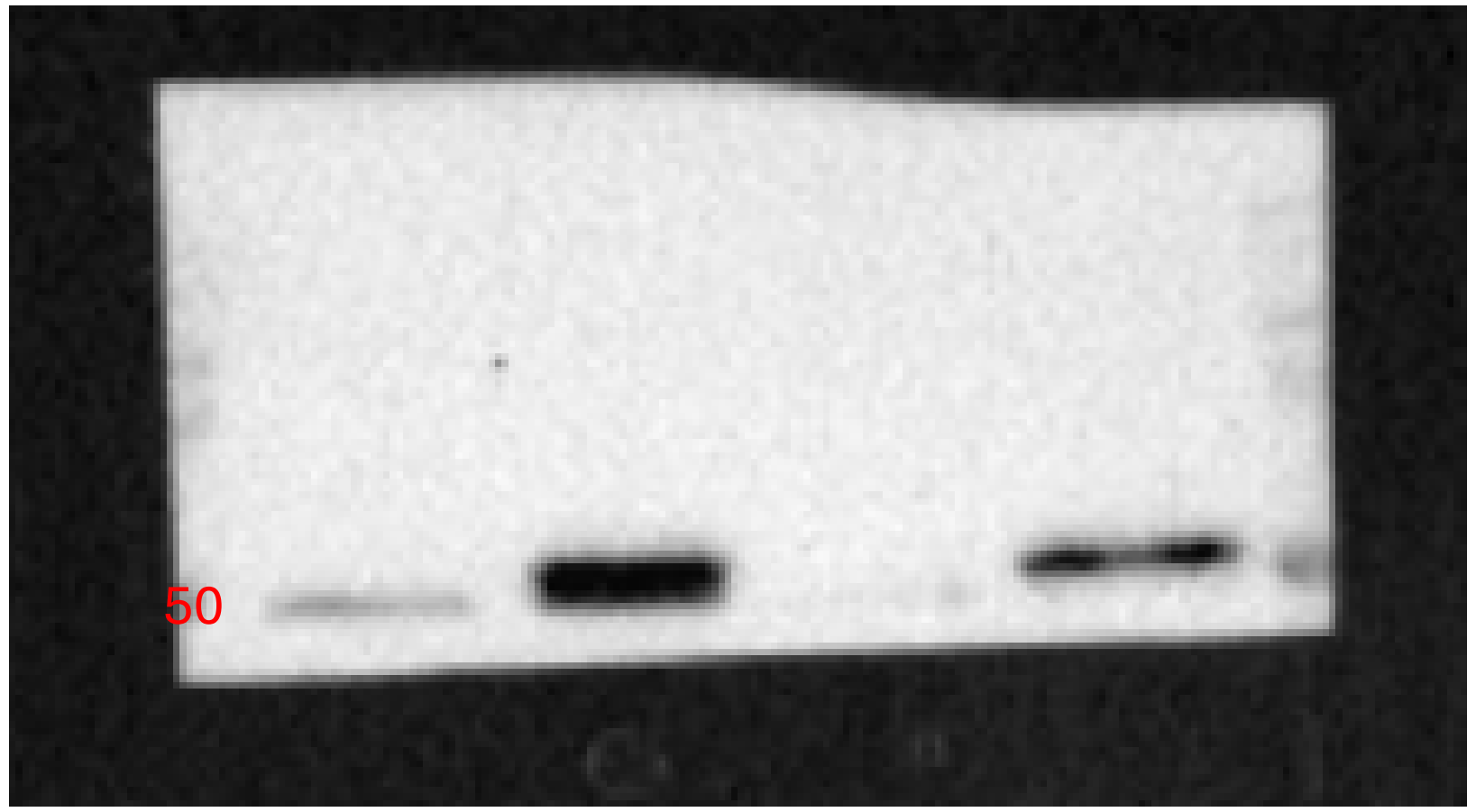

GAPDH (13.4 sec exposure – accumulation mode, max substrate)

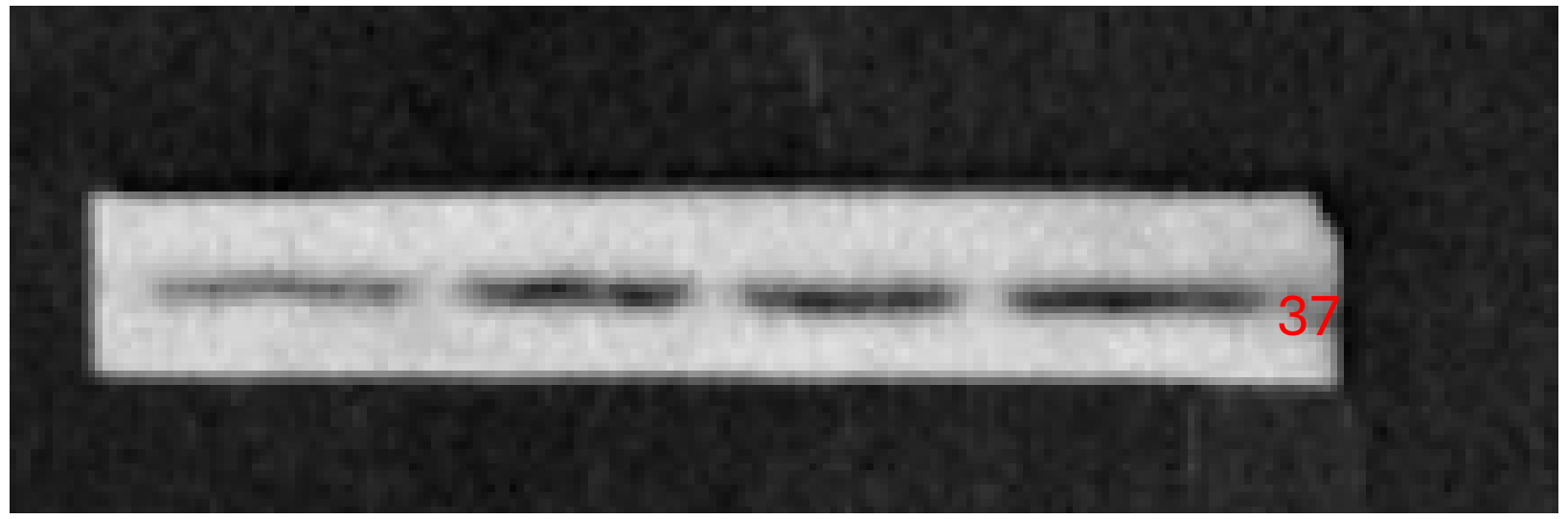

GFP (7.2 sec exposure – accumulation mode)

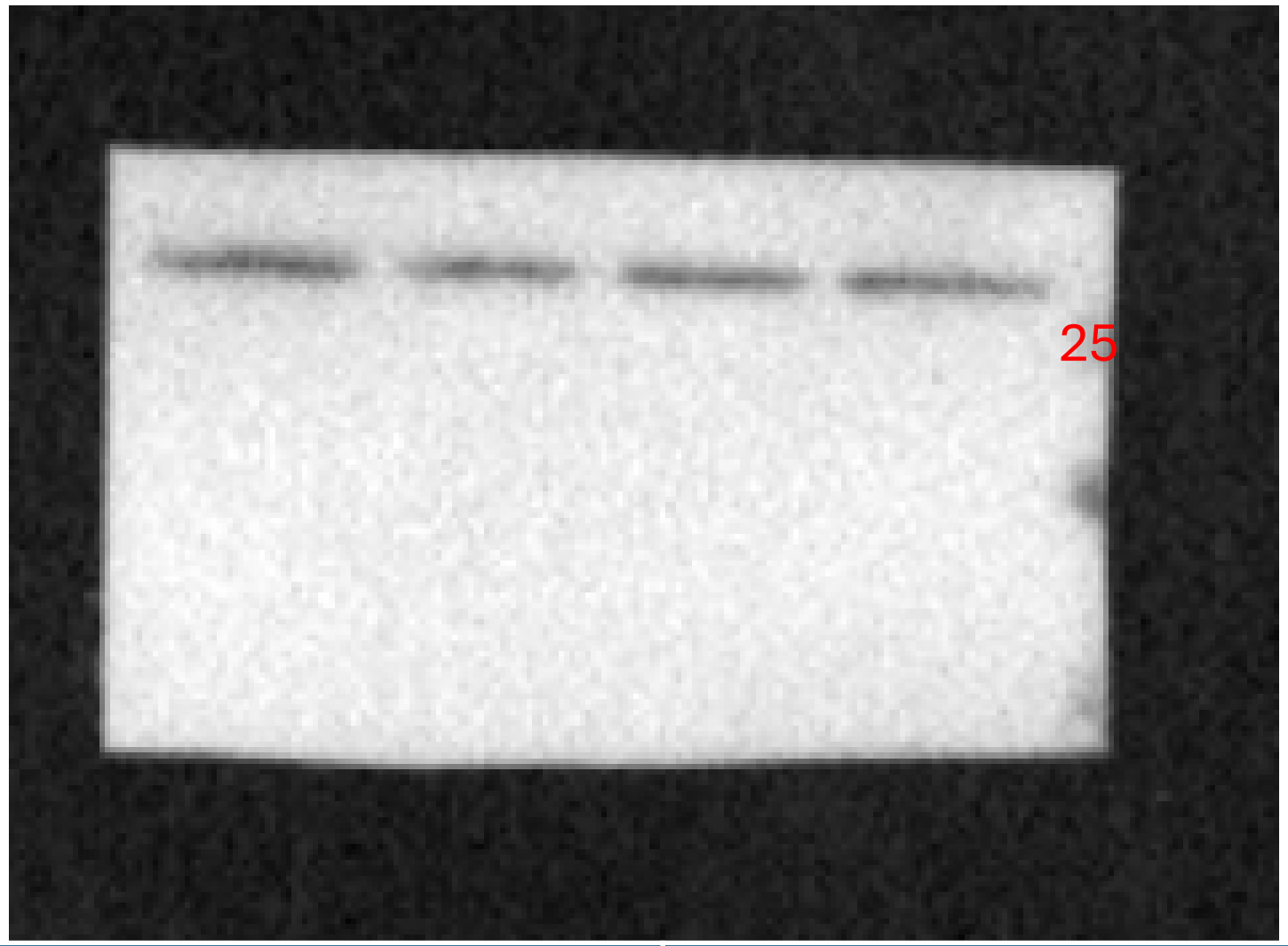

Lane	Sample
1	Ladder
2	Ctrl guide 2, Empty Vector
3	Ctrl guide 2, FLAG-PABPN1
4	PABPN1 guide 2, Empty Vector
5	PABPN1 guide 2, FLAG-PABPN1

Supplementary Figure 13B –rep 1

B-tubulin (13.4 sec exposure – accumulation mode, max substrate)

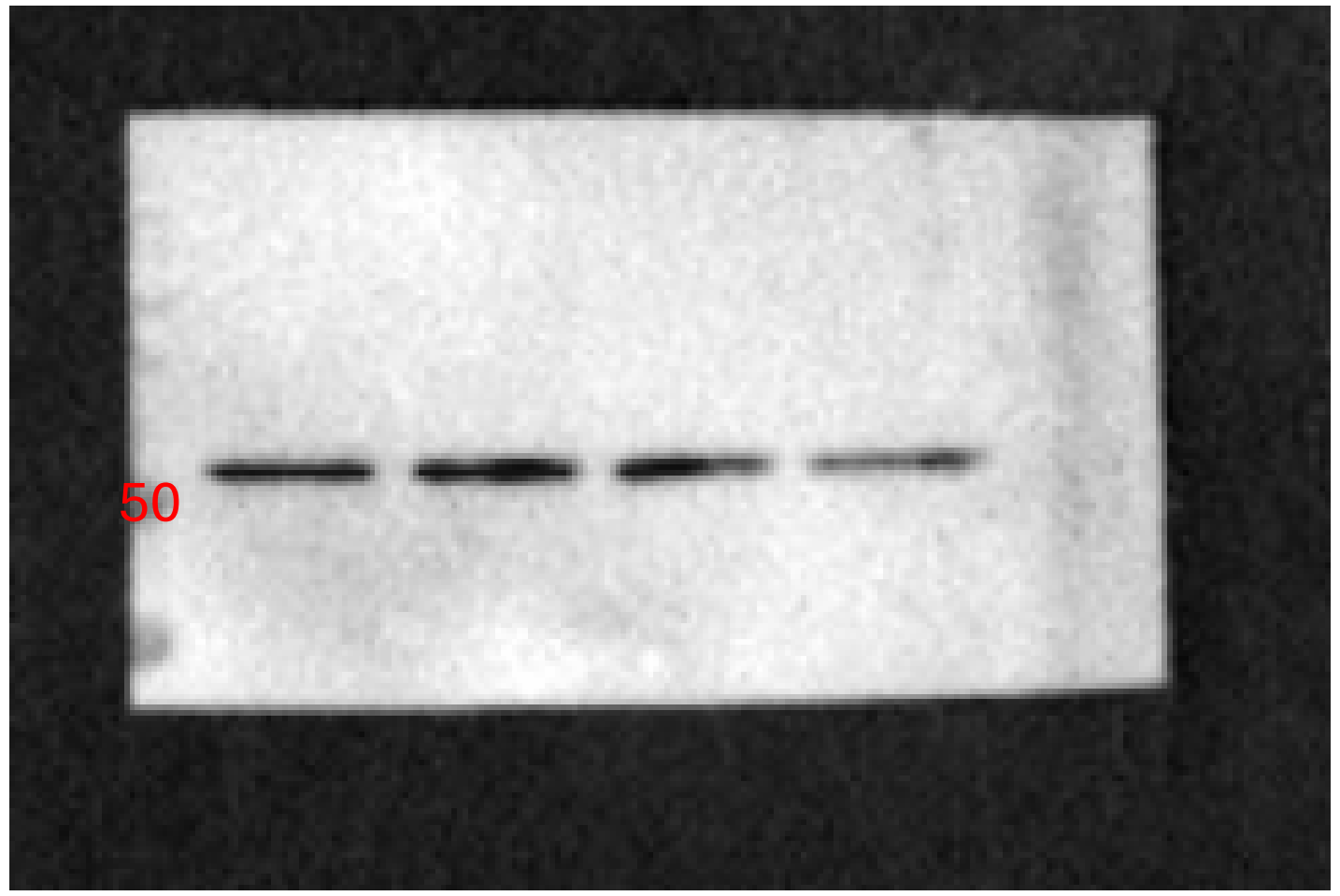

mCherry (25.8 sec exposure – accumulation mode, max substrate)

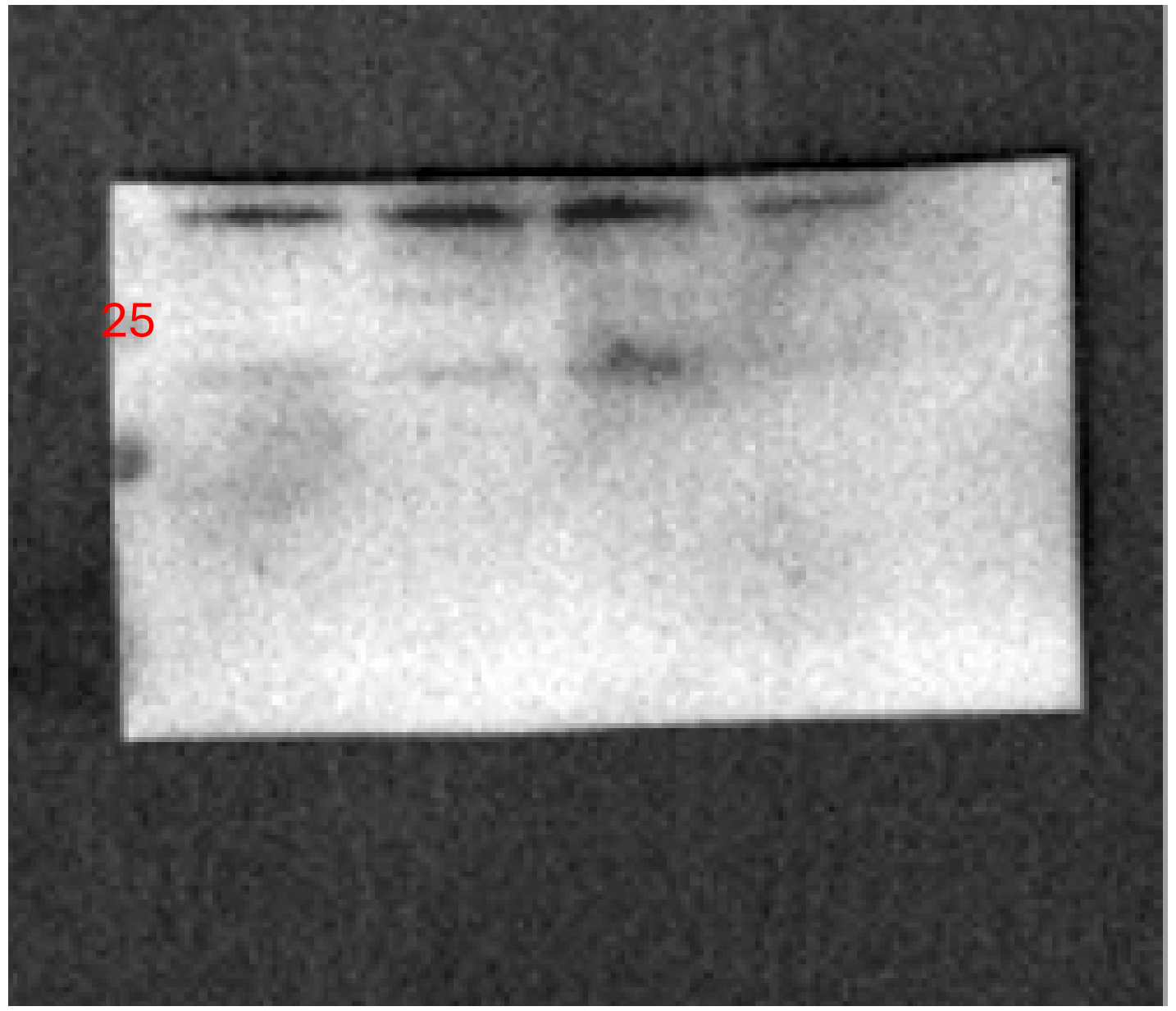

Supplementary Figure 13B- rep 2

PABPN1 (1 sec exposure –accumulation mode)

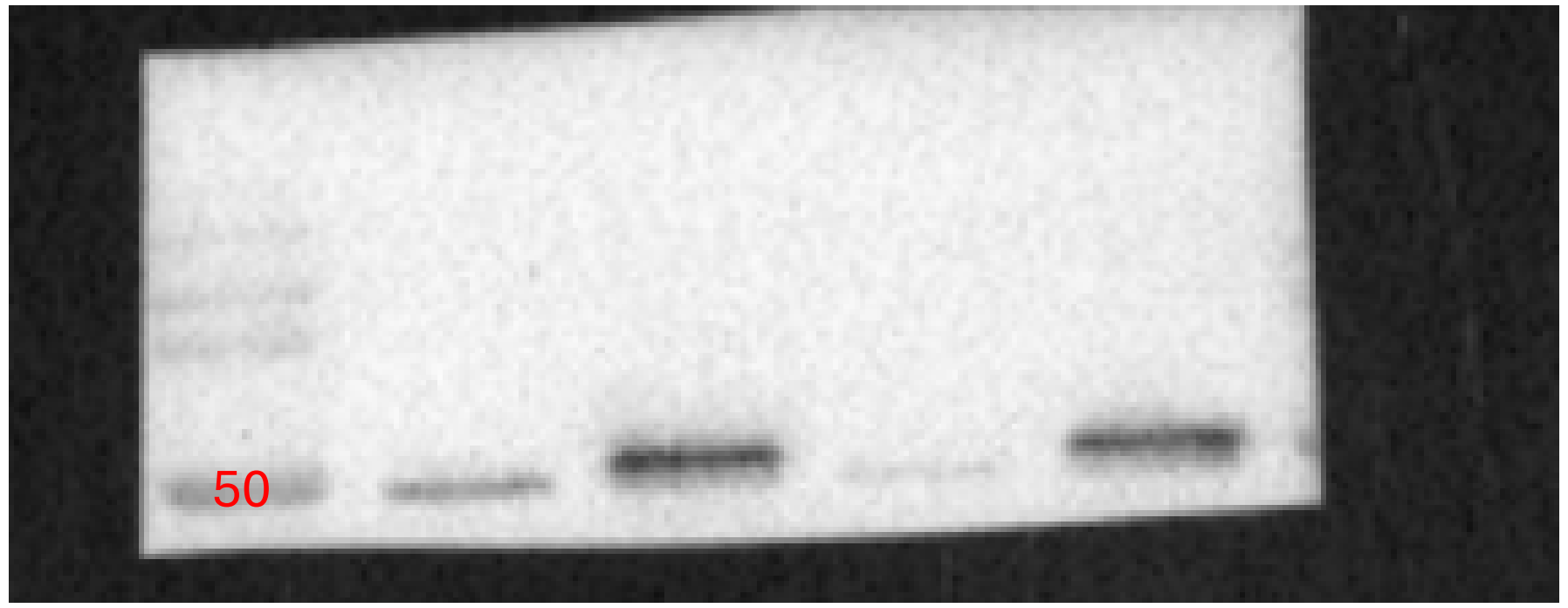

GAPDH (13.4 sec exposure – accumulation mode)

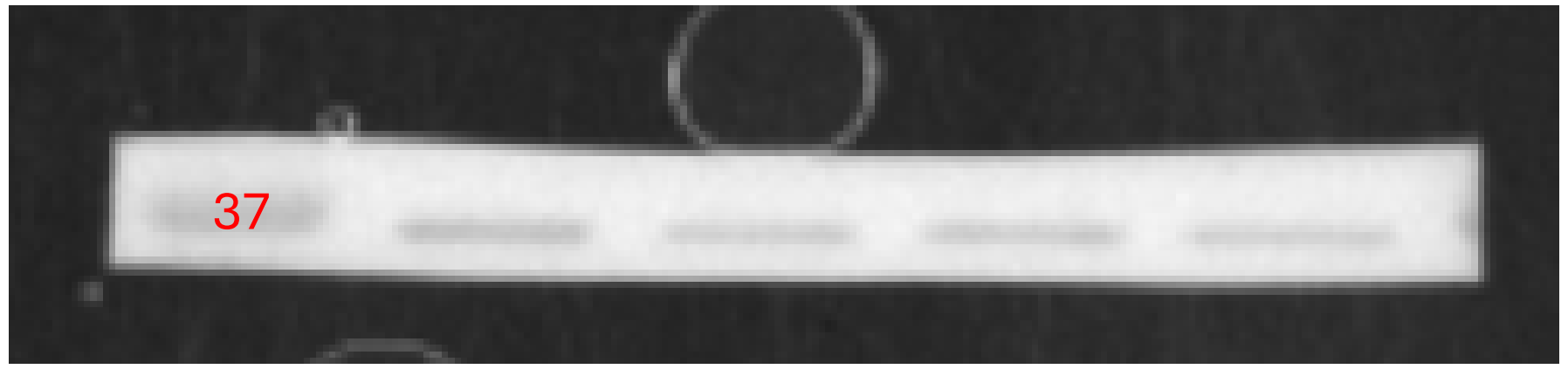

GFP (.25 sec exposure)

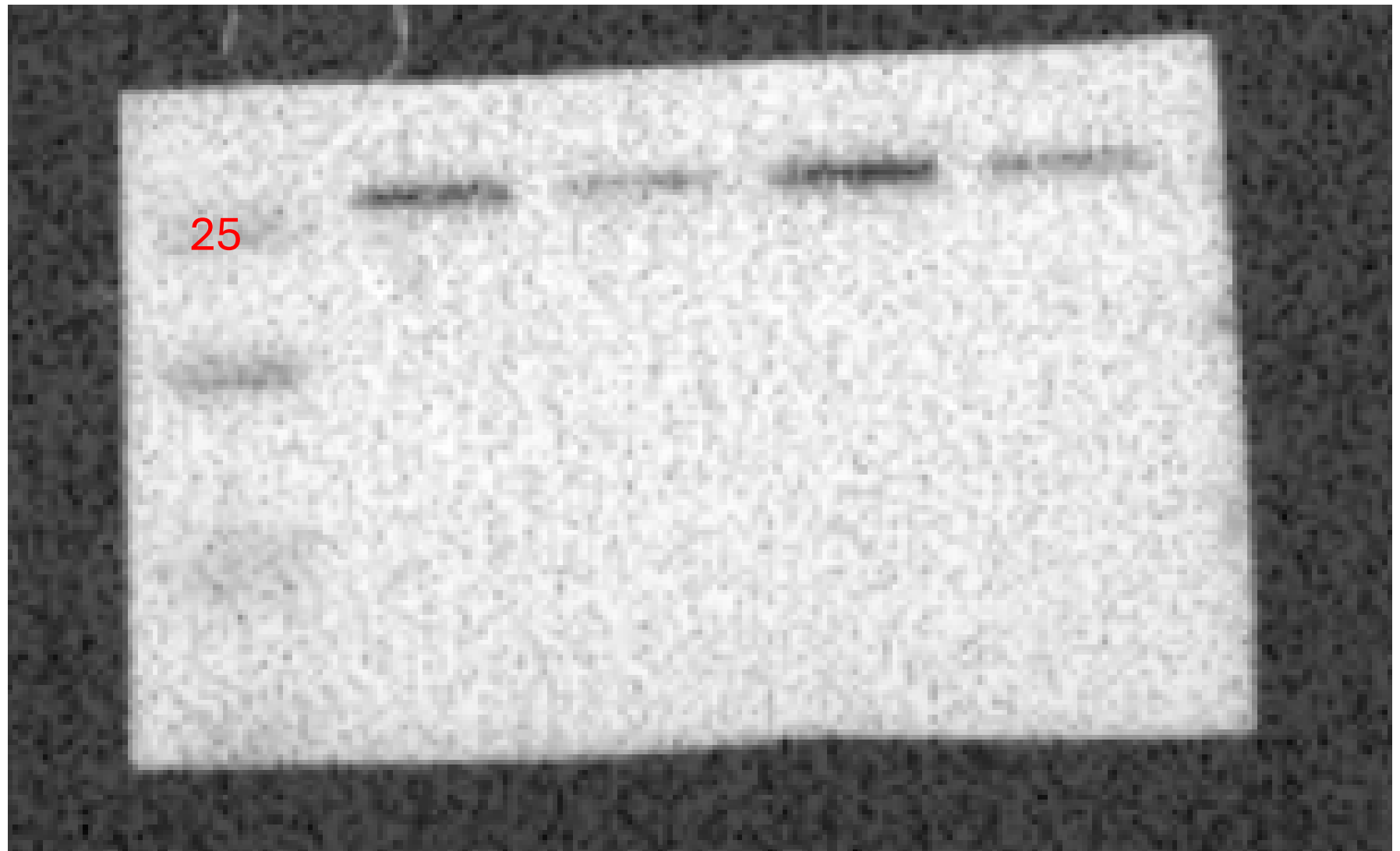

Lane	Sample
1	Ladder
2	Ctrl guide 2, Empty Vector
3	Ctrl guide 2, FLAG-PABPN1
4	PABPN1 guide 2, Empty Vector
5	PABPN1 guide 2, FLAG-PABPN1

Supplementary Figure 13B – rep 2

B-tubulin (4.1 sec exposure – accumulation mode)

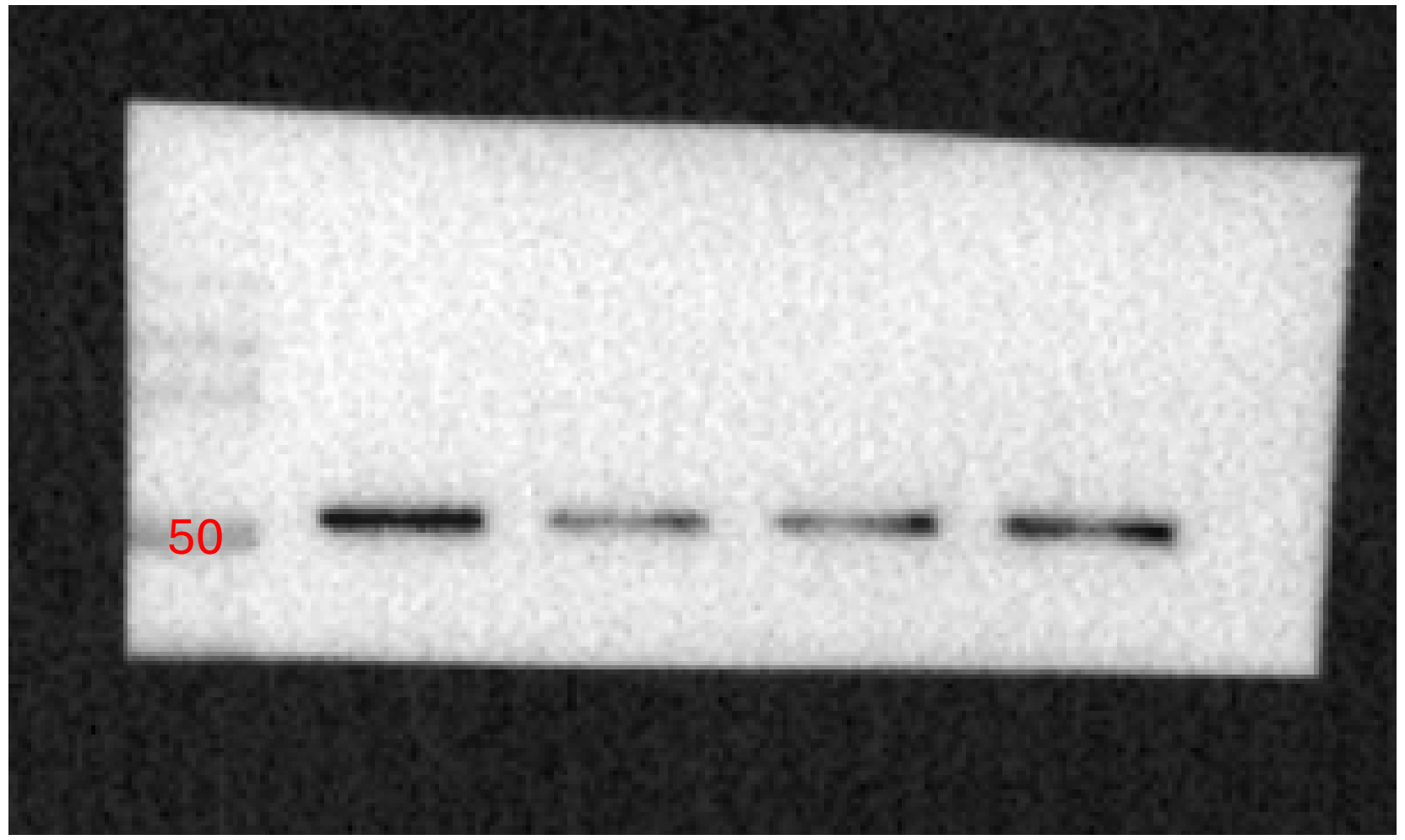

mCherry (25.8 sec exposure – accumulation mode, max substrate)

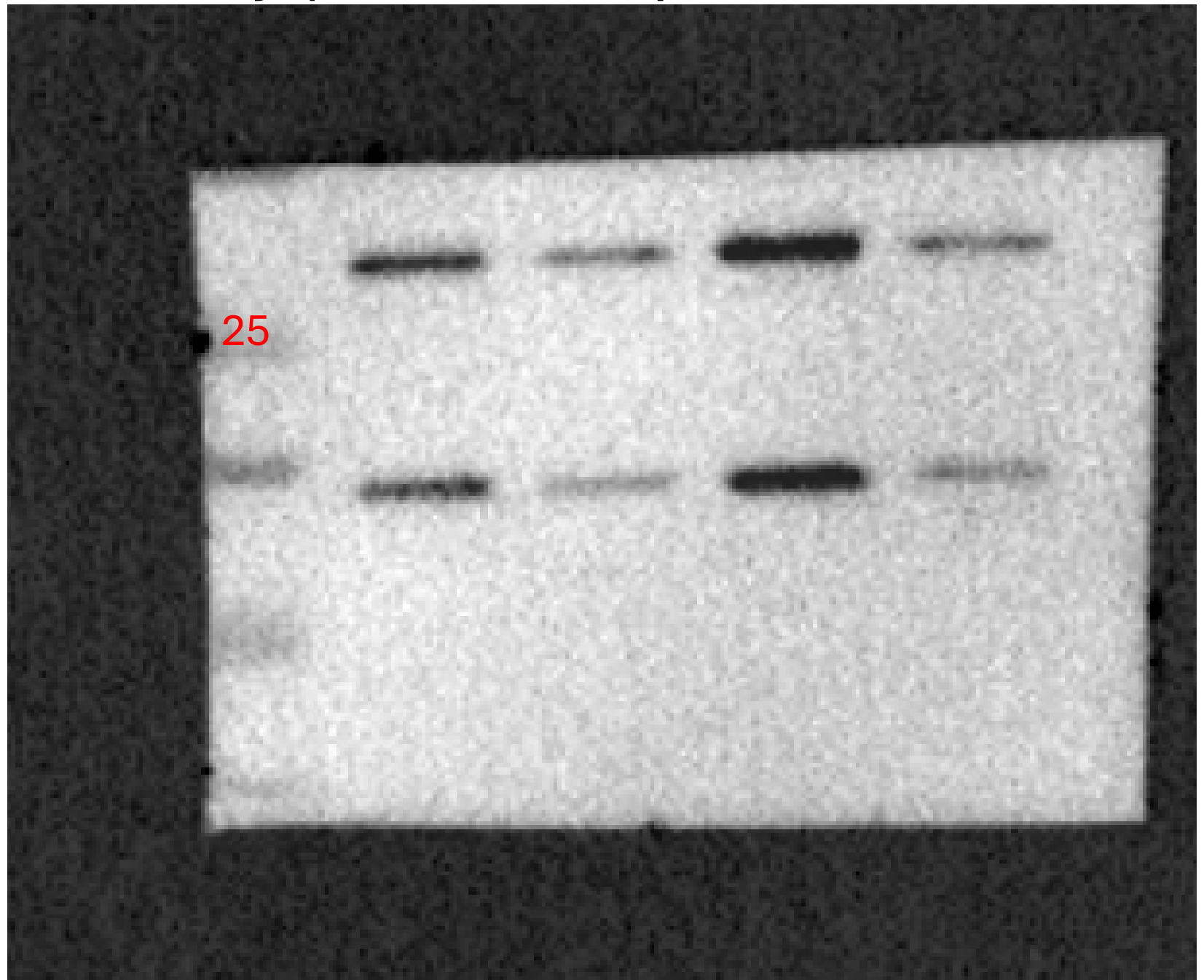

Supplementary Figure 14

hMTR4 (10.3 sec exposure – accumulation mode)

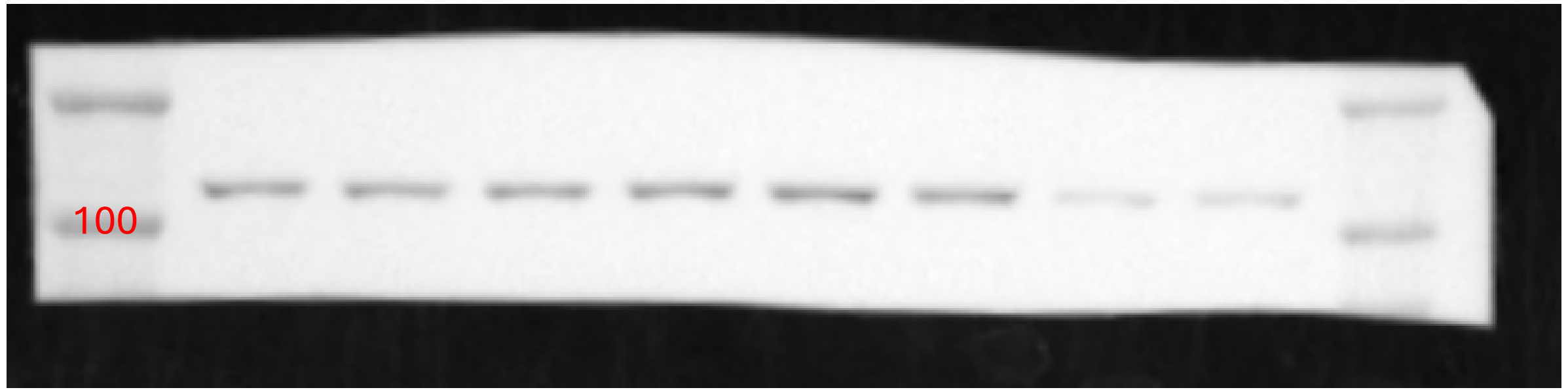

PABPN1 (1 sec exposure – accumulation mode)

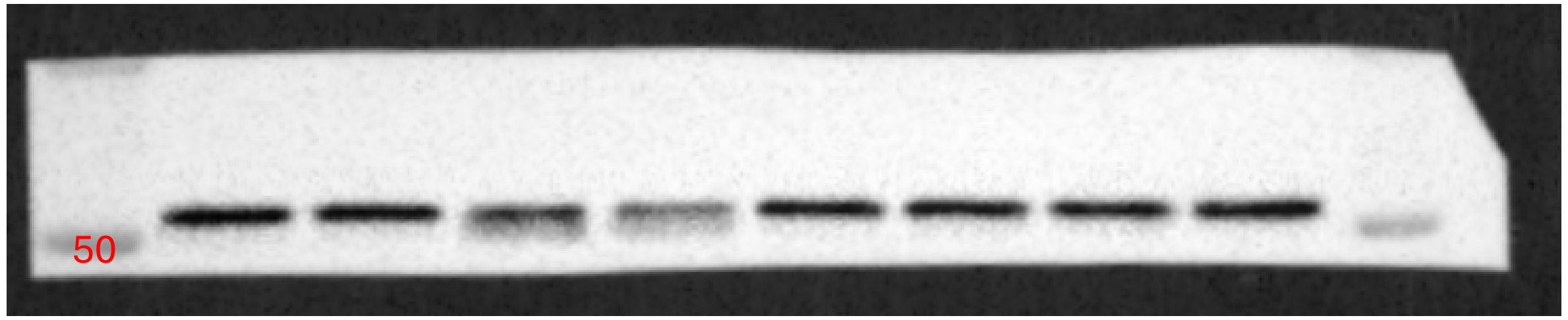

GAPDH (19.6 sec exposure – accumulation mode)

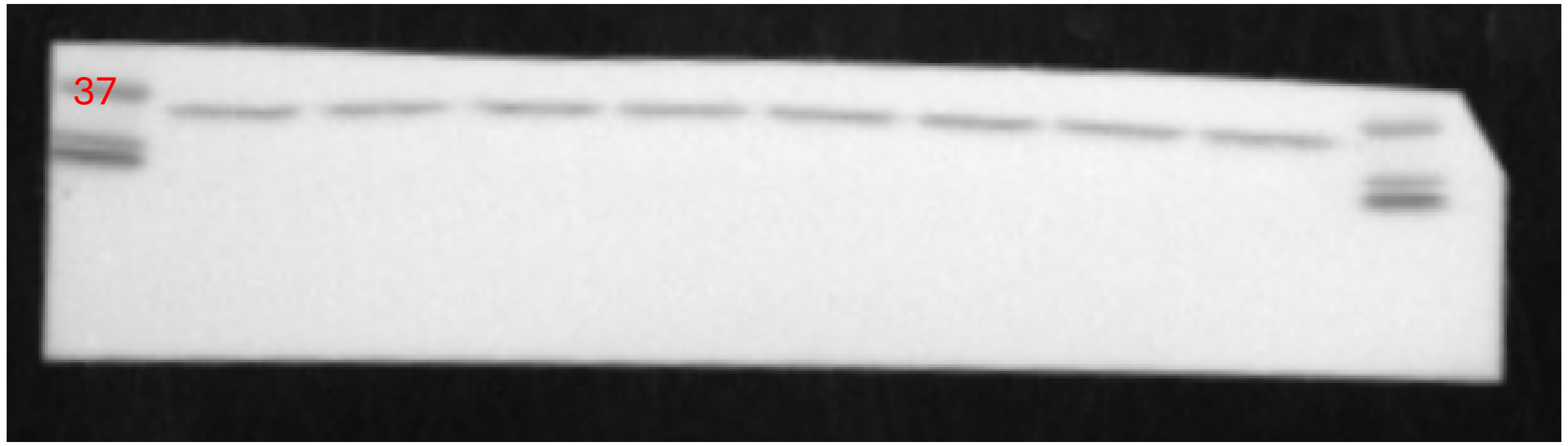

ZFC3H1 (28.9sec exposure – accumulation mode, max substrate)

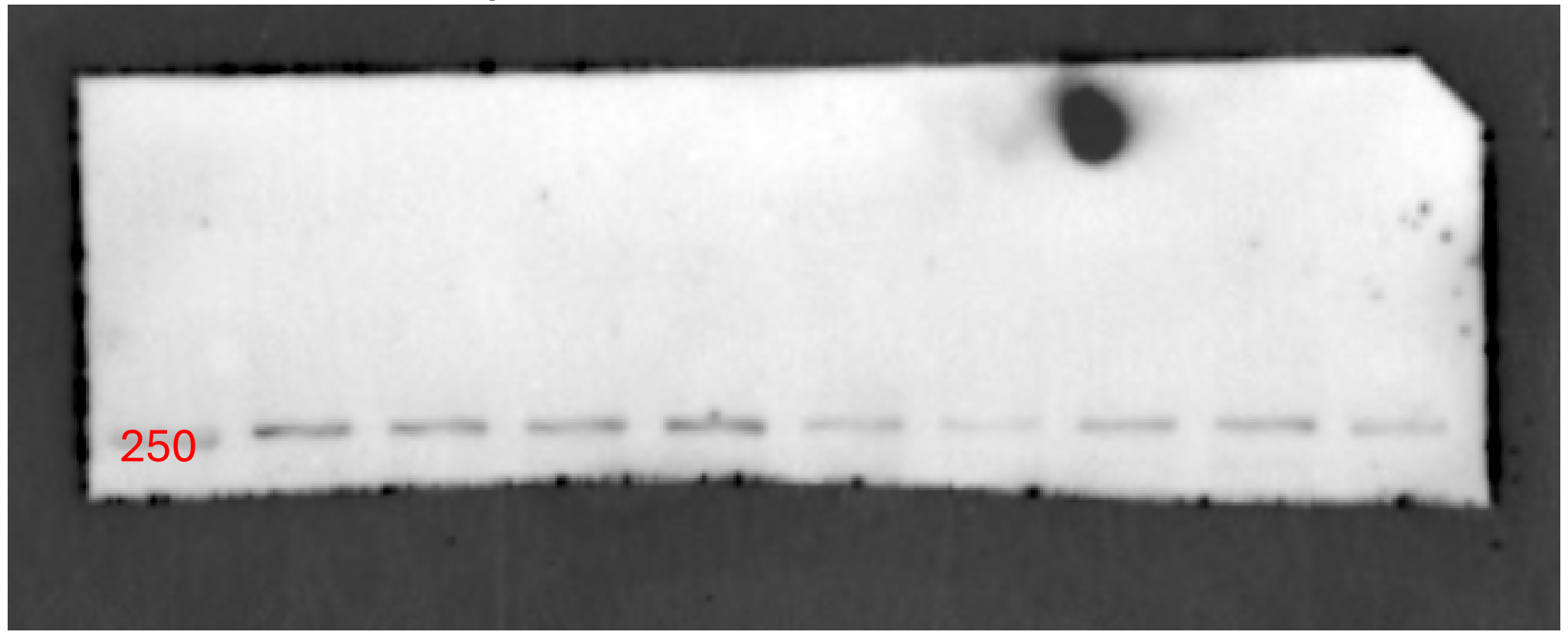

Lane	Sample
1	Ladder
2	Ctrl guide 1, Bio Rep 1
3	Ctrl guide 1, Bio Rep 2
4	PABPN1 guide 2, Bio Rep 1
5	PABPN1 guide 2, Bio Rep 2
6	ZFC3H1 guide 1, Bio Rep 1
7	ZFC3H1guide 1, Bio Rep 2
10	MTREX guide 1, Bio Rep 1
11	MTREX guide 1, Bio Rep 2
14	Ladder

Supplementary Figure 14 – Rep 2

ZFC3H1 (47.6 sec exposure – accumulation mode, max substrate)

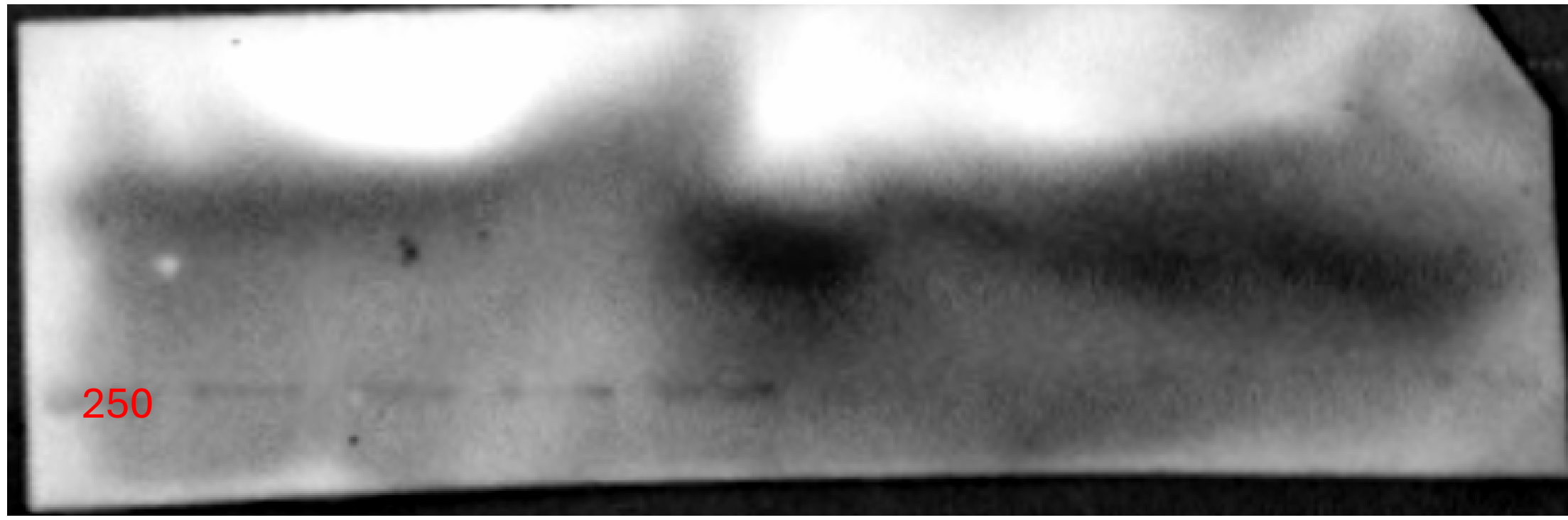

MTREX (38.3 sec exposure – accumulation mode)

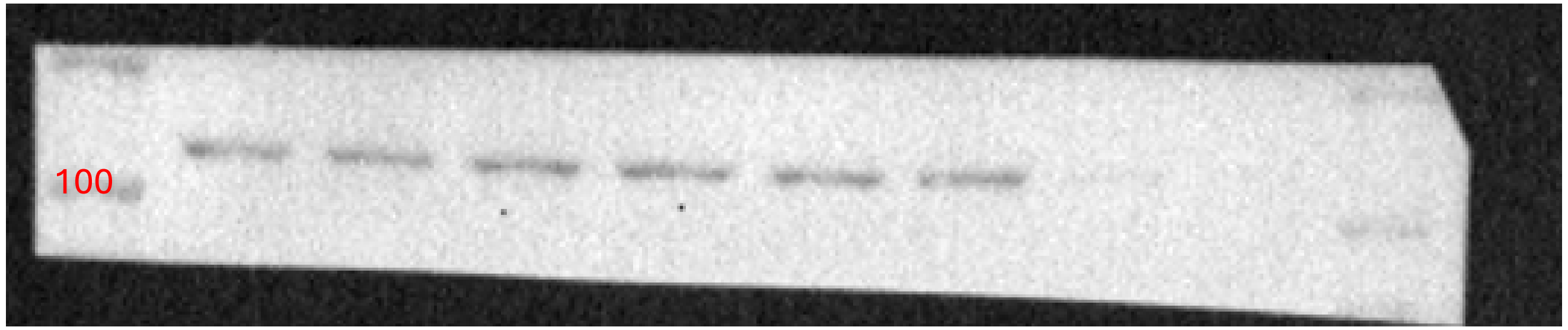

PABPN1 (1 sec exposure – accumulation mode)

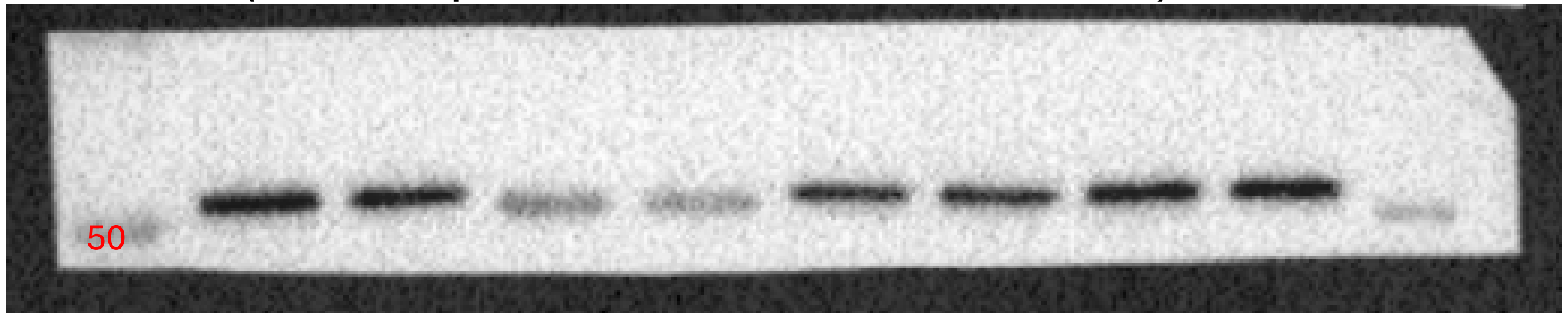

GAPDH (38.3 sec exposure – accumulation mode)

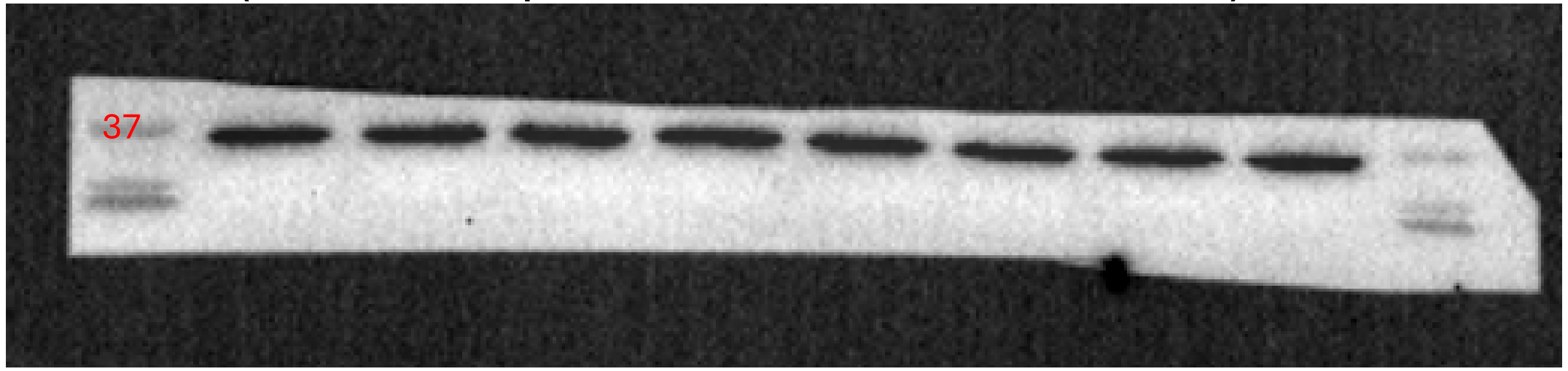

Lane	Sample
1	Ladder
2	Ctrl guide 1, Bio Rep 1
3	Ctrl guide 1, Bio Rep 2
4	PABPN1 guide 2, Bio Rep 1
5	PABPN1 guide 2, Bio Rep 2
6	ZFC3H1 guide 1, Bio Rep 1
7	ZFC3H1guide 1, Bio Rep 2
10	MTREX guide 1, Bio Rep 1
11	MTREX guide 1, Bio Rep 2
14	Ladder

Supplementary Figure 15

FLAG (44.5 sec exposure – accumulation mode, max substrate)

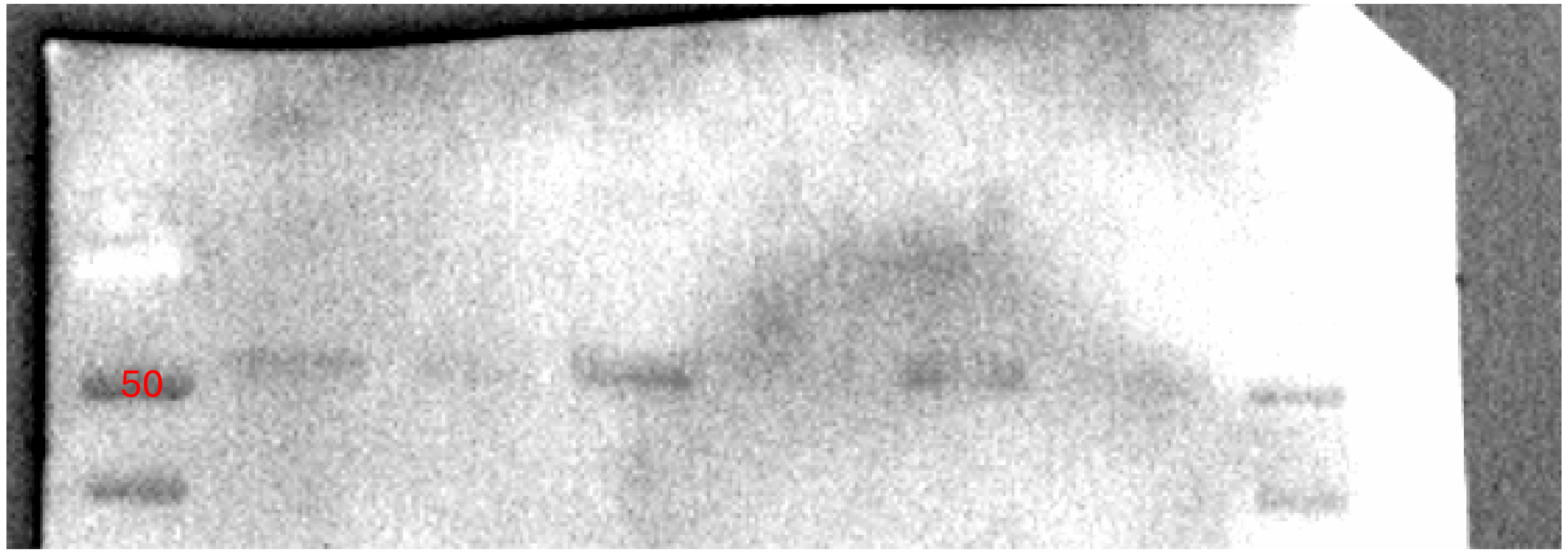

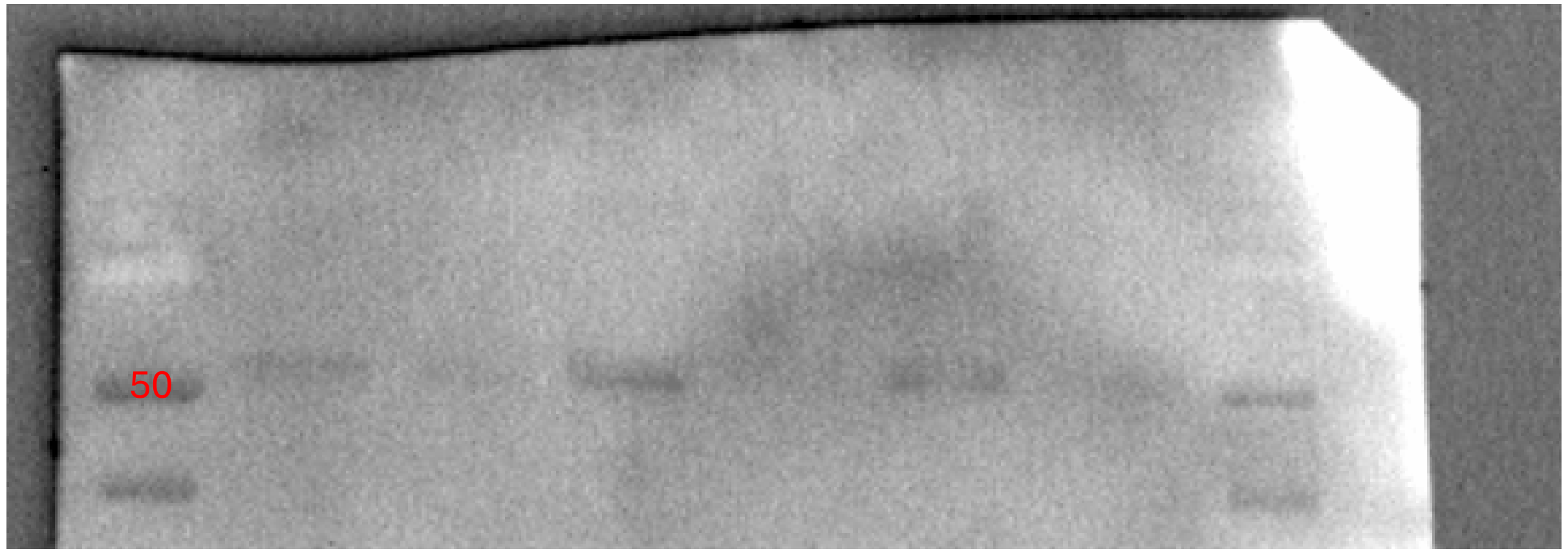

Lane	Sample
1	Ladder
2	FLAG rep 1
3	IgG rep 1
4	FLAG rep 2
5	IgG rep 2
6	FLAG rep 3
7	IgG rep 3
8	Ladder

FLAG (19.6 sec exposure – accumulation mode, max substrate)

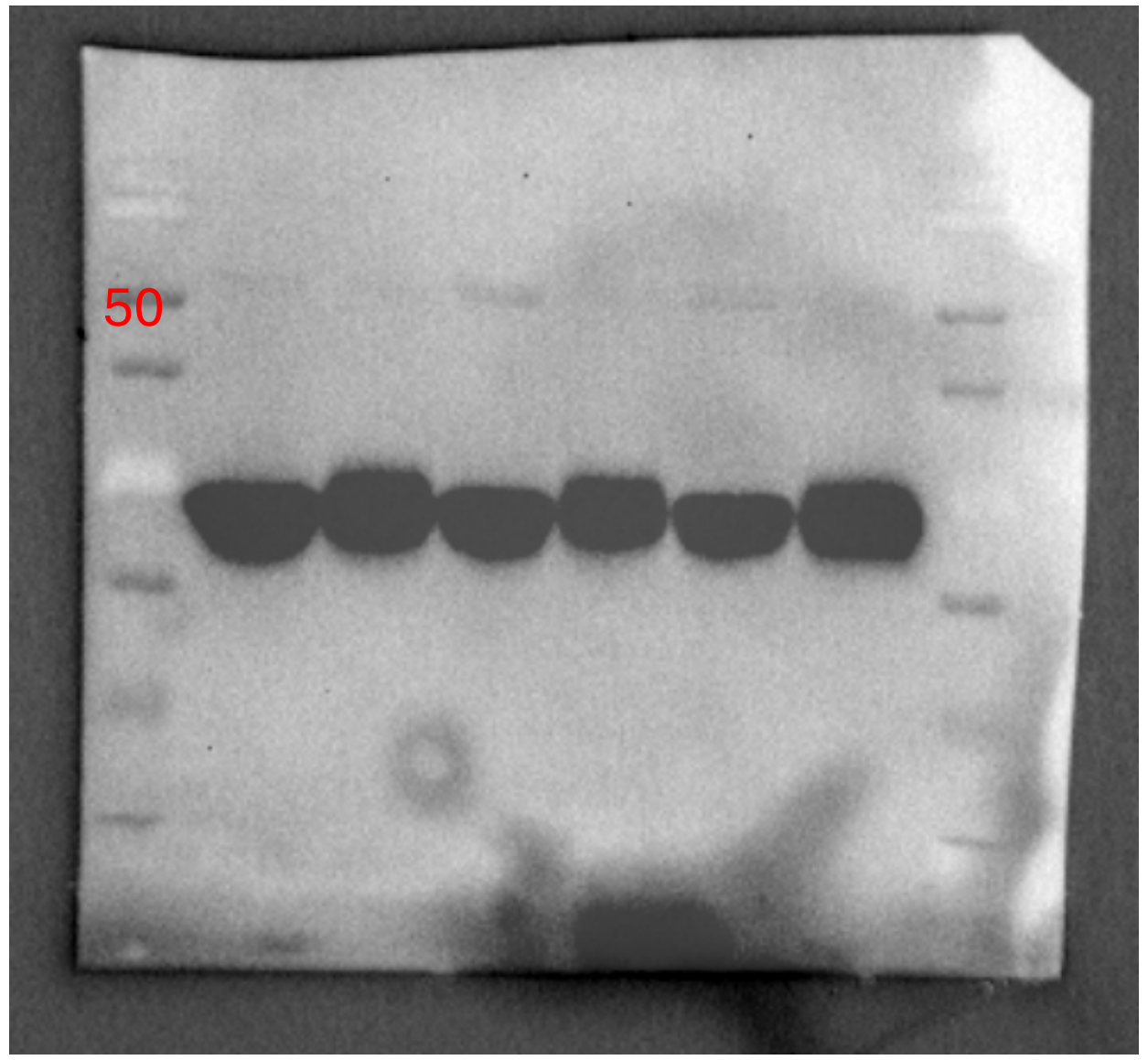

Supplementary Figure 16A

ZFC3H1 (7.2 sec exposure – accumulation mode, max substrate)

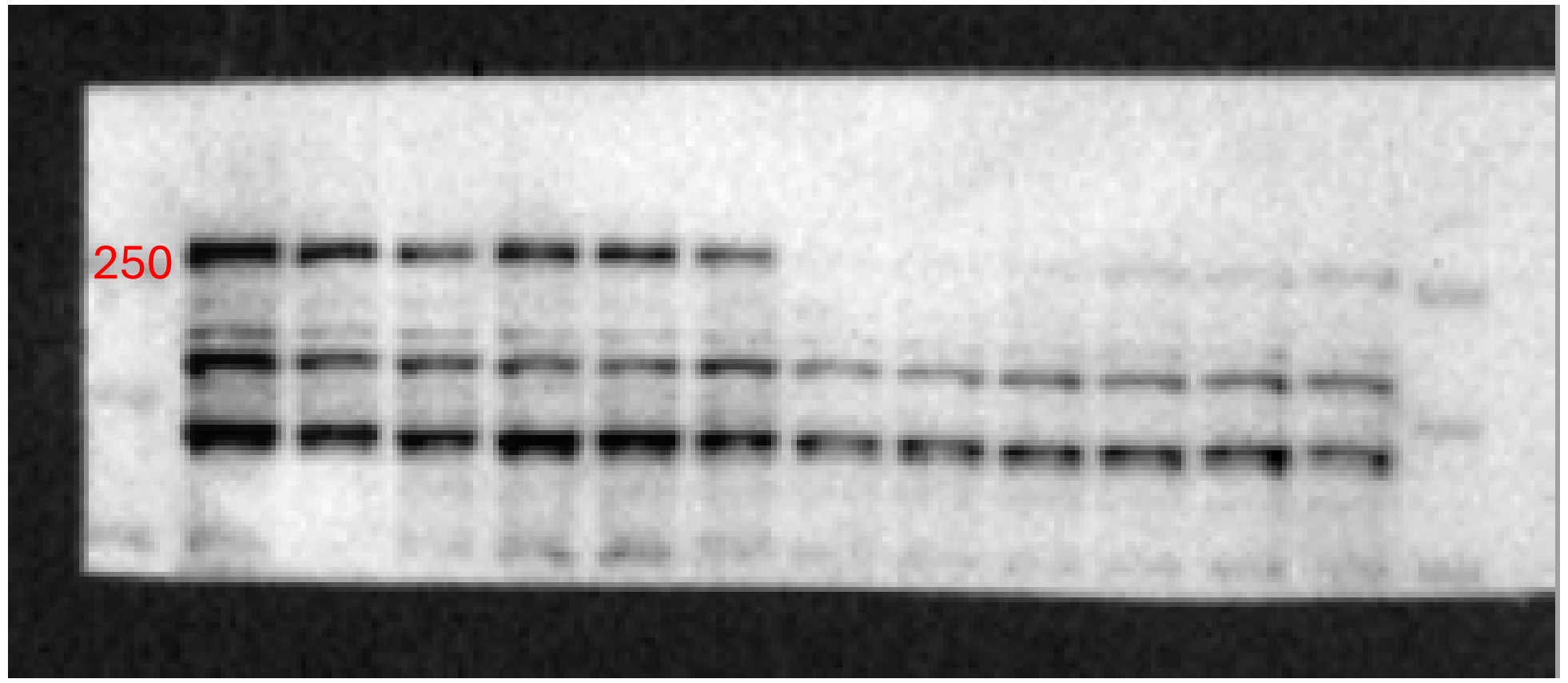

B-tubulin for ZFC3H1 (1 sec exposure – accumulation mode)

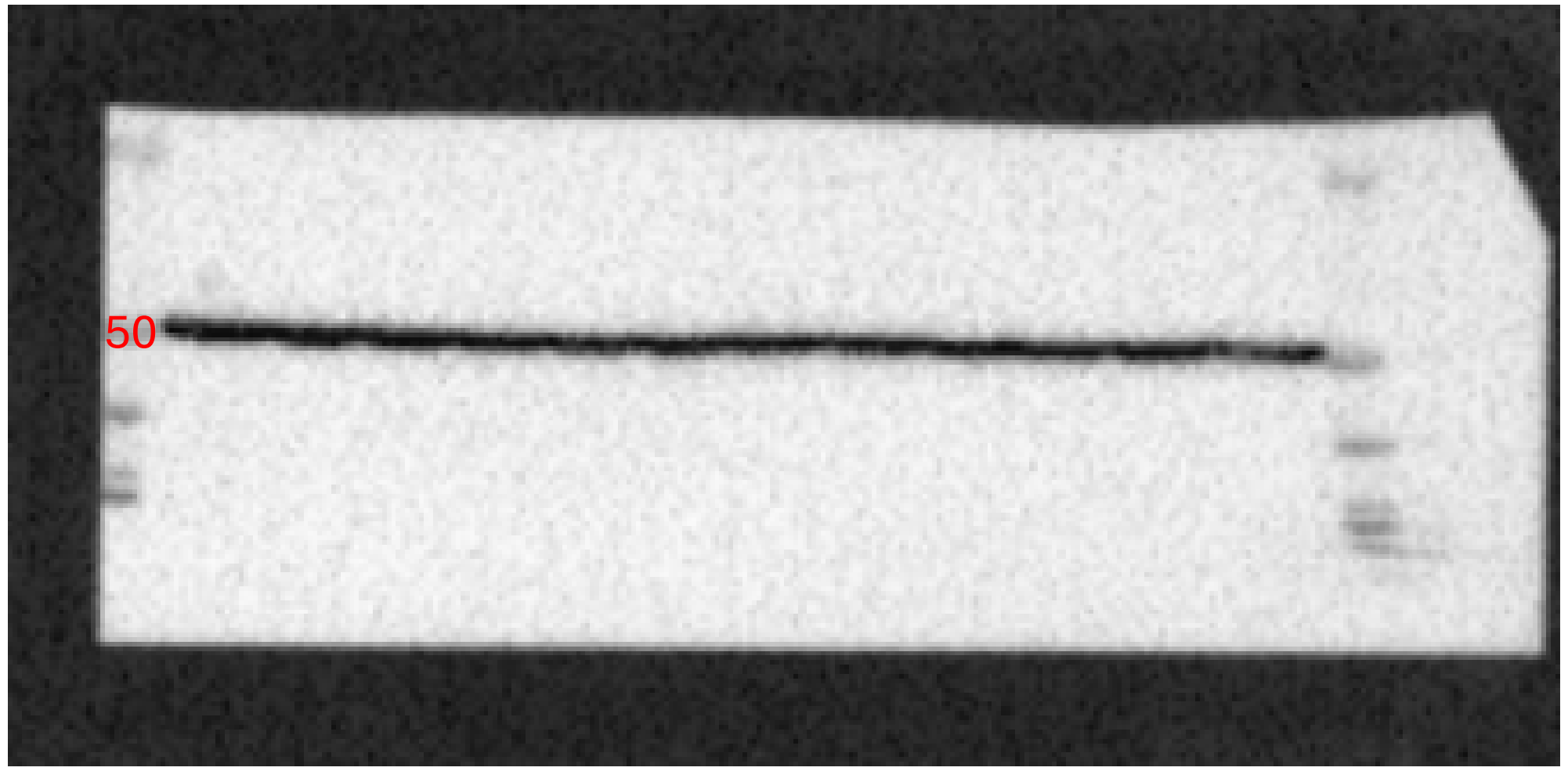

Lane	Sample
1	Ladder
2	Ctrl guide 1, Bio Rep 1
3	Ctrl guide 1, Bio Rep 2
4	Ctrl guide 1, Bio Rep 3
5	Ctrl guide 2, Bio Rep 1
6	Ctrl guide 2, Bio Rep 2
7	Ctrl guide 2, Bio Rep 3
8	ZFC3H1 guide 1, Bio Rep 1
9	ZFC3H1guide 1, Bio Rep 2
10	ZFC3H1 guide 1, Bio Rep 3
11	ZFC3H1 guide 2, Bio Rep 1
12	ZFC3H1guide 2, Bio Rep 2
13	ZFC3H1 guide 2, Bio Rep 3
14	Ladder

Supplementary Figure 16B

ZFC3H1 (7.2 sec exposure – accumulation mode, max substrate)

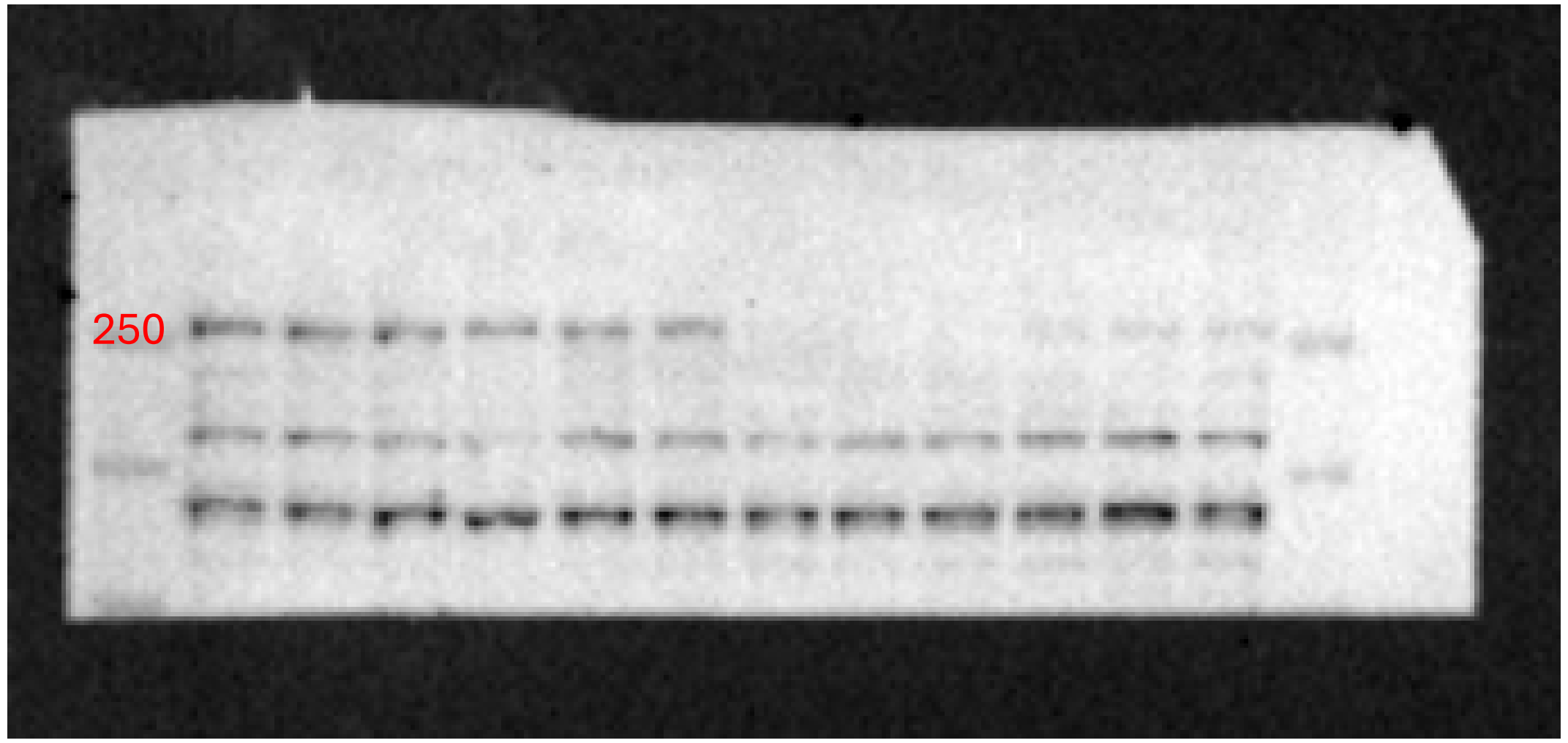

B-tubulin for ZFC3H1 (1 sec exposure – accumulation mode)

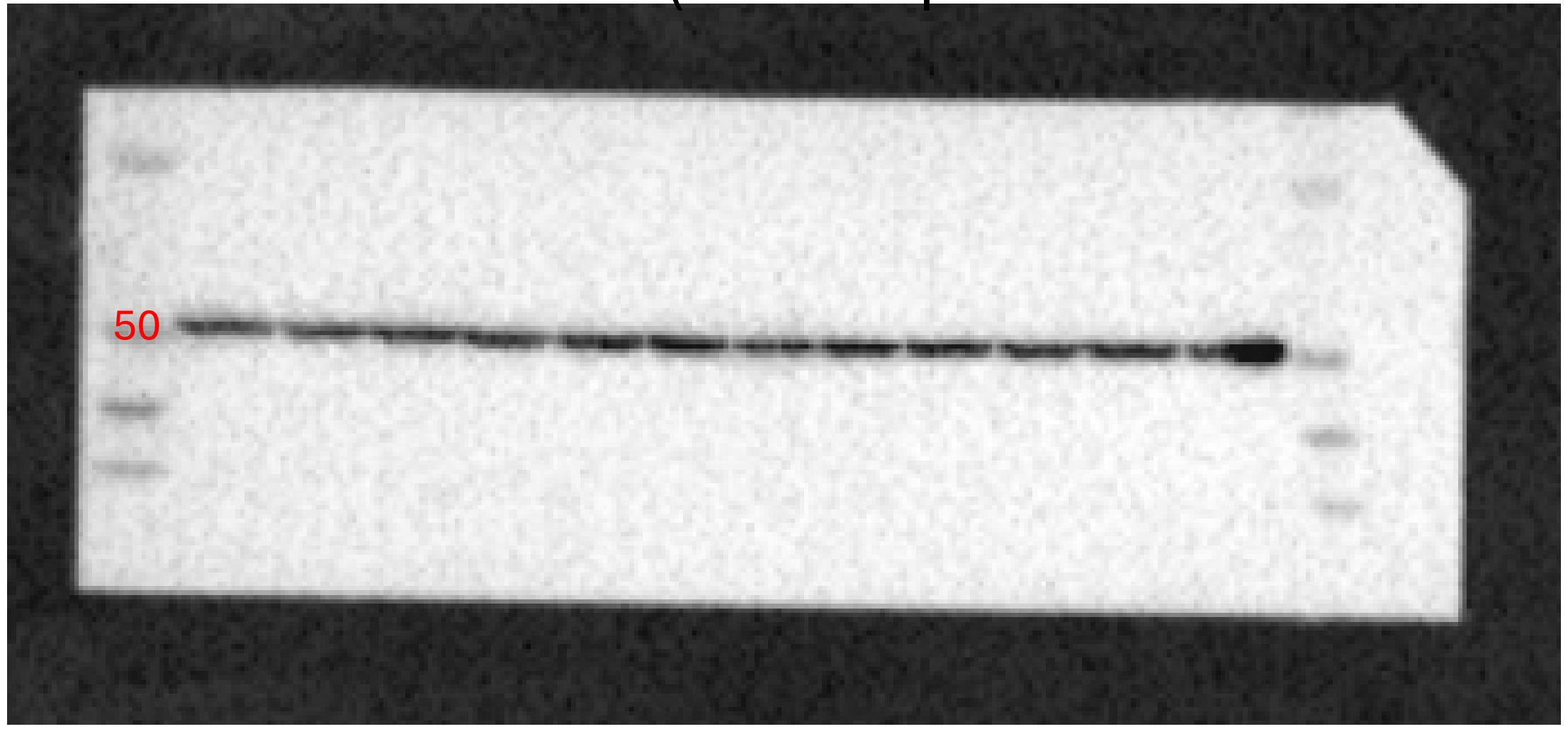

Lane	Sample
1	Ladder
2	Ctrl guide 1, Bio Rep 1
3	Ctrl guide 1, Bio Rep 2
4	Ctrl guide 1, Bio Rep 3
5	Ctrl guide 2, Bio Rep 1
6	Ctrl guide 2, Bio Rep 2
7	Ctrl guide 2, Bio Rep 3
8	ZFC3H1 guide 1, Bio Rep 1
9	ZFC3H1guide 1, Bio Rep 2
10	ZFC3H1 guide 1, Bio Rep 3
11	ZFC3H1 guide 2, Bio Rep 1
12	ZFC3H1guide 2, Bio Rep 2
13	ZFC3H1 guide 2, Bio Rep 3
14	Ladder

Supplementary Figure 18A

ZFC3H1 (16.5 sec exposure – accumulation mode, max substrate)

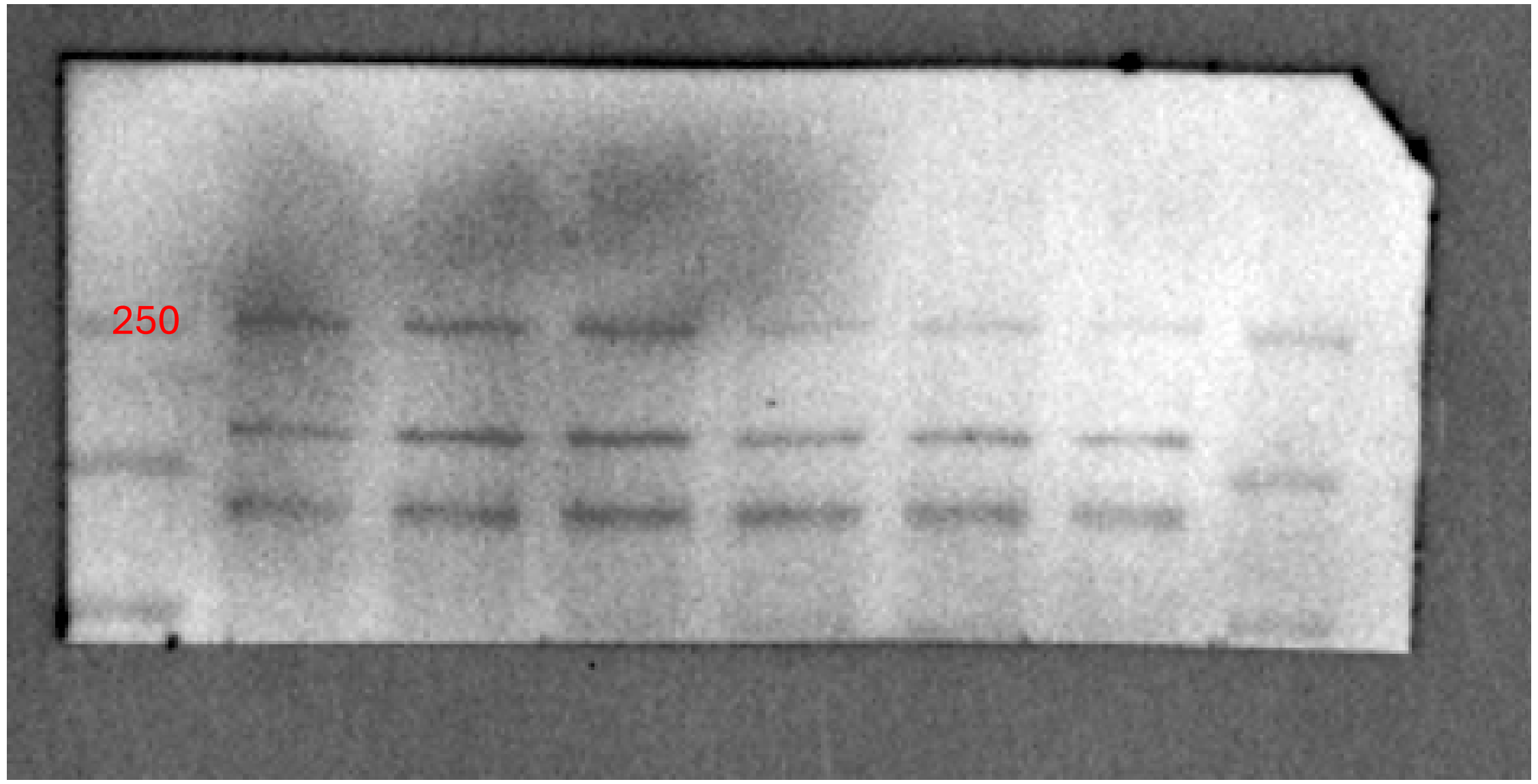

B-tubulin for ZFC3H1 (4.1 sec exposure – accumulation mode)

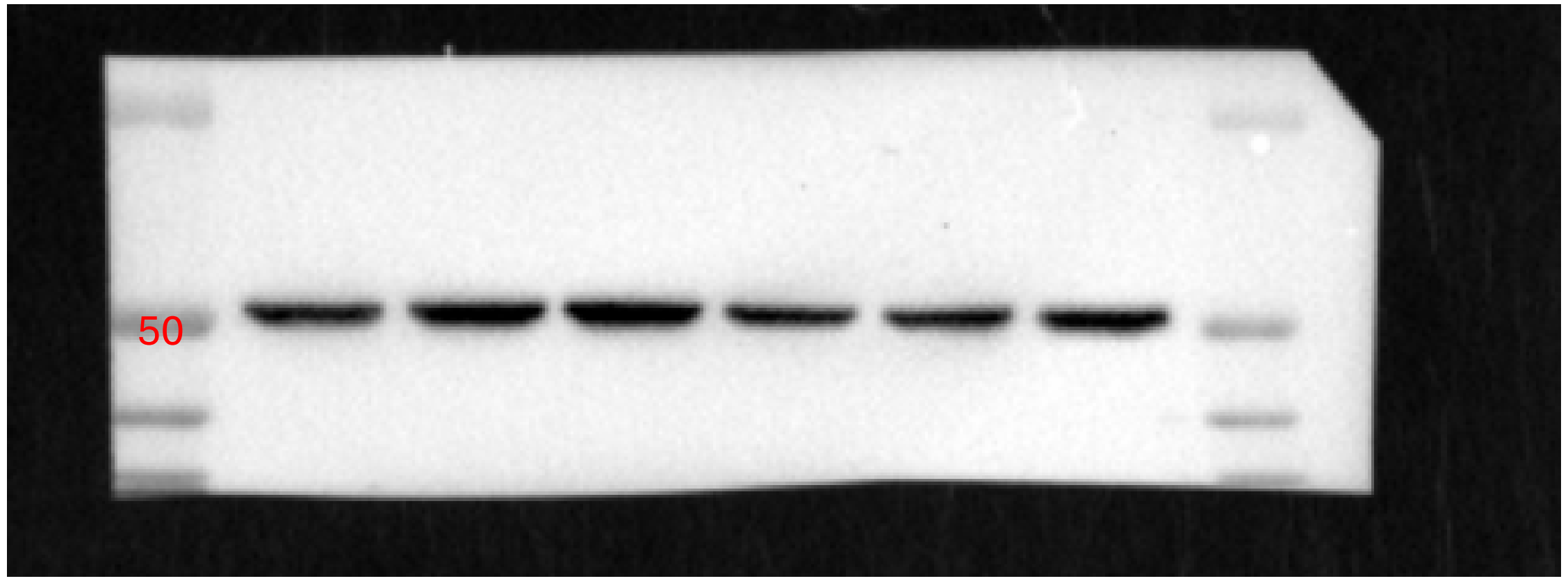

Lane	Sample
1	Ladder
2	Ctrl guide 1
3	Ctrl guide 1
4	Ctrl guide 2
5	ZFC3H1 guide 1
6	ZFC3H1 guide 1
7	ZFC3H1 guide 2
8	ladder
